# Rethinking the history of common walnut (*Juglans regia* L.) in Europe: Its origins and human interactions

**DOI:** 10.1371/journal.pone.0172541

**Published:** 2017-03-03

**Authors:** Paola Pollegioni, Keith Woeste, Francesca Chiocchini, Stefano Del Lungo, Marco Ciolfi, Irene Olimpieri, Virginia Tortolano, Jo Clark, Gabriel E. Hemery, Sergio Mapelli, Maria Emilia Malvolti

**Affiliations:** 1 Institute of Agro-environmental and Forest Biology, National Research Council, Porano, Terni, Italy; 2 U.S.D.A. Forest Service, Hardwood Tree Improvement and Regeneration Center, Department of Forestry and Natural Resources, Purdue University, West Lafayette, Indiana, United States of America; 3 The Institute of Archaeological and Monumental Heritage, National Research Council, Tito Scalo, Potenza, Italy; 4 Earth Trust, Little Wittenham, Abingdon, Oxfordshire, United Kingdom; 5 Sylva Foundation, Little Wittenham, Oxfordshire, United Kingdom; 6 Institute of Agricultural Biology and Biotechnology, National Research Council, Milan, Italy; National Cheng Kung University, TAIWAN

## Abstract

Common walnut (*Juglans regia* L) is an economically important species cultivated worldwide for its high-quality wood and nuts. It is generally accepted that after the last glaciation *J*. *regia* survived and grew in almost completely isolated stands in Asia, and that ancient humans dispersed walnuts across Asia and into new habitats via trade and cultural expansion. The history of walnut in Europe is a matter of debate, however. In this study, we estimated the genetic diversity and structure of 91 Eurasian walnut populations using 14 neutral microsatellites. By integrating fossil pollen, cultural, and historical data with population genetics, and approximate Bayesian analysis, we reconstructed the demographic history of walnut and its routes of dispersal across Europe. The genetic data confirmed the presence of walnut in glacial refugia in the Balkans and western Europe. We conclude that human-mediated admixture between Anatolian and Balkan walnut germplasm started in the Early Bronze Age, and between western Europe and the Balkans in eastern Europe during the Roman Empire. A population size expansion and subsequent decline in northeastern and western Europe was detected in the last five centuries. The actual distribution of walnut in Europe resulted from the combined effects of expansion/contraction from multiple refugia after the Last Glacial Maximum and its human exploitation over the last 5,000 years.

## Introduction

Common walnut (*Juglans regia* L.) is an economically important tree species, highly valued for its timber and edible nuts. This species grows well in virtually all parts of the world with a temperate climate [1]. Its ancient history of cultivation and widespread use throughout much of Eurasia, from China to western Europe, and the relative scarcity of wide-scale molecular phylogeographic studies, made accurate determination of its native geographic range difficult. New data and resources, however, including the development of highly informative molecular markers in *Juglans* spp. such as SSR markers [2], the recent advent of landscape genetics [3], the persistence of centuries-old walnut trees in natural reserves isolated from plantations, access to remote uncultivated Asian walnut populations [4] and a rich fossil pollen record in Europe [5], provide the opportunity to resolve the complex human and natural interactions that shaped the evolutionary history of walnut since the Holocene in Eurasia.

The history of walnut in Asia has emerged as a complex interaction of biogeographic and human forces. After the Last Glacial Maximum (LGM), *J*. *regia* survived and grew spontaneously in almost completely isolated stands in Asia, from Xinjiang province of western China through central Asia to the Caucasus. Barriers to gene flow, such as the Tien Shan and Himalaya mountains, and the progressive desertification of central Asia during the Holocene promoted the fragmentation and isolation of natural *J*. *regia* populations in these regions [4]. Genetic analysis combined with ethno-linguistic and historical data revealed that what appeared to be native walnut stands were actually the result, at least in part, of ancient human efforts to modify the Asian landscape [6]. Humans, trading walnut along "green corridors” such as the Silk Roads and the Persian Royal Road, overcame geographic barriers and facilitated walnut exchanges across Asia [6–7]. The origin and human-mediated expansion of walnut across Europe, however, is a matter of long-standing debate.

Fossil pollen deposits clearly indicate that *J*. *regia* was growing in southern Spain, Italy, France, Switzerland (Alps), Bulgaria (Rhodopes Mountains), Greece (Epirus), southwestern Turkey and Albania during the Upper Pleistocene (126,000–12,000 BP) (S1 Table and S1 Video). Considering the scarce remains of walnut pollen in the European post-Eemian sequences, several authors proposed that *J*. *regia* essentially disappeared in Europe at the onset of the Holocene (~ ≥11,700 BP), after the Last Glacial Maximum [8] and its human-mediated reintroduction into Europe from western Asia (e.g. eastern Turkey and Trans Caucasus) to the Aegean basin occurred not before the middle of the fourth millennium BP [9]. A second wave of migration into southwestern Europe followed the Greek colonization around the 8^th^ to 5^th^ centuries BCE (2800–2500 BP) [10–11]. This view is being challenged now. Subsequent palynological finds demonstrated that *J*. *regia* may have survived the cold, dry glacial intervals in refugia located in southern Europe [12–14] and the Balkans [15–18] (S1 Video). Carpological fossils of walnut have been found in northeastern Italy (Sammardenchia, 7,550 BP) [19], Switzerland (near Lake Constance, ~ 6,000–4,350 BP) [20] and Slovenia (Hocevarica, 5,600–5,500 BP) [21]. Assuming walnut was harvested/cultivated from the early Neolithic to Bronze Age in Europe [13], we can’t rule out the use of local stands, formerly protected for their fuel and food during times of dramatic land transformations due to global climate oscillations and growing economic pressures [22–23]. Despite information from the fossil record, there is no molecular evidence that *J*. *regia* survived the LGM in Europe as no polymorphism was detected in 29 demes of walnut in Eurasia using chloroplast PCR-RFLP markers [24].

Irrespective of the resolution of the conflict between fossil and molecular evidence, the distribution of *J*. *regia* in Europe was surely modified by human management over the last 2,500 years. Sudden increases in *J*. *regia* fossil pollen curves were recorded in the time window between 2500 and 1000 years BP, presumably reflecting the widespread increase of walnut cultivation from Greek and Roman times onwards [11] (S1 Video). As detected in Italy, the selection and frequent inter-regional transfer of walnut seeds along ancient routes probably affected the spatial genetic structure of *J*. *regia*, decreasing its diversity by selection and increasing its genetic homogeneity by dispersal [25–26]. Nevertheless, despite long-term scientific interest, a comparative and full-scale overview of the genetic resources of walnut in Europe has never been undertaken, and neither the putative dispersal routes of walnut across Europe nor the time-scales over which such processes occurred since the Late Holocene have been studied using molecular phylogeny.

Here, we report on a large-scale study of the spatial genetic structure of walnut populations in Europe using microsatellite markers, and its comparison with autochthonous walnut populations sampled in Asia. Our objective was the integration of fossil pollen, cultural and historical data with population genetics (the landscape genetic overlay approach) [27], and the inference of demographic history using approximate Bayesian computation (ABC) [28] to evaluate if (1) ancient reservoirs of walnut diversity still exist in Europe, (2) clines of genetic diversity are present between Europe and Asian regions where walnut still grows naturally, (3) genetic clines in *J*. *regia* can be explained by the recolonization of western Europe from refugia of western Asia through the Balkans, or (4) whether western Asia and the southern European/Balkans peninsulas represent two separate regions of walnut evolution. In addition, we evaluated the hypothesis that cultural/historical factors played a key role in shaping the genetic resources of walnut in Europe since the Late Holocene.

## Materials and methods

### Common walnut collection

Over the last two decades, the CNR-Institute of Agro-environmental and Forest Biology (IBAF, Porano, Italy), CNR-Institute of Agricultural Biology and Biotechnology (Milan, Italy) and the Earth Trust (Oxfordshire, UK) have monitored and sampled common walnut trees in Europe and across the species’ range in Asia. Although we were unable to obtain samples from all areas, we assembled a large and unique collection of *J*. *regia*, including 40 Asian autochthonous walnut populations sampled from China (6 populations), Kyrgyzstan (9), Uzbekistan (17), Tajikistan (1), Pakistan (2), Iran (1), Turkey (2) and Georgia (2) which have already been genotyped [4, 6] and 51 European walnut populations sampled from Greece (4), Romania (1), Moldova (1), Hungary (10), Slovakia (1), Spain (1), France (4) and Italy (29), for a total of 91 populations and 2,008 genotypes (S2 Table, S1 Fig). These walnut populations span thirteen mountain systems in Eurasia (Tien Shan, Gissar, Zaamin, Nurata, Pamir, Himalayas, Alborz, Trans-Caucasus, Balkans, Carpathians, Alps, Pyrenees, and Apennines) (S1 Fig). Only centuries-old trees in ancient forests or nature reserves were sampled in Asia. In Europe, we carefully avoided collecting walnut samples from plantations or non-protected areas (S2 Table). Sampled trees in each population were separated by > 100 metres. Mature leaves were sampled from each walnut tree, dehydrated, and then stored at −80°C at CNR-IBAF (Porano, Italy).

### Microsatellite analysis

Dehydrated leaf tissue (60 mg) from each European walnut sample was homogenized in a 2ml micro-centrifuge tube containing a 5 -mm steel bead cooled with liquid nitrogen using a Mixer Mill 300 (Qiagen, Hilden, Germany). Genomic DNA was extracted and purified using a DNeasy96 Plant Kit (Qiagen), and stored at -20°C.

In this study, all European samples were genotyped using 14 unlinked nuclear, neutral microsatellite (SSR) markers (WGA1, WGA4, WGA9, WGA27, WGA32, WGA69, WGA72, WGA79, WGA89, WGA118, WGA202, WGA276, WGA321, WGA331) already selected and used for genetic characterization of walnut in its Asian range [4, 6]. PCR amplification and the visualization of amplified SSR alleles for each sample were carried out as described by Pollegioni et al [26]. The allele size scoring was performed using GeneMapper version 4 (Applied Biosystems, Foster City, CA, USA).

### Data analysis

#### Genetic diversity of walnut populations

Standard measures of genetic diversity, total number of observed alleles (*A*), effective number of alleles (*A*_e_), observed (*H*_O_) and expected (*H*_E_) heterozygosity and polymorphic information content (*PIC*), the three unbiased estimators of Wright’s *F*-statistics, within-population inbreeding coefficient *f* (*F*_IS_), total-population inbreeding coefficient F (*F*_IT_) and among-population genetic differentiation coefficient *θ*(*F*_ST_) and the unbiased estimator of Jost’s (*D*_est_) [29] were computed for each locus and over all loci as described by Pollegioni et al [4]. Similarly, allele dropout and null alleles were tested for each locus using FreeNa software [30].

Levels of genetic diversity were estimated for each walnut population across all loci in terms of the mean number of alleles per locus (*A*), observed (*H*_o_) expected (*H*_E_) and unbiased expected heterozygosity (*UH*_E_) using the GenAlEx software 6.3 [31]. To account for difference in sample size, allelic richness (*Rs*) and private allele richness (*PAR*) were computed by the rarefaction method with HP-Rare software [32]. The estimates of *Rs* and *PAR* were standardized on a minimum sample size of eight individuals. Following the procedure of Pollegioni et al [4], the Inverse Distance Weighted (IDW) interpolation function implemented in the Geographic Information System (GIS) software ArcGIS 9.3 (ESRI, Redlands, Calif. USA) was used to display the geographic patterns of allelic richness (*Rs*) and unbiased expected heterozygosity (*UH*_E_) computed for all 91 walnut populations. The within-population inbreeding coefficient *F*_IS_ [33] was estimated for each population using hierarchical locus-by-locus AMOVA as implemented in Arlequin 3.11 software [34]. The statistical significance of *F*_IS_ was tested using a non-parametric approach with 1,000 permutations. To determine whether within-population genetic variation was correlated with geographic gradients, we performed a simple linear regression analysis followed by the sequential Bonferroni correction of allelic richness (*Rs)* and unbiased expected heterozygosity (*UH*_E_) against three geographic variables, latitude, longitude and elevation of sampled sites.

Evidence of recent population size decrease was investigated using the program BOTTLENECK 1.2.02 [35]. As described by Pollegioni et al [4], significance was assessed using the ‘Wilcoxon’s signed-rank’ test, which provides relatively high power and can be used with as few as four polymorphic loci and any number of individuals. Bottleneck tests were conducted under Two-Phase Model (TPM) of evolution which most accurately reflected the mutational mechanism of the microsatellite loci used in this study [26]. As recommended by Piry et al [35], we used the TPM with 70% Stepwise Mutations Model (SMM) and 30% multistep mutations. For each mutational model, 10,000 replicates were performed. Nevertheless, as reported by Henry et al [36], the heterozygosity excess test is sensitive to very recent disturbances. Two additional tests were used to identify bottleneck signatures from larger time scales: shifted allele distribution analysis [37] and the M-ratio test [38] implemented in BOTTLENECK and Arlequin software, respectively [4].

#### Genetic structure analysis of common walnut populations

Two complementary statistical approaches were used to detect the genetic structure of walnut populations in Eurasia. First, a Bayesian clustering approach implemented in STRUCTURE software 2.3.3 [39] was applied as described by Pollegioni et al [6]. We reconstructed the underlying genetic structure of walnut populations and computed the proportion of membership (*Q*-value) for each predefined population and each individual multilocus genotype in each of the inferred clusters using Markov Chain Monte Carlo (MCMC) simulations. STRUCTURE was run assuming a pre-assigned number of clusters (K) ranging from 1 to 90, the admixture model on the whole dataset without a priori population information and with the correlated allele frequencies between populations option. Based on initial results, a series of six independent runs was performed for K between 1 and 12 with a burn-in period of 100,000 steps followed by 10^6^ MCMC replicates. The final posterior probability of *K*, *Ln P(K)*, and Δ*K*, the rate of change of *Ln P(K)* between successive *K* values [40], was calculated to detect the most likely number of populations. Therefore, the groups inferred by the first STRUCTURE analysis were subsequently reprocessed separately in order to identify possible sub-structure (sub-clusters). The six runs from the most probable number of clusters were averaged applying the FullSearch algorithm provided by CLUMPP software 1.1.2. [41]. The corresponding *Q*-matrices were graphically displayed by DISTRUCT software [42]. After determining the most probable number of clusters, an arbitrary threshold of Q ≥ 0.75 was used to assign populations and/or genotypes to one group. Populations with 0.25 < Q < 0.75 were classified as admixed. Following the procedure of Pollegioni et al [6], we derived K continuous clustering surfaces by interpolation of the population membership *Q*-values for the K clusters estimated from STRUCTURE using the IDW function implemented in ArcGIS 9.3. A synthetic map representing the genetic structure of walnut in Eurasia was obtained by overlaying the computed K clustering surface maps. We combined multiple K interpolated raster bands in a single multiband raster dataset by the Composite Bands function implemented in ArcGIS 9.3. The integrated use of the Composite Bands-tool and RGB color code allowed us to display the inferred genetic clusters of *J*. *regia* populations.

Finally, to confirm the genetic repartition of walnut populations inferred by STRUCTURE, a Neighbor-Joining phylogenetic tree was constructed based on [43] genetic distance using POPTREE2 software [44] and drawn using FigTree software (http://tree.bio.ed.ac.uk/software/figtree/).

#### Reconstruction of postglacial dispersal routes of walnut in Europe

The demographic history of walnut and its human-mediated postglacial dispersal routes in Europe were further explored using the approximate Bayesian computation (ABC) procedure originally introduced by Beaumont et al [28] and recently implemented in DIYABC v. 2.0.4 software [45].

Due to computational limitations and the complexity of possible scenarios, we split DIYABC analysis into two stages and we pooled a subset of Eurasian walnut samples into four groups as inferred by STRUCTURE and Neighbor-Joining tree analysis (S3 Table). Gene pool 1 consisted of 41 individuals from Anatolia (Turkey, Sub-cluster 1–1); gene pool 2 comprised 131 individuals from the Balkans (admixed genotypes between Cluster 1 and Cluster 4); gene pool 3 comprised 279 individuals from northeastern Europe (Sub-cluster 4–2); and gene pool 4 included 650 individuals from western Europe (Sub-cluster 4–1).

In the first stage of the DIYABC analysis, we tested five competing broad-scale scenarios (Scenarios 1a-5a) based on historical and fossil pollen data (S4 Table). Scenarios 1a and 2a assumed a first introduction of walnut from Anatolia to the Balkans followed by a expansion from the Balkans through northeastern Europe to western Europe (Scenario 1a, Land Route) or from the Balkans through western Europe to northeastern Europe (Scenario 2a, Maritime Route). Then, we tested the presence of two refugia (Anatolia and the Balkans) with subsequent expansion of walnut from the Balkans through northeastern Europe to western Europe (Scenario 3a) or from the Balkans through western Europe to northeastern Europe (Scenario 4a). The final model of stage 1 (Scenario 5a) tested a complex scenario of independent human-induced dispersal of walnut from Anatolia and western Europe to the Balkans where admixture occurred, a subsequent expansion from western to northeastern Europe and a final population decline of western Europe. Alternative scenarios (e.g. western Europe as a unique centre of walnut origin) were considered unrealistic and therefore were excluded (S4 Table).

The second stage of the DIYABC analysis was at a finer scale, assuming two refugia (Anatolia and western Europe; Scenarios 1b-3b) or three refugia (Anatolia, the Balkans and western Europe; Scenarios 4b-6b), with admixture in the Balkans, a population size expansion in northeastern Europe, and decline in western Europe. We also evaluated the dispersal of walnut into northern Europe from western Europe (Scenario 1b, 4b), from the Balkans (Scenario 2b, 5b) or as a result of admixture between western Europe and the Balkan germplasm (Scenario 3b, 6b) (S4 Table).

The prior values used for all the demographic parameters selected for ten scenarios are listed in S3 Table. Following the procedure of Bai et al [46] for shorter-lived Asian butternuts, we assumed 80–110 years as a reasonable generation time for walnut (production of nuts 10–20 years after germination; mean life span of ~ 100–200 years; [47]). Based on these assumptions, we chose prior distributions for the timing of events (in generations) of stage 2 including Last Glacial Period (100–10,000), post LGM period (10–200), Bronze and Iron Age (20–60), Hellenistic-Roman period (10–25) and Middle Ages (1–15 or 1–10). We assumed a generalized stepwise mutation model for SSR loci, with a uniform mutation rate prior (μ) between 10^−4^ and 10^−3^. The observed and simulated genetic datasets were summarized using the mean number of alleles, mean genetic diversity, mean allele size variance for each population, Wright’s *F*_ST_, mean individual assignment likelihoods and mean index of classification (DAS) among populations. We generated a reference table containing 10^5^ simulated datasets for each scenario, and subsequently 1% of the simulated datasets closest to the observed genetic dataset were used to estimate posterior probabilities (with 95% confidence intervals) for each scenario using direct and logistic regression approaches [45]. The posterior distribution of historical demographic parameters was estimated using a logistic regression of 1% of the closest datasets simulated according to the most likely scenario. To validate the confidence in a scenario choice, we calculated (1) Type *I* Error (False negative rate) as the proportion of times that the selected scenario did not exhibit the highest posterior probability compared with the competing scenarios for 500 pseudo-observed datasets generated under the best-supported model, and (2) Type *II* Error (False positive rate) as the proportion of times that the selected scenario was incorrectly selected as the most likely scenario for 500 pseudo-observed datasets generated under each of the competing scenarios. Finally, we performed a model checking analysis to evaluate if a model-posterior combination fitted the observed data correctly. The observed summary statistics were compared with those computed for 1,000 datasets simulated from the posterior distribution of parameters obtained under the selected scenario [45].

## Results

### Genetic diversity of Eurasian walnut populations

**Fig 1:**
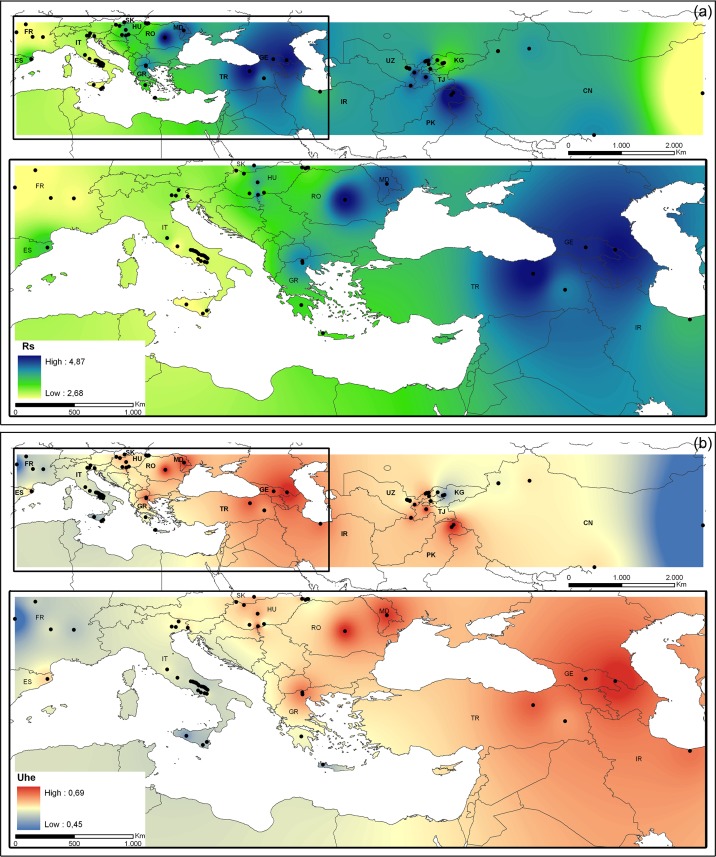
Genetic diversity of 91 walnut populations in Eurasia. Inverse Distance Weighted (IDW) interpolation of the allelic richness values (*Rs*) (a) and unbiased heterozygosity *UH*_E_ (b) calculated for 91 walnut populations (black dots) in Eurasia using 14 SSR markers (abbreviations CN = China, UZ = Uzbekistan, KG = Kyrgyzstan, TJ = Tajikistan, PK = Pakistan, IR = Iran, GE = Georgia, TR = Turkey, MD = Moldova, RO = Romania, HU = Hungary, SK = Slovakia, GR = Greece, IT = Italy, FR = France, ES = Spain).

All 14 SSR loci used in the present study were highly polymorphic in the sampled walnut populations. A total of 199 alleles were detected in the 2,008 walnut trees genotyped, with an average of 14.21 alleles per locus. None of the SSR loci showed evidence of null alleles (for a complete description of the SSR loci, see S5 Table). Genetic diversity parameters varied greatly among Eurasian walnut populations (S6 Table). Three geographic areas showed high values of allelic richness (*Rs*) and unbiased expected heterozygosity (*UH*_E_): 1) south–central Asia including sites in Tibet (32-DASH), Kashmir–western Himalaya (34-HUNZA and 33-GILGIT, Pakistan), northern Pamir ridges (35-SHOULI, Tajikistan), Gissar Mountain (18-BAKHMAL, Uzbekistan), western Tien Shan (19-KARANKUL and 17-BONSTANLYK, Uzbekistan); 2) four Trans-Caucasus sites (37-LAGO and 38-SKRA, Georgia; 39-ANATOLIA and 40-TRABZON Turkey) and 3) four Balkan populations (41-PAIKO_A, 42_PAIKO_B, Greece; 45-BRASOV, Romania; and 46-CHISINAU, Moldova) (Fig 1).

A significant decline in allelic richness was found with decreasing longitude (r = 0.522, *P* = 0.00015) and increasing latitude (r = 0.425, *P* = 0.00135). Of 199 alleles detected, 33 were unique to a single geographic site, with 23 unique to Asian and ten to European walnut populations (S6 Table). Overall, *F*_IS_ ranged from -0.066 (74-SANNIO) to 0.280 (28-GUILI-2), and was significantly greater than zero in 31 of 91 sampled sites, indicating a surplus of homozygotes in 18 (45%) and 13 (26%) of Asian and European walnut populations, respectively (S6 Table). Based on the TMP model, significant (*P* < 0.05) signals of recent reduction in effective population size were detected in a large proportion of European sites (73%), but only in four (10%) Asian populations (4-SHAIDAN, 5-KYZYL, 17-BOSTANLYK and 36-KARAJ). By contrast, using the M ratio test, we observed a genetic signature consistent with a bottleneck in all populations. The G–W values ranged from 0.28117 (36-KARAJ) to 0.37939 (72-VALCO), which is substantially lower than the critical threshold of 0.68 (S7 Table).

### Spatial genetic structure of walnut populations in Eurasia

**Fig 2:**
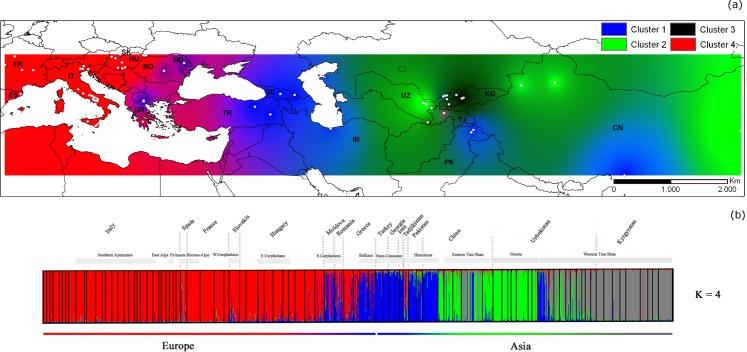
Spatial genetic structure of 91 walnut populations in Eurasia. Population structure inference for 91 walnut populations by Bayesian assignment using STRUCTURE for K = 4. Synthetic map of Inverse Distance Weighted (IDW) interpolations of the estimated mean population membership values (*Q*_*i*_) (a) and bar plot showing assignment probabilities of individuals to K clusters (b). Abbreviations: CN = China, UZ = Uzbekistan, KG = Kyrgyzstan, TJ = Tajikistan, PK = Pakistan, IR = Iran, GE = Georgia, TR = Turkey, MD = Moldova, RO = Romania, HU = Hungary, SK = Slovakia, GR = Greece, IT = Italy, FR = France, ES = Spain.

The genetic structure of the 91 sampled walnut populations was evaluated using STRUCTURE and Neighbor-Joining tree clustering analysis. STRUCTURE indicated four as the most appropriate number of population groups. The ad-hoc statistic ΔK of Evanno et al [40] combined with the analysis of Log-likelihood distribution of data L(K) as a function of K, recognized K = 4 as the best representation of the underlying hierarchical structure of the 91 walnut populations in Eurasia (S2 Fig). Cluster 1, centered in western and south-central Asia, included all walnuts sampled from four Trans-Caucasus sites (37-LAGO and 38-SKRA, Georgia; 39-ANATOLIA and 40-TRABZON Turkey), Alborz ridges, Iran (36-KARAJ), Kashmir-western Himalayas, Pakistan (33-GILGIT and 34-HUNZA) and Tibetan-eastern Himalaya, China (32-DASH) (*Q*_*1*_ ≥ 0.8227) (S8 Table). In addition, 21 walnut trees (58.3%) collected in 19-KARANKUL (eastern Uzbekistan) and ten of 67 walnut trees (14.9%) from 28-GONGLIU-2 (Xinjiang province, China) were unambiguously assigned to cluster 1 (Q1 ≥ 0.800) (Fig 2).

Cluster 2 assembled seven populations from the Nurata ridge located in east-central Uzbekistan and five populations of northern and eastern China, i.e., four sites in the eastern Tien Shan mountains, Xinjiang province, and one site from Shandong province (*Q*_*2*_ ≥ 0.7639). Cluster 3 comprised all nine Kyrgyz and four Uzbek populations (10-Kamchik, 11-Yakkatut, 12-Sidjak and 13-Charvak) sampled in the walnut forests of the western Tien Shan mountains (*Q*_*3*_ ≥ 0.7856). The remaining three walnut populations in the western Tien Shan mountains (14-Nanai, 16-Bogustan, 17-Bostanlyk) and two populations from the Gissar mountains (15-Djarkurgan) and the Zaamin mountains (18-Bakhmal) in eastern Uzbekistan were mainly admixtures between cluster 2 (0.2013 ≤ *Q*_*2*_ ≥ 0.3740) and cluster 3 (0.4987 ≤ *Q*_*3*_ ≥ 0.7118). Cluster 4 included all European walnut populations sampled in Spain, Italy, France, Slovakia, Crete (Greece) and Hungary (*Q*_*4*_ ≥ 0.7648), except for five easternmost Balkan populations located in Greece (Macedonia, 41-PAIKO_A, 42_PAIKO_B; Peloponnese, 43-ARCADIA), Romania (45-BRASOV), and Moldova (46-CHISINAU) and the remaining population from Tajikistan (35-SHOULI), which showed admixed profiles between cluster 1 (0.3101 ≤ Q_1_ ≥ 0.7420) and cluster 4 (0.2230 ≤ *Q*_*4*_ ≥ 0.6663) (Fig 2, S8 Table).

**Fig 3:**
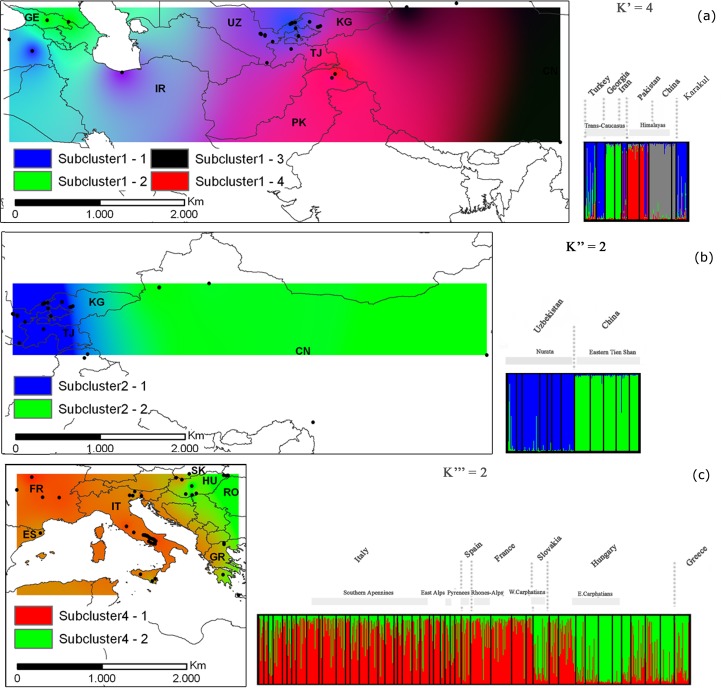
Spatial genetic sub-structure of the inferred clusters 1, 2 and 4 of walnut populations. Synthetic maps of Inverse Distance Weighted (IDW) interpolations of the estimated mean population membership values (*Q*_*i*_) and bar plot for (a) K’, the most probable number of sub-clusters, based on microsatellite analysis of 217 walnut samples of cluster 1, (b) for K”, the most probable number of sub-clusters, based on microsatellite analysis of 280 walnut samples of cluster 2 and (c) for K”‘ the most probable number of sub-clusters, based on microsatellite analysis of 929 walnut samples of cluster 4.

Subsequent STRUCTURE analysis within each of the previously inferred clusters revealed genetic substructure, except for cluster 3 (S2 Fig and S8 Table). The inferred cluster 1 was composed of four sub-clusters (K’ = 4). These four sub-clusters split walnut trees of 39-ANATOLIA, 40-TRABZON (Turkey), 36-KARAJ (Iran) and 19-KARANKUL (21 samples, eastern Uzbekistan) (sub-cluster 1–1), from 38-LAGO, 39-SKRA (Georgia) (sub-cluster 1–2), 28-GONGLIU-2 (ten samples, Xinjiang province, China), 32-DASH (Tibet, China) (sub-cluster 1–3) and 33-GILGIT, 34-HUNZA (Kashmir, Pakistan) (sub-cluster 1–4) (Fig 3A).

**Fig 4:**
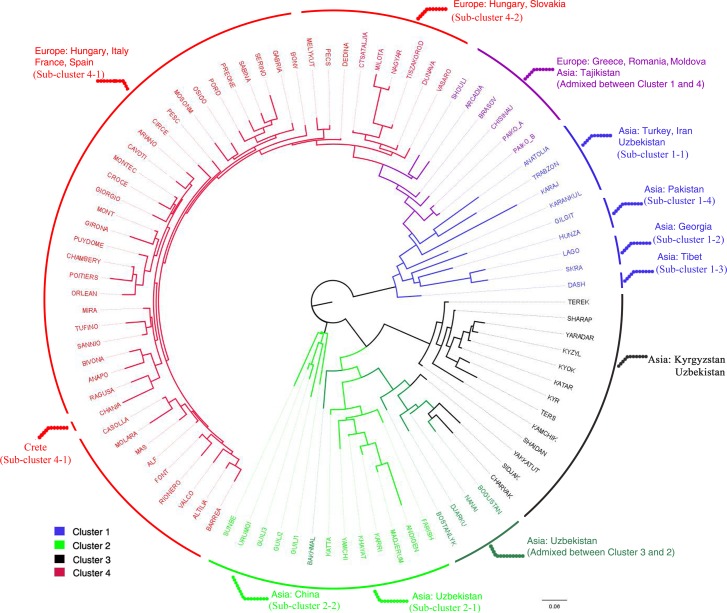
Neighbor Joining cluster analysis of 91 walnut populations based on unbiased Nei’s genetic distance. Neighbor Joining-based circular tree for 91 walnut populations from the species’ Eurasian range based on unbiased Nei’s genetic distance [43]. The assignment of walnut populations to four clusters and eight sub-clusters inferred by STRUCTURE is shown.

Two sub-clusters of the inferred cluster 2 (K” = 2) corresponded to two distinct groups of walnut populations sampled in east-central Uzbekistan (sub-cluster 2–1) and China (sub-cluster 2–2, Fig 3B). Finally, two sub-clusters of the inferred cluster 4 (K”‘ = 2) extended from Spain to northwestern Hungary (Györ-Moson-Sopron Province, 55-BONY, 56-MOSONM) (sub-cluster 4–1) and from eastern Hungary and Slovakia (Carpathians Mountains) to western Crete (44-CHANIA) (sub-cluster 4–2) (Fig 3C). A Neighbor-Joining tree based on Nei’s [39] genetic distance confirmed the previous results and showed that five easternmost Balkan sites in Greece (Macedonia and Peloponnese), Romania and Moldova might represent the contact zones between European (Cluster 4) and south west Asian (Cluster 1) germplasm passing through the Anatolian plateau (sub-cluster 1–1) (Fig 4).

### Inferring demographic history of walnut in Europe using ABC analysis

The ABC method enabled us to test ten demographic scenarios (S4 Table) and to delineate postglacial dispersal routes of walnut across Europe. In the first stage of the DIYABC analysis, both direct (*P*_*1*_) and logistic regression (*P*_*2*_) approaches identified scenario 5a as the most likely model (Table 1, S3 Fig, *P*_*1*_ = 0.3620, 95% CIs: 0.0000–0.7832, *P*_*2*_ = 0.9474, 95% CIs: 0.8396–1.0000).

**Table 1:** Most likely demographic scenario for European walnut by the DIYABC approach. Posterior probability (*P*) and 95% confidence interval of *P* (in brackets) computed using a direct (*P*_*1*_) and logistic regression (*P*_*2*_) approach are provided for each scenario tested by the DIYABC approach. The most likely scenario for each stage is reported in grey. Confidence in scenarios was evaluated using type *I* error (False negative) and type *II* error (False positive) rates for logistic regression.

Stage 1–2	*P*_*1*_	*P*_*2*_	Type *I* Error	Type *II* Error ^a^
Scenario 1a	0.3120 [0.0000–0.7181]	0.0002 [0.0000–0.0049]	0.2360	0.0000
Scenario 2a	0.1320 [0.0000–0.4287]	0.0002 [0.0000–0.0049]	0.3200	0.0000
Scenario 3a	0.1300 [0.0000–0.4248]	0.0449 [0.0000–0.1460]	0.3880	0.0002
Scenario 4a	0.0640 [0.0000–0.2787]	0.0072 [0.0000–0.0250]	0.4040	0.0080
Scenario 5a	0.3620 [0.0000–0.7832]	0.9474 [0.8396–1.0000]	0.0400	-
Scenario 1b	0.1120 [0.0000–0.3884]	0.1638 [0.0000–0.3696]	0.1560	0.0000
Scenario 2b	0.0200 [0.0000–0.1427]	0.0000 [0.0000–0.0426]	0.1580	0.0000
Scenario 3b	0.0520 [0.0000–0.2466]	0.0519 [0.0000–0.1156]	0.4140	0.0140
Scenario 4b	0.2600 [0.0000–0.6445]	0.0005 [0.0000–0.0428]	0.0640	0.0260
Scenario 5b	0.1720 [0.0000–0.5028]	0.0000 [0.0000–0.0426]	0.0720	0.0300
Scenario 6b	0.3840 [0.0000–0.8103]	0.7838 [0.5558–1.0000]	0.1600	-

^a^ Type *II* Error is the proportion of pseudo-observed datasets simulated using each competing scenario (1a-4a, 1b-5b) that support focal scenario (scenario 5a for stage 1and scenario 6b for stage 2).

Four excluded scenarios assumed that populations from Anatolia (Scenario 1a-2a) or the Balkans (Scenario 3a-4a) served as a unique source for the human-mediated walnut colonization of the rest of Europe. Scenario 5a proposed two independent dispersal events of walnut from Anatolia (Pool 1) and western Europe (Pool 4) to the Balkans with subsequent genetic admixture (Pool 2). In the second stage of the DIYABC analysis, we tested six fine-scale scenarios always including two (Anatolia and western Europe, scenario 1b-3b) or three glacial refugia (Anatolia, the Balkans and western Europe, scenario 4b-6b) with admixture events in the Balkans (S4 Table). A comparison of posterior probabilities of the six scenarios unambiguously indicated that scenario 6b was the most likely model (Table 1, S3 Fig, *P*_*1*_ = 0.3840, 95% CIs: 0.0000–0.8103, *P*_*2*_ = 0.7838, 95% CIs: 0.558–1.0000). The model checking procedure showed that the observed summary statistics for our data were not significantly different (*P* < 0.01) from the simulated ones calculated from the posterior predictive distribution of parameters for scenario 6b. These findings provided further support for a high-goodness-of-fit observed dataset to selected scenario 6b (S9 Table). The computation of marked low false negative (Type *I* Error = 16%) and false positive (Type *II* Error = 0–3%) rates clearly indicated that our method selected the true scenario with high confidence, showing high power to distinguish among alternative demographic scenarios (Table 1).

Scenario 6b inferred an initial split between an Anatolian (Pool 1) and a hypothetical ancestral European pool (NG1) at 579 generations (*t*_*4*_ median value, 95% CIs: 217–2,010), and between western Europe (Pool 4) and pool NG1 at 118 generations (*t*_*3*_ median value, 95% CIs: 42.3–192) (Table 2).

**Table 2:** Parameters estimates of the most likely scenarios of walnut expansion. Parameters estimates of the most likely scenarios (scenarios 6b) inferred by the Approximate Bayesian DIYABC Computation [45] in the stage 2. Estimation of parameters is based on 1% of the closest data sets and subsequent logit transformation.

Parameter	Description	Mean	Median	Q _0.05_	Q _0.95_
N1 ^a^	Effective population size of Pool 1 (Anatolia, Asia)	5.62E+003	5.46E+003	2.09E+003	9.38E+003
N2	Effective population size of Pool 2 (The Balkans, Europe)	3.22E+003	2.55E+003	7.00E+002	8.27E+003
N3	Effective population size of Pool 3 (north-eastern Europe)	6.30E+003	6.46E+003	2.17E+003	9.69E+003
N4	Effective population size of Pool 4 (western Europe)	6.14E+003	6.32E+003	2.55E+003	9.12E+003
Nm	Effective population size of Pool 4 (western Europe) before human-induced population decline at tm	7.73E+003	8.04E+003	4.61E+003	9.86E+003
Nd	Effective population size of Pool 3 (north-eastern Europe) before human-induced population expansion at td	1.44E+003	9.72E+002	2.14E+002	4.32E+003
NG1	Effective population size of an ancestral population NG1 in the Balkans (Europe)	2.14E+003	1.62E+003	4.40E+002	6.26E+003
NA	Effective population size before divergence between Pool 1 (Anatolia, Asia) and ancestral population NG1.	5.94E+003	6.21E+003	1.61E+003	9.44E+003
t_m_	Time of human-mediated decline of Pool 4 (western Europe).	2.86E+000	1.96E+000	1.00E+000	1.00E+001
t_d_	Time of human-mediated expansion of Pool 3 (north-eastern Europe)	6.03E+000	5.23E+000	1.00E+000	1.38E+001
t_1_	Time of admixture between Pool 4 (western Europe) and Pool 2 (The Balkans) giving rise to Pool 3 (north-eastern Europe)	1.92E+001	1.97E+001	1.19E+001	2.50E+001
t_2_	Time of admixture between Pool 1 (Anatolia, Asia) and NG1 (ancestral population in the Balkans) giving rise to Pool 2 (The Balkans)	4.85E+001	5.13E+001	2.86E+001	5.97E+001
t_3_	Divergence time between Pool 4 (western Europe) and ancestral population NG1 in the Balkans.	1.18E+002	1.18E+002	4.23E+001	1.92E+002
t_4_	Divergence time between Pool 1 (Anatolia, Asia) and ancestral population NG1 in the Balkans.	8.00E+002	5.79E+002	2.17E+002	2.01E+003
ra	Admixture rate between Pool 1 (Anatolia) and ancestral population NG1 in the Balkans.	2.62E-001	2.58E-001	1.40E-001	3.90E-001
rb	Admixture rate between Pool 4 (western Europe) and ancestral population NG1 in the Balkans.	3.95E-001	3.81E-001	1.53E-001	6.81E-001

^a^ N# refers to effective population size of each corresponding gene pool, and t# to time-scale in terms of the number of generations.

**Fig 5:**
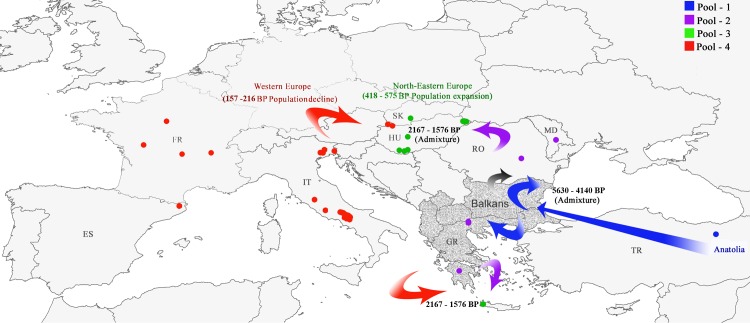
Human-mediated dispersal routes of walnut during the Late Holocene as inferred by DIYABC analysis. Human-mediated dispersal routes of walnut during the Late Holocene as inferred by approximate Bayesian computation [45]. Arrows represent the relationships between population pools used in DIYABC analysis (Pool 1, Pool 2, Pool 3, Pool 4) as inferred from stage 2, scenario 6b. Hypothetical glacial refugia located in the Balkans (ancestral pool NG1) are reported in dark grey. Abbreviations: TR = Turkey, MO = Moldova, RO = Romania, HU = Hungary, SK = Slovakia, GR = Greece, IT = Italy, FR = France, ES = Spain.

A secondary contact between NG1 and Anatolian groups occurred at 51.3 generations (*t*_*2*_ median value, 95% CIs: 28.6–59.7) resulting in the walnut populations currently present in the Balkans (Pool 2). Walnut dispersal from the Balkans and western Europe at 19.7 generations (*t*_*1*_ median value, 95% CIs: 11.9–25.9) promoted a second admixture across northeastern Europe (Pool 3) (Table 2). Assuming *c*. 80–110 years per generation for *J*. *regia*, divergence and admixture times scaled to 46,320 to 63,690 BP (*t*_*4*_), 9,440 to 12,980 BP (*t*_*3*_), 4,140 to 5,630 BP (*t*_*2*_) and 1,576 to 2,167 BP (*t*_*1*_*)* (S3 Fig, Fig 5).

The inferred median effective population sizes for scenario 6b were 5,460 (N1), 2,550 (N2), 6,460 (N3), 6,320 (N4), 1,620 (NG1) and 6,210 (NA) (Table 2). In addition, the DIYABC model supported a subsequent expansion of northeastern European admixed pool 3 (from Nd = 972 to N3 = 6,460, 7-fold increase) at 5.23 generations (*t*_*d*_ median value, 95% CIs: 1–13.8), corresponding to ~418–575 BP, and a recent population decline of western European pool 4 (from Nm = 8,040 to N4 = 6,320) at 1.96 generations (*t*_*m*_ median value, 95% CIs: 1–10). During the last two centuries (~ 157–216 BP), western European germplasm was reduced to 21.4% of its former size (Fig 5).

## Discussion

### Genetic diversity and postglacial recolonization of walnut in Europe: A single refugium vs multiple glacial refugia

Our analyses detected a high degree of walnut genetic diversity in the Balkans (Greece, Romania and Moldova), similar to that recorded in other native regions such as the Caucasus (Georgia, Turkey), and the mountainous regions of central Asia including the Himalayas (Tibet and Kashmir, Pakistan), northern Pamir (Tajikistan), the Zaamin ridges, the northern Gissar ridges, and part of the Tien Shan mountains (eastern Uzbekistan and Xinjiang Province, China). In addition, we demonstrated a clear longitudinal trend of walnut genetic diversity in Eurasia with a marked loss of allelic richness and heterozygosity running from eastern to western Europe, as well as molecular signals of recent reduction in effective population size.

Based on fossil pollen evidence (S1 Video), peaks of genetic diversity occurred in some macro-regions of the eastern Mediterranean basin where *J*. *regia* might have survived after the LGM. Walnut was detected during the Early Holocene in Turkey (Yeniçaga Gölü, 11,485 BP [9]), F.Y.R. of Macedonia (Lake Prespa, ~9,000 BP [17], Greece (Lake Kastoria, 8,203 BP [48]), and in Bulgaria near the Macedonian border (western Rhodopes Mountains, Beliya Kanton, 11,708 BP [49]) and the Black Sea (Varna, 11,332 BP [50]). As reported for many plant species of the Northern Hemisphere, glacial refugia are considered reservoirs of high levels of genetic diversity [51]. The traditionally recognized paradigm for temperate tree species in Europe included post-glacial recolonizations of de-glaciated areas from main refugia in the southern Mediterranean (Iberia, Italy, the Balkans and Turkey) [51–53]. Therefore, assuming this basic expansion/contraction model, and the spatial concordance between regional hotspots of walnut genetic diversity and two phylogeographically well-defined plant refugia (the Balkans and Turkey), we consider the extinction of *J*. *regia* in Europe to be unlikely [9–10], and postulate its persistence at least in the eastern Mediterranean during the cold and dry periods of the Pleistocene. As already suggested for *Pinus halepensis* Mill. [54], *Quercus suber* L. [55–56] and *Quercus cerris* [57], the inferred longitudinal imprint of walnut genetic diversity could be the result of genetic drift due to long distance dispersal and founder effects that occurred prior to or during the Holocene recolonization of western Europe from eastern Mediterranean refugia.

Our data, however, imply a more complex explanation for the observed longitudinal cline in European walnut genetic diversity. In the last decade, meta-analysis of tree and animal species in the Mediterranean highlighted the importance of two, non-mutually exclusive major processes [58]: the contraction of formerly continuous ranges in relation to Quaternary climate oscillations, and the subsequent westward and eastward waves of colonization during the Holocene fostered by an east (warm/wet) to west (cold/dry) climatic cline at the Last Glacial Maximum. We argue that these events can explain the longitudinal gradient of walnut genetic diversity across Europe [59–61]. We agree with Feliner [58] that spatial phylogenetic coincidences among populations in different plant/animal groups does not always imply common evolutionary processes (pseudo-congruence phenomenon). In particular, the distribution of Mediterranean tree species, economically important for their nut and/or wood production, is known to have been substantially altered by humans over several thousand years [13], [62]. For instance, a severe and prolonged demographic decline of *Pinus pinea* L. across the Mediterranean, followed by human-mediated dispersal starting around 3000 BP, resulted in a nearly complete loss of genetic diversity in the species [63]. Perhaps, as attested by different human density/forest clearance/land use rates computed after 1,650 BP [64], the relatively low genetic diversity of walnut populations in western Europe might reflect a stronger impact of human activities on population size in that region compared to the Balkans. In both cases, as indicated by the earliest traces of *Juglans*-type fossil pollen (S1 Video), and by the recent computation of fossil pollen density of *Juglans* from the Late Glacial across Europe based on an extended EPD data [65], shelter zones for walnut in western Europe such as the Italian peninsula (e.g. Tourbière de Pilaz, North-Western Alps, 9,756 BP [66]), Spain (e.g. Sanabria Marsh, Northwestern Iberia, 8,721 BP [67–69]) or France (e.g. Lignin Lake, Southern Alps, 7,545 BP [70]; Armorican massif, Western Atlantic region, ~8000 BP [71]) seem plausible. They might have contributed to the bidirectional colonization of Europe by walnut from western and eastern Mediterranean reservoirs with natural- or human-mediated bottlenecks.

Our genetic structure analysis of Eurasian populations corroborated the theory of separate regions of walnut evolution in Eurasia, dividing the walnut samples into four main clusters: clusters 1–3 centred in Asia with cluster 4 located in Europe (see Fig 2). Our genetic data indicated a subsequent bidirectional spread of walnut across Europe from western and eastern Mediterranean refugia. Walnut trees from the Balkans were in fact genetically distinct from the other European lineages. They exhibited admixture between clusters 1 and 4 and represented a putative contact zone between European and Asian continents. In addition, cluster analyses showed a subtle genetic subdivision of cluster 4 into two sub-clusters, separating all western European and north-western Hungarian walnut trees (sub-cluster 4–1) from eastern Hungary, Slovakia and western Crete (sub-cluster 4–2).

The approximate Bayesian analysis of ten alternative hypotheses confirmed the existence of three glacial refugia for walnut in the Mediterranean. It also revealed complex population dynamics during the late Holocene, presumably the consequence of anthropogenic translocation and use of walnut germplasm. Although time estimates should be taken with caution, the inferred demographic scenario indicated that a continuous ancestral European pool (NG1) diverged from the Anatolian-Turkish pool during the last Early-Middle Pleniglacial Period, from 63,690 BP (with a generation time of 110 years) to 46,320 BP (generation time of 80 years), predating the LGM. Our data confirmed the splitting of NG1 into western European and Balkan germplasm from the Younger Dryas (12,900 BP) to the Early Holocene (9,440 BP) (Fig 5).

Last Glacial reconstructions have revealed a long-term contraction of temperate tree species in southern Europe, superimposed on millennia-scale climatic fluctuations [72–74]. This period has been classified as one of the most severe of the whole Pleistocene, in terms of ice volume, and extent, and in the reduction of tree populations. The Last Glacial and the Early Holocene coincided with an abrupt decrease in fossil pollen deposits of walnut in Turkey (20,000–17,000 BP and 15,000–10,000 BP, Lake Van [75]), Bulgaria (11,103–10,498 BP, Beliya Kanton [49]), Greece (11,500–8,300 BP, Myrtoon basin [18]) and Italy (11,498–9,445 BP, Lago di Martignago [76]). Congruent with the evolution of vegetated landscapes, our most-likely scenario indicated that the cold steppe-type environment of the Early-Middle Pleniglacial (63,000–29,000 BP) and Younger Dryas period (12,900–11,700 BP) [77–78] may have been the deciding factor for the progressive fragmentation of walnut populations in the Mediterranean. Based on the results of this study, despite an increasing amount of evidence for the survival of tree taxa in central and even northern Europe over the Quaternary [52, 74], we conclude that walnut persisted in environmentally favourable pockets in the Anatolian plateau, the Balkans and southern-western Europe over the LGM. However, the detection of few pollen grains of walnut after the LGM in Northern Czech Republic (10,546 BP, Dolskym), Western Austria (11,645 BP, Seefelder See; 11,267 BP, Fuchsschwanzmoos), Southern Germany (10,005 BP, Feuenried), Southern Switzerland (10,399 BP, Lac du Mont d'Orge Sion) and Central France (10,581 BP, Tourbières des Granges des Chavants; 9097 BP, Marais du Grang Chaumet; 9466 BP Tourbière de Roussy; 9334 BP, Tourbière de Parcay-sur-Vienne) (S1 Table and S1 Video) might indicate an early spread of walnut at relatively high latitudes (>46° N) at the onset of the Holocene in Europe. Although the presence of re-deposited pollen of Juglandaceae from the Tertiary deposits can’t be ruled out (for more details see S1 Table), the pollen abundance of *Juglans* starting from 15,000 BP [65] suggested that the putative presence of cryptic refugia of walnut in the Alps and Central Europe is worth exploring. In addition, the absence of a sharp genetic differentiation between western and eastern Mediterranean populations, means that we can’t rule out long-distance gene flow among walnut lineages from different refugia. The DIYABC analysis confirmed that, in all probability, two subsequent human-mediated events occurred: admixture between the Anatolian-Turkish pool and natural stands in the Balkans (4,140–5,643 BP) and between western Europe and Balkan germplasm in eastern Europe (1,576–2,167 BP). A final population size expansion in northeastern Europe and population size decline in western Europe were detected in the last five centuries. As proposed for other cultivated tree species in the Mediterranean, such as *Castanea sativa* Mill. [79–80] and *Olea europaea* [81–82], these findings strongly imply that the modern genetic structure of *J*. *regia* in Europe resulted from the combined effects of expansion from multiple European refugia after the LGM and human dispersal of walnut germplasm.

### Late Holocene human dispersal pathways of walnut in Europe

Our data clearly indicate a contact zone in southeastern Europe where walnut populations accumulated high genetic diversity as a result of admixture of genetic lineages from the Balkans and western Asia. Although DIYABC analysis only tests instantaneous admixture events or population size changes [83], we can realistically assume a model of gradual admixture for these populations. Walnut germplasm might have been imported from Anatolia to southeastern Europe, and then hybridized with autochthonous Balkan trees over several generations starting from the Early Bronze Age (EBA) (6,000–3,950 BP). Consequently, the general view that common walnut was introduced into the Balkans from Iran and eastern Turkey by Greek commerce during the Achaemenid phase (2,500–2,330 BP) must be partially revised [1]. Although the establishment of the Persian Empire corresponded with the maximum expansion of walnut cultivation across the Irano-Turanian regions [84], fossil pollen evidence (S1 Video) and our DIYABC inference indicated that Turkey might have acted as a bridge for inter-regional exchange of walnut germplasm between the Near East and the Aegean region from the 6^th^ millennium BP onward. The occurrence of non-native oilseed species, such as *Lallemantia* [85] and *Carthamus* spp [86], and fruit species such as *Punica granatum* L. [87] and *Cucumis melo* L. [88], in northern Greece and the eastern part of the Thracian plain as early as 5,000–4,600 BP, reflected strong, far-reaching cultural and economic contacts during the EBA I and II between the eastern Mediterranean and adjacent regions (Anatolian cultures, phases of Troia I and IIa). The sea- and inland-based trading networks associated with bronze technology of the Aegean-Anatolian circuit prospered and promoted the exchanges of prestige goods, ideas and new technologies (e.g. wheel-made pottery, metallurgy and agriculture) [89]. However, although barter was the usual form of trade, and crops were often documented as a means of payment [90], the motivation of human-mediated long distance dispersal of walnut across its native region of southeastern Europe needs further investigation.

As outlined for the distinct Beysehir Occupation Phase (~ 3,200–1,350 BP) [9], the massive climatic fluctuations that occurred from ~ 5,300 to 2,800 BP led many Bronze Age populations to adopt new agricultural strategies in southeastern Europe [23]. Repeated agro-arboricultural reorientations may have been required within the Aegean basin, and thus active transport and plantation of walnuts from the humid highland regions of eastern Anatolian and Iran where cultivation was less affected by climatic changes [23]. Walnut trees we sampled in the Balkans were nearly indistinguishable morphologically from remaining European trees, with the exception that some Balkan trees only bore fruit terminally (European type), while others had intermediate and lateral (Asian type) bearing habits (M.E Malvolti, S. Mapelli pers. obs.). We postulate that high-yielding lateral bearing forms of walnut were repeatedly introduced from western Asia to Greece and the Thracian plain during the Bronze Age, and later during the Hellenistic period (Alexander the Great ~2,273BP), promoting gene flow between lateral and terminal bearing tree types.

Another important feature that may have contributed to dispersal, planting and maintenance of walnut across Eurasia is the high quality of its wood. Unfortunately, the history of tree plantation and forest management in Asia and the eastern Mediterranean is poorly documented [6]. Conversely, it is very well known that walnut plantations for fruit [13] and timber production [91] were integral parts of Roman agro-forest management across Europe. Our genetic analysis, together with the time sequence of pollen presence (S1 Video), confirmed that the main expansion of *J*. *regia* throughout Europe occurred after 2,500 BP, and was likely the result of walnut’s widespread use during Greek and Roman periods [11, 13, 92–93]. After the consolidation of the Roman Empire between the 1^st^ and 2^nd^ centuries CE (1,850–1,750 BP), the cultivation of walnut was incorporated into the local agricultural tradition of north-central Europe, losing its character of exclusivity as an imported luxury food [94]. In particular, with military campaigns (e.g. the conquests of Emperor Augustus (2,030–1,986 BP)), and the subsequent reorganization in Roman provinces of north-central European regions, the western (Italy, Spain and France) and eastern (Macedonia and the Balkans) halves of Europe were united by overland routes [95]. Therefore, as our genetic model indicates, the Pannonian basin (modern Hungary and Slovakia) became a crossroads of European cultures, promoting contacts and hybridization between walnut genetic lineages from western Europe and the Balkans. Subsequently, after the decline of the western Roman Empire (1,600 BP) and tumultuous Migration Period (1,700–1,100 BP), and as a consequence of urban development and increasing human population density, the Late Middle Ages (900–500 BP) marked a favorable period for fruit cultivation and agriculture in Hungary and its adjacent regions, leading to a local walnut population expansion [96]. Conversely, from ~ 200 BP, we detected signs of decline in the effective population size of walnuts collected in Italy, Spain and France. During the early 19^th^ century, forests of many western European countries were substantially cleared, followed by the introduction of intensive agriculture based on new crops such as the potato and other changes precipitated by the Industrial Revolution [64]. This strong human pressure resulted in a progressive depletion of walnut genetic resources in western Europe [47].

In conclusion, our results demonstrate that the present spatial genetic structure of walnut in Europe resulted from the combined effects of expansion and contraction from multiple refugia after the LGM and the human dispersal and management of walnut over the last 5,000 yr. The evolutionary history of walnut is tightly linked with human history during the Late Holocene, making it a permanent feature of the economic, cultural and rural heritage of Europe.

## Supporting information

S1 Fig Geographic location of 91 common walnut populations collected across Eurasia. Kyrgyzstan (1–9), Uzbekistan (10–26), China (27–32), Pakistan (33–34), Tajikistan (35), Iran (36), Georgia (37–38), Turkey (39–40), Greece (41–44), Romania (45), Moldova (46), Hungary (47–56), Slovakia (57),France (58–61), Spain (62) and Italy (63–91).
(TIF)

S2 Fig Bayesian inference of the most probable number of clusters and sub-clusters for 91 walnut populations. Bayesian Inference of (a) K, the most probable number of clusters, based on microsatellite analysis of all 2,008 common walnut samples, (b) K’, the most probable number of sub-clusters, based on microsatellite analysis of 217 common walnut samples of cluster 1, (c) K”, the most probable number of sub-clusters, based on microsatellite analysis of 280 common walnut samples of cluster 2, and (d) K”‘ the most probable number of sub-clusters, based on microsatellite analysis of 929 common walnut samples of cluster 4 using STRUCTURE software [39]. Log-likelihood value of data L(K) as a function of K averaged over six replicates and second order of change of the log-likelihood of the data (ΔK) as a function of K, calculated over six replicates [40] was reported for each analysis.
(TIF)

S3 Fig All scenarios tested in stages 1–2 of DIYABC analysis. All scenarios tested in stage 1 (a) and stage 2 (b) of DIYABC analysis. In these scenarios, N# refers to effective population size of each corresponding gene pool, and t# refers to time-scale in terms of the number of generations (more details for population parameters and models in S3 and S4 Tables). Posterior probability (*P*) of each scenario and its 95% confidence interval of *P* (in brackets) computed using a direct (*P1*) and logistic regression (*P2*) approach are provided under each scenario. The most likelihood scenario for each stage is marked with a red rectangle.
(TIF)

S1 Table List of 450 fossil pollen sites from Eurasia considered in this study. The geographic coordinates in decimal degrees (Latitude, Longitude), early presence (1700–11.923 Ka BP), first detection of discontinuous and continuous occurrence of *Juglans*-type fossil pollen (radiocarbon-dating) during the Holocene and the related citation (a) and the presence/absence of *Juglans*-type fossil pollen and its classification (discontinuous, continuous, in expansion, in contraction and not recorded) in each selected time interval (b) were recorded for each site.
(PDF)

S2 Table Description of 91 common walnut populations sampled in Eurasia. Number of samples (N), geographic coordinates (Lat, Long) and elevation above sea level (Elev) for 91 common walnut populations collected in Eurasia.
(DOCX)

S3 Table Parameters used for DIYABC analysis. Population pools and the prior distributions of the parameters used for the two stages of DIYABC analysis.
(DOCX)

S4 Table Scenarios considered in the two stages of DIYABC analysis. Description of the scenarios of common walnut expansion across Europe considered in the two stages of DIYABC analysis.
(DOCX)

S5 Table Genetic characterization of 14 microsatellite loci for 91 common walnut populations. Total number of alleles (A), the effective number of alleles (Ae), observed (Ho) and expected heterozygosity (H_E_), polymorphic information content (PIC), and the unbiased estimate of Wright’s fixation indices, within-population inbreeding coefficient *f* (F_IS_), total-population inbreeding coefficient F (F_IT_) and among-population genetic differentiation coefficient θ (F_ST_), among-population genetic differentiation coefficient calculated on allele frequencies adjusted for null allele estimates F_ST (null)_ and the estimator of actual differentiation D_est_, [29] are shown are for each locus.
(DOCX)

S6 Table Genetic diversity of 91 common walnut populations. Mean number of alleles per locus (A), effective number of alleles (Ne), allelic richness (Rs) and private allelic richness (PAR) standardized to eight individuals from the original number of trees per population, observed (H_O_), expected (H_E_), and unbiased expected heterozygosity (UH_E_) and inbreeding coefficient (F_IS_) are shown.
(DOCX)

S7 Table Bottleneck analysis of 91 common walnut populations sampled across Eurasia using 14 SSR markers. Wilcoxon’s signed-rank’ test [35], shifted allele distribution analysis [37] and the M-ratio test [38] for each walnut population are reported.
(DOCX)

S8 Table Mean percentage of membership (*Qi*) of each common walnut population inferred by STRUCTURE. Percentage of membership (admixture proportion-*Q*) of each predefined common walnut population in each of the four (K = 4) clusters, two (K’ = 4) sub-clusters for cluster 1, two (K” = 2) sub-clusters for cluster 2 and two (K”‘ = 2) sub-clusters for cluster 4 inferred by Bayesian approach using STRUCTURE software (Pritchard *et al*., 2000). *Q*-values greater than 0.75 are reported in bold.
(DOCX)

S9 Table Model checking of the most likely scenario inferred in the first and second stage. Model checking of the most likely scenario inferred in the first stage (scenario 5a) and in the second stage (scenario 6b) of DIYABC analysis. Deviation of summary statistics computed for the observed dataset from the posterior predictive distribution of the most likely scenario is given as a proportion of data sets simulated from the posterior having a value lower than the observed dataset (*S*_*simul*._ *< S*_*obs*._).
(DOCX)

S1 Video Distribution of *Juglans*-types fossil pollen in Europe before the LGM and during the Holocene. Distribution of the radio-carbon dated *Juglans*-types fossil pollen in Europe and western Asia before the LGM and during the Holocene.
(MOV)
