# Supplementary figures and images for: Rethinking the history of common walnut (Juglans regia L.) in Europe: Its origins and human interactions
Source: PLoS One. 2017 Mar 3;12(3):e0172541. doi: 10.1371/journal.pone.0172541 (PMC5336217; doi:10.1371/journal.pone.0172541)

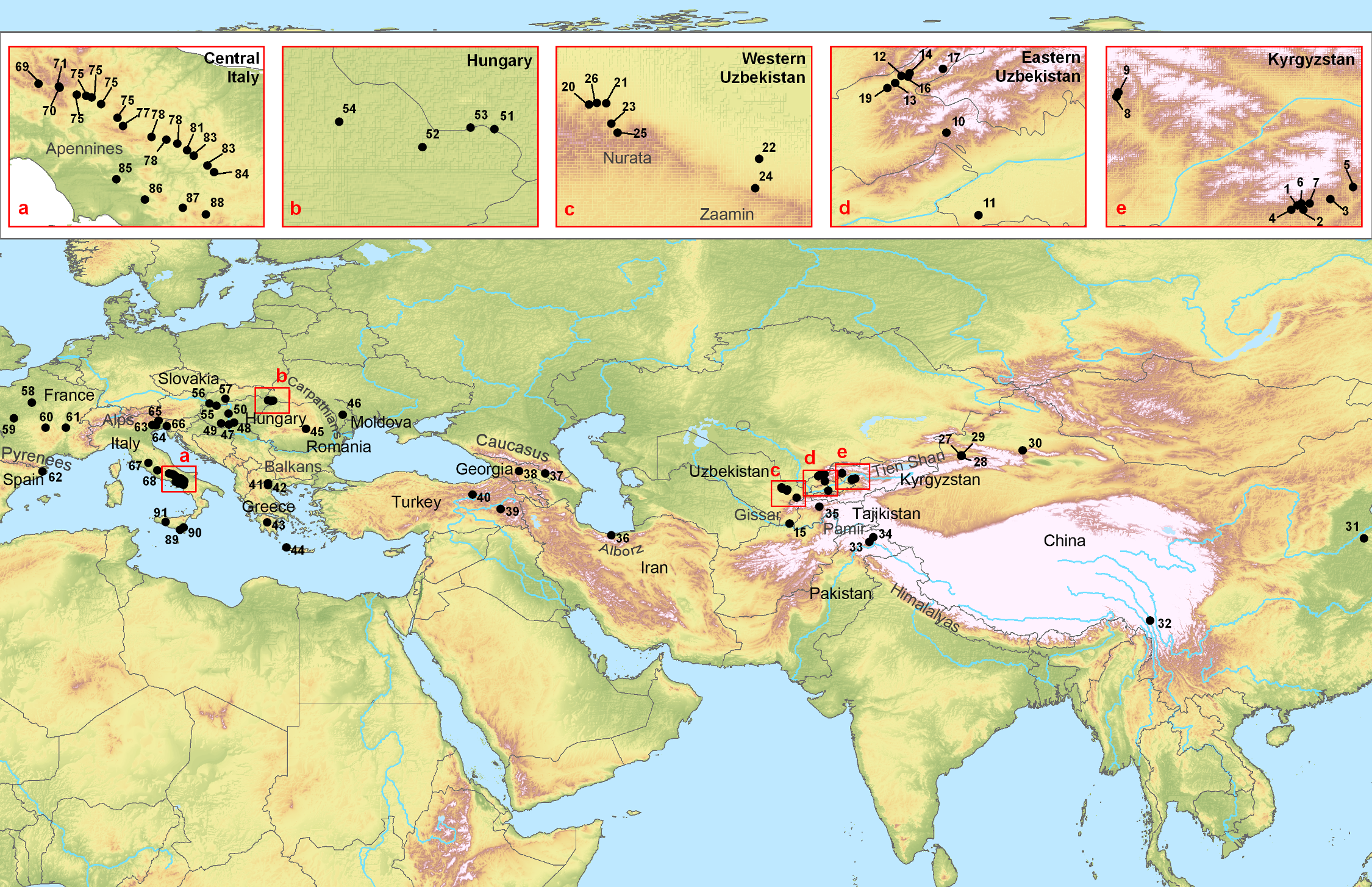

Supplement: S1 Fig — Kyrgyzstan (1–9), Uzbekistan (10–26), China (27–32), Pakistan (33–34), Tajikistan (35), Iran (36), Georgia (37–38), Turkey (39–40), Greece (41–44), Romania (45), Moldova (46), Hungary (47–56), Slovakia (57),France (58–61), Spain (62) and Italy (63–91). (TIF) [file pone.0172541.s001.tif]

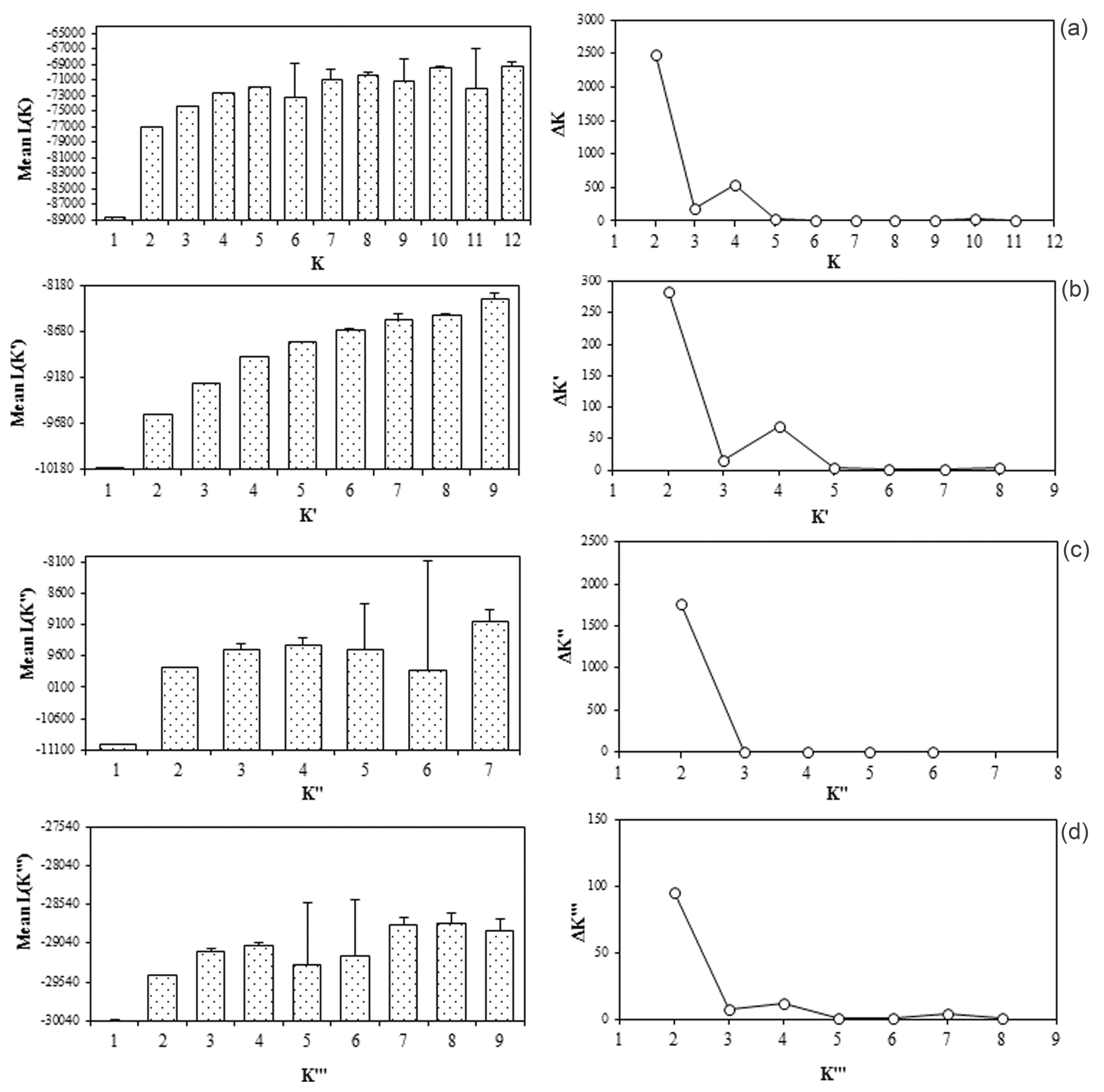

Supplement: S2 Fig — Bayesian Inference of (a) K, the most probable number of clusters, based on microsatellite analysis of all 2,008 common walnut samples, (b) K’, the most probable number of sub-clusters, based on microsatellite analysis of 217 common walnut samples of cluster 1, (c) K”, the most probable number of sub-clusters, based on microsatellite analysis of 280 common walnut samples of cluster 2, and (d) K”‘ the most probable number of sub-clusters, based on microsatellite analysis of 929 common walnut samples of cluster 4 using STRUCTURE software [39]. Log-likelihood value of data L(K) as a function of K averaged over six replicates and second order of change of the log-likelihood of the data (ΔK) as a function of K, calculated over six replicates [40] was reported for each analysis. (TIF) [file pone.0172541.s002.tif]

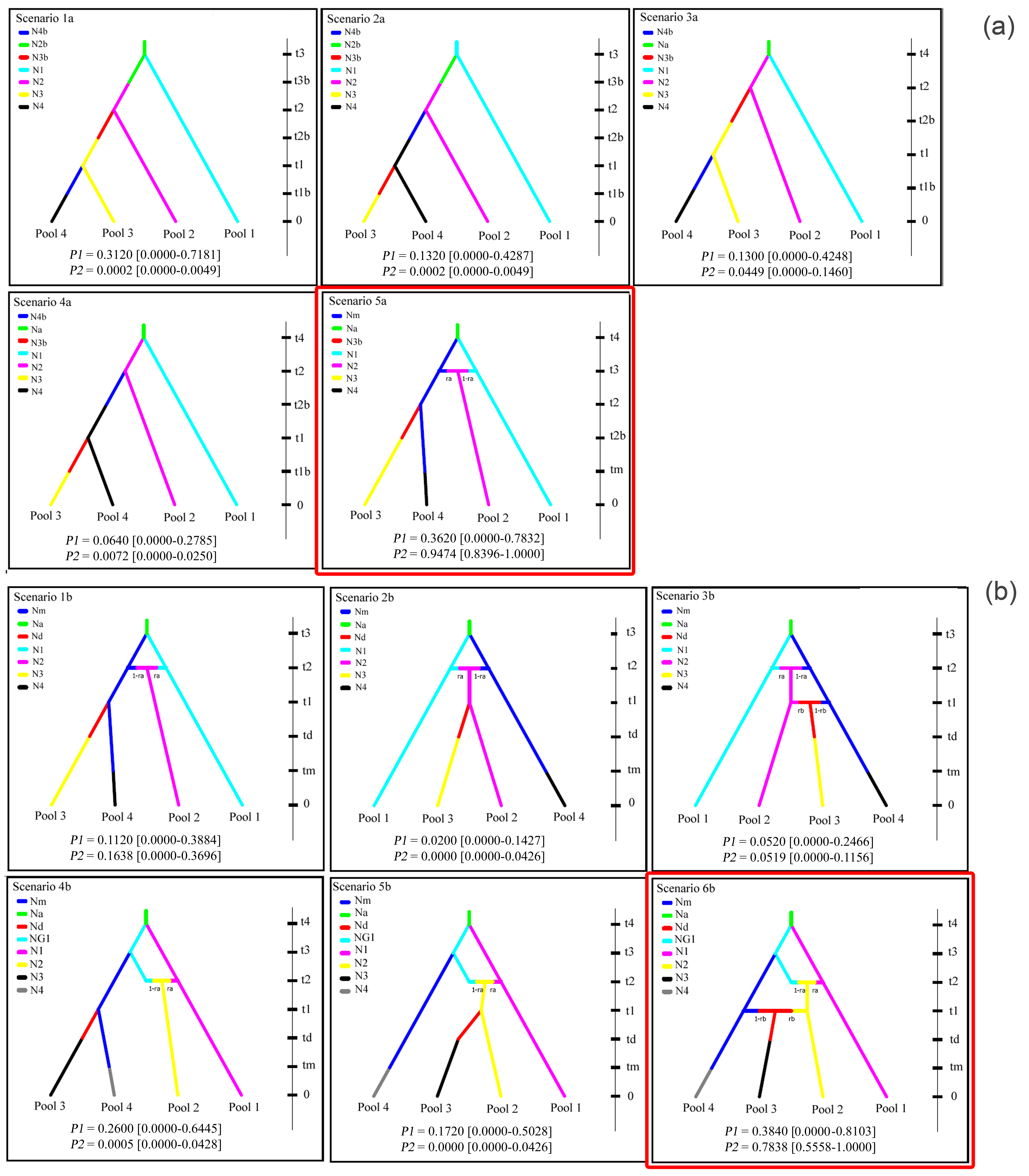

Supplement: S3 Fig — All scenarios tested in stage 1 (a) and stage 2 (b) of DIYABC analysis. In these scenarios, N# refers to effective population size of each corresponding gene pool, and t# refers to time-scale in terms of the number of generations (more details for population parameters and models in S3 and S4 Tables). Posterior probability (P) of each scenario and its 95% confidence interval of P (in brackets) computed using a direct (P1) and logistic regression (P2) approach are provided under each scenario. The most likelihood scenario for each stage is marked with a red rectangle. (TIF) [file pone.0172541.s003.tif]
