# Supplementary material for: Rethinking the history of common walnut (Juglans regia L.) in Europe: Its origins and human interactions
Source: PLoS One. 2017 Mar 3;12(3):e0172541. doi: 10.1371/journal.pone.0172541 (PMC5336217; doi:10.1371/journal.pone.0172541)
Supplement: S1 Table — The geographic coordinates in decimal degrees (Latitude, Longitude), early presence (1700–11.923 Ka BP), first detection of discontinuous and continuous occurrence of Juglans-type fossil pollen (radiocarbon-dating) during the Holocene and the related citation (a) and the presence/absence of Juglans-type fossil pollen and its classification (discontinuous, continuous, in expansion, in contraction and not recorded) in each selected time interval (b) were recorded for each site. (PDF) [file pone.0172541.s004.pdf]

S1 Table. List of 450 fossil pollen sites from Eurasia considered in this study. The geographic coordinates in decimal degrees (Latitude, Longitude), early presence (1700 - 11.923 Ka BP), first detection of discontinuous and continuous occurrence of *Juglans*-type fossil pollen (radiocarbon-dating) during the Holocene and the related citation (a) and the presence/absence of *Juglans*-type fossil pollen and its classification (discontinuous, continuous, in expansion, in contraction and not recorded) in each selected time interval (b) were recorded for each site.

(a)

Country	Site name	Latitude	Longitude	Early presence cal. yr BP	Age disc.[a] cal. yr BP	Age cont. [a] cal. yr BP	Age disc.[a] cal. yr BP	References
Turkey	Lake Manyas	40.200000	28.000000	-	-	2500	-	[1]
	Marmara Sea	41.005178	28.977125	-	4750	2000	-	[1]
	Abant Gölü	40.600000	31.283333	-	-	5311	2100	[2]
	Yeniçaga Gölü	40.783333	32.033333	-	11485	0	0	[2]
	Beysehir Gölü I	37.541667	31.500000	-	-	3505	1462	[2]
	Gölhisar Gölü I	37.133333	29.600000	-	-	4260	0	[2]
	Gölhisar Gölü II	37.133472	29.600000	-	3000	2500	1300	[3]
	Hoyran Gölü	38.275000	30.875000	-	4725	2148	859	[2]
	Kararmik Batakligi	38.425000	30.800000	15367	6186	-	-	[4]
	Köycegiz Gölu	36.875000	28.641667	-	4306	-	-	[2]
	Ova Gölü	36.266667	29.300000	-	-	3538	2517	[2]
	Pinarbasi	37.466667	30.050000	16004	-	4349	0	[2]
	Sögüt Gölü	36.997500	29.898333	16374	-	2647	1290	[4]
	Sagalassos	37.660473	30.525923	-	-	2300	1300	[5]
	Ladik Gölü	40.916667	36.016667	-	9375	5574	-	[2]
	Lake Van	38.500000	43.000000	-	11215	1815	-	[6]
Greece	Kournas	35.330383	24.276198	-	3000	1000	-	[7]
	Asi GoniaII	35.273964	24.283052	-	448	-	-	[8]
	Asi GoniaI	35.273964	24.283000	-	582	-	-	[8]
	Paiko	40.953197	22.335828	-	-	2079	-	[9]
	Lailias	41.595771	24.896196	-	1711	1503	123	[9]
	Elatia-Rhodopes	41.496426	24.314432	-	-	3952	-	[9]
	Kastoria	40.520244	21.268981	-	-	1790	-	[2]
	Orestias	40.526398	21.227013	-	8203	7515	-	[10]
	KhimaditisIII	40.612500	21.586111	-		2730	-	[11]
	Vegoritis	40.771625	21.799904	-	-	2816	-	[2]
	Edessa	40.818056	21.952500	-	-	4244	-	[2]
	Beles	41.319444	23.016111	-	586	522	-	[12]
	Litochoro	40.138889	22.546111	-	-	2953	-	[13]
	Tenaghi Philippon	40.983333	24.783333	-	2782	-	-	[14]
	Lerna	37.550005	22.716667	-	6788	2768	190	[15]
	Trikhonis	38.600000	21.500000	-	-	2052	0	[11]
	IoanninaI	39.762500	20.730556	13474	5264	-	-	[2]
	IoanninaII	39.691944	20.839722	-	7383	746	-	[2]
	Halos	39.166667	22.833333	-	3681	-	-	[2]
	Xinias	39.050000	22.266667	-	8240	4980	-	[16]
	Voulkaria	38.866667	20.833333	-	830	771	340	[17]
	Pertouli	39.524167	21.477500	-	4381	1070	-	[13]
	Myrtoon basin	36.258055	24.258055	18000	9700	6200	-	[243]
Bulgaria	Beliya Kanton	41.733611	24.139722	13524	-	11708	-	[18]
	Kupena I	41.983333	24.333333	-	9350	7250	-	[19]
	Kupena II	41.983347	24.333333	-	9288	-	-	[19]
	Ribno I	41.739079	23.424769	-	8400	2200	-	[20]
	Popovo Ezero	41.716667	23.666667	-	1150	-	-	[21]
	Mutorog	41.516667	23.616667	-	3240	1944	-	[22]
	Trilistnika	42.197750	23.587904	-	-	3350	-	[23]
	Suho Ezero	42.133333	23.416667	-	2275	1160	-	[24]
	Sozopol	42.417263	27.696174	-	-	6139	-	[25]

	Arkutino I	42.366667	27.733333	-	5012	-	-	[26]
	Arkutino II	42.366667	27.733347	-	6210	-	-	[26]
	Black Sea South	42.067500	28.485000	-	1398	-	-	[27]
	Black Sea Southwest	42.184167	28.916667	-	1547	-	-	[27]
	Black Sea West	42.833333	29.916667	-	7382	6475	-	[28]
	Duranunlak II	43.666667	28.550000	-	5823	-	-	[29]
	Duranunlak I	43.666667	28.550000	-	8474	-	-	[29]
	Shabla-Ezeretz	43.583333	28.550000	-	8375	-	-	[29]
	Varna I	44.110956	27.073560	-	5882	-	-	EPD
	Varna II	43.192123	27.807515	-	11332	-	-	EPD
	Srebarna	43.192123	27.807514	-	-	2955	-	[30]
	Mire Garvan	44.116944	26.950000	-	5309	1584	-	[31]
	Maleshevska	41.666667	23.000000	-	-	6750	-	[32]
	Osogovo	42.160450	22.516726	-	-	3500	-	[23]
	Begbunar	42.202577	22.588840	-	-	5027	-	[33]
	Sredna Gora	42.500000	25.000000	-	1313	368	-	[34]
	Straldzha mire	42.630833	26.772778	-	3805	-	-	[35]
	Tchokljovo Marsh	42.366667	22.833333	-	6415	-	-	[36]
	Vitosha	42.566658	23.283323	-	-	557	-	[37]
Romania	AvrigI	45.727863	24.377838	-	3056	-	-	[38]
	AvrigII	45.721950	24.320583	-	888	-	-	[38]
	Mohos	46.132081	25.904150	-	232	88	-	[39]
	Luci	46.296944	25.737500	-	2100	-	-	[40]
	Bisoca	45.553231	26.687611	-	600	380	-	[41]
	Calineasa	46.608562	22.764285	-	-	365	-	[42]
	Semenic	45.090515	21.996336	-	3299	-	-	[43]
	Steregoiu	47.813889	23.540278	-	361	-	-	[44]
Hungary	Alsópáhok	46.774444	17.170278	-	-	1750	-	[45]
	Balaton I	46.744444	17.400833	-	3885	1720	-	EPD
	Balaton II	47.001667	18.104167	-	3500	2500	-	EPD
	Balaton III	46.608567	17.735000	-	1408	1162	-	EPD
	Nagy-Mohos	48.326944	20.436389	-	3573	-	-	[46]
	Pölöske	46.756111	16.924722	-	1195	-	-	[47]
	Pötréte	46.678889	16.933056	-	2300	-	-	[46]
	Szigliget	46.800000	17.433333	-	-	4086	-	[46]
Slovakia	Bobrov	49.445833	19.566667	-	245	-	-	[48]
	Zlatnicka Dolina	49.516667	19.283333	-	421	-	-	[48]
	Strbské pleso	49.122222	20.055556	-	554	-	-	[49]
	Tlstá hora	48.894167	17.888611	-	4927	2289	-	[50]
Czech Republic	Královec	49.131944	18.027778	-	1096	438	-	[50]
	Machová	48.830833	17.541111	-	1100	403	-	[50]
	Dvur Ansov	48.791667	16.387500	-	3521	-	-	[51]
	Olbramovice	48.991667	16.400000	-	3224	-	-	[52]
	Vracov	48.977778	17.202778	-	3370	1204	-	[52]
	Palasiny	49.688889	15.483333	-	477	-	-	[52]
	Blato I	49.041667	15.191667	-	6472	-	-	[53]
	Kozli	49.376389	14.025833	-	1312	-	-	[54]
	Rezabinec	49.250000	14.116667	-	976	-	-	[55]
	Borkovicka blata	49.216667	14.900000	-	6310	254	-	[56]
	Branna	48.950000	14.933333	-	3091	-	-	[57]
	Loucky	49.325000	15.502778	-	4842	-	-	[57]
	Chrást	50.227222	14.544167	-	6092	-	-	[58]
	Velka niva	48.924167	13.818611	-	826	-	-	[59]
	Malá niva	48.913889	13.816111	-	322	-	-	[60]
	Mrtv8 luh	48.866944	13.883056	-	585	-	-	[60]
	Strázenská slat	48.898889	13.742222	-	1875	-	-	[61]
	Komoranské jezero	50.500000	13.500000	-	1402	-	-	[61]
	Dolskym	50.852500	14.338889	-	10546	-	-	[62]
	Na bahne	50.198889	15.961389	-	110	-	-	[63]
	Velky Ded	50.083333	17.216667	-	-	1414	-	[64]
	Velky Maj	50.050000	17.216667	-	-	1756	1695	EPD
	Pancavská louka	50.766389	15.541111	-	713	-	-	EPD
	Pryskyricn8 dul	50.887778	14.413333	-	78	-	-	[65]
	Tpské raseliniste Mire	50.738889	15.71250	-	-	929	94	[66]
	Tpské raseliniste MireII	50.738889	15.712500	-	3543	-	-	[65]
	Tpské raseliniste MireIII	50.738889	15.712500	-	1866	433	-	[60]
Ukraine	Dovjok Swamp	48.750000	28.250000	-	1016	-	-	[67]
	Maly Podleski	49.916667	24.016667	-	6700	-	-	[68]
	Pecheniya	49.666667	23.933333	-	3600	-	-	[68]
	Yukharina Balka	44.551111	33.488056	-	-	1450	-	[69]
Croatia	Bokanjacko	44.183333	15.233333	-	2401	-	-	[70]
	Mljet	42.776667	17.347325	-	-	3250	-	[71]
Poland	Bledowo Lake	52.550000	20.666667	-	893	-	-	[72]
	Giecz	52.319444	17.363333	-	1260	-	-	[73]
	Lake Gosciaz	52.583333	19.35000	-	7994	-	-	[74]
	Lake Skrzetuszewskie	52.550000	17.360556	-	1468	-	-	EPD

	Slawsko	52.666667	18.250000	-	2610	-	-	[75]
	Swietokrzyskie Lake	52.544444	17.598611	-	679	214	-	EPD
	Cergowa Gora	49.533333	21.700000	-	4355	-	-	[76]
	Godziszewskie Lake	54.093333	18.552778	-	167	-	-	[77]
	Lake Mikolajki	53.768056	21.418056	-	100	-	-	[78]
	Puscizna Rekowianska	49.483333	19.816667	-	-	203	-	[79]
Austria	Buntes Moor	47.000000	11.141667	-	3141	1888	-	[80]
	Dortmunder Hütte	47.125095	11.003877	-	1375	-	-	[81]
	Franz Senn-Hütte	47.058366	11.167340	-	-	1073	-	[80]
	Egelsee	47.612500	12.170833	-	1912	1726	-	[82]
	Gerlos	47.243056	12.138889	-	-	1626	571	[83]
	Giering	47.471389	12.358333	-	751	-	-	[85]
	Gradenmoos	48.182082	12.921333	-	-	1357	-	[86]
	Grünau Moor	46.984167	11.190556	-	1290	-	-	[80]
	Lindenmoos	47.509722	12.043056	-	1803	894	-	[83]
	Mieminger See	47.291667	10.976389	-	-	1162	332	[82]
	Moor Alpenrose	47.050000	11.758333	-	2582	-	-	[80]
	Rotmoos Obergurgl	46.841667	11.025000	-	643	385	-	[83]
	Schwarzsee Reschenscheideck	46.870833	10.480556	-	1898	-	-	[84]
	Schwemm	47.658333	12.300000	-	-	1127	684	[87]
	Seefelder See	47.323611	11.191667	-	11645	1862	1089	[82]
	Zirbenwaldmoor	46.858333	11.025000	-	2120	446	-	[88]
	Wasenmoos beim Zellhof	47.986111	13.105556	-	4702	698	122	[89]
	Grosses Überling Schattseit-Moor	47.172222	13.900000	-	730	-	-	[90]
	Dürrenecksee-Moor	47.173611	13.875000	-	4398	-	-	[90]
	Fuchsschwanzmoos	47.123611	13.905556	-	11267	-	-	[90]
	Fuschlsee	47.783333	13.266667	-	-	1279	-	[91]
Germany	Ahlenmoor	53.700000	8.733333	-	1138	-	-	[92]
	Ahlequellmoor	51.730556	9.509444	-	418	-	-	[93]
	Bruchberg	51.758889	10.460000	-	1027	-	-	[94]
	Lüttersee	51.576667	10.161667	-	1126	250	-	[95]
	Silberhohl	51.910000	10.182500	-	2738	-	-	[95]
	Sonnenberger Moor	51.768056	10.516111	-	656	-	-	[94]
	Dunum (Hilliges Moor)	53.583333	7.633333	-	651	-	-	[92]
	Felchosee	53.050000	14.133333	-	986	-	-	[96]
	Großer Krebssee	52.850000	14.100000	-	1119	-	-	[96]
	Löddigsee	53.433333	11.850000	-	748	-	-	[97]
	Wachel 3	53.438889	8.868889	-	-	251	-	[98]
	Brentenlohe	49.787222	12.462500	-	2188	209	-	[99]
	Bruckmisse	48.732500	8.644167	-	3064	-	-	[100]
	Glaswaldsee	48.426667	8.249167	-	2273	1055	-	[100]
	Herrenwiesser see	48.669167	8.296389	-	2857	1447	-	[101]
	Durchenbergried	47.783333	8.983333	-	2107	-	-	[102]
	Feuenried	47.750000	8.916667	-	10005	370	-	[102]
	Gaienhofen	47.679444	8.975833	-	1269	-	-	[103]
	Hornstaad-Bodensee	47.700000	9.016667	-	4190	1520	-	[102]
	Huzenbacher See	48.574444	8.348056	-	2416	-	-	[104]
	Mindelsee	47.755556	9.023056	-	2064	1852	-	[105]
	Oberderdingen-Großvillars	49.042500	8.760278	-	1270	-	-	[106]
	Steerenmoos	47.800000	8.200000	-	3642	-	-	[107]
	Wilder See beim Ruhestein	48.569722	8.236944	-	1449	1074	-	[100]
	Wildseemoor bei Kaltenbronn	48.719722	8.458889	-	5476	-	-	[100]
Switzerland	Derrière les Embreux	47.263056	7.117222	-	-	587	-	[108]
	Etang de la Gruère	47.240556	7.050000	-	2881	-	-	[109]
	Le Loclat	47.020833	6.997222	-	-	2208	-	[110]
	Lobsigensee	47.030556	7.298056	13201	-	2391	-	[111]
	Montilier	46.935000	7.123611	14017	-	2304	-	[112]
	Aegelsee	46.648611	7.543611	-	134	-	-	[113]
	Amsoldingersee	46.722778	7.576944	-	1438	-	-	EPD
	Bachalpsee	46.669444	8.020833	-	1754	1225	-	[114]
	Hinterburgseeli	46.718889	8.068889	-	2097	1706	-	[84]
	Hängstli	46.794167	7.833056	-	1619	-	-	[115]
	Linden	46.850833	7.686111	-	2657	971	-	[84]
	Oberaar	46.547222	8.255556	-	1030	-	-	[116]
	Rotsee	47.075833	8.325833	-	2603	1863	-	[113]
	Schwarzsee FR	46.670278	7.268056	-	2195	557	-	[117]
	Schöpfenwaldmoor	46.743611	7.847778	-	1043	412	-	[118]
	Sägistalsee	46.681389	7.977500	-	1886	1121	-	[118]
	Süftenenegg	46.734444	7.398889	-	1882	500	-	[119]
	Trogenmoos	46.761944	7.863889	-	500	444	-	[118]
	Aletschwald	46.389722	8.025556	-	5117	1563	-	[84]
	Alp Lüsga Belalp 1	46.230833	7.590278	-	1321	-	-	[84]
	Alpi di Robièi Val Bavona	46.445000	8.518333	-	647	355	-	[84]
	Alpi di Robièi Val Bavona Bodenprofil	46.445000	8.518056	-	2000	-	-	[119]

	Bitsch-Naters	46.341111	7.991667	-	1928	1767	-	[84]
	Eggen ob Blatten	46.371944	7.990556	-	7339	1108	-	[84]
	Etang d'y Cor Montana	46.311667	7.479167	14763	2139	1585	-	[84]
	Etang de Luissel Bex	46.237222	7.017500	-	6543	2285	-	[119]
	Gondo Alpjen	46.211667	8.113333	-	4396	1449	-	[84]
	Greicheralp Riederalp	46.379722	8.030278	-	3659	3341	-	[84]
	Grächen See	46.195556	7.845000	-	2337	1471	815	[119]
	Hopschensee	46.252500	8.023056	-	-	2336	-	[84]
	Lac du Mont d'Orge Sion	46.234444	7.341389	12511	10399	2647	-	[84]
	Mittlere Hellelen	46.283056	7.844167	-	3344	1508	-	[84]
	Pillon Gsteig-Diablerets	46.359722	7.198611	-	3084	1226	-	[84]
	Simplon-Gampisch-Alter Spittel	46.231111	8.011389	-	2747	-	-	[84]
	Wallbach Lenk	46.428333	7.403056	-	1483	1483	0	[84]
	Mont Roux	46.468056	6.150000	13373	-	1381	-	[120]
	Gamperfin	47.171389	9.381111	-	1424	-	-	[121]
	Creux de Croue	46.500000	6.128333	-	-	1554	1146	[84]
	Motta Naluns	46.810833	10.264722	-	2017	-	-	[118]
	Praz Rodet	46.566667	6.173611	-	2249	427	-	[108]
Italy	Bondone	46.014444	11.045833	-	-	3931	-	[124]
	Dura-Moor	46.640000	11.458889	-	2336	1551	-	[125]
	MalschötscherHotter	46.666111	11.458333	-	-	2308	-	[125]
	Rinderplatz	46.644722	11.494444	-	1477	1197	-	[125]
	Schwarzsee	46.666389	11.431944	-	2508	1792	-	[125]
	Sommersüss	46.760833	11.678333	-	2634	1183	-	[125]
	Grunsee	46.862778	10.478056	-	7691	-	-	[122]
	DossaccioBormio	46.470556	10.337222	-	-	1835	-	[84]
	Lac de Lod	45.802500	7.6097220	-	-	1182	-	[126]
	Lac de Villa	45.684722	7.7611110	-	-	2262	-	[126]
	Lac du Verney-Dessus	45.693333	6.8725000	-	2156	-	-	[127]
	Torveraz	45.695278	6.8605560	-	2366	-	-	[127]
	Tourbière de Pilaz	45.816944	7.8333330	-	-	9756	-	[126]
	Tourbière de Santa Anna	45.858333	7.6541670	-	793	-	-	[126]
	Laghi dell'Orgials	44.233333	7.1333330	-	1802	-	-	[128]
	Lago Grande di Avigliana	45.065000	7.3866670	-	-	122	-	[129]
	Lago Piccolo di Avigliana	45.053333	7.3841670	-	-	2203	-	[129]
	Ortasee	45.816667	8.4000000	-	7329	-	-	[130]
	OrtaseeII	45.816670	8.4000000	-	2175	1925	-	[130]
	Refugio Mondovi	44.380960	7.8211450	-	-	1758	-	[128]
	Selle di Carnino	44.150000	7.6944440	11923	11923	970	-	[131]
	Torbiera del Biecai	44.200000	7.7000000	-	5507	3287	-	[128]
	Lago della Costa	45.231274	11.737721	-	-	6300	-	[132]
	Lago Padule	44.298611	10.214722	-	1680	0	-	[133]
	Lago dell'Accesa	42.986667	10.891667	-	8437	3146	-	[134]
	Colfiorito	43.025000	12.925000	-	3913	1998	472	[135]
	Lago Pratignano	44.176111	10.819722	-	3542	-	-	EPD
	Ospitale	44.146513	10.786631	-	1303	-	-	EPD
	Pavullo	44.318333	10.837500	-	4200	1000	-	[136]
	Parma	44.711209	10.312795	-	2000	-	-	[137]
	Lago di Martignano	42.116667	12.333333	-	11498	6560	2814	[138]
	Lago di Vico	42.324823	12.182378	-	-	2630	-	[112]
	Lago Albano	41.750000	12.666667	-	8402	3400	-	[139]
	Lago di Nemi	41.712222	12.702500	-	11300	2700	-	[139]
	Central Adriatic Sea	42.666944	15.667500	-	6400	2032	-	[139]
	Salerno Bay	40.472778	14.706667	20000	4200	-	-	[140]
	Lago Grande di Monticchio	40.944444	15.600000	132000	2520	1852	-	[141]
	BradanoValley	40.652161	16.592063	-	-	2250	2000	[142]
France	Aronde	49.462500	2.691111	-	2216	-	-	EPD
	Auneau	48.456111	1.793611	-	1101	-	-	EPD
	Baie de Seine estuary	49.416667	-0.033333	-	-	1759	-	[143]
	Change-Glatinier	48.116667	-0.788889	-	1941	822	152	[144]
	Coulvain	49.066667	-0.716667	-	1052	-	-	EPD
	Fougères	48.516667	-0.833333	-	634	-	-	[145]
	La Verderie	48.350000	-0.427778	-	1370	876	-	[144]
	La Vie	48.548333	-0.258333	13390	2952	-	-	[144]
	Lavaré	48.433333	-0.916667	-	1389	-	-	[144]
	Le Fourneau	48.444444	-0.191667	17204	5535	-	-	[144]
	Lingreville	48.929722	-1.543056	-	2696	507	-	[146]
	Malingue	48.438889	-0.575000	-	1823	1765	-	[144]
	Marais de Kerdual	47.586667	-3.051667	-	3725	-	-	[147]
	Marais de Lisle	47.429722	-1.494444	-	5147	694	-	[148]
	Marais de Marchesieux	49.173333	-1.300000	-	2768	-	-	[149]
	Mobeche Forest	48.516667	-1.000000	-	891	-	-	[145]
	Moulin de Thévalles	47.969444	-0.416389	-	912	769	-	[150]
	Pezou	47.870833	1.150833	-	2092	825	37	[151]
	Rimarde	48.100556	2.310000	-	2191	1180	783	[152]

Saint-Ursin	48.519444	-0.253333	-	2250	-	-	[144]
Serrent	47.809444	-2.468056	-	566	-	-	EPD
Altenweiher	48.013333	6.994444	-	4288	2632	-	[153]
Bellefontaine	46.575278	6.093056	-	8364	-	-	[123]
Grozon	46.890556	5.700278	-	2708	1594	-	[154]
Hières sur Amby	45.790833	5.283333	-	3059	-	-	[155]
Lac Cerin	45.777222	5.561944	-	-	1760	-	[156]
Lac de Clairvaux	46.565000	5.749167	-	904	666	-	[154]
Lac de Malpas	46.833889	6.293889	-	1605	944	378	[154]
Lake of Annecy	45.856667	6.172222	-	2460	1966	-	[157]
Loras	45.666389	5.243333	-	2834	2078	-	[155]
Lutinière	46.444444	6.411059	-	1837	974	-	EPD
Moselotte	48.031944	7.000000	-	1609	790	244	[153]
Tourbière de Narbief	47.080278	6.697500	-	2840	1541	836	[154]
col du Petit Saint Bernard	45.671944	6.875556	-	1800	1146	491	[158]
Etang du Lautrey	46.587222	5.863889	-	11740	-	-	[123].
La Beuffarde	46.823611	6.423056	-	1861	1487	12	[127]
Le Grand Lemps	45.473333	5.416667	-	-	2085	-	[154]
Tourbière de Censeau	46.813889	6.055278	-	1454	527	225	[155]
Tourbière du Mou de Pleure	46.913889	5.450556	-	1401	594	298	[154]
Tourbières des Granges des Chavants	45.893056	6.768333	-	10581	913	-	[154]
Ampoix	45.633333	2.933333	-	5530	-	-	[159]
Champ Gazon	47.203333	4.048611	-	3781	707	-	[160]
Etang Bouquin	47.273611	4.018611	-	2029	878	-	[161]
Etang de Cheylade	45.090000	2.895000	-	-	2647	1727	[161]
Etang de la Villetelle	46.023889	1.707778	-	1447	1048	-	[162]
La Taphanel	45.274444	2.679167	-	2394	-	-	[163]
Lac du Bouchet	44.916667	3.783333	-	-	792	-	[164]
Lac du Mont de Belier	45.337778	2.643056	-	3381	-	-	[165]
Le Grand Montarnu	47.011944	4.050000	-	-	881	-	[164]
Le Jolan	45.139444	2.859167	-	-	881	-	[161]
Le Miroir	46.539444	5.326667	-	805	-	-	[164]
Les Cars	45.607500	2.375000	-	588	-	-	[166]
Les Chaux de Coudert	45.274444	1.946389	-	-	2073	-	[163]
Les Nans	46.789722	5.974444	-	-	1346	-	[163]
Marais de Maurepas	48.102222	2.582778	-	1713	823	-	[154]
Marais du Grang Chaumet	46.937500	1.891389	-	9097	3828	-	[152]
Mars	45.305278	1.836111	-	1575	598	-	[167]
Montbé	47.214722	4.106944	-	1533	740	211	[168]
Moulin de Prugnolas	45.849722	1.645833	-	3421	-	-	[161]
Nataloup	47.227500	4.035278	-	1171	354	-	[169]
Peyre peat-bog	44.960278	2.718889	-	-	1428	300	[161]
Port des Lamberts	46.960833	4.010556	-	2402	1175	-	[170]
Quart du Bois	46.892500	4.034167	-	1249	455	-	[171]
Saint-Benoit-sur-Loire	47.810278	2.319167	-	2348	2107	-	[161]
Sources de l'Yonne	46.956389	4.010000	-	1600	709	69	[172]
Tourbière de Longeyroux	45.601944	2.102500	-	1745	-	-	[161]
Tourbière de Roussy	45.076111	2.517500	-	9466	1298	-	[168]
Tourbière des Dauges	46.012500	1.416667	-	-	1801	466	[173]
La Baforière	46.485019	-0.305556	-	-	2254	-	[144]
Tourbière de Chabannes	45.649167	2.310556	-	-	1432	216	[173]
Font Carluze	45.540833	2.344444	-	1137	-	-	[174]
Ancenis	47.382500	-1.165000	-	5581	-	-	[175]
Basse-Ville	47.186111	-1.858056	-	771	514	-	[176]
Bois-Jésus	47.413333	0.638056	-	1082	-	-	[177]
Carquefou	47.309167	-1.476944	-	1642	379	-	[179]
Caves d'Amont	47.326667	0.475278	-	8535	713	581	[180]
Changeon	47.276111	0.131944	-	-	1178	-	[175]
Cinq-Mars-la-Pile	47.337778	0.451111	-	8979	2286	420	[180]
Cordemais	47.280833	-1.876111	-	1789	1328	-	[181]
Corniche de Pail	48.416667	-0.211111	-	-	1488	-	[144]
Ecours	47.382500	-1.165000	-	7970	-	-	[158]
Jaunay	46.662778	-1.892222	-	8636	166	-	[158]
La Bergerie en Charron	46.301111	-1.043889	-	2723	1400	1228	[182]
La Caudelais	47.261111	-1.780556	-	6268	1836	-	[176]
La Grande Brousse	47.486667	0.674167	-	3252	-	-	[175]
La Grange	46.089722	-0.775278	-	5803	-	-	[158]
La Prairie du Cassoir	47.256667	0.147500	-	1882	828	652	[178]
La boire Torse	47.386667	-1.070000	-	5704	1640	-	[183]
Le Gesvres	47.272222	-1.595833	-	6556	703	-	[184]
Le Marais de la Perge	45.382500	-1.115000	-	1169	-	-	[185]
Les Naudières	47.537500	0.702222	-	1308	-	-	[186]
Logne	47.328333	-1.501111	-	3098	-	-	EPD
Marais de Champtocé	47.413611	-0.860556	-	2560	908	-	[175]
Marais de Mazerolles	47.358056	-1.478611	-	1420	699	-	[184]

	Marais de Munet	47.221667	-0.095833	-	1162	579	-	[175]
	Marais de Méron	47.380556	-1.123333	-	5574	803	-	[184]
	Marais de la Poupinière	47.402778	-1.502778	-	938	-	-	[158]
	Marais des Bourbes	46.550000	-1.816667	-	2601	296	131	[184]
	Oudon	47.355556	-1.293611	-	2210	1298	145	[187]
	Pas du Gu	47.238333	-2.150000	-	1079	-	-	[188]
	Petit Marais	47.217222	-2.102222	-	2766	-	-	[158]
	Petit Rocher	46.663056	-1.915556	-	6969	-	-	[158]
	Riabelais	47.402500	0.648333	-	1710	-	-	[187]
	Saint Viaud Contin	47.265000	-2.016667	-	1546	-	-	[148]
	Tourbière de Nay	47.310556	-1.534167	-	1367	751	459	[189]
	Tourbière de Parcay-sur-Vienne	47.092222	0.480556	-	9334	-	-	[131]
	Vertonne	46.549722	-1.764722	-	2970	1043	-	[190]
	Clapeyret	44.147222	7.238889	-	3059	-	-	[131]
	Col Luitel	45.088333	5.849722	-	-	2544	-	[191]
	Col des Lauzes	44.767500	6.536944	-	6036	2561	-	[192]
	Correo	44.508333	5.983056	-	1871	-	-	[193]
	Embouchac	43.566389	3.916667	-	6556	-	-	[194]
	Etang d'Ouveillan	43.266667	3.000000	-	3972	-	-	[155]
	Fangeas	47.355556	-1.293611	-	1195	1079	-	[131]
	Grand Ratz le Pellet	45.425000	5.608333	-	1295	1022	-	[191]
	Lac Long Inférieur	44.057778	7.450000	-	2955	-	-	EPD
	Lac Miroir	44.635278	6.793889	-	3848	-	-	[191]
	Lac Saint Léger	44.420000	6.336389	-	4861	1234	-	[191]
	Lac de Praver	45.073611	5.856389	-	5207	2003	-	[195]
	Lac des Boites	45.056111	5.885278	-	-	2729	-	[154]
	Lac du Lauzon	44.675278	5.793333	-	6698	2164	1572	[191]
	Lake Racou	42.554167	2.008333	-	-	1260	-	EPD
	Lignin Lake	44.104167	6.708611	-	7864	7545	-	[178]
	Marais de Charauze	45.368333	5.566944	-	870	777	-	[131]
	Pelléautier	44.522222	6.183333	-	987	688	-	[191]
	Peuil Peat Bog	45.125000	5.643611	-	6679	-	-	[196]
	Pré Rond	44.918889	6.594167	-	602	201	-	[131]
	Sabbion	44.130000	7.473333	-	2979	-	-	[164]
	Saint Hilaire du Rosier	45.070833	5.256667	-	2234	-	-	[155]
	Saint Julien de Ratz	45.348333	5.655278	-	-	4637	-	[155]
	Saint Sixte	45.425833	5.628611	-	5600	1765	-	[173]
	Tourbière de Gatimort	43.575278	2.785556	12595	1328	851	-	[191]
	Tourbière de Mont Sec	45.068889	5.806667	-	11114	698	-	[191]
	Tourbière de Raux	44.503333	5.935278	-	1984	1332	245	[197]
	Tourbière de la Lande	43.566667	2.966667	-	1164	835	-	[197]
	Tourbière des Narses Mortes	44.433333	3.600000	-	1823	-	-	[198]
	Tourbière du Peschio	44.450000	3.600000	-	976	698	-	[197]
Spain	Albufera Alcudia	39.792778	3.119167	-	2617	-	-	[199]
	Algendar	39.940556	3.958611	-	6350	-	-	[200]
	Cala Galdana	39.936944	3.965000	-	2119	-	-	[201]
	Cala'n Porter	39.870556	4.131389	-	4450	-	-	[200]
	Antas	37.208333	-1.823611	-	8029	-	-	[202]
	Bajondillo	36.619722	-4.496389	-	-	-	-	[203]
	Delta del Rio Besos	41.380278	2.248333	134950	902	-	-	[204]
	El Pirulejo	37.438889	-4.195000	15000	-	-	-	[205]
	Laguna Salada Chiprana	41.233333	-0.166667	-	-	1788	857	EPD
	Navarrés	39.100000	-0.683333	-	4149	-	-	[206]
	Carihuela Cave	37.139183	-3.596765	0.12 Ma	-	-	-	[207]
	Cueva Perneras	37.535556	-1.430000	12000	-	-	-	[208]
	Atxuri	43.250000	-1.550000	-	811	-	-	[209]
	Cueto de la Avellanosa	43.116667	-4.364167	-	-	2231	-	[210]
	Lago de Ajo	43.050000	-6.150000	-	3948	2871	-	[211]
	Laguna de la Roya	42.216667	-6.766667	-	1751	884	-	[211]
	PRD-4	42.533333	-8.516667	-	11499	-	-	[212]
	Posidonia Lligat	42.292222	-3.291111	-	658	-	-	[213]
	Puerto de Los Tornos	43.150000	-3.433333	-	1323	991	-	[214]
	Saldropo	43.050000	-2.716667	-	999	893	-	[209]
	Sanabria Marsh	42.100000	-6.733333	-	8721	-	-	[211]
	El Payo	40.253333	-6.771111	-	2598	-	-	[215]
	Lanzahíta	40.222222	-4.935833	-	-	2434	-	[205]
	Patateros bog	39.597222	-4.674167	-	1064	-	-	[216]
	Peña Negra	40.334722	-5.792222	-	4077	1011	-	[217]
	Puerto de Serranillos	40.307222	-4.934167	-	247	-	-	[218]
	Quintanar de la Sierra	42.033333	-3.016667	-	2632	-	-	[209]
	Salada Pequeña	41.033333	-0.216667	-	2727	-	-	EPD
	Turbera de La Panera Cabras	40.165833	-5.758056	-	124	-	-	[215]
Portugal	Charco da Candieira	40.341667	-7.576389	-	1410	-	-	[219]
	Lagoa Comprida 2	40.362778	-7.636111	-	7734	-	-	[220]
	Lagoa Travessa I	38.304444	-8.772500	-	6239	-	-	[221]

Syria	Bouara	35.233333	41.183333	-	2990	-	-	[222]
	Ghab	35.683333	36.300000	62850	11250	-	-	[223]
Israel	Dead Sea-Ze'elim	31.333333	35.500000	-	-	2344	1300	[224]
	Birkat Ram	33.250000	35.666667	-	6840	2586	-	[225]
	Lake Kinneret	32.768712	35.592129	-	2350	-	-	[226]
	Hula Valley	33.085768	35.610141	115000	5000	-	-	[227]
Iran	Gomishan	37.151667	54.056667	-	2414	-	-	[228]
	Lake Almalou	37.665278	46.631944	-	2026	1701	-	[229]
	Lake Urmia	37.793611	45.375833	200405	8632	3966	-	[230]
	Lake Zeribar	35.533333	46.116667	-	3214	-	-	EPD
	Maharlou Lake	29.477222	52.759722	-	4105	2964	-	[229]
Iraq	Shanidar Cave	36.611914	44.194742	46000	-	-	-	[231]
Georgia	Adange	43.305556	41.333333	-	3945	-	-	[232]
	Amtkel	43.283333	41.291667	-	2295	-	-	[232]
	Gagra	43.283333	40.266667	-	5754	-	-	[233]
	Khodzal	42.954167	41.911111	-	441	-	-	[232]
	Lagodekhi	41.914444	46.373611	-	73	-	-	[234]
	Sibista	43.233333	41.430556	-	1633	-	-	[232]
	Dzudzuana Cave	42.500000	43.516667	24000	-	-	-	[235]
	Dmanisi	41.316667	44.350000	1.7 Ma	-	-	-	[236]
	Akhali Ateni	43.000000	40.983333	-	6300	-	-	[237]
	Supsa River	42.009167	41.860278	-	1260	-	-	[237]
	Lake Bazaleti	42.039000	44.678000	-	-	2570	-	[237]
	Imera lake	41.591389	44.086944	-	-	2360	-	[237]
Albania	Lake Maliq	40.766666	20.783333	3611	-	-	-	[238]
	Lake Ohrid	40.900000	20.800000	170000	-	-	-	[239]

(b)

Interval Time	Country	Site name	Classified [b]
1700-11.923 Ka BP			
	Turkey	Abant Gölü	Not recorded
		Yeniçaga Gölü	Not recorded
		Gölhisar Gölü I	Not recorded
		Kararmik Batakligi	Early presence
		Pinarbasi	Early presence
		Sögüt Gölü	Early presence
		Ladik Gölü	Not recorded
	Greece	Orestias	not recorded
		Ioannina I	Early presence
		Ioannina II	Not recorded
		Xinias	Not recorded
		Myrtoon basin	Early presence
	Bulgary	Beliya Kanton	Early presence
		Kupena I	Not recorded
		Kupena II	Not recorded
		Black Sea South	Not recorded
		Varna II	Not recorded
	Romania	Avrig I	Not recorded
		Mohos	Not recorded
		Luci	Not recorded
		Bisoca	Not recorded
		Steregoiu	Not recorded
	Hungary	Alsópáhok	Not recorded
		Balaton Centre	Not recorded
		Balaton Southwest	Not recorded
		Nagy-Mohos	Not recorded
		Pötréte	Not recorded
		Szigliget	Not recorded
	Czech Republic	Vracov	Not recorded
		Borkovicka blata	Not recorded
		Chrást	Not recorded
		Velka niva	Not recorded
		Strázenská slat	Not recorded
	Poland	Bledowo Lake	Not recorded
		Lake Gosciaz	Not recorded
		Lake Mikolajki	Not recorded
	Austria	Egelsee	Not recorded
		Gerlos	Not recorded
		Giering	Not recorded
		Lindenmoos	Not recorded
		Mieminger See	Not recorded
		Moor Alpenrose	Not recorded

	Schwarzsee Reschenscheideck	Not recorded
	Schwemm	Not recorded
	Seefelder See	Not recorded
	Grosses Überling Schattseit-Moor	Not recorded
	Fuchsschwanzmoos	Not recorded
	Fuschlsee	Not recorded
Germany	Lüttersee	Not recorded
	Löddigsee	Not recorded
	Brentenlohe	Not recorded
	Durchenbergried	Not recorded
	Feuenried	Not recorded
	Hornstaad-Bodensee	Not recorded
Switzerland	Etang de la Gruère	Not recorded
	Le Loclat	Not recorded
	Lobsigensee	Early presence
	Montilier	Early presence
	Aegelsee	Not recorded
	Amsoldingersee	Not recorded
	Bachalpsee	Not recorded
	Hinterburgseeli	Not recorded
	Linden	Not recorded
	Etang d'y Cor Montana	Early presence
	Hopschensee	Not recorded
	Lac du Mont d'Orge Sion	Early presence
	Mittlere Hellelen	Not recorded
	Pillon Gsteig-Diablerets	Not recorded
	Wallbach Lenk	Not recorded
	Mont Roux	Early presence
	Motta Naluns	Not recorded
	Praz Rodet	Not recorded
Italy	Bondone	Not recorded
	Dura-Moor	Not recorded
	Malschötscher Hotter	Not recorded
	Rinderplatz	Not recorded
	Sommersüss	Not recorded
	Grunsee	Not recorded
	Dossaccio Bormio	Not recorded
	Lac de Villa	Not recorded
	Laghi dell'Orgials	Not recorded
	Lago Piccolo di Avigliana	Not recorded
	Ortasee	Not recorded
	Refugio Mondovi	Not recorded
	Selle di Carnino	Early presence
	Torbiera del Biecai	Not recorded
	Lago della Costa	Not recorded
	Lago dell'Accesa	Not recorded
	Colfiorito	Not recorded
	Lago di Vico	Not recorded
	Lago Albano	Not recorded
	Salerno Bay	Early presence
	Lago Grande di Monticchio LGM	Early presence
France	La Vie	Early presence
	Le Fourneau	Early presence
	Pezou	Not recorded
	Saint-Ursin	Not recorded
	Altenweiher	Not recorded
	Bellefontaine	Not recorded
	Hières sur Amby	Not recorded
	Loras	Not recorded
	Etang du Lautrey	Not recorded
	Tourbières des Granges des Chavants	Not recorded
	Champ Gazon	Not recorded
	Etang de Cheylade	Not recorded
	Lac du Bouchet	Not recorded
	Le Miroir	Not recorded
	Marais du Grang Chaumet	Not recorded
	Nataloup	Not recorded
	Peyre peat-bog	Not recorded
	Saint-Benoit-sur-Loire	Not recorded
	Tourbière des Dauges	Not recorded
	La Grande Brousse	Not recorded
	Marais de Munet	Not recorded
	Marais de la Poupinière	Not recorded
	Tourbière de Parcay-sur-Vienne	Not recorded
	Col des Lauzes	Not recorded
	Correo	Not recorded

		Grand Ratz le Pellet	Not recorded
		Lac Long Inférieur	Not recorded
		Lac des Boites	Not recorded
		Lake Racou	Not recorded
		Peuil Peat Bog	Not recorded
		Saint Hilaire du Rosier	Not recorded
		Saint Julien de Ratz	Not recorded
		Tourbière de Gatimort	Early presence
		Tourbière de Mont Sec	Not recorded
	Spain	Bajondillo	Early presence
		El Pirulejo	Early presence
		Navarrés	Not recorded
		Carihuela Cave	Early presence
		Cueva Perneras	Early presence
		Lago de Ajo	Not recorded
		Laguna de la Roya	Not recorded
		PRD-4	Not recorded
		Sanabria Marsh	Not recorded
		Quintanar de la Sierra	Not recorded
	Portugal	Charco da Candieira	Not recorded
		Lagoa Comprida 2	Not recorded
	Syria	Ghab	Early presence
	Israel	Hula Valley	Early presence
	Iran	Gomishan	Not recorded
		Lake Urmia	Early presence
		Lake Zeribar	Not recorded
	Iraq	Shanidar Cave	Early presence
	Georgia	Gagra	Not recorded
		Dzudzuana Cave	Early presence
		Dmanisi	Early presence
	Albania	Lake Maliq	Not recorded
		Lake Ohrid	Early presence
11.7 - 9 Ka BP			
	Turkey	Abant Gölü	Not recorded
		Yeniçaga Gölü	Discontinuous
		Gölhisar Gölü I	Not recorded
		Gölhisar Gölü II	Not recorded
		Kararmik Batakligi	Not recorded
		Pinarbasi	Not recorded
		Sögüt Gölü	Not recorded
		Ladik Gölü	Discontinuous
		Lake Van	Discontinuous
	Greece	Orestias	Not recorded
		Khimaditis III	Not recorded
		Edessa	Not recorded
		Ioannina I	Not recorded
		Ioannina II	Not recorded
		Halos	Not recorded
		Xinias	Not recorded
		Myrtoon basin	Discontinuous
	Bulgary	Beliya Kanton	Continuous
		Kupena I	Discontinuous
		Kupena II	Discontinuous
		Ribno I	Not recorded
		Black Sea South	Not recorded
		Black Sea Southwest	Not recorded
		Black Sea West	Not recorded
		Duranunlak II	Not recorded
		Duranunlak I	Not recorded
		Varna II	Discontinuous
		Vitosha	Not recorded
	Romania	Avrig I	Not recorded
		Mohos	Not recorded
		Luci	Not recorded
		Bisoca	Not recorded
		Semenic	Not recorded
		Steregoiu	Not recorded
	Hungary	Alsópáhok	Not recorded
		Balaton Centre	Not recorded
		Balaton Northeast	Not recorded
		Balaton Southwest	Not recorded
		Nagy-Mohos	Not recorded
		Pötréte	Not recorded
		Szigliget	Not recorded
	Slovakia	Bobrov	Not recorded
	Czech Republic	Vracov	Not recorded

	Palasiny	Not recorded
	Blato I	Not recorded
	Kozli	Not recorded
	Rezabinec	Not recorded
	Borkovicka blata	Not recorded
	Loucky	Not recorded
	Chrást	Not recorded
	Velka niva	Not recorded
	Strázenská slat	Not recorded
	Komoranské jezero	Not recorded
	Dolskym	Discontinuous
Poland	Bledowo Lake	Not recorded
	Lake Gosciaz	Not recorded
	Lake Skrzetuszewskie	Not recorded
	Swietokrzyskie Lake	Not recorded
	Lake Mikolajki	Not recorded
	Puscizna Rekowianska	Not recorded
Austria	Dortmunder Hütte	Not recorded
	Franz Senn-Hütte	Not recorded
	Egelsee	Not recorded
	Gerlos	Not recorded
	Giering	Not recorded
	Lindenmoos	Not recorded
	Mieminger See	Not recorded
	Moor Alpenrose	Not recorded
	Schwarzsee Reschenscheideck	Not recorded
	Schwemm	Not recorded
	Seefelder See	Discontinuous
	Wasenmoos beim Zellhof	Not recorded
	Grosses Überling Schattseit-Moor	Not recorded
	Dürrenecksee-Moor	Not recorded
	Fuchsschwanzmoos	Discontinuous
	Fuschlsee	Not recorded
Germany	Lüttersee	Not recorded
	Löddigsee	Not recorded
	Brentenlohe	Not recorded
	Glaswaldsee	Not recorded
	Herrenwiesser see	Not recorded
	Durchenbergried	Not recorded
	Feuenried	Discontinuous
	Hornstaad-Bodensee	Not recorded
	Huzenbacher See	Not recorded
	Steerenmoos	Not recorded
	Wilder See beim Ruhestein	Not recorded
	Wildseemoor bei Kaltenbronn	Not recorded
Switzerland	Etang de la Gruère	Not recorded
	Le Loclat	Not recorded
	Lobsigensee	Not recorded
	Montilier	Not recorded
	Aegelsee	Not recorded
	Amsoldingersee	Not recorded
	Bachalpsee	Not recorded
	Hinterburgseeli	Not recorded
	Linden	Not recorded
	Oberaar	Not recorded
	Rotsee	Not recorded
	Sägistalsee	Not recorded
	Süftenenegg	Not recorded
	Aletschwald	Not recorded
	Alpi di Robièi Val Bavona	Not recorded
	Bitsch-Naters	Not recorded
	Eggen ob Blatten	Not recorded
	Etang d'y Cor Montana	Not recorded
	Hopschensee	Not recorded
	Lac du Mont d'Orge Sion	Discontinuous
	Mittlere Hellelen	Not recorded
	Pillon Gsteig-Diablerets	Not recorded
	Simplon-Gampisch-Alter Spittel	Not recorded
	Wallbach Lenk	Not recorded
	Mont Roux	Not recorded
	Gamperfin	Not recorded
	Creux de Croue	Not recorded
	Motta Naluns	Not recorded
	Praz Rodet	Not recorded
Italy	Bondone	Not recorded
	Dura-Moor	Not recorded

	Malschötscher Hotter	Not recorded
	Rinderplatz	Not recorded
	Schwarzsee	Not recorded
	Sommersüss	Not recorded
	Grunsee	Not recorded
	Dossaccio Bormio	Not recorded
	Lac de Villa	Not recorded
	Lac du Verney-Dessus	Not recorded
	Torveraz	Not recorded
	Tourbière de Pilaz	Continuous
	Tourbière de Santa Anna	Not recorded
	Laghi dell'Orgials	Not recorded
	Lago Piccolo di Avigliana	Not recorded
	Ortasee	Not recorded
	Refugio Mondovi	Not recorded
	Selle di Carnino	Not recorded
	Torbiera del Biecai	Not recorded
	Lago della Costa	Not recorded
	Lago Padule	Not recorded
	Lago dell'Accesa	Not recorded
	Colfiorito	Not recorded
	Pavullo	Not recorded
	Lago di Martignano	Discontinuous
	Lago di Vico	Not recorded
	Lago Albano	Not recorded
	Lago di Nemi	Discontinuous
	Salerno Bay	Not recorded
	Lago Grande di Monticchio LGM	Not recorded
France	Aronde	Not recorded
	Auneau	Not recorded
	La Vie	Not recorded
	Le Fourneau	Not recorded
	Moulin de Thévalles	Not recorded
	Pezou	Not recorded
	Saint-Ursin	Not recorded
	Altenweiher	Not recorded
	Bellefontaine	Not recorded
	Hières sur Amby	Not recorded
	Loras	Not recorded
	Etang du Lautrey	Not recorded
	Le Grand Lemps	Not recorded
	Tourbières des Granges des Chavants	Discontinuous
	Ampoix	Not recorded
	Champ Gazon	Not recorded
	Etang de Cheylade	Not recorded
	La Taphanel	Not recorded
	Lac du Bouchet	Not recorded
	Lac du Mont de Belier	Not recorded
	Le Miroir	Not recorded
	Marais du Grang Chaumet	Discontinuous
	Moulin de Prugnolas	Not recorded
	Nataloup	Not recorded
	Peyre peat-bog	Not recorded
	Saint-Benoit-sur-Loire	Not recorded
	Tourbière de Longeyroux	Not recorded
	Tourbière de Roussy	Discontinuous
	Tourbière des Dauges	Not recorded
	Bois-Jésus	Not recorded
	Cinq-Mars-la-Pile	Not recorded
	Cordemais	Not recorded
	Jaunay	Not recorded
	La Grande Brousse	Not recorded
	Marais de Champtocé	Not recorded
	Marais de Munet	Not recorded
	Marais de la Poupinière	Not recorded
	Tourbière de Parcay-sur-Vienne	Discontinuous
	Clapeyret	Not recorded
	Col des Lauzes	Not recorded
	Correo	Not recorded
	Grand Ratz le Pellet	Not recorded
	Lac Long Inférieur	Not recorded
	Lac des Boites	Not recorded
	Lake Racou	Not recorded
	Lignin Lake	Not recorded
	Peuil Peat Bog	Not recorded
	Pré Rond	Not recorded

		Sabbion	Not recorded
		Saint Hilaire du Rosier	Not recorded
		Saint Julien de Ratz	Not recorded
		Tourbière de Mont Sec	Discontinuous
	Spain	Cala Galdana	Not recorded
		Bajondillo	Not recorded
		El Pirulejo	Not recorded
		Navarrés	Not recorded
		Lago de Ajo	Not recorded
		Laguna de la Roya	Not recorded
		PRD-4	Discontinuous
		Saldropo	Not recorded
		Sanabria Marsh	Not recorded
		Quintanar de la Sierra	Not recorded
	Portugal	Charco da Candieira	Not recorded
		Lagoa Comprida 2	Not recorded
	Syria	Ghab	Discontinuous
	Israel	Hula Valley (Israel)	Not recorded
	Iran	Gomishan	Not recorded
		Lake Urmia	Not recorded
		Lake Zeribar	Not recorded
	Georgia	Gagra (Georgia)	Not recorded
	Albania	Lake Maliq (Albania)	Not recorded
9 - 8 Ka BP			
	Turkey	Abant Gölü	Not recorded
		Yeniçaga Gölü	Not recorded
		Gölhisar Gölü I	Not recorded
		Gölhisar Gölü II	Not recorded
		Kararmik Batakligi	Not recorded
		Pinarbasi	Not recorded
		Sögüt Gölü	Not recorded
		Ladik Gölü	Not recorded
		Lake Van	Discontinuous
	Greece	Orestias	Discontinuous
		Khimaditis III	Not recorded
		Edessa	Not recorded
		Ioannina I	Not recorded
		Ioannina II	Not recorded
		Halos	Not recorded
		Xinias	Discontinuous
		Myrtoon basin	Discontinuous
	Bulgary	Beliya Kanton	Continuous
		Kupena I	Not recorded
		Kupena II	Not recorded
		Ribno I	Discontinuous
		Black Sea South	Not recorded
		Black Sea Southwest	Not recorded
		Black Sea West	Not recorded
		Duranunlak II	Not recorded
		Duranunlak I	Discontinuous
		Shabla-Ezeretz	Discontinuous
		Varna II	Not recorded
		Tchokljovo Marsh	Not recorded
		Vitosha	Not recorded
	Romania	Avrig I	Not recorded
		Mohos	Not recorded
		Luci	Not recorded
		Bisoca	Not recorded
		Semenic	Not recorded
		Steregoiu	Not recorded
	Hungary	Alsópáhok	Not recorded
		Balaton Centre	Not recorded
		Balaton Northeast	Not recorded
		Balaton Southwest	Not recorded
		Nagy-Mohos	Not recorded
		Pötréte	Not recorded
		Szigliget	Not recorded
	Slovakia	Bobrov	Not recorded
		Zlatnicka Dolina	Not recorded
	Czech Republic	Dvur Ansov	Not recorded
		Vracov	Not recorded
		Palasiny	Not recorded
		Blato I	Not recorded
		Kozli	Not recorded
		Rezabinec	Not recorded
		Borkovicka blata	Not recorded

	Loucky	Not recorded
	Chrást	Not recorded
	Velka niva	Not recorded
	Strázenská slat	Not recorded
	Komoranské jezero	Not recorded
	Dolskym	Not recorded
Ukraina	Dovjok Swamp	Not recorded
	Maly Podleski	Not recorded
Poland	Bledowo Lake	Not recorded
	Lake Gosciaz	Not recorded
	Lake Skrzetuszewskie	Not recorded
	Swietokrzyskie Lake	Not recorded
	Godziszewskie Lake	Not recorded
	Lake Mikolajki	Not recorded
	Puscizna Rekowianska	Not recorded
Austria	Dortmunder Hütte	Not recorded
	Franz Senn-Hütte	Not recorded
	Egelsee	Not recorded
	Gerlos	Not recorded
	Giering	Not recorded
	Grünau Moor	Not recorded
	Lindenmoos	Not recorded
	Mieminger See	Not recorded
	Moor Alpenrose	Not recorded
	Schwarzsee Reschenscheideck	Not recorded
	Schwemm	Not recorded
	Seefelder See	Not recorded
	Zirbenwaldmoor	Not recorded
	Wasenmoos beim Zellhof	Not recorded
	Grosses Überling Schattseit-Moor	Not recorded
	Dürrenecksee-Moor	Not recorded
	Fuchsschwanzmoos	Discontinuous
	Fuschlsee	Not recorded
Germany	Bruchberg	Not recorded
	Lüttersee	Not recorded
	Löddigsee	Not recorded
	Wachel 3	Not recorded
	Brentenlohe	Not recorded
	Glaswaldsee	Not recorded
	Herrenwiesser see	Not recorded
	Durchenbergried	Not recorded
	Feuenried	Not recorded
	Hornstaad-Bodensee	Not recorded
	Huzenbacher See	Not recorded
	Steerenmoos	Not recorded
	Wilder See beim Ruhestein	Not recorded
	Wildseemoor bei Kaltenbronn	Not recorded
Switzerland	Etang de la Gruère	Not recorded
	Lobsigensee	Not recorded
	Montilier	Not recorded
	Aegelsee	Not recorded
	Amsoldingersee	Not recorded
	Bachalpsee	Not recorded
	Hinterburgseeli	Not recorded
	Linden	Not recorded
	Oberaar	Not recorded
	Rotsee	Not recorded
	Sägistalsee	Not recorded
	Süftenenegg	Not recorded
	Aletschwald	Not recorded
	Alpi di Robièi Val Bavona	Not recorded
	Bitsch-Naters	Not recorded
	Eggen ob Blatten	Not recorded
	Etang d'y Cor Montana	Not recorded
	Hopschensee	Not recorded
	Lac du Mont d'Orge Sion	Not recorded
	Mittlere Hellelen	Not recorded
	Pillon Gsteig-Diablerets	Not recorded
	Simplon-Gampisch-Alter Spittel	Not recorded
	Wallbach Lenk	Not recorded
	Mont Roux	Not recorded
	Gamperfin	Not recorded
	Creux de Croue	Not recorded
	Motta Naluns	Not recorded
	Praz Rodet	Not recorded
Italy	Bondone	Not recorded

	Dura-Moor	Not recorded
	Malschötscher Hotter	Not recorded
	Rinderplatz	Not recorded
	Schwarzsee	Not recorded
	Sommersüss	Not recorded
	Grunsee	Not recorded
	Dossaccio Bormio	Not recorded
	Lac de Villa	Not recorded
	Lac du Verney-Dessus	Not recorded
	Torveraz	Not recorded
	Tourbière de Pilaz	Continuous
	Tourbière de Santa Anna	Not recorded
	Laghi dell'Orgials	Not recorded
	Lago Piccolo di Avigliana	Not recorded
	Ortasee	Not recorded
	Refugio Mondovi	Not recorded
	Selle di Carnino	Not recorded
	Torbiera del Biecai	Not recorded
	Lago della Costa	Not recorded
	Lago Padule	Not recorded
	Lago dell'Accesa	Discontinuous
	Colfiorito	Not recorded
	Lago Pratignano	Not recorded
	Pavullo	Not recorded
	Lago di Martignano	Not recorded
	Lago di Vico	Not recorded
	Lago Albano	Discontinuous
	Lago di Nemi	Discontinuous
	Salerno Bay	Not recorded
	Lago Grande di Monticchio LGM	Not recorded
France	Aronde	Not recorded
	Auneau	Not recorded
	Baie de Seine estuary	Not recorded
	La Vie	Not recorded
	Le Fourneau	Not recorded
	Moulin de Thévalles	Not recorded
	Pezou	Not recorded
	Saint-Ursin	Not recorded
	Serrent	Not recorded
	Altenweiher	Not recorded
	Bellefontaine	Discontinuous
	Hières sur Amby	Not recorded
	Loras	Not recorded
	Etang du Lautrey	Not recorded
	Le Grand Lemps	Not recorded
	Tourbières des Granges des Chavants	Not recorded
	Ampoix	Not recorded
	Champ Gazon	Not recorded
	Etang de Cheylade	Not recorded
	La Taphanel	Not recorded
	Lac du Bouchet	Not recorded
	Lac du Mont de Belier	Not recorded
	Le Miroir	Not recorded
	Marais du Grang Chaumet	Not recorded
	Moulin de Prugnolas	Not recorded
	Nataloup	Not recorded
	Peyre peat-bog	Not recorded
	Saint-Benoit-sur-Loire	Not recorded
	Tourbière de Longeyroux	Not recorded
	Tourbière de Roussy	Not recorded
	Tourbière des Dauges	Not recorded
	Tourbière de Chabannes	Not recorded
	Ancenis	Not recorded
	Bois-Jésus	Not recorded
	Caves d'Amont	Discontinuous
	Cinq-Mars-la-Pile	Discontinuous
	Cordemais	Not recorded
	Ecours	Not recorded
	Jaunay	Discontinuous
	La Grande Brousse	Not recorded
	Le Gesvres	Not recorded
	Marais de Champtocé	Not recorded
	Marais de Munet	Not recorded
	Marais de la Poupinière	Not recorded
	Petit Rocher	Not recorded
	Tourbière de Parcay-sur-Vienne	Not recorded

		Clapeyret	Not recorded
		Col des Lauzes	Not recorded
		Correo	Not recorded
		Embouchac	Not recorded
		Etang d'Ouveillan	Not recorded
		Grand Ratz le Pellet	Not recorded
		Lac Long Inférieur	Not recorded
		Lac des Boites	Not recorded
		Lake Racou	Not recorded
		Lignin Lake	Not recorded
		Peuil Peat Bog	Not recorded
		Pré Rond	Not recorded
		Sabbion	Not recorded
		Saint Hilaire du Rosier	Not recorded
		Saint Julien de Ratz	Not recorded
		Tourbière de Mont Sec	Not recorded
	Spain	Algendar	Not recorded
		Cala Galdana	Not recorded
		Antas	Discontinuous
		Bajondillo	Not recorded
		El Pirulejo	Not recorded
		Navarrés	Not recorded
		Lago de Ajo	Not recorded
		Laguna de la Roya	Not recorded
		PRD-4	Discontinuous
		Saldropo	Not recorded
		Sanabria Marsh	Discontinuous
		Quintanar de la Sierra	Not recorded
	Portugal	Charco da Candieira	Not recorded
		Lagoa Comprida 2	Not recorded
	Syria	Ghab	Not recorded
	Israel	Hula Valley	Not recorded
	Iran	Gomishan	Not recorded
		Lake Urmia	Discontinuous
		Lake Zeribar	Not recorded
	Georgia	Gagra	Not recorded
		Lagodekhi	Not recorded
	Albania	Lake Maliq	Not recorded
8 - 7 Ka BP			
	Turkey	Abant Gölü	Not recorded
		Yeniçaga Gölü	Not recorded
		Gölhisar Gölü I	Not recorded
		Gölhisar Gölü II	Not recorded
		Kararmik Batakligi	Not recorded
		Pinarbasi	Not recorded
		Sögüt Gölü	Not recorded
		Ladik Gölü	Not recorded
		Lake Van	Not recorded
	Greece	Orestias (Greece)	In expansion
		Khimaditis III	Not recorded
		Edessa	Not recorded
		Trikhonis	Not recorded
		Ioannina I	Not recorded
		Ioannina II	Discontinuous
		Halos	Not recorded
		Xinias	Not recorded
		Myrtoon Basin	Discontinuous
	Bulgary	Beliya Kanton	Continuous
		Kupena I	In expansion
		Kupena II	Not recorded
		Ribno I	Discontinuous
		Popovo Ezero	Not recorded
		Black Sea South	Not recorded
		Black Sea Southwest	Not recorded
		Black Sea West	Discontinuous
		Duranunlak II	Not recorded
		Duranunlak I	Discontinuous
		Shabla-Ezeretz	Discontinuous
		Varna I	Not recorded
		Varna II	Not recorded
		Tchokljovo Marsh	Not recorded
		Vitosha	Not recorded
	Romania	Avrig I	Not recorded
		Mohos	Not recorded
		Luci	Not recorded
		Bisoca	Not recorded

	Calinease	Not recorded
	Semenic	Not recorded
	Steregoiu	Not recorded
Hungary	Alsópáhok	Not recorded
	Balaton Centre	Not recorded
	Balaton Northeast	Not recorded
	Balaton Southwest	Not recorded
	Nagy-Mohos	Not recorded
	Pötréte	Not recorded
	Szigliget	Not recorded
Slovakia	Bobrov	Not recorded
	Zlatnicka Dolina	Not recorded
Czech Republic	Dvur Ansov	Not recorded
	Vracov	Not recorded
	Palasiny	Not recorded
	Blato I	Not recorded
	Kozli	Not recorded
	Rezabinec	Not recorded
	Borkovicka blata	Not recorded
	Branna	Not recorded
	Loucky	Not recorded
	Chrást	Not recorded
	Velka niva	Not recorded
	Strázenská slat	Not recorded
	Komoranské jezero	Not recorded
	Dolskym	Not recorded
Ukraina	Dovjok Swamp	Not recorded
	Maly Podleski	Not recorded
Croatia	Bokanjacko	Not recorded
Poland	Bledowo Lake	Not recorded
	Giecz	Not recorded
	Lake Gosciaz	Discontinuous
	Lake Skrzetuszewskie	Not recorded
	Swietokrzyskie Lake	Not recorded
	Godziszewskie Lake	Not recorded
	Lake Mikolajki	Not recorded
	Puscizna Rekowianska	Not recorded
Austria	Dortmunder Hütte	Not recorded
	Franz Senn-Hütte	Not recorded
	Egelsee	Not recorded
	Gerlos	Not recorded
	Giering	Not recorded
	Grünau Moor	Not recorded
	Lindenmoos	Not recorded
	Mieminger See	Not recorded
	Moor Alpenrose	Not recorded
	Schwarzsee Reschenscheideck	Not recorded
	Schwemm	Not recorded
	Seefelder See	Not recorded
	Zirbenwaldmoor	Not recorded
	Wasenmoos beim Zellhof	Not recorded
	Grosses Überling Schattseit-Moor	Not recorded
	Dürrenecksee-Moor	Not recorded
	Fuchsschwanzmoos	Discontinuous
	Fuschlsee	Not recorded
Germany	Ahlequellmoor	Not recorded
	Bruchberg	Not recorded
	Lüttersee	Not recorded
	Löddigsee	Not recorded
	Wachel 3	Not recorded
	Brentenlohe	Not recorded
	Glaswaldsee	Not recorded
	Herrenwiesser see	Not recorded
	Durchenbergried	Not recorded
	Feuenried	Discontinuous
	Hornstaad-Bodensee	Not recorded
	Huzenbacher See	Not recorded
	Mindelsee	Not recorded
	Steerenmoos	Not recorded
	Wilder See beim Ruhestein	Not recorded
	Wildseemoor bei Kaltenbronn	Not recorded
Switzerland	Etang de la Gruère	Not recorded
	Le Loclat	Not recorded
	Lobsigensee	Not recorded
	Montilier	Not recorded
	Aegelsee	Not recorded

	Amsoldingersee	Not recorded
	Bachalpsee	Not recorded
	Hinterburgseeli	Not recorded
	Hängstli	Not recorded
	Linden	Not recorded
	Oberaar	Not recorded
	Rotsee	Not recorded
	Schwarzsee FR	Not recorded
	Sägistalsee	Not recorded
	Süftenenegg	Not recorded
	Aletschwald	Not recorded
	Alp Lüsga Belalp 1	Not recorded
	Alpi di Robièi Val Bavona	Not recorded
	Alpi di Robièi Val Bavona Bodenprofil	Not recorded
	Bitsch-Naters	Not recorded
	Eggen ob Blatten	Discontinuous
	Etang d'y Cor Montana	Not recorded
	Gondo Alpjen	Not recorded
	Grächen See	Not recorded
	Hopschensee	Not recorded
	Lac du Mont d'Orge Sion	Discontinuous
	Mittlere Hellelen	Not recorded
	Pillon Gsteig-Diablerets	Not recorded
	Simplon-Gampisch-Alter Spittel	Not recorded
	Wallbach Lenk	Not recorded
	Mont Roux	Not recorded
	Gamperfin	Not recorded
	Creux de Croue	Not recorded
	Motta Naluns	Not recorded
	Praz Rodet	Not recorded
Italy	Bondone	Not recorded
	Dura-Moor	Not recorded
	Malschötscher Hotter	Not recorded
	Rinderplatz	Not recorded
	Schwarzsee	Not recorded
	Sommersüss	Not recorded
	Grunsee	Discontinuous
	Dossaccio Bormio	Not recorded
	Lac de Villa	Not recorded
	Lac du Verney-Dessus	Not recorded
	Torveraz	Not recorded
	Tourbière de Pilaz	Continuous
	Tourbière de Santa Anna	Not recorded
	Laghi dell'Orgials	Not recorded
	Lago Piccolo di Avigliana	Not recorded
	Ortasee	Discontinuous
	Refugio Mondovi	Not recorded
	Selle di Carnino	Not recorded
	Torbiera del Biecai	Not recorded
	Lago della Costa	Not recorded
	Lago Padule	Not recorded
	Lago dell'Accesa	Not recorded
	Colfiorito	Not recorded
	Lago Pratignano	Not recorded
	Pavullo	Not recorded
	Lago di Martignano	Discontinuous
	Lago di Vico	Not recorded
	Lago Albano	Discontinuous
	Lago di Nemi	Discontinuous
	Central Adriatic Sea	Not recorded
	Salerno Bay	Not recorded
	Lago Grande di Monticchio LGM	Not recorded
France	Aronde	Not recorded
	Auneau	Not recorded
	Baie de Seine estuary	Not recorded
	La Vie	Not recorded
	Le Fourneau	Not recorded
	Moulin de Thévalles	Not recorded
	Pezou	Not recorded
	Saint-Ursin	Not recorded
	Serrent	Not recorded
	Altenweiher	Not recorded
	Bellefontaine	Not recorded
	Hières sur Amby	Not recorded
	Loras	Not recorded
	Lutinière	Not recorded

	Etang du Lautrey	Discontinuous
	Le Grand Lemps	Not recorded
	Tourbières des Granges des Chavants	Not recorded
	Ampoix	Not recorded
	Champ Gazon	Not recorded
	Etang de Cheylade	Not recorded
	La Taphanel	Not recorded
	Lac du Bouchet	Not recorded
	Lac du Mont de Belier	Not recorded
	Le Grand Montarnu	Not recorded
	Le Miroir	Not recorded
	Marais du Grang Chaumet	Not recorded
	Moulin de Prugnolas	Not recorded
	Nataloup	Not recorded
	Peyre peat-bog	Not recorded
	Saint-Benoit-sur-Loire	Not recorded
	Tourbière de Longeyroux	Not recorded
	Tourbière de Roussy	Not recorded
	Tourbière des Dauges	Not recorded
	Tourbière de Chabannes	Not recorded
	Ancenis	Not recorded
	Basse-Ville	Not recorded
	Bois-Jésus	Not recorded
	Caves d'Amont	Not recorded
	Cinq-Mars-la-Pile	Not recorded
	Cordemais	Not recorded
	Ecours	Discontinuous
	Jaunay	Discontinuous
	La Grande Brousse	Not recorded
	La Grange	Not recorded
	Le Gesvres	Not recorded
	Marais de Champtocé	Not recorded
	Marais de Mazerolles	Not recorded
	Marais de Munet	Not recorded
	Marais de la Poupinière	Not recorded
	Oudon	Not recorded
	Petit Marais	Not recorded
	Petit Rocher	Not recorded
	Tourbière de Nay	Not recorded
	Tourbière de Parcay-sur-Vienne	Not recorded
	Vertonne	Not recorded
	Clapeyret	Not recorded
	Col Luitel	Not recorded
	Col des Lauzes	Not recorded
	Correo	Not recorded
	Embouchac	Not recorded
	Etang d'Ouveillan	Not recorded
	Grand Ratz le Pellet	Not recorded
	Lac Long Inférieur	Not recorded
	Lac des Boites	Not recorded
	Lac du Lauzon	Not recorded
	Lake Racou	Not recorded
	Lignin Lake	In expansion
	Peuil Peat Bog	Not recorded
	Pré Rond	Not recorded
	Sabbion	Not recorded
	Saint Hilaire du Rosier	Not recorded
	Saint Julien de Ratz	Not recorded
	Tourbière de Gatimort	Not recorded
	Tourbière de Mont Sec	Not recorded
Spain	Albufera Alcudia	Not recorded
	Algendar	Not recorded
	Cala Galdana	Not recorded
	Cala'n Porter	Not recorded
	Antas	Not recorded
	Bajondillo	Not recorded
	Navarrés	Not recorded
	Lago de Ajo	Not recorded
	Laguna de la Roya	Not recorded
	PRD-4	Discontinuous
	Puerto de Los Tornos	Not recorded
	Saldropo	Not recorded
	Sanabria Marsh	Not recorded
	Quintanar de la Sierra	Not recorded
Portugal	Charco da Candieira	Not recorded
	Lagoa Comprida 2	Discontinuous

		Lagoa Travessa I	Not recorded
	Syria	Ghab (Syria)	Not recorded
	Israel	Hula Valley	Not recorded
	Iran	Gomishan	Not recorded
		Lake Urmia	Not recorded
		Lake Zeribar	Not recorded
	Georgia	Gagra	Not recorded
		Lagodekhi	Not recorded
	Albania	Lake Maliq	Not recorded
7 - 6 Ka BP			
	Turkey	Abant Gölü	Not recorded
		Yeniçaga Gölü	Discontinuous
		Beysehir Gölü I	Not recorded
		Gölhisar Gölü I	Not recorded
		Gölhisar Gölü II	Not recorded
		Kararmik Batakligi	Discontinuous
		Ova Gölü	Not recorded
		Pinarbasi	Not recorded
		Sögüt Gölü	Not recorded
		Ladik Gölü	Discontinuous
		Lake Van	Not recorded
	Greece	Orestias	Continuous
		Khimaditis III	Not recorded
		Edessa	Not recorded
		Lerna	Discontinuous
		Trikhonis	Not recorded
		Ioannina I	Not recorded
		Ioannina II	Not recorded
		Halos	Not recorded
		Xinias	Discontinuous
		Pertouli	Not recorded
		Myrtoon Basin	Discontinuous
	Bulgary	Beliya Kanton	Continuous
		Kupena I	Continuous
		Kupena II	Not recorded
		Ribno I	Discontinuous
		Popovo Ezero	Not recorded
		Sozopol	Continuous
		Arkutino I	Not recorded
		Arkutino II	Discontinuous
		Black Sea South	Not recorded
		Black Sea Southwest	Not recorded
		Black Sea West	In expansion
		Duranunlak II	Not recorded
		Duranunlak I	Discontinuous
		Shabla-Ezeretz	Discontinuous
		Varna I	Not recorded
		Varna II	Discontinuous
		Mire Garvan	Not recorded
		Maleshevska	Continuous
		Tchokljovo Marsh	Discontinuous
		Vitosha	Not recorded
	Romania	Avrig I	Not recorded
		Mohos	Not recorded
		Luci	Not recorded
		Bisoca	Not recorded
		Calinease	Not recorded
		Semenic	Not recorded
		Steregoiu	Not recorded
	Hungary	Alsópáhok	Not recorded
		Balaton Centre	Not recorded
		Balaton Northeast	Not recorded
		Balaton Southwest	Not recorded
		Nagy-Mohos	Not recorded
		Pötréte	Not recorded
		Szigliget	Not recorded
	Slovakia	Bobrov	Not recorded
		Zlatnicka Dolina	Not recorded
	Czech Republic	Dvur Ansov	Not recorded
		Vracov	Not recorded
		Palasiny	Not recorded
		Blato I	Discontinuous
		Kozli	Not recorded
		Rezabinec	Not recorded
		Borkovicka blata	Discontinuous
		Branna	Not recorded

	Loucky	Not recorded
	Chrást	Discontinuous
	Velka niva	Not recorded
	Mrtv8 luh	Not recorded
	Strázenská slat	Not recorded
	Komoranské jezero	Not recorded
	Dolskym	Not recorded
Ukraina	Dovjok Swamp	Not recorded
	Maly Podleski	Discontinuous
Croatia	Bokanjacko	Not recorded
Poland	Bledowo Lake	Not recorded
	Giecz	Not recorded
	Lake Gosciaz	Not recorded
	Lake Skrzetuszewskie	Not recorded
	Slawsko	Not recorded
	Swietokrzyskie Lake	Not recorded
	Godziszewskie Lake	Not recorded
	Lake Mikolajki	Not recorded
	Puscizna Rekowianska	Not recorded
Austria	Buntes Moor	Not recorded
	Dortmunder Hütte	Not recorded
	Franz Senn-Hütte	Not recorded
	Egelsee	Not recorded
	Gerlos	Not recorded
	Giering	Not recorded
	Grünau Moor	Not recorded
	Lindenmoos	Not recorded
	Mieminger See	Not recorded
	Moor Alpenrose	Not recorded
	Rotmoos Obergurgl	Not recorded
	Schwarzsee Reschenscheideck	Not recorded
	Schwemm	Not recorded
	Seefelder See	Not recorded
	Zirbenwaldmoor	Not recorded
	Wasenmoos beim Zellhof	Not recorded
	Grosses Überling Schattseit-Moor	Not recorded
	Dürrenecksee-Moor	Not recorded
	Fuchsschwanzmoos	Discontinuous
	Fuschlsee	Not recorded
Germany	Ahlequellmoor	Not recorded
	Bruchberg	Not recorded
	Lüttersee	Not recorded
	Silberhohl	Not recorded
	Löddigsee	Not recorded
	Wachel 3	Not recorded
	Brentenlohe	Not recorded
	Glaswaldsee	Not recorded
	Herrenwiesser see	Not recorded
	Durchenbergried	Not recorded
	Feuenried	Not recorded
	Hornstaad-Bodensee	Not recorded
	Huzenbacher See	Not recorded
	Mindelsee	Not recorded
	Steerenmoos	Not recorded
	Wilder See beim Ruhestein	Not recorded
	Wildseemoor bei Kaltenbronn	Not recorded
Switzerland	Etang de la Gruère	Not recorded
	Le Loclat	Not recorded
	Lobsigensee	Not recorded
	Montilier	Not recorded
	Aegelsee	Not recorded
	Amsoldingersee	Not recorded
	Bachalpsee	Not recorded
	Hinterburgseeli	Not recorded
	Hängstli	Not recorded
	Linden	Not recorded
	Oberaar	Not recorded
	Rotsee	Not recorded
	Schwarzsee FR	Not recorded
	Sägistalsee	Not recorded
	Süftenenegg	Not recorded
	Aletschwald	Not recorded
	Alp Lüsga Belalp 1	Not recorded
	Alpi di Robièi Val Bavona	Not recorded
	Alpi di Robièi Val Bavona Bodenprofil	Not recorded
	Bitsch-Naters	Not recorded

	Eggen ob Blatten	Not recorded
	Etang d'y Cor Montana	Not recorded
	Etang de Luissel Bex	Discontinuous
	Gondo Alpjen	Not recorded
	Greicheralp Riederalp	Not recorded
	Grächen See	Not recorded
	Hopschensee	Not recorded
	Lac du Mont d'Orge Sion	Not recorded
	Mittlere Hellelen	Not recorded
	Pillon Gsteig-Diablerets	Not recorded
	Simplon-Gampisch-Alter Spittel	Not recorded
	Wallbach Lenk	Not recorded
	Mont Roux	Not recorded
	Gamperfin	Not recorded
	Creux de Croue	Not recorded
	Motta Naluns	Not recorded
	Praz Rodet	Not recorded
Italy	Bondone	Not recorded
	Dura-Moor	Not recorded
	Malschötscher Hotter	Not recorded
	Rinderplatz	Not recorded
	Schwarzsee	Not recorded
	Sommersüss	Not recorded
	Grunsee	Not recorded
	Dossaccio Bormio	Not recorded
	Lac de Villa	Not recorded
	Lac du Verney-Dessus	Not recorded
	Torveraz	Not recorded
	Tourbière de Pilaz	Continuous
	Tourbière de Santa Anna	Not recorded
	Laghi dell'Orgials	Not recorded
	Lago Piccolo di Avigliana	Not recorded
	Ortasee	Not recorded
	Refugio Mondovi	Not recorded
	Selle di Carnino	Not recorded
	Torbiera del Biecai	Not recorded
	Lago della Costa	Continuous
	Lago Padule	Not recorded
	Lago dell'Accesa	Discontinuous
	Colfiorito	Not recorded
	Lago Pratignano	Not recorded
	Pavullo	Not recorded
	Lago di Martignano	In expansion
	Lago di Vico	Not recorded
	Lago Albano	Discontinuous
	Lago di Nemi	Discontinuous
	Central Adriatic Sea	Discontinuous
	Salerno Bay	Not recorded
	Lago Grande di Monticchio LGM	Not recorded
France	Aronde	Not recorded
	Auneau	Not recorded
	Baie de Seine estuary	Not recorded
	La Vie	Not recorded
	Le Fourneau	Not recorded
	Marais de Lisle	Not recorded
	Moulin de Thévalles	Not recorded
	Pezou	Not recorded
	Saint-Ursin	Not recorded
	Serrent	Not recorded
	Altenweiher	Not recorded
	Hières sur Amby	Not recorded
	Loras	Not recorded
	Lutinière	Not recorded
	Moselotte	Not recorded
	Le Grand Lemps	Not recorded
	Tourbières des Granges des Chavants	Not recorded
	Ampoix	Not recorded
	Champ Gazon	Not recorded
	Etang de Cheylade	Not recorded
	La Taphanel	Not recorded
	Lac du Bouchet	Not recorded
	Lac du Mont de Belier	Not recorded
	Le Grand Montarnu	Not recorded
	Le Jolan	Not recorded
	Le Miroir	Not recorded
	Marais du Grang Chaumet	Not recorded

	Moulin de Prugnolas	Not recorded
	Nataloup	Not recorded
	Peyre peat-bog	Not recorded
	Saint-Benoit-sur-Loire	Not recorded
	Tourbière de Longeyroux	Not recorded
	Tourbière de Roussy	Not recorded
	Tourbière des Dauges	Not recorded
	La Baforière	Not recorded
	Tourbière de Chabannes	Not recorded
	Ancenis	Not recorded
	Basse-Ville	Not recorded
	Bois-Jésus	Not recorded
	Carquefou	Not recorded
	Caves d'Amont	Discontinuous
	Cinq-Mars-la-Pile	Discontinuous
	Cordemais	Not recorded
	Ecours	Discontinuous
	Jaunay	Discontinuous
	La Caudelais	Discontinuous
	La Grande Brousse	Not recorded
	La Grange	Not recorded
	Le Gesvres	Discontinuous
	Le Marais de la Perge	Not recorded
	Marais de Champtocé	Not recorded
	Marais de Mazerolles	Not recorded
	Marais de Munet	Not recorded
	Marais de la Poupinière	Not recorded
	Oudon	Not recorded
	Pas du Gu	Not recorded
	Petit Marais	Not recorded
	Petit Rocher	Discontinuous
	Riabelais	Not recorded
	Saint Viaud Contin	Not recorded
	Tourbière de Nay	Not recorded
	Tourbière de Parcay-sur-Vienne	Not recorded
	Vertonne	Not recorded
	Clapeyret	Not recorded
	Col Luitel	Not recorded
	Col des Lauzes	Discontinuous
	Correo	Not recorded
	Embouchac	Discontinuous
	Etang d'Ouveillan	Not recorded
	Grand Ratz le Pellet	Not recorded
	Lac Long Inférieur	Not recorded
	Lac des Boites	Not recorded
	Lac du Lauzon	Discontinuous
	Lake Racou	Not recorded
	Lignin Lake	Continuous
	Pelléautier	Not recorded
	Peuil Peat Bog	Discontinuous
	Pré Rond	Not recorded
	Sabbion	Not recorded
	Saint Hilaire du Rosier	Not recorded
	Saint Julien de Ratz	Not recorded
	Saint Sixte	Not recorded
	Tourbière de Gatimort	Not recorded
	Tourbière de Mont Sec	Not recorded
	Tourbière de la Lande	Not recorded
	Tourbière du Peschio	Not recorded
Spain	Albufera Alcudia	Not recorded
	Algendar	Discontinuous
	Cala Galdana	Not recorded
	Cala'n Porter	Not recorded
	Antas	Not recorded
	Bajondillo	Not recorded
	Laguna Salada Chiprana	Not recorded
	Navarrés	Not recorded
	Atxuri	Not recorded
	Cueto de la Avellanosa	Not recorded
	Lago de Ajo	Not recorded
	Laguna de la Roya	Not recorded
	PRD-4	Not recorded
	Puerto de Los Tornos	Not recorded
	Saldropo	Not recorded
	Sanabria Marsh	Not recorded
	Quintanar de la Sierra	Not recorded

	Portugal	Charco da Candieira	Not recorded
		Lagoa Comprida 2	Not recorded
		Lagoa Travessa I	Discontinuous
	Syria	Bouara	Not recorded
		Ghab	Not recorded
	Israel	Dead Sea-Ze’elim	Not recorded
		Birkat Ram	Discontinuous
		Hula Valley	Not recorded
	Iran	Gomishan	Not recorded
		Lake Urmia	Not recorded
		Lake Zeribar	Not recorded
	Georgia	Gagra	Not recorded
		Lagodekhi	Not recorded
		Akhali Ateni	Discontinuous
	Albania	Lake Maliq	Not recorded
6 - 5 Ka BP			
	Turkey	Marmara Sea	Not recorded
		Abant Gölü	Continuous
		Yeniçaga Gölü	Not recorded
		Beysehir Gölü I	Not recorded
		Gölhisar Gölü I	Not recorded
		Gölhisar Gölü II	Not recorded
		Hoyran Gölü	Not recorded
		Kararmik Batakligi	Not recorded
		Köycegiz Gölü	Not recorded
		Ova Gölü	Not recorded
		Pinarbasi	Not recorded
		Sögüt Gölü	Not recorded
		Ladik Gölü	In expansion
		Lake Van	Discontinuous
	Greece	Kastoria	Not recorded
		Orestias	Continuous
		Khimaditis III	Not recorded
		Vegoritis	Not recorded
		Edessa	Not recorded
		Litochoro	Not recorded
		Tenaghi Philippon	Not recorded
		Lerna	Discontinuous
		Trikhonis	Not recorded
		Ioannina I	Discontinuous
		Ioannina II	Not recorded
		Halos	Not recorded
		Xinias	Discontinuous
		Voulkaria	Not recorded
		Pertouli	Not recorded
		Myrtoon Basin	Continuous
	Bulgary	Beliya Kanton	Continuous
		Kupena II	Not recorded
		Ribno I	Discontinuous
		Popovo Ezero	Not recorded
		Sozopol	Continuous
		Arkutino I	Discontinuous
		Arkutino II	Discontinuous
		Black Sea South	Not recorded
		Black Sea Southwest	Not recorded
		Black Sea West	Continuous
		Duranunlak II	Discontinuous
		Duranunlak I	Not recorded
		Shabla-Ezeretz	Discontinuous
		Varna I	Discontinuous
		Varna II	Discontinuous
		Mire Garvan	Discontinuous
		Maleshevska	Continuous
		Begbunar	Continuous
		Tchokljovo Marsh	Discontinuous
		Vitosha	Not recorded
	Romania	Avrig I	Not recorded
		Mohos	Not recorded
		Luci	Not recorded
		Bisoca	Not recorded
		Calinease	Not recorded
		Semenic	Not recorded
		Steregoiu	Not recorded
	Hungary	Alsópáhok	Not recorded
		Balaton Centre	Not recorded
		Balaton Northeast	Not recorded

	Balaton Southwest	Not recorded
	Nagy-Mohos	Not recorded
	Pötréte	Not recorded
	Szigliget	Not recorded
Slovakia	Bobrov	Not recorded
	Zlatnicka Dolina	Not recorded
Czech Republic	Dvur Ansov	Not recorded
	Vracov	Not recorded
	Palasiny	Not recorded
	Kozli	Not recorded
	Rezabinec	Not recorded
	Borkovicka blata	Not recorded
	Branna	Not recorded
	Loucky	Not recorded
	Chrást	Not recorded
	Velka niva	Not recorded
	Malá niva	Not recorded
	Mrtv8 luh	Not recorded
	Strázenská slat	Not recorded
	Komoranské jezero	Not recorded
	Dolskym	Discontinuous
	Tpské raseliniste Mire III	Not recorded
Ukraina	Dovjok Swamp	Not recorded
	Maly Podleski	Not recorded
Croatia	Bokanjacko	Not recorded
Poland	Bledowo Lake	Not recorded
	Giecz	Not recorded
	Lake Gosciaz	Not recorded
	Lake Skrzetuszewskie	Not recorded
	Slawsko	Not recorded
	Swietokrzyskie Lake	Not recorded
	Cergowa Gora	Not recorded
	Godziszewskie Lake	Not recorded
	Lake Mikolajki	Not recorded
	Puscizna Rekowianska	Not recorded
Austria	Buntes Moor	Not recorded
	Dortmunder Hütte	Not recorded
	Franz Senn-Hütte	Not recorded
	Egelsee	Not recorded
	Gerlos	Not recorded
	Giering	Not recorded
	Grünau Moor	Not recorded
	Lindenmoos	Not recorded
	Mieminger See	Not recorded
	Moor Alpenrose	Not recorded
	Rotmoos Obergurgl	Not recorded
	Schwarzsee Reschenscheideck	Not recorded
	Schwemm	Not recorded
	Seefelder See	Not recorded
	Zirbenwaldmoor	Not recorded
	Wasenmoos beim Zellhof	Not recorded
	Grosses Überling Schattseit-Moor	Not recorded
	Dürrenecksee-Moor	Not recorded
	Fuchsschwanzmoos	Not recorded
	Fuschlsee	Not recorded
Germany	Ahlequellmoor	Not recorded
	Bruchberg	Not recorded
	Lüttersee	Not recorded
	Silberhohl	Not recorded
	Felchosee	Not recorded
	Löddigsee	Not recorded
	Wachel 3	Not recorded
	Brentenlohe	Not recorded
	Bruckmisse	Not recorded
	Glaswaldsee	Not recorded
	Herrenwiesser see	Not recorded
	Durchenbergried	Not recorded
	Feuenried	Not recorded
	Hornstaad-Bodensee	Not recorded
	Huzenbacher See	Not recorded
	Mindelsee	Not recorded
	Steerenmoos	Not recorded
	Wilder See beim Ruhestein	Not recorded
	Wildseemoor bei Kaltenbronn	Discontinuous
Switzerland	Etang de la Gruère	Not recorded
	Le Loclat	Not recorded

	Lobsigensee	Not recorded
	Montilier	Not recorded
	Aegelsee	Not recorded
	Amsoldingersee	Not recorded
	Bachalpsee	Not recorded
	Hinterburgseeli	Not recorded
	Hängstli	Not recorded
	Linden	Not recorded
	Oberaar	Not recorded
	Rotsee	Not recorded
	Schwarzsee FR	Not recorded
	Sägistalsee	Not recorded
	Süftenenegg	Not recorded
	Aletschwald	Discontinuous
	Alp Lüsga Belalp 1	Not recorded
	Alpi di Robièi Val Bavona	Not recorded
	Alpi di Robièi Val Bavona Bodenprofil	Not recorded
	Bitsch-Naters	Not recorded
	Eggen ob Blatten	Discontinuous
	Etang d'y Cor Montana	Not recorded
	Etang de Luissel Bex	Not recorded
	Gondo Alpjen	Not recorded
	Greicheralp Riederalp	Not recorded
	Grächen See	Not recorded
	Hopschensee	Not recorded
	Lac du Mont d'Orge Sion	Discontinuous
	Mittlere Hellelen	Not recorded
	Pillon Gsteig-Diablerets	Not recorded
	Simplon-Gampisch-Alter Spittel	Not recorded
	Wallbach Lenk	Not recorded
	Mont Roux	Not recorded
	Gamperfin	Not recorded
	Creux de Croue	Not recorded
	Motta Naluns	Not recorded
	Praz Rodet	Not recorded
Italy	Bondone	Not recorded
	Dura-Moor	Not recorded
	Malschötscher Hotter	Not recorded
	Rinderplatz	Not recorded
	Schwarzsee	Not recorded
	Sommersüss	Not recorded
	Grunsee	Not recorded
	Dossaccio Bormio	Not recorded
	Lac de Villa	Not recorded
	Lac du Verney-Dessus	Not recorded
	Torveraz	Not recorded
	Tourbière de Pilaz	Continuous
	Tourbière de Santa Anna	Not recorded
	Laghi dell'Orgials	Not recorded
	Lago Piccolo di Avigliana	Not recorded
	Ortasee	Not recorded
	Refugio Mondovi	Not recorded
	Selle di Carnino	Not recorded
	Torbiera del Biecai	Discontinuous
	Lago della Costa	Continuous
	Lago Padule	Not recorded
	Lago dell'Accesa	Not recorded
	Colfiorito	Not recorded
	Lago Pratignano	Not recorded
	Ospitale	Not recorded
	Pavullo	Not recorded
	Lago di Martignano	Continuous
	Lago di Vico	Not recorded
	Lago Albano	Discontinuous
	Lago di Nemi	Discontinuous
	Central Adriatic Sea	Discontinuous
	Salerno Bay	Not recorded
	Lago Grande di Monticchio LGM	Not recorded
France	Aronde	Not recorded
	Auneau	Not recorded
	Baie de Seine estuary	Not recorded
	Change-Glatinier	Not recorded
	La Vie	Not recorded
	Le Fourneau	Discontinuous
	Marais de Lisle	Discontinuous
	Moulin de Thévalles	Not recorded

Pezou	Not recorded
Saint-Ursin	Not recorded
Serrent	Not recorded
Altenweiher	Not recorded
Hières sur Amby	Not recorded
Lake of Annecy	Not recorded
Loras	Not recorded
Lutinière	Not recorded
Moselotte	Not recorded
Tourbière de Narbief	Not recorded
Le Grand Lemps	Not recorded
Tourbières des Granges des Chavants	Not recorded
Ampoix	Discontinuous
Champ Gazon	Not recorded
Etang de Cheylade	Not recorded
La Taphanel	Not recorded
Lac du Bouchet	Not recorded
Lac du Mont de Belier	Not recorded
Le Grand Montarnu	Not recorded
Le Jolan	Not recorded
Le Miroir	Not recorded
Marais du Grang Chaumet	Not recorded
Moulin de Prugnolas	Not recorded
Nataloup	Not recorded
Peyre peat-bog	Not recorded
Saint-Benoit-sur-Loire	Not recorded
Tourbière de Longeyroux	Not recorded
Tourbière de Roussy	Not recorded
Tourbière des Dauges	Not recorded
La Baforière	Not recorded
Tourbière de Chabannes	Not recorded
Ancenis	Discontinuous
Basse-Ville	Not recorded
Bois-Jésus	Not recorded
Carquefou	Not recorded
Caves d'Amont	Not recorded
Cinq-Mars-la-Pile	Not recorded
Cordemais	Not recorded
Corniche de Pail	Not recorded
Ecours	Discontinuous
Jaunay	Not recorded
La Caudelais	Not recorded
La Grande Brousse	Not recorded
La Grange	Discontinuous
La boire Torse	Discontinuous
Le Gesvres	Not recorded
Le Marais de la Perge	Not recorded
Marais de Champtocé	Not recorded
Marais de Mazerolles	Not recorded
Marais de Munet	Not recorded
Marais de Méron	Discontinuous
Marais de la Poupinière	Not recorded
Oudon	Not recorded
Pas du Gu	Not recorded
Petit Marais	Not recorded
Petit Rocher	Discontinuous
Riabelais	Not recorded
Saint Viaud Contin	Not recorded
Tourbière de Nay	Not recorded
Tourbière de Parcay-sur-Vienne	Not recorded
Vertonne	Not recorded
Clapeyret	Not recorded
Col Luitel	Not recorded
Col des Lauzes	Not recorded
Correo	Not recorded
Embouchac	Not recorded
Etang d'Ouveillan	Not recorded
Grand Ratz le Pellet	Not recorded
Lac Long Inférieur	Not recorded
Lac Saint Léger	Not recorded
Lac de Praver	Discontinuous
Lac des Boites	Not recorded
Lac du Lauzon	Discontinuous
Lake Racou	Not recorded
Pelléautier	Not recorded
Peuil Peat Bog	Not recorded

		Pré Rond	Not recorded
		Sabbion	Not recorded
		Saint Hilaire du Rosier	Not recorded
		Saint Julien de Ratz	Not recorded
		Saint Sixte	Discontinuous
		Tourbière de Gatimort	Not recorded
		Tourbière de Mont Sec	Not recorded
		Tourbière de la Lande	Not recorded
		Tourbière du Peschio	Not recorded
	Spain	Albufera Alcudia	Not recorded
		Algendar	Discontinuous
		Cala Galdana	Not recorded
		Cala'n Porter	Not recorded
		Antas	Not recorded
		Bajondillo	Not recorded
		Laguna Salada Chiprana	Not recorded
		Navarrés	Not recorded
		Atxuri	Not recorded
		Cueto de la Avellanosa	Not recorded
		Lago de Ajo	Not recorded
		Laguna de la Roya	Not recorded
		PRD-4	Discontinuous
		Puerto de Los Tornos	Not recorded
		Saldropo	Not recorded
		Sanabria Marsh	Not recorded
		Quintanar de la Sierra	Discontinuous
	Portugal	Charco da Candieira	Not recorded
		Lagoa Comprida 2	Not recorded
		Lagoa Travessa I	Not recorded
	Syria	Bouara	Not recorded
		Ghab	Not recorded
	Israel	Dead Sea-Ze'elim	Not recorded
		Birkat Ram	Discontinuous
		Lake Kinneret	Not recorded
		Hula Valley	Discontinuous
	Iran	Gomishan	Not recorded
		Lake Urmia	Not recorded
		Lake Zeribar	Not recorded
	Georgia	Gagra	Discontinuous
		Lagodekhi	Not recorded
		Akhali Ateni	Discontinuous
	Albania	Lake Maliq	Not recorded
5 - 4 Ka BP			
	Turkey	Marmara Sea	Discontinuous
		Abant Gölü	Continuous
		Yeniçaga Gölü	Not recorded
		Beysehir Gölü I	Not recorded
		Gölhisar Gölü I	Continuous
		Gölhisar Gölü II	Not recorded
		Hoyran Gölü	Discontinuous
		Kararmik Batakligi	Not recorded
		Köycegiz Gölü	Discontinuous
		Ova Gölü	Not recorded
		Pinarbasi	Continuous
		Sögüt Gölü	Not recorded
		Ladik Gölü	Continuous
		Lake Van	Discontinuous
	Greece	Kastoria	Not recorded
		Orestias	Continuous
		Khimaditis III	Not recorded
		Vegoritis	Not recorded
		Edessa	Continuous
		Litochoro	Not recorded
		Tenaghi Philippon	Not recorded
		Lerna	Discontinuous
		Trikhonis	Not recorded
		Ioannina I	Not recorded
		Ioannina II	Not recorded
		Halos	Not recorded
		Xinias	In expansion
		Voulkaria	Not recorded
		Pertouli	Discontinuous
		Myrtoon Basin	Continuous
	Bulgary	Beliya Kanton	Continuous
		Kupena II	Not recorded
		Ribno I	Discontinuous

	Popovo Ezero	Not recorded
	Sozopol	Continuous
	Arkutino I	Not recorded
	Arkutino II	Discontinuous
	Black Sea South	Not recorded
	Black Sea Southwest	Not recorded
	Duranunlak II	Not recorded
	Duranunlak I	Discontinuous
	Shabla-Ezeretz	Discontinuous
	Varna I	Discontinuous
	Varna II	Discontinuous
	Mire Garvan	Discontinuous
	Maleshevska	Continuous
	Begbunar	Continuous
	Straldzha mire	Not recorded
	Tchokljovo Marsh	Discontinuous
	Vitosha	Not recorded
Romania	Avrig I	Not recorded
	Avrig II	Not recorded
	Mohos	Not recorded
	Luci	Not recorded
	Bisoca	Not recorded
	Calinease	Not recorded
	Semenic	Not recorded
	Steregoiu	Not recorded
Hungary	Alsópáhok	Not recorded
	Balaton Centre	Not recorded
	Balaton Northeast	Not recorded
	Balaton Southwest	Not recorded
	Nagy-Mohos	Not recorded
	Pötréte	Not recorded
	Szigliget	Continuous
Slovakia	Bobrov	Not recorded
	Zlatnicka Dolina	Not recorded
	Strbské pleso	Not recorded
	Tlstá hora	Discontinuous
Czech Republic	Dvur Ansov	Not recorded
	Vracov	Not recorded
	Palasiny	Not recorded
	Kozli	Not recorded
	Rezabinec	Not recorded
	Borkovicka blata	Not recorded
	Branna	Not recorded
	Loucky	Discontinuous
	Chrást	Not recorded
	Velka niva	Not recorded
	Malá niva	Not recorded
	Mrtv8 luh	Not recorded
	Strázenská slat	Not recorded
	Komoranské jezero	Not recorded
	Dolskym	Not recorded
	Velky Ded	Not recorded
	Tpské raseliniste Mire III	Not recorded
Ukraina	Dovjok Swamp	Not recorded
	Maly Podleski	Not recorded
	Pecheniya	Not recorded
Croatia	Bokanjacko	Not recorded
Poland	Bledowo Lake	Not recorded
	Giecz	Not recorded
	Lake Gosciaz	Not recorded
	Lake Skrzetuszewskie	Not recorded
	Slawsko	Not recorded
	Swietokrzyskie Lake	Not recorded
	Cergowa Gora	Discontinuous
	Godziszewskie Lake	Not recorded
	Lake Mikolajki	Not recorded
	Puscizna Rekowianska	Not recorded
Austria	Buntes Moor	Not recorded
	Dortmunder Hütte	Not recorded
	Franz Senn-Hütte	Not recorded
	Egelsee	Not recorded
	Gerlos	Not recorded
	Giering	Not recorded
	Grünau Moor	Not recorded
	Lindenmoos	Not recorded
	Mieminger See	Not recorded

	Moor Alpenrose	Not recorded
	Rotmoos Obergurgl	Not recorded
	Schwarzsee Reschenscheideck	Not recorded
	Schwemm	Not recorded
	Seefelder See	Not recorded
	Zirbenwaldmoor	Not recorded
	Wasenmoos beim Zellhof	Not recorded
	Grosses Überling Schattseit-Moor	Not recorded
	Dürrenecksee-Moor	Discontinuous
	Fuchsschwanzmoos	Not recorded
	Fuschlsee	Not recorded
Germany	Ahlenmoor	Not recorded
	Ahlequellmoor	Not recorded
	Bruchberg	Not recorded
	Lüttersee	Not recorded
	Silberhohl	Not recorded
	Felchosee	Not recorded
	Löddigsee	Not recorded
	Wachel 3	Not recorded
	Brentenlohe	Not recorded
	Bruckmisse	Not recorded
	Glaswaldsee	Not recorded
	Herrenwiesser see	Not recorded
	Durchenbergried	Not recorded
	Feuenried	Not recorded
	Gaienhofen	Not recorded
	Hornstaad-Bodensee	Discontinuous
	Huzenbacher See	Not recorded
	Mindelsee	Not recorded
	Steerenmoos	Not recorded
	Wilder See beim Ruhestein	Not recorded
	Wildseemoor bei Kaltenbronn	Discontinuous
Switzerland	Derrière les Embreux	Not recorded
	Etang de la Gruère	Not recorded
	Le Loclat	Not recorded
	Lobsigensee	Not recorded
	Montilier	Not recorded
	Aegelsee	Not recorded
	Amsoldingersee	Not recorded
	Bachalpsee	Not recorded
	Hinterburgseeli	Not recorded
	Hängstli	Not recorded
	Linden	Not recorded
	Oberaar	Not recorded
	Rotsee	Not recorded
	Schwarzsee FR	Not recorded
	Sägistalsee	Not recorded
	Süftenenegg	Not recorded
	Aletschwald	Not recorded
	Alp Lüsga Belalp 1	Not recorded
	Alpi di Robièi Val Bavona	Not recorded
	Alpi di Robièi Val Bavona Bodenprofil	Not recorded
	Bitsch-Naters	Not recorded
	Eggen ob Blatten	Not recorded
	Etang d'y Cor Montana	Not recorded
	Etang de Luissel Bex	Discontinuous
	Gondo Alpjen	Discontinuous
	Greicheralp Riederalp	Not recorded
	Grächen See	Not recorded
	Hopschensee	Not recorded
	Lac du Mont d'Orge Sion	Not recorded
	Mittlere Hellelen	Not recorded
	Pillon Gsteig-Diablerets	Not recorded
	Simplon-Gampisch-Alter Spittel	Not recorded
	Wallbach Lenk	Not recorded
	Mont Roux	Not recorded
	Gamperfin	Not recorded
	Creux de Croue	Not recorded
	Motta Naluns	Not recorded
	Praz Rodet	Not recorded
Italy	Bondone	Not recorded
	Dura-Moor	Not recorded
	Malschötscher Hotter	Not recorded
	Rinderplatz	Not recorded
	Schwarzsee	Not recorded
	Sommersüss	Not recorded

	Grunsee	Not recorded
	Dossaccio Bormio	Not recorded
	Lac de Villa	Not recorded
	Lac du Verney-Dessus	Not recorded
	Torveraz	Not recorded
	Tourbière de Pilaz	Continuous
	Tourbière de Santa Anna	Not recorded
	Laghi dell'Orgials	Not recorded
	Lago Piccolo di Avigliana	Not recorded
	Ortasee	Not recorded
	Refugio Mondovi	Not recorded
	Selle di Carnino	Not recorded
	Torbiera del Biecai	Not recorded
	Lago della Costa	Continuous
	Lago Padule	Not recorded
	Lago dell'Accesa	Not recorded
	Colfiorito	Not recorded
	Lago Pratignano	Not recorded
	Ospitale	Not recorded
	Pavullo	Discontinuous
	Lago di Martignano	Continuous
	Lago di Vico	Not recorded
	Lago Albano	Discontinuous
	Lago di Nemi	Discontinuous
	Central Adriatic Sea	Discontinuous
	Salerno Bay	Discontinuous
	Lago Grande di Monticchio LGM	Not recorded
France	Aronde	Not recorded
	Auneau	Not recorded
	Baie de Seine estuary	Not recorded
	Change-Glatinier	Not recorded
	La Vie	Not recorded
	Le Fourneau	Not recorded
	Marais de Kerdual	Not recorded
	Marais de Lisle	Not recorded
	Marais de Marchesieux	Not recorded
	Mobeche Forest	Not recorded
	Moulin de Thévalles	Not recorded
	Pezou	Not recorded
	Saint-Ursin	Not recorded
	Serrent	Not recorded
	Altenweiher	Discontinuous
	Hières sur Amby	Not recorded
	Lac Cerin	Not recorded
	Lake of Annecy	Not recorded
	Loras	Not recorded
	Lutinière	Not recorded
	Moselotte	Not recorded
	Tourbière de Narbief	Not recorded
	Le Grand Lemps	Not recorded
	Tourbières des Granges des Chavants	Discontinuous
	Ampoix	Not recorded
	Champ Gazon	Not recorded
	Etang de Cheylade	Not recorded
	La Taphanel	Not recorded
	Lac du Bouchet	Not recorded
	Lac du Mont de Belier	Not recorded
	Le Grand Montarnu	Not recorded
	Le Jolan	Not recorded
	Le Miroir	Not recorded
	Marais du Grang Chaumet	Not recorded
	Montbé	Not recorded
	Moulin de Prugnolas	Not recorded
	Nataloup	Not recorded
	Peyre peat-bog	Not recorded
	Port des Lamberts	Not recorded
	Saint-Benoit-sur-Loire	Not recorded
	Tourbière de Longeyroux	Not recorded
	Tourbière de Roussy	Not recorded
	Tourbière des Dauges	Not recorded
	La Baforière	Not recorded
	Tourbière de Chabannes	Not recorded
	Ancenis	Discontinuous
	Basse-Ville	Not recorded
	Bois-Jésus	Not recorded
	Carquefou	Not recorded

	Caves d'Amont	Not recorded
	Cinq-Mars-la-Pile	Not recorded
	Cordemais	Not recorded
	Corniche de Pail	Not recorded
	Ecours	Not recorded
	Jaunay	Not recorded
	La Caudelais	Not recorded
	La Grande Brousse	Not recorded
	La Grange	Not recorded
	La boire Torse	Not recorded
	Le Gesvres	Not recorded
	Le Marais de la Perge	Not recorded
	Logne	Not recorded
	Marais de Champtocé	Not recorded
	Marais de Mazerolles	Not recorded
	Marais de Munet	Not recorded
	Marais de Méron	Not recorded
	Marais de la Poupinière	Not recorded
	Marais des Bourbes	Not recorded
	Oudon	Not recorded
	Pas du Gu	Not recorded
	Petit Marais	Not recorded
	Riabelais	Not recorded
	Saint Viaud Contin	Not recorded
	Tourbière de Nay	Not recorded
	Tourbière de Parcay-sur-Vienne	Not recorded
	Vertonne	Not recorded
	Clapeyret	Not recorded
	Col Luitel	Not recorded
	Col des Lauzes	Not recorded
	Correo	Not recorded
	Embouchac	Not recorded
	Etang d'Ouveillan	Not recorded
	Fangeas	Not recorded
	Grand Ratz le Pellet	Not recorded
	Lac Long Inférieur	Not recorded
	Lac Miroir	Not recorded
	Lac Saint Léger	Discontinuous
	Lac de Praver	Not recorded
	Lac des Boites	Not recorded
	Lac du Lauzon	Discontinuous
	Lake Racou	Not recorded
	Pelléautier	Not recorded
	Peuil Peat Bog	Discontinuous
	Pré Rond	Not recorded
	Sabbion	Not recorded
	Saint Hilaire du Rosier	Not recorded
	Saint Julien de Ratz	Continuous
	Saint Sixte	Discontinuous
	Tourbière de Gatimort	Not recorded
	Tourbière de Mont Sec	Not recorded
	Tourbière de la Lande	Not recorded
	Tourbière du Peschio	Not recorded
Spain	Albufera Alcudia	Not recorded
	Algendar	Not recorded
	Cala Galdana	Not recorded
	Cala'n Porter	Discontinuous
	Antas	Not recorded
	Bajondillo	Not recorded
	Laguna Salada Chiprana	Not recorded
	Navarrés	Discontinuous
	Atxuri	Not recorded
	Cueto de la Avellanosa	Not recorded
	Lago de Ajo	Not recorded
	Laguna de la Roya	Not recorded
	PRD-4	Not recorded
	Puerto de Los Tornos	Not recorded
	Saldropo	Not recorded
	Sanabria Marsh	Not recorded
	Peña Negra	Discontinuous
	Quintanar de la Sierra	Not recorded
	Salada Pequeña	Not recorded
Portugal	Charco da Candieira	Not recorded
	Lagoa Comprida 2	Discontinuous
	Lagoa Travessa I	Not recorded
Syria	Bouara	Not recorded

		Ghab	Not recorded
	Israel	Dead Sea-Ze'elim	Not recorded
		Birkat Ram	Discontinuous
		Lake Kinneret	Not recorded
		Hula Valley	Discontinuous
	Iran	Gomishan	Not recorded
		Lake Urmia	Not recorded
		Lake Zeribar	Not recorded
		Maharlou Lake	Discontinuous
	Georgia	Adange	Not recorded
		Gagra	Not recorded
		Lagodekhi	Not recorded
		Supsa River	Not recorded
		Imera lake	Not recorded
	Albania	Lake Maliq	Not recorded
4 – 3.5 Ka BP			
	Turkey	Lake Manyas	Not recorded
		Marmara Sea	Discontinuous
		Abant Gölü	Continuous
		Yeniçaga Gölü	Discontinuous
		Beysehir Gölü I	Continuous
		Gölhisar Gölü I	Continuous
		Gölhisar Gölü II	Not recorded
		Hoyran Gölü	Discontinuous
		Kararmik Batakligi	Not recorded
		Köycegiz Gölü	Not recorded
		Ova Gölü	Continuous
		Pinarbasi	Continuous
		Sögüt Gölü	Not recorded
		Ladik Gölü	Continuous
		Lake Van	Discontinuous
	Greece	Kournas	Not recorded
		Elatia-Rhodopes	Continuous
		Kastoria	Not recorded
		Orestias	Continuous
		Khimaditis III	Not recorded
		Vegoritis	Not recorded
		Edessa	Continuous
		Litochoro	Not recorded
		Tenaghi Philippon	Not recorded
		Lerna	Not recorded
		Trikhonis	Not recorded
		Ioannina I	Not recorded
		Ioannina II	Not recorded
		Halos	Discontinuous
		Xinias	Continuous
		Voulkaria	Not recorded
		Pertouli	Not recorded
		Myrtoon Basin	Continuous
	Bulgary	Beliya Kanton	Continuous
		Kupena II	Not recorded
		Ribno I	Discontinuous
		Popovo Ezero	Not recorded
		Mutorog	Not recorded
		Sozopol	Continuous
		Arkutino I	Not recorded
		Arkutino II	Discontinuous
		Black Sea South	Not recorded
		Black Sea Southwest	Not recorded
		Duranunlak II	Not recorded
		Duranunlak I	Not recorded
		Shabla-Ezeretz	Discontinuous
		Varna I	Discontinuous
		Varna II	Discontinuous
		Mire Garvan	Not recorded
		Maleshevska	Continuous
		Osogovo	Continuous
		Begbunar	Continuous
		Straldzha mire	Discontinuous
		Tchokljovo Marsh	Discontinuous
		Vitosha	Not recorded
	Romania	Avrig I	Not recorded
		Avrig II	Not recorded
		Mohos	Not recorded
		Luci	Not recorded
		Bisoca	Not recorded

	Calinease	Not recorded
	Semenic	Not recorded
	Steregoiu	Not recorded
Hungary	Alsópáhok	Not recorded
	Balaton Centre	Discontinuous
	Balaton Northeast	Discontinuous
	Balaton Southwest	Not recorded
	Nagy-Mohos	Discontinuous
	Pölöske	Not recorded
	Pötréte	Not recorded
	Szigliget	Continuous
Slovakia	Bobrov	Not recorded
	Zlatnicka Dolina	Not recorded
	Strbské pleso	Not recorded
	Tlstá hora	Not recorded
Czech Republic	Dvur Ansov	Discontinuous
	Olbramovice	Not recorded
	Vracov	Not recorded
	Palasiny	Not recorded
	Kozli	Not recorded
	Rezabinec	Not recorded
	Borkovicka blata	Not recorded
	Branna	Not recorded
	Loucky	Not recorded
	Chrást	Not recorded
	Velka niva	Not recorded
	Malá niva	Not recorded
	Mrtv8 luh	Not recorded
	Strázenská slat	Not recorded
	Komoranské jezero	Not recorded
	Dolskym	Not recorded
	Velky Ded	Not recorded
	Tpské raseliniste Mire II	Discontinuous
	Tpské raseliniste Mire III	Not recorded
Ukraina	Dovjok Swamp	Not recorded
	Maly Podleski	Not recorded
	Pecheniya	Not recorded
Croatia	Bokanjacko	Not recorded
Poland	Bledowo Lake	Not recorded
	Giecz	Not recorded
	Lake Gosciaz	Not recorded
	Lake Skrzetuszewskie	Not recorded
	Slawsko	Not recorded
	Swietokrzyskie Lake	Not recorded
	Cergowa Gora	Not recorded
	Godziszewskie Lake	Not recorded
	Lake Mikolajki	Not recorded
	Puscizna Rekowianska	Not recorded
Austria	Buntes Moor	Not recorded
	Dortmunder Hütte	Not recorded
	Franz Senn-Hütte	Not recorded
	Egelsee	Not recorded
	Gerlos	Not recorded
	Giering	Not recorded
	Grünau Moor	Not recorded
	Lindenmoos	Not recorded
	Mieminger See	Not recorded
	Moor Alpenrose	Not recorded
	Rotmoos Obergurgl	Not recorded
	Schwarzsee Reschenscheideck	Not recorded
	Schwemm	Not recorded
	Seefelder See	Not recorded
	Zirbenwaldmoor	Not recorded
	Wasenmoos beim Zellhof	Not recorded
	Grosses Überling Schattseit-Moor	Not recorded
	Dürrenecksee-Moor	Discontinuous
	Fuchsschwanzmoos	Not recorded
	Fuschlsee	Not recorded
Germany	Ahlenmoor	Not recorded
	Ahlequellmoor	Not recorded
	Bruchberg	Not recorded
	Lüttersee	Not recorded
	Silberhohl	Not recorded
	Felchosee	Not recorded
	Löddigsee	Not recorded
	Wachel 3	Not recorded

	Brentenlohe	Not recorded
	Bruckmisse	Not recorded
	Glaswaldsee	Not recorded
	Herrenwiesser see	Not recorded
	Durchenbergried	Not recorded
	Feuenried	Not recorded
	Gaienhofen	Not recorded
	Hornstaad-Bodensee	Not recorded
	Huzenbacher See	Not recorded
	Mindelsee	Not recorded
	Steerenmoos	Discontinuous
	Wilder See beim Ruhestein	Not recorded
	Wildseemoor bei Kaltenbronn	Discontinuous
Switzerland	Derrière les Embreux	Not recorded
	Etang de la Gruère	Not recorded
	Le Loclat	Not recorded
	Lobsigensee	Not recorded
	Montilier	Not recorded
	Aegelsee	Not recorded
	Amsoldingersee	Not recorded
	Bachalpsee	Not recorded
	Hinterburgseeli	Not recorded
	Hängstli	Not recorded
	Linden	Not recorded
	Oberaar	Not recorded
	Rotsee	Not recorded
	Schwarzsee FR	Not recorded
	Sägistalsee	Not recorded
	Süftenenegg	Not recorded
	Aletschwald	Not recorded
	Alp Lüsga Belalp 1	Not recorded
	Alpi di Robièi Val Bavona	Not recorded
	Alpi di Robièi Val Bavona Bodenprofil	Not recorded
	Bitsch-Naters	Not recorded
	Eggen ob Blatten	Not recorded
	Etang d'y Cor Montana	Not recorded
	Etang de Luissel Bex	Not recorded
	Gondo Alpjen	Not recorded
	Greicheralp Riederalp	Discontinuous
	Grächen See	Not recorded
	Hopschensee	Not recorded
	Lac du Mont d'Orge Sion	Not recorded
	Mittlere Hellelen	Not recorded
	Pillon Gsteig-Diablerets	Not recorded
	Simplon-Gampisch-Alter Spittel	Not recorded
	Wallbach Lenk	Not recorded
	Mont Roux	Not recorded
	Gamperfin	Not recorded
	Creux de Croue	Not recorded
	Motta Naluns	Not recorded
	Praz Rodet	Not recorded
Italy	Bondone	Continuous
	Dura-Moor	Not recorded
	Malschötscher Hotter	Not recorded
	Rinderplatz	Not recorded
	Schwarzsee	Not recorded
	Sommersüss	Not recorded
	Grunsee	Not recorded
	Dossaccio Bormio	Not recorded
	Lac de Villa	Not recorded
	Lac du Verney-Dessus	Not recorded
	Torveraz	Not recorded
	Tourbière de Pilaz	Continuous
	Tourbière de Santa Anna	Not recorded
	Laghi dell'Orgials	Not recorded
	Lago Piccolo di Avigliana	Not recorded
	Ortasee	Discontinuous
	Refugio Mondovi	Not recorded
	Selle di Carnino	Not recorded
	Torbiera del Biecai	Not recorded
	Lago della Costa	Continuous
	Lago Padule	Not recorded
	Lago dell'Accesa	Not recorded
	Colfiorito	Discontinuous
	Lago Pratignano	Discontinuous
	Ospitale	Not recorded

	Pavullo	Discontinuous
	Lago di Martignano	Continuous
	Lago di Vico	Not recorded
	Lago Albano	Discontinuous
	Lago di Nemi	Discontinuous
	Central Adriatic Sea	Discontinuous
	Salerno Bay	Discontinuous
	Lago Grande di Monticchio LGM	Not recorded
France	Aronde	Not recorded
	Auneau	Not recorded
	Baie de Seine estuary	Not recorded
	Change-Glatinier	Not recorded
	La Vie	Not recorded
	Le Fourneau	Discontinuous
	Marais de Kerdual	Discontinuous
	Marais de Lisle	Discontinuous
	Marais de Marchesieux	Not recorded
	Mobeche Forest	Not recorded
	Moulin de Thévalles	Not recorded
	Pezou	Not recorded
	Saint-Ursin	Not recorded
	Serrent	Not recorded
	Altenweiher	Not recorded
	Grozon	Not recorded
	Hières sur Amby	Not recorded
	Lac Cerin	Not recorded
	Lac de Clairvaux	Not recorded
	Lake of Annecy	Not recorded
	Loras	Not recorded
	Lutinière	Not recorded
	Moselotte	Not recorded
	Tourbière de Narbief	Not recorded
	Le Grand Lemps	Not recorded
	Tourbières des Granges des Chavants	Not recorded
	Ampoix	Not recorded
	Champ Gazon	Discontinuous
	Etang de Cheylade	Not recorded
	La Taphanel	Not recorded
	Lac du Bouchet	Not recorded
	Lac du Mont de Belier	Not recorded
	Le Grand Montarnu	Not recorded
	Le Jolan	Not recorded
	Le Miroir	Not recorded
	Marais du Grang Chaumet	In expansion
	Montbé	Not recorded
	Moulin de Prugnolas	Not recorded
	Nataloup	Not recorded
	Peyre peat-bog	Not recorded
	Port des Lamberts	Not recorded
	Saint-Benoit-sur-Loire	Not recorded
	Tourbière de Longeyroux	Not recorded
	Tourbière de Roussy	Not recorded
	Tourbière des Dauges	Not recorded
	La Baforière	Not recorded
	Tourbière de Chabannes	Not recorded
	Ancenis	Not recorded
	Basse-Ville	Not recorded
	Bois-Jésus	Not recorded
	Carquefou	Not recorded
	Caves d'Amont	Not recorded
	Cinq-Mars-la-Pile	Not recorded
	Cordemais	Not recorded
	Corniche de Pail	Not recorded
	Ecours	Discontinuous
	Jaunay	Not recorded
	La Bergerie en Charron	Not recorded
	La Caudelais	Discontinuous
	La Grande Brousse	Not recorded
	La Grange	Not recorded
	La boire Torse	Discontinuous
	Le Gesvres	Not recorded
	Le Marais de la Perge	Not recorded
	Logne	Not recorded
	Marais de Champtocé	Not recorded
	Marais de Mazerolles	Not recorded
	Marais de Munet	Not recorded

		Marais de Méron	Not recorded
		Marais de la Poupinière	Not recorded
		Marais des Bourbes	Not recorded
		Oudon	Not recorded
		Pas du Gu	Not recorded
		Petit Marais	Not recorded
		Riabelais	Not recorded
		Saint Viaud Contin	Not recorded
		Tourbière de Nay	Not recorded
		Tourbière de Parcay-sur-Vienne	Not recorded
		Vertonne	Not recorded
		Clapeyret	Not recorded
		Col Luitel	Not recorded
		Col des Lauzes	Not recorded
		Correo	Not recorded
		Embouchac	Not recorded
		Etang d'Ouveillan	Discontinuous
		Fangeas	Not recorded
		Grand Ratz le Pellet	Not recorded
		Lac Long Inférieur	Not recorded
		Lac Miroir	Discontinuous
		Lac Saint Léger	Not recorded
		Lac de Praver	Not recorded
		Lac des Boites	Not recorded
		Lac du Lauzon	Not recorded
		Lake Racou	Not recorded
		Pelléautier	Not recorded
		Pré Rond	Not recorded
		Sabbion	Not recorded
		Saint Hilaire du Rosier	Not recorded
		Saint Julien de Ratz	Continuous
		Saint Sixte	Discontinuous
		Tourbière de Gatimort	Not recorded
		Tourbière de Mont Sec	Not recorded
		Tourbière de la Lande	Not recorded
		Tourbière du Peschio	Not recorded
	Spain	Albufera Alcudia	Not recorded
		Algendar	Discontinuous
		Cala Galdana	Not recorded
		Cala'n Porter	Not recorded
		Antas	Not recorded
		Laguna Salada Chiprana	Not recorded
		Navarrés	Not recorded
		Atxuri	Not recorded
		Cueto de la Avellanosa	Not recorded
		Lago de Ajo	Discontinuous
		Laguna de la Roya	Not recorded
		PRD-4	Not recorded
		Puerto de Los Tornos	Not recorded
		Saldropo	Not recorded
		Sanabria Marsh	Discontinuous
		El Payo	Not recorded
		Peña Negra	Discontinuous
		Quintanar de la Sierra	Not recorded
		Salada Pequeña	Not recorded
	Portugal	Charco da Candieira	Not recorded
		Lagoa Comprida 2	Discontinuous
		Lagoa Travessa I	Not recorded
	Syria	Bouara	Not recorded
		Ghab	Discontinuous
	Israel	Dead Sea-Ze'elim	Not recorded
		Birkat Ram	Discontinuous
		Lake Kinneret	Not recorded
		Hula Valley	Discontinuous
	Iran	Gomishan	Not recorded
		Lake Almalou	Not recorded
		Lake Urmia	In expansion
		Lake Zeribar	Not recorded
		Maharlou Lake	Not recorded
	Georgia	Adange	Discontinuous
		Gagra	Not recorded
		Lagodekhi	Not recorded
		Supsa River	Not recorded
		Imera lake	Not recorded
	Albania	Lake Maliq	Discontinuous
3.5 – 3 Ka BP			

Turkey	Lake Manyas	Not recorded
	Marmara Sea	Discontinuous
	Abant Gölü	Continuous
	Yeniçaga Gölü	Not recorded
	Beysehir Gölü I	Continuous
	Gölhisar Gölü I	Continuous
	Gölhisar Gölü II	Not recorded
	Hoyran Gölü	Not recorded
	Kararmik Batakligi	Discontinuous
	Köycegiz Gölü	Discontinuous
	Ova Gölü	Continuous
	Pinarbasi	Continuous
	Sögüt Gölü	Not recorded
	Ladik Gölü	Continuous
	Lake Van	Discontinuous
Greece	Kournas	Not recorded
	Paiko	Not recorded
	Elatia-Rhodopes	Continuous
	Kastoria	Not recorded
	Orestias	Continuous
	Khimaditis III	Not recorded
	Vegoritis	Not recorded
	Edessa	Continuous
	Litochoro	Not recorded
	Tenaghi Philippon	Not recorded
	Lerna	Not recorded
	Trikhonis	Not recorded
	Ioannina I	Not recorded
	Ioannina II	Not recorded
	Halos	Not recorded
	Xinias	Continuous
	Voulkaria	Not recorded
	Pertouli	Not recorded
	Myrtoon Basin	Continuous
Bulgary	Beliya Kanton	Continuous
	Kupena II	Not recorded
	Ribno I	Not recorded
	Popovo Ezero	Not recorded
	Mutorog	Discontinuous
	Trilistnika	Continuous
	Sozopol	Continuous
	Arkutino I	Discontinuous
	Arkutino II	Discontinuous
	Black Sea South	Not recorded
	Black Sea Southwest	Not recorded
	Duranunlak II	Not recorded
	Duranunlak I	Not recorded
	Shabla-Ezeretz	Discontinuous
	Varna I	Not recorded
	Varna II	Discontinuous
	Mire Garvan	Not recorded
	Maleshevska	Continuous
	Osogovo	Continuous
	Begbunar	Continuous
	Sredna Gora	Not recorded
	Straldzha mire	Not recorded
	Tchokljovo Marsh	Not recorded
	Vitosha	Not recorded
Romania	Avrig I	Discontinuous
	Avrig II	Not recorded
	Mohos	Not recorded
	Luci	Not recorded
	Bisoca	Not recorded
	Calinease	Not recorded
	Semenic	Discontinuous
	Steregoiu	Not recorded
Hungary	Alsópáhok	Not recorded
	Balaton Centre	Discontinuous
	Balaton Northeast	Discontinuous
	Balaton Southwest	Not recorded
	Nagy-Mohos	Not recorded
	Pölöske	Not recorded
	Pötréte	Not recorded
	Szigliget	Continuous
Slovakia	Bobrov	Not recorded
	Zlatnicka Dolina	Not recorded

	Strbské pleso	Not recorded
	Tlstá hora	Not recorded
Czech Republic	Dvur Ansov	Not recorded
	Olbramovice	Discontinuous
	Vracov	Discontinuous
	Palasiny	Not recorded
	Kozli	Not recorded
	Rezabinec	Not recorded
	Borkovicka blata	Not recorded
	Branna	Discontinuous
	Loucky	Not recorded
	Chrást	Not recorded
	Velka niva	Not recorded
	Malá niva	Not recorded
	Mrtv8 luh	Not recorded
	Strázenská slat	Not recorded
	Komoranské jezero	Not recorded
	Dolskym	Not recorded
	Velky Ded	Not recorded
	Pancavská louka	Not recorded
	Tpské raseliniste Mire II	Not recorded
	Tpské raseliniste Mire III	Not recorded
Ukraina	Dovjok Swamp	Not recorded
	Maly Podleski	Not recorded
	Pecheniya	Discontinuous
Croatia	Bokanjacko	Not recorded
	Mljet	Continuous
Poland	Bledowo Lake	Not recorded
	Giecz	Not recorded
	Lake Gosciaz	Not recorded
	Lake Skrzetuszewskie	Not recorded
	Slawsko	Not recorded
	Swietokrzyskie Lake	Not recorded
	Cergowa Gora	Not recorded
	Godziszewskie Lake	Not recorded
	Lake Mikolajki	Not recorded
	Puscizna Rekowianska	Not recorded
Austria	Buntes Moor	Discontinuous
	Dortmunder Hütte	Not recorded
	Franz Senn-Hütte	Not recorded
	Egelsee	Not recorded
	Gerlos	Not recorded
	Giering	Not recorded
	Grünau Moor	Not recorded
	Lindenmoos	Not recorded
	Mieminger See	Not recorded
	Moor Alpenrose	Not recorded
	Rotmoos Obergurgl	Not recorded
	Schwarzsee Reschenscheideck	Not recorded
	Schwemm	Not recorded
	Seefelder See	Not recorded
	Zirbenwaldmoor	Not recorded
	Wasenmoos beim Zellhof	Discontinuous
	Grosses Überling Schattseit-Moor	Not recorded
	Dürrenecksee-Moor	Discontinuous
	Fuchsschwanzmoos	Discontinuous
	Fuschlsee	Not recorded
Germany	Ahlenmoor	Not recorded
	Ahlequellmoor	Not recorded
	Bruchberg	Not recorded
	Lüttersee	Not recorded
	Silberhohl	Not recorded
	Felchosee	Not recorded
	Löddigsee	Not recorded
	Wachel 3	Not recorded
	Brentenlohe	Not recorded
	Bruckmisse	Discontinuous
	Glaswaldsee	Not recorded
	Herrenwiesser see	Not recorded
	Durchenbergried	Not recorded
	Feuenried	Not recorded
	Gaienhofen	Not recorded
	Hornstaad-Bodensee	Not recorded
	Huzenbacher See	Not recorded
	Mindelsee	Not recorded
	Steerenmoos	Discontinuous

	Wilder See beim Ruhestein	Not recorded
	Wildseemoor bei Kaltenbronn	Discontinuous
Switzerland	Derrière les Embreux	Not recorded
	Etang de la Gruère	Not recorded
	Le Loclat	Not recorded
	Lobsigensee	Not recorded
	Montilier	Not recorded
	Aegelsee	Not recorded
	Amsoldingersee	Not recorded
	Bachalpsee	Not recorded
	Hinterburgseeli	Not recorded
	Hängstli	Not recorded
	Linden	Not recorded
	Oberaar	Not recorded
	Rotsee	Not recorded
	Schwarzsee FR	Not recorded
	Sägistalsee	Not recorded
	Süftenenegg	Not recorded
	Aletschwald	Not recorded
	Alp Lüsga Belalp 1	Not recorded
	Alpi di Robièi Val Bavona	Not recorded
	Alpi di Robièi Val Bavona Bodenprofil	Not recorded
	Bitsch-Naters	Not recorded
	Eggen ob Blatten	Not recorded
	Etang d'y Cor Montana	Not recorded
	Etang de Luissel Bex	Discontinuous
	Gondo Alpjen	Discontinuous
	Greicheralp Riederalp	In expansion
	Grächen See	Not recorded
	Hopschensee	Not recorded
	Lac du Mont d'Orge Sion	Discontinuous
	Mittlere Hellelen	Discontinuous
	Pillon Gsteig-Diablerets	Discontinuous
	Simplon-Gampisch-Alter Spittel	Not recorded
	Wallbach Lenk	Not recorded
	Mont Roux	Not recorded
	Gamperfin	Not recorded
	Creux de Croue	Not recorded
	Motta Naluns	Not recorded
	Praz Rodet	Not recorded
Italy	Bondone	Continuous
	Dura-Moor	Not recorded
	Malschötscher Hotter	Not recorded
	Rinderplatz	Not recorded
	Schwarzsee	Not recorded
	Sommersüss	Not recorded
	Grunsee	Discontinuous
	Dossaccio Bormio	Not recorded
	Lac de Villa	Not recorded
	Lac du Verney-Dessus	Not recorded
	Torveraz	Not recorded
	Tourbière de Pilaz	Continuous
	Tourbière de Santa Anna	Not recorded
	Laghi dell'Orgials	Not recorded
	Lago Piccolo di Avigliana	Not recorded
	Ortasee	Not recorded
	Refugio Mondovi	Not recorded
	Selle di Carnino	Not recorded
	Torbiera del Biecai	In expansion
	Lago della Costa	Continuous
	Lago Padule	Not recorded
	Lago dell'Accesa	In expansion
	Colfiorito	Not recorded
	Lago Pratignano	Not recorded
	Ospitale	Not recorded
	Pavullo	Discontinuous
	Lago di Martignano	Continuous
	Lago di Vico	Not recorded
	Lago Albano	In expansion
	Lago di Nemi	Not recorded
	Central Adriatic Sea	Discontinuous
	Salerno Bay	Discontinuous
	Lago Grande di Monticchio LGM	Not recorded
France	Aronde	Not recorded
	Auneau	Not recorded
	Baie de Seine estuary	Not recorded

Change-Glatinier	Not recorded
La Vie	Not recorded
Le Fourneau	Discontinuous
Marais de Kerdual	Discontinuous
Marais de Lisle	Discontinuous
Marais de Marchesieux	Not recorded
Mobeche Forest	Not recorded
Moulin de Thévalles	Not recorded
Pezou	Not recorded
Saint-Ursin	Not recorded
Serrent	Not recorded
Altenweiher	Not recorded
Grozon	Not recorded
Hières sur Amby	Discontinuous
Lac Cerin	Not recorded
Lac de Clairvaux	Not recorded
Lake of Annecy	Not recorded
Loras	Not recorded
Lutinière	Not recorded
Moselotte	Not recorded
Tourbière de Narbief	Not recorded
La Beuffarde	Not recorded
Le Grand Lemps	Not recorded
Tourbières des Granges des Chavants	Discontinuous
Ampoix	Not recorded
Champ Gazon	Not recorded
Etang de Cheylade	Not recorded
La Taphanel	Not recorded
Lac du Bouchet	Not recorded
Lac du Mont de Belier	Discontinuous
Le Grand Montarnu	Not recorded
Le Jolan	Not recorded
Le Miroir	Not recorded
Montbé	Not recorded
Moulin de Prugnolas	Discontinuous
Nataloup	Not recorded
Peyre peat-bog	Not recorded
Port des Lamberts	Not recorded
Saint-Benoit-sur-Loire	Not recorded
Tourbière de Longeyroux	Not recorded
Tourbière de Roussy	Not recorded
Tourbière des Dauges	Not recorded
La Baforière	Not recorded
Tourbière de Chabannes	Not recorded
Ancenis	Not recorded
Basse-Ville	Not recorded
Bois-Jésus	Not recorded
Carquefou	Not recorded
Caves d'Amont	Not recorded
Cinq-Mars-la-Pile	Not recorded
Cordemais	Not recorded
Corniche de Pail	Not recorded
Ecours	Discontinuous
Jaunay	Not recorded
La Bergerie en Charron	Not recorded
La Caudelais	Not recorded
La Grande Brousse	Discontinuous
La Grange	Discontinuous
La boire Torse	Not recorded
Le Gesvres	Not recorded
Le Marais de la Perge	Not recorded
Logne	Discontinuous
Marais de Champtocé	Not recorded
Marais de Mazerolles	Not recorded
Marais de Munet	Not recorded
Marais de Méron	Not recorded
Marais de la Poupinière	Not recorded
Marais des Bourbes	Not recorded
Oudon	Not recorded
Pas du Gu	Not recorded
Petit Marais	Not recorded
Riabelais	Not recorded
Saint Viaud Contin	Not recorded
Tourbière de Nay	Not recorded
Tourbière de Parcay-sur-Vienne	Not recorded
Vertonne	Not recorded

	Clapeyret	Discontinuous
	Col Luitel	Not recorded
	Col des Lauzes	Not recorded
	Correo	Not recorded
	Embouchac	Discontinuous
	Etang d'Ouveillan	Not recorded
	Fangeas	Not recorded
	Grand Ratz le Pellet	Not recorded
	Lac Long Inférieur	Not recorded
	Lac Miroir	Discontinuous
	Lac Saint Léger	Not recorded
	Lac de Praver	Not recorded
	Lac des Boites	Not recorded
	Lac du Lauzon	Discontinuous
	Lake Racou	Not recorded
	Pelléautier	Not recorded
	Pré Rond	Not recorded
	Sabbion	Not recorded
	Saint Hilaire du Rosier	Not recorded
	Saint Julien de Ratz	Continuous
	Saint Sixte	Discontinuous
	Tourbière de Gatimort	Not recorded
	Tourbière de Mont Sec	Not recorded
	Tourbière de Raux	Not recorded
	Tourbière de la Lande	Not recorded
	Tourbière du Peschio	Not recorded
Spain	Albufera Alcudia	Not recorded
	Algendar	Not recorded
	Cala Galdana	Not recorded
	Cala'n Porter	Discontinuous
	Antas	Not recorded
	Laguna Salada Chiprana	Not recorded
	Navarrés	Not recorded
	Atxuri	Not recorded
	Cueto de la Avellanosa	Not recorded
	Lago de Ajo	Discontinuous
	Laguna de la Roya	Not recorded
	PRD-4	Not recorded
	Puerto de Los Tornos	Not recorded
	Saldropo	Not recorded
	Sanabria Marsh	Not recorded
	El Payo	Not recorded
	Peña Negra	Discontinuous
	Quintanar de la Sierra	Not recorded
	Salada Pequeña	Not recorded
Portugal	Charco da Candieira	Not recorded
	Lagoa Comprida 2	Not recorded
	Lagoa Travessa I	Not recorded
Syria	Bouara	Not recorded
	Ghab	Not recorded
Israel	Dead Sea-Ze'elim	Not recorded
	Birkat Ram	Discontinuous
	Lake Kinneret	Not recorded
	Hula Valley	Discontinuous
Iran	Gomishan	Not recorded
	Lake Almalou	Not recorded
	Lake Urmia	Continuous
	Lake Zeribar	Discontinuous
	Maharlou Lake	Discontinuous
Georgia	Adange	Not recorded
	Gagra	Not recorded
	Lagodekhi	Not recorded
	Supsa River	Not recorded
	Lake Bazaleti	Not recorded
	Imera lake	Not recorded
	Lake Maliq (Albania)	Discontinuous
	Maharlou Lake (Iran)	Discontinuous
	Adange	Not recorded
	Gagra	Not recorded
	Lagodekhi	Not recorded
	Supsa River	Not recorded
	Lake Bazaleti	Not recorded
	Imera lake	Not recorded
	Lake Maliq (Albania)	Discontinuous
	Maharlou Lake (Iran)	Discontinuous
	Adange	Not recorded

		Gagra	Not recorded
		Lagodekhi	Not recorded
		Supsa River	Not recorded
		Lake Bazaleti	Not recorded
		Imera lake	Not recorded
	Albania	Lake Maliq	Discontinuous
3 – 2.5 Ka BP			
	Turkey	Lake Manyas	Continuous
		Marmara Sea	Discontinuous
		Abant Gölü	Continuous
		Yeniçaga Gölü	Not recorded
		Beysehir Gölü I	Continuous
		Gölhisar Gölü I	Continuous
		Gölhisar Gölü II	In expansion
		Hoyran Gölü	Not recorded
		Kararmik Batakligi	Discontinuous
		Köycegiz Gölü	Discontinuous
		Ova Gölü	In contraction
		Pinarbasi	Continuous
		Sögüt Gölü	Continuous
		Sagalassos-Gravgaz	Not recorded
		Ladik Gölü	Continuous
		Lake Van	Discontinuous
	Greece	Kournas	Discontinuous
		Paiko	Not recorded
		Lailias	Not recorded
		Elatia-Rhodopes	Continuous
		Kastoria	Not recorded
		Orestias	Continuous
		Khimaditis III	Continuous
		Vegoritis	Continuous
		Edessa	Continuous
		Litochoro	Continuous
		Tenaghi Philippon	Discontinuous
		Lerna	In expansion
		Trikhonis	Not recorded
		Ioannina I	Not recorded
		Ioannina II	Not recorded
		Halos	Discontinuous
		Xinias	Continuous
		Voulkaria	Not recorded
		Pertouli	Not recorded
		Myrtoon Basin	Continuous
	Bulgary	Beliya Kanton	Continuous
		Kupena II	Not recorded
		Ribno I	Discontinuous
		Popovo Ezero	Not recorded
		Mutorog	Not recorded
		Trilistnika	Continuous
		Sozopol	Continuous
		Arkutino I	Not recorded
		Arkutino II	Discontinuous
		Black Sea South	Not recorded
		Black Sea Southwest	Not recorded
		Duranunlak II	Not recorded
		Duranunlak I	Not recorded
		Shabla-Ezeretz	Discontinuous
		Varna I	Not recorded
		Varna II	Discontinuous
		Srebarna	Continuous
		Mire Garvan	Not recorded
		Maleshevska	Continuous
		Osogovo	Continuous
		Begbunar	Continuous
		Sredna Gora	Not recorded
		Straldzha mire	Not recorded
		Tchokljovo Marsh	Not recorded
		Vitosha	Not recorded
	Romania	Avrig I	Discontinuous
		Avrig II	Not recorded
		Mohos	Not recorded
		Luci	Not recorded
		Bisoca	Not recorded
		Calinease	Not recorded
		Semenic	Not recorded
		Steregoiu	Not recorded

Hungary	Alsópáhok	Not recorded
	Balaton Centre	Not recorded
	Balaton Northeast	In expansion
	Balaton Southwest	Not recorded
	Nagy-Mohos	Not recorded
	Pölöske	Not recorded
	Pötréte	Not recorded
	Szigliget	Continuous
Slovakia	Bobrov	Not recorded
	Zlatnicka Dolina	Not recorded
	Strbské pleso	Not recorded
	Tlstá hora	Not recorded
Czech Republic	Dvur Ansov	Not recorded
	Olbramovice	Not recorded
	Vracov	Discontinuous
	Palasiny	Not recorded
	Kozli	Not recorded
	Rezabinec	Not recorded
	Borkovicka blata	Not recorded
	Branna	Discontinuous
	Loucky	Not recorded
	Chrást	Not recorded
	Velka niva	Not recorded
	Malá niva	Not recorded
	Mrtv8 luh	Not recorded
	Strázenská slat	Not recorded
	Komoranské jezero	Not recorded
	Dolskym	Not recorded
	Velky Ded	Not recorded
	Pancavská louka	Not recorded
	Pryskyricn8 dul	Not recorded
	Tpské raseliniste Mire II	Not recorded
	Tpské raseliniste Mire III	Not recorded
Ukraina	Dovjok Swamp	Not recorded
	Maly Podleski	Not recorded
	Pecheniya	Not recorded
Croatia	Bokanjacko (Croatia)	Not recorded
	Mljet (Croatia)	Continuous
Poland	Bledowo Lake	Not recorded
	Giecz	Not recorded
	Lake Gosciaz	Not recorded
	Lake Skrzetuszewskie	Not recorded
	Slawsko	Discontinuous
	Swietokrzyskie Lake	Not recorded
	Cergowa Gora	Not recorded
	Godziszewskie Lake	Not recorded
	Lake Mikolajki	Not recorded
	Puscizna Rekowianska	Not recorded
	Buntes Moor (Austria)	Not recorded
Austria	Dortmunder Hütte	Not recorded
	Franz Senn-Hütte	Not recorded
	Egelsee	Not recorded
	Gerlos	Not recorded
	Giering	Not recorded
	Gradenmoos	Not recorded
	Grünau Moor	Not recorded
	Lindenmoos	Not recorded
	Mieminger See	Not recorded
	Moor Alpenrose	Discontinuous
	Rotmoos Obergurgl	Not recorded
	Schwarzsee Reschenscheideck	Not recorded
	Schwemm	Not recorded
	Seefelder See	Not recorded
	Zirbenwaldmoor	Not recorded
	Wasenmoos beim Zellhof	Not recorded
	Grosses Überling Schattseit-Moor	Not recorded
	Dürrenecksee-Moor	Not recorded
	Fuchsschwanzmoos	Not recorded
	Fuschlsee	Not recorded
Germany	Ahlenmoor	Not recorded
	Ahlequellmoor	Not recorded
	Bruchberg	Not recorded
	Lüttersee	Not recorded
	Silberhohl	Discontinuous
	Sonnenberger Moor	Not recorded
	Felchosee	Not recorded

	Löddigsee	Not recorded
	Wachel 3	Not recorded
	Brentenlohe	Not recorded
	Bruckmisse	Not recorded
	Glaswaldsee	Not recorded
	Herrenwiesser see	Discontinuous
	Durchenbergried	Not recorded
	Feuenried	Not recorded
	Gaienhofen	Not recorded
	Hornstaad-Bodensee	Not recorded
	Huzenbacher See	Not recorded
	Mindelsee	Not recorded
	Steerenmoos	Not recorded
	Wilder See beim Ruhestein	Not recorded
	Wildseemoor bei Kaltenbronn	Discontinuous
Switzerland	Derrière les Embreux	Not recorded
	Etang de la Gruère	Discontinuous
	Le Loclat	Not recorded
	Lobsigensee	Not recorded
	Montilier	Not recorded
	Aegelsee	Not recorded
	Amsoldingersee	Not recorded
	Bachalpsee	Not recorded
	Hinterburgseeli	Not recorded
	Hängstli	Not recorded
	Linden	Discontinuous
	Oberaar	Not recorded
	Rotsee	Discontinuous
	Schwarzsee FR	Not recorded
	Sägistalsee	Not recorded
	Süftenenegg	Not recorded
	Aletschwald	Not recorded
	Alp Lüsga Belalp 1	Not recorded
	Alpi di Robièi Val Bavona	Not recorded
	Alpi di Robièi Val Bavona Bodenprofil	Not recorded
	Bitsch-Naters	Not recorded
	Eggen ob Blatten	Not recorded
	Etang d'y Cor Montana	Not recorded
	Etang de Luissel Bex	Not recorded
	Gondo Alpjen	Not recorded
	Grächen See	Not recorded
	Hopschensee	Not recorded
	Lac du Mont d'Orge Sion	In expansion
	Mittlere Hellelen	Discontinuous
	Pillon Gsteig-Diablerets	Not recorded
	Simplon-Gampisch-Alter Spittel	Discontinuous
	Wallbach Lenk	Not recorded
	Mont Roux	Not recorded
	Gamperfin	Not recorded
	Creux de Croue	Not recorded
	Motta Naluns	Not recorded
	Praz Rodet	Not recorded
Italy	Dura-Moor	Not recorded
	Malschötscher Hotter	Not recorded
	Rinderplatz	Not recorded
	Schwarzsee	Discontinuous
	Sommersüss	Discontinuous
	Grunsee	Not recorded
	Dossaccio Bormio	Not recorded
	Lac de Lod	Not recorded
	Lac de Villa	Not recorded
	Lac du Verney-Dessus	Not recorded
	Torveraz	Not recorded
	Tourbière de Pilaz	Continuous
	Tourbière de Santa Anna	Not recorded
	Laghi dell'Orgials	Not recorded
	Lago Piccolo di Avigliana	Not recorded
	Ortasee	Not recorded
	Ortasee II	Not recorded
	Refugio Mondovi	Not recorded
	Selle di Carnino	Not recorded
	Torbiera del Biecai	Continuous
	Lago della Costa	Continuous
	Lago Padule	Not recorded
	Lago dell'Accesa	Continuous
	Colfiorito	Discontinuous

	Lago Pratignano	Discontinuous
	Ospitale	Not recorded
	Pavullo	Discontinuous
	Lago di Martignano	In contraction
	Lago di Vico	Continuous
	Lago Albano	Continuous
	Lago di Nemi	In expansion
	Central Adriatic Sea	Discontinuous
	Salerno Bay	Discontinuous
	Lago Grande di Monticchio LGM	Discontinuous
France	Aronde	Not recorded
	Auneau	Not recorded
	Baie de Seine estuary	Not recorded
	Change-Glatinier	Not recorded
	Coulvain	Not recorded
	La Verderie	Not recorded
	La Vie	Discontinuous
	Lavaré	Not recorded
	Le Fourneau	Not recorded
	Lingreville	Discontinuous
	Marais de Kerdual	Discontinuous
	Marais de Lisle	Not recorded
	Marais de Marchesieux	Discontinuous
	Mobeche Forest	Not recorded
	Moulin de Thévalles	Not recorded
	Pezou	Not recorded
	Saint-Ursin	Not recorded
	Serrent	Not recorded
	Altenweiher	In expansion
	Grozon	Discontinuous
	Hières sur Amby	Discontinuous
	Lac Cerin	Not recorded
	Lac de Clairvaux	Not recorded
	Lake of Annecy	Not recorded
	Loras	Discontinuous
	Lutinière	Not recorded
	Moselotte	Not recorded
	Tourbière de Narbief	Discontinuous
	col du Petit Saint Bernard	Not recorded
	La Beuffarde	Not recorded
	Le Grand Lemps	Not recorded
	Tourbière du Mou de Pleure (Francia)	Not recorded
	Tourbières des Granges des Chavants	Not recorded
	Ampoix	Discontinuous
	Champ Gazon	Not recorded
	Etang Bouquin	Not recorded
	Etang de Cheylade	Continuous
	La Taphanel	Not recorded
	Lac du Bouchet	Not recorded
	Lac du Mont de Belier	Not recorded
	Le Grand Montarnu	Not recorded
	Le Jolan	Not recorded
	Le Miroir	Not recorded
	Les Chaux de Coudert	Not recorded
	Marais de Maurepas	Not recorded
	Montbé	Not recorded
	Moulin de Prugnolas	Not recorded
	Nataloup	Not recorded
	Peyre peat-bog	Not recorded
	Port des Lamberts	Not recorded
	Saint-Benoit-sur-Loire	Not recorded
	Sources de l'Yonne	Not recorded
	Tourbière de Longeyroux	Not recorded
	Tourbière de Roussy	Not recorded
	Tourbière des Dauges	Not recorded
	La Baforière	Not recorded
	Tourbière de Chabannes	Not recorded
	Ancenis	Discontinuous
	Basse-Ville	Not recorded
	Bois-Jésus	Not recorded
	Carquefou	Not recorded
	Caves d'Amont	Not recorded
	Cinq-Mars-la-Pile	Not recorded
	Cordemais	Not recorded
	Corniche de Pail	Not recorded
	Jaunay	Not recorded

	La Bergerie en Charron	Discontinuous
	La Caudelais	Not recorded
	La Grande Brousse	Not recorded
	La Grange	Not recorded
	La boire Torse	Not recorded
	Le Gesvres	Not recorded
	Le Marais de la Perge	Not recorded
	Logne	Not recorded
	Marais de Champtocé	Discontinuous
	Marais de Mazerolles	Not recorded
	Marais de Munet	Not recorded
	Marais de Méron	Not recorded
	Marais de la Poupinière	Not recorded
	Marais des Bourbes	Discontinuous
	Oudon	Not recorded
	Pas du Gu	Not recorded
	Petit Marais	Discontinuous
	Riabelais	Not recorded
	Saint Viaud Contin	Not recorded
	Tourbière de Nay	Not recorded
	Tourbière de Parcay-sur-Vienne	Not recorded
	Vertonne	Discontinuous
	Clapeyret	Discontinuous
	Col Luitel	Continuous
	Col des Lauzes	In expansion
	Correo	Not recorded
	Embouchac	Not recorded
	Etang d'Ouveillan	Discontinuous
	Fangeas	Not recorded
	Grand Ratz le Pellet	Not recorded
	Lac Long Inférieur	Discontinuous
	Lac Miroir	Not recorded
	Lac Saint Léger	Not recorded
	Lac de Praver	Not recorded
	Lac des Boites	Continuous
	Lac du Lauzon	Discontinuous
	Lake Racou	Not recorded
	Pelléautier	Not recorded
	Pré Rond	Not recorded
	Sabbion	Discontinuous
	Saint Hilaire du Rosier	Not recorded
	Saint Julien de Ratz	Continuous
	Saint Sixte	Not recorded
	Tourbière de Gatimort	Not recorded
	Tourbière de Mont Sec	Not recorded
	Tourbière de Raux	Not recorded
	Tourbière de la Lande	Not recorded
	Tourbière du Peschio	Not recorded
Spain	Albufera Alcudia	Discontinuous
	Algendar	Discontinuous
	Cala Galdana	Not recorded
	Antas	Not recorded
	Laguna Salada Chiprana	Not recorded
	Navarrés	Not recorded
	Atxuri	Not recorded
	Cueto de la Avellanosa	Not recorded
	Lago de Ajo	In expansion
	Laguna de la Roya	Not recorded
	PRD-4	Not recorded
	Puerto de Los Tornos	Not recorded
	Saldropo	Not recorded
	Sanabria Marsh	Not recorded
	El Payo	Discontinuous
	Peña Negra	Discontinuous
	Quintanar de la Sierra	Discontinuous
	Salada Pequeña	Discontinuous
Portugal	Charco da Candieira	Not recorded
	Lagoa Comprida 2	Not recorded
	Lagoa Travessa I	Not recorded
Syria	Bouara	Discontinuous
	Ghab	Not recorded
Israel	Dead Sea-Ze'elim	Not recorded
	Birkat Ram	In expansion
	Lake Kinneret	Not recorded
	Hula Valley	Discontinuous
Iran	Gomishan	Not recorded

		Lake Almalou	Not recorded
		Lake Urmia	Continuous
		Lake Zeribar	Discontinuous
		Maharlou Lake	In expansion
	Georgia	Adange	Discontinuous
		Amtkel	Not recorded
		Gagra	Not recorded
		Lagodekhi	Not recorded
		Supsa River	Not recorded
		Lake Bazaleti	Continuous
		Imera lake	Not recorded
	Albania	Lake Maliq	Discontinuous
2.5 – 2 Ka BP			
	Turkey	Lake Manyas	Continuous
		Marmara Sea	In expansion
		Abant Gölü	In contraction
		Beysehir Gölü I	Continuous
		Gölhisar Gölü I	Continuous
		Gölhisar Gölü II	Continuous
		Hoyran Gölü	In expansion
		Kararmik Batakligi	Not recorded
		Köycegiz Gölü	Not recorded
		Ova Gölü	Discontinuous
		Pinarbasi	Continuous
		Sögüt Gölü	Continuous
		Sagalassos-Gravgaz	Continuous
		Ladik Gölü	Continuous
		Lake Van	Discontinuous
	Greece	Kournas	Discontinuous
		Paiko	Continuous
		Lailias	Not recorded
		Elatia-Rhodopes	Continuous
		Kastoria	Not recorded
		Orestias	Continuous
		Khimaditis III	Continuous
		Vegoritis	Continuous
		Edessa	Continuous
		Litochoro	Continuous
		Tenaghi Philippon	Discontinuous
		Lerna	Continuous
		Trikhonis	Continuous
		Ioannina I	Discontinuous
		Ioannina II	Not recorded
		Halos	Not recorded
		Xinias	Continuous
		Voulkaria	Not recorded
		Pertouli	Not recorded
		Myrtoon Basin	Continuous
	Bulgary	Beliya Kanton	Continuous
		Kupena II	Discontinuous
		Ribno I	In expansion
		Popovo Ezero	Not recorded
		Mutorog	Not recorded
		Trilistnika	Continuous
		Suho Ezero	Discontinuous
		Sozopol	Continuous
		Arkutino I	Discontinuous
		Arkutino II	Not recorded
		Black Sea South	Not recorded
		Black Sea Southwest	Not recorded
		Duranunlak II	Discontinuous
		Duranunlak I	Not recorded
		Shabla-Ezeretz	Discontinuous
		Varna I	Discontinuous
		Varna II	Discontinuous
		Srebarna	Continuous
		Mire Garvan	Discontinuous
		Maleshevska	Continuous
		Osogovo	Continuous
		Begbunar	Continuous
		Sredna Gora	Not recorded
		Straldzha mire	Not recorded
		Tchokljovo Marsh	Not recorded
		Vitosha	Not recorded
	Romania	Avrig I	Discontinuous
		Avrig II	Not recorded

	Mohos	Not recorded
	Luci	Discontinuous
	Bisoca	Not recorded
	Calinease	Not recorded
	Semenic	Not recorded
	Steregoiu	Not recorded
Hungary	Alsópáhok	Not recorded
	Balaton Centre	Discontinuous
	Balaton Northeast	Continuous
	Balaton Southwest	Not recorded
	Nagy-Mohos	Discontinuous
	Pölöske	Not recorded
	Pötréte	Discontinuous
	Szigliget	Continuous
Slovakia	Bobrov	Not recorded
	Zlatnicka Dolina	Not recorded
	Strbské pleso	Not recorded
	Tlstá hora	In expansion
Czech Republic	Dvur Ansov	Not recorded
	Olbramovice	Discontinuous
	Vracov	Discontinuous
	Palasiny	Not recorded
	Kozli	Not recorded
	Rezabinec	Not recorded
	Borkovicka blata	Not recorded
	Branna	Not recorded
	Loucky	Discontinuous
	Chrást	Not recorded
	Velka niva	Not recorded
	Malá niva	Not recorded
	Mrtv8 luh	Not recorded
	Strázenská slat	Not recorded
	Komoranské jezero	Not recorded
	Dolskym	Not recorded
	Na bahne	Not recorded
	Velky Ded	Not recorded
	Pancavská louka	Not recorded
	Pryskyricn8 dul	Not recorded
	Tpské raseliniste Mire II	Not recorded
	Tpské raseliniste Mire III	Not recorded
Ukraina	Dovjok Swamp	Not recorded
	Maly Podleski	Not recorded
	Pecheniya	Not recorded
Croatia	Bokanjacko	Discontinuous
	Mljet	Continuous
Poland	Bledowo Lake	Not recorded
	Giecz	Not recorded
	Lake Gosciaz	Not recorded
	Lake Skrzetuszewskie	Not recorded
	Slawsko	Not recorded
	Swietokrzyskie Lake	Not recorded
	Cergowa Gora	Not recorded
	Godziszewskie Lake	Not recorded
	Lake Mikolajki	Not recorded
	Puscizna Rekowianska	Not recorded
Austria	Buntes Moor	Discontinuous
	Dortmunder Hütte	Not recorded
	Franz Senn-Hütte	Not recorded
	Egelsee	Not recorded
	Gerlos	Not recorded
	Giering	Not recorded
	Gradenmoos	Not recorded
	Grünau Moor	Not recorded
	Lindenmoos	Not recorded
	Mieminger See	Not recorded
	Moor Alpenrose	Not recorded
	Rotmoos Obergurgl	Not recorded
	Schwarzsee Reschenscheideck	Not recorded
	Schwemm	Not recorded
	Seefelder See	Not recorded
	Zirbenwaldmoor	Not recorded
	Wasenmoos beim Zellhof	Not recorded
	Grosses Überling Schattseit-Moor	Not recorded
	Dürrenecksee-Moor	Not recorded
	Fuchsschwanzmoos	Not recorded
	Fuschlsee	Not recorded

Germany	Ahlenmoor	Not recorded
	Ahlequellmoor	Not recorded
	Bruchberg	Not recorded
	Lüttersee	Not recorded
	Silberhohl	Discontinuous
	Sonnenberger Moor	Not recorded
	Dunum (Hilliges Moor)	Not recorded
	Felchosee	Not recorded
	Löddigsee	Not recorded
	Wachel 3	Not recorded
	Brentenlohe	Discontinuous
	Bruckmisse	Discontinuous
	Glaswaldsee	Discontinuous
	Herrenwiesser see	Discontinuous
	Durchenbergried	Discontinuous
	Feuenried	Not recorded
	Gaienhofen	Not recorded
	Hornstaad-Bodensee	Discontinuous
	Huzenbacher See	Discontinuous
	Mindelsee	Discontinuous
	Oberderdingen-Großvillars	Not recorded
	Steerenmoos	Not recorded
	Wilder See beim Ruhestein	Not recorded
Switzerland	Derrière les Embreux	Not recorded
	Etang de la Gruère	Not recorded
	Le Loclat	Continuous
	Lobsigensee	Continuous
	Montilier	Continuous
	Aegelsee	Not recorded
	Amsoldingersee	Not recorded
	Bachalpsee	Not recorded
	Hinterburgseeli	Discontinuous
	Hängstli	Not recorded
	Linden	Not recorded
	Oberaar	Not recorded
	Rotsee	Not recorded
	Schwarzsee FR	Discontinuous
	Sägistalsee	Not recorded
	Süftenenegg	Not recorded
	Aletschwald	Not recorded
	Alp Lüsga Belalp 1	Not recorded
	Alpi di Robièi Val Bavona	Not recorded
	Alpi di Robièi Val Bavona Bodenprofil	Discontinuous
	Bitsch-Naters	Not recorded
	Eggen ob Blatten	Not recorded
	Etang d'y Cor Montana	Discontinuous
	Etang de Luissel Bex	In expansion
	Gondo Alpjen	Not recorded
	Grächen See	Discontinuous
	Hopschensee	Continuous
	Lac du Mont d'Orge Sion	Continuous
	Mittlere Hellelen	Not recorded
	Pillon Gsteig-Diablerets	Not recorded
	Wallbach Lenk	Not recorded
	Mont Roux	Not recorded
	Gamperfin	Not recorded
	Creux de Croue	Not recorded
	Motta Naluns	Discontinuous
	Praz Rodet	Discontinuous
Italy	Dura-Moor	Discontinuous
	Malschötscher Hotter	Continuous
	Rinderplatz	Not recorded
	Schwarzsee	Discontinuous
	Sommersüss	Discontinuous
	Grunsee	Not recorded
	Dossaccio Bormio	Not recorded
	Lac de Lod	Not recorded
	Lac de Villa	Continuous
	Lac du Verney-Dessus	Discontinuous
	Torveraz	Discontinuous
	Tourbière de Pilaz	Continuous
	Tourbière de Santa Anna	Not recorded
	Laghi dell'Orgials	Not recorded
	Lago Piccolo di Avigliana	Continuous
	Ortasee	Discontinuous
	Ortasee II	Discontinuous

	Refugio Mondovi	Not recorded
	Selle di Carnino	Not recorded
	Torbiera del Biecai	Continuous
	Lago della Costa	Continuous
	Lago Padule	Not recorded
	Colfiorito	Discontinuous
	Lago Pratignano	Discontinuous
	Ospitale	Not recorded
	Pavullo	Discontinuous
	Parma	Discontinuous
	Lago di Martignano	Discontinuous
	Lago di Vico	Continuous
	Lago Albano	Continuous
	Lago di Nemi	Continuous
	Central Adriatic Sea	In expansion
	Salerno Bay	Discontinuous
	Lago Grande di Monticchio LGM	Discontinuous
	Bradano Valley	In contraction
	Aronde (France)	Discontinuous
France	Auneau	Not recorded
	Baie de Seine estuary	Not recorded
	Change-Glatinier	Not recorded
	Coulvain	Not recorded
	La Verderie	Not recorded
	La Vie	Not recorded
	Lavaré	Not recorded
	Le Fourneau	Not recorded
	Lingreville	Not recorded
	Marais de Kerdual	Not recorded
	Marais de Lisle	Discontinuous
	Marais de Marchesieux	Discontinuous
	Mobeche Forest	Not recorded
	Moulin de Thévalles	Not recorded
	Pezou	Discontinuous
	Rimarde	Discontinuous
	Saint-Ursin	Discontinuous
	Serrent	Not recorded
	Grozon	Discontinuous
	Hières sur Amby	Discontinuous
	Lac Cerin	Not recorded
	Lac de Clairvaux	Not recorded
	Lac de Malpas	Not recorded
	Lake of Annecy	Discontinuous
	Loras	In expansion
	Lutinière	Not recorded
	Moselotte	Not recorded
	Tourbière de Narbief	Discontinuous
	col du Petit Saint Bernard	Not recorded
	La Beuffarde	Not recorded
	Le Grand Lemps	Continuous
	Tourbière de Censeau	Not recorded
	Tourbière du Mou de Pleure (Francia)	Not recorded
	Tourbières des Granges des Chavants	Not recorded
	Ampoix	Discontinuous
	Champ Gazon	Not recorded
	Etang Bouquin	Discontinuous
	Etang de Cheylade	Continuous
	Etang de la Villetelle	Not recorded
	La Taphanel	Discontinuous
	Lac du Bouchet	Not recorded
	Lac du Mont de Belier	Not recorded
	Le Grand Montarnu	Not recorded
	Le Jolan	Not recorded
	Le Miroir	Not recorded
	Les Chaux de Coudert	Continuous
	Marais de Maurepas	Not recorded
	Montbé	Not recorded
	Moulin de Prugnolas	Discontinuous
	Nataloup	Not recorded
	Peyre peat-bog	Not recorded
	Port des Lamberts	Discontinuous
	Saint-Benoit-sur-Loire	In expansion
	Sources de l'Yonne	Not recorded
	Tourbière de Longeyroux	Not recorded
	Tourbière de Roussy	Not recorded
	Tourbière des Dauges	Not recorded

	La Baforière	Continuous
	Tourbière de Chabannes	Not recorded
	Font Carluze	Not recorded
	Ancenis	Not recorded
	Basse-Ville	Not recorded
	Bois-Jésus	Not recorded
	Carquefou	Not recorded
	Caves d'Amont	Discontinuous
	Cinq-Mars-la-Pile	In expansion
	Cordemais	Not recorded
	Corniche de Pail	Not recorded
	Jaunay	Not recorded
	La Bergerie en Charron	Not recorded
	La Caudelais	Not recorded
	La Grande Brousse	Not recorded
	La Grange	Not recorded
	La Prairie du Cassoir	Not recorded
	La boire Torse	Not recorded
	Le Gesvres	Not recorded
	Le Marais de la Perge	Not recorded
	Logne	Not recorded
	Marais de Champtocé	Not recorded
	Marais de Mazerolles	Not recorded
	Marais de Munet	Not recorded
	Marais de Méron	Not recorded
	Marais de la Poupinière	Not recorded
	Marais des Bourbes	Not recorded
	Oudon	Discontinuous
	Pas du Gu	Not recorded
	Petit Marais	Discontinuous
	Riabelais	Not recorded
	Saint Viaud Contin	Not recorded
	Tourbière de Nay	Not recorded
	Tourbière de Parcay-sur-Vienne	Not recorded
	Vertonne	Discontinuous
	Col Luitel	Continuous
	Col des Lauzes	Continuous
	Correo	Not recorded
	Embouchac	Discontinuous
	Etang d'Ouveillan	Not recorded
	Fangeas	Not recorded
	Grand Ratz le Pellet	Not recorded
	Lac Long Inférieur	Discontinuous
	Lac Miroir	Discontinuous
	Lac Saint Léger	Not recorded
	Lac de Praver	In expansion
	Lac des Boites	Continuous
	Lac du Lauzon	In expansion
	Lake Racou	Not recorded
	Pelléautier	Not recorded
	Pré Rond	Not recorded
	Sabbion	Discontinuous
	Saint Hilaire du Rosier	Discontinuous
	Saint Julien de Ratz	Continuous
	Saint Sixte	Discontinuous
	Tourbière de Gatimort	Not recorded
	Tourbière de Mont Sec	Discontinuous
	Tourbière de Raux	Not recorded
	Tourbière de la Lande	Not recorded
	Tourbière des Narses Mortes	Not recorded
	Tourbière du Peschio	Not recorded
Spain	Albufera Alcudia	Discontinuous
	Algendar	Not recorded
	Cala Galdana	Discontinuous
	Antas	Not recorded
	Laguna Salada Chiprana	Not recorded
	Atxuri	Not recorded
	Cueto de la Avellanosa	Continuous
	Laguna de la Roya	Not recorded
	PRD-4	Discontinuous
	Puerto de Los Tornos	Not recorded
	Saldropo	Not recorded
	Sanabria Marsh	Not recorded
	El Payo	Discontinuous
	Lanzahíta	Continuous
	Pataderos bog	Not recorded

		Peña Negra	Not recorded
		Quintanar de la Sierra	Not recorded
		Salada Pequeña	Not recorded
	Portugal	Charco da Candieira	Not recorded
		Lagoa Comprida 2	Not recorded
		Lagoa Travessa I	Not recorded
	Syria	Bouara	Not recorded
		Ghab	Not recorded
	Israel	Dead Sea-Ze'elim	Discontinuous
		Birkat Ram	Continuous
		Lake Kinneret	Discontinuous
		Hula Valley	Discontinuous
	Iran	Gomishan	Discontinuous
		Lake Almalou	Discontinuous
		Lake Zeribar	Discontinuous
		Maharlou Lake	Continuous
	Georgia	Adange	Discontinuous
		Amtkel	Discontinuous
		Gagra	Not recorded
		Lagodekhi	Not recorded
		Sibista	Not recorded
		Supsa River	Not recorded
		Lake Bazaleti	Continuous
		Imera lake	Continuous
	Albania	Lake Maliq	Discontinuous
2 – 1.5 Ka BP			
	Turkey	Lake Manyas	Continuous
		Marmara Sea	Continuous
		Abant Gölü	Not recorded
		Beysehir Gölü I	Continuous
		Gölhisar Gölü I	Continuous
		Gölhisar Gölü II	Continuous
		Hoyran Gölü	Continuous
		Kararmik Batakligi	Not recorded
		Köycegiz Gölü	Discontinuous
		Ova Gölü	Not recorded
		Pinarbasi	Continuous
		Sögüt Gölü	Continuous
		Sagalassos-Gravgaz	Continuous
		Ladik Gölü	Continuous
		Lake Van	In expansion
	Greece	Kournas	Discontinuous
		Paiko	Continuous
		Lailias	In expansion
		Elatia-Rhodopes	Continuous
		Kastoria	Continuous
		Orestias	Continuous
		Khimaditis III	Continuous
		Vegoritis	Continuous
		Edessa	Continuous
		Litochoro	Continuous
		Lerna	Continuous
		Trikhonis	Continuous
		Ioannina I	Not recorded
		Ioannina II	Not recorded
		Halos	Not recorded
		Xinias	Continuous
		Voulkaria	Not recorded
		Pertouli	Not recorded
		Myrtoon Basin	Continuous
	Bulgary	Beliya Kanton	Continuous
		Kupena II	Not recorded
		Ribno I	Continuous
		Popovo Ezero	Not recorded
		Mutorog	In expansion
		Trilistnika	Continuous
		Suho Ezero	Discontinuous
		Sozopol	Continuous
		Arkutino I	Not recorded
		Arkutino II	Not recorded
		Black Sea South	Not recorded
		Black Sea Southwest	Discontinuous
		Duranunlak II	Not recorded
		Duranunlak I	Not recorded
		Shabla-Ezeretz	Not recorded
		Varna I	Discontinuous

	Varna II	Discontinuous
	Srebarna	Continuous
	Mire Garvan	In expansion
	Osogovo	Continuous
	Begbunar	Continuous
	Sredna Gora	Not recorded
	Straldzha mire	Discontinuous
	Tchokljovo Marsh	Not recorded
	Vitosha	Not recorded
Romania	Avrig I	Not recorded
	Avrig II	Not recorded
	Mohos	Not recorded
	Luci	Discontinuous
	Bisoca	Not recorded
	Calinease	Not recorded
	Semenic	Not recorded
	Steregoiu	Not recorded
Hungary	Alsópáhok	Continuous
	Balaton Centre	In expansion
	Balaton Northeast	Continuous
	Balaton Southwest	Not recorded
	Nagy-Mohos	Not recorded
	Pölöske	Not recorded
	Pötréte	Discontinuous
	Szigliget	Continuous
Slovakia	Bobrov	Not recorded
	Zlatnicka Dolina	Not recorded
	Strbské pleso	Not recorded
	Tlstá hora	Continuous
Czech Republic	Dvur Ansov	Not recorded
	Olbramovice	Discontinuous
	Vracov	Not recorded
	Palasiny	Not recorded
	Kozli	Not recorded
	Rezabinec	Not recorded
	Borkovicka blata	Not recorded
	Branna	Discontinuous
	Loucky	Not recorded
	Chrást	Not recorded
	Velka niva	Not recorded
	Malá niva	Not recorded
	Mrtv8 luh	Not recorded
	Strázenská slat	Discontinuous
	Komoranské jezero	Not recorded
	Dolskym	Not recorded
	Na bahne	Not recorded
	Velky Ded	Not recorded
	Velky Maj	Continuous
	Pancavská louka	Not recorded
	Pryskyricn8 dul	Not recorded
	Tpské raseliniste Mire	Not recorded
	Tpské raseliniste Mire II	Not recorded
	Tpské raseliniste Mire III	Discontinuous
Ukraina	Dovjok Swamp	Not recorded
	Maly Podleski	Discontinuous
	Pecheniya	Discontinuous
Croatia	Bokanjacko	Discontinuous
	Mljet	Continuous
Poland	Bledowo Lake	Not recorded
	Giecz	Not recorded
	Lake Gosciaz	Not recorded
	Lake Skrzetuszewskie	Not recorded
	Slawsko	Not recorded
	Swietokrzyskie Lake	Not recorded
	Cergowa Gora	Not recorded
	Godziszewskie Lake	Not recorded
	Lake Mikolajki	Not recorded
	Puscizna Rekowianska	Not recorded
Austria	Buntes Moor	In expansion
	Dortmunder Hütte	Not recorded
	Franz Senn-Hütte	Not recorded
	Egelsee	In expansion
	Gerlos	Continuous
	Giering	Not recorded
	Gradenmoos	Not recorded
	Grünau Moor	Not recorded

	Lindenmoos	Discontinuous
	Mieminger See	Not recorded
	Moor Alpenrose	Not recorded
	Rotmoos Obergurgl	Not recorded
	Schwarzsee Reschenscheideck	Discontinuous
	Schwemm	Not recorded
	Seefelder See	In expansion
	Zirbenwaldmoor	Discontinuous
	Wasenmoos beim Zellhof	Not recorded
	Grosses Überling Schattseit-Moor	Not recorded
	Dürrenecksee-Moor	Not recorded
	Fuchsschwanzmoos	Not recorded
	Fuschlsee	Not recorded
Germany	Ahlenmoor	Not recorded
	Ahlequellmoor	Not recorded
	Bruchberg	Not recorded
	Lüttersee	Not recorded
	Silberhohl	Not recorded
	Sonnenberger Moor	Not recorded
	Dunum (Hilliges Moor)	Not recorded
	Felchosee	Not recorded
	Großer Krebssee	Not recorded
	Löddigsee	Not recorded
	Wachel 3	Not recorded
	Brentenlohe	Not recorded
	Bruckmisse	Not recorded
	Glaswaldsee	Discontinuous
	Herrenwiesser see	Discontinuous
	Durchenbergried	Discontinuous
	Feuenried	Not recorded
	Gaienhofen	Not recorded
	Hornstaad-Bodensee	In expansion
	Huzenbacher See	Discontinuous
	Mindelsee	In expansion
	Oberderdingen-Großvillars	Not recorded
	Steerenmoos	Not recorded
	Wilder See beim Ruhestein	Not recorded
Switzerland	Derrière les Embreux	Not recorded
	Etang de la Gruère	Not recorded
	Le Loclat	Continuous
	Lobsigensee	Continuous
	Montilier	Continuous
	Aegelsee	Not recorded
	Amsoldingersee	Not recorded
	Bachalpsee	Discontinuous
	Hinterburgseeli	In expansion
	Hängstli	Discontinuous
	Linden	Discontinuous
	Oberaar	Not recorded
	Rotsee	In expansion
	Schwarzsee FR	Discontinuous
	Sägistalsee	Discontinuous
	Süftenenegg	Discontinuous
	Aletschwald	In expansion
	Alp Lüsga Belalp 1	Not recorded
	Alpi di Robièi Val Bavona	Not recorded
	Alpi di Robièi Val Bavona Bodenprofil	Not recorded
	Bitsch-Naters	In expansion
	Eggen ob Blatten	Not recorded
	Etang d'y Cor Montana	In expansion
	Etang de Luissel Bex	Continuous
	Gondo Alpjen	Discontinuous
	Grächen See	Not recorded
	Hopschensee	Continuous
	Mittlere Hellelen	In expansion
	Pillon Gsteig-Diablerets	Not recorded
	Wallbach Lenk	Not recorded
	Mont Roux	Not recorded
	Gamperfin	Not recorded
	Creux de Croue	Continuous
	Motta Naluns	Discontinuous
	Praz Rodet	Discontinuous
Italy	Dura-Moor	In expansion
	Malschötscher Hotter	Continuous
	Rinderplatz	Not recorded
	Schwarzsee	In expansion

	Sommersüss	Not recorded
	Dossaccio Bormio	Continuous
	Lac de Lod	Not recorded
	Lac de Villa	Continuous
	Lac du Verney-Dessus	Discontinuous
	Torveraz	Not recorded
	Tourbière de Pilaz	Continuous
	Tourbière de Santa Anna	Not recorded
	Laghi dell'Orgials	Discontinuous
	Lago Piccolo di Avigliana	Continuous
	Ortasee	Discontinuous
	Ortasee II	In expansion
	Refugio Mondovi	Continuous
	Selle di Carnino	Not recorded
	Torbiera del Biecai	Continuous
	Lago della Costa	Continuous
	Lago Padule	Discontinuous
	Colfiorito	In expansion
	Lago Pratignano	Not recorded
	Ospitale	Not recorded
	Pavullo	Discontinuous
	Parma	Discontinuous
	Lago di Martignano	Discontinuous
	Lago di Vico	Continuous
	Lago Albano	Continuous
	Lago di Nemi	Continuous
	Central Adriatic Sea	Continuous
	Salerno Bay	Discontinuous
	Lago Grande di Monticchio LGM	In expansion
	Bradano Valley	In contraction
France	Aronde	Not recorded
	Auneau	Not recorded
	Baie de Seine estuary	Continuous
	Change-Glatinier	Discontinuous
	Coulvain	Not recorded
	La Verderie	Not recorded
	La Vie	Not recorded
	Lavaré	Not recorded
	Le Fourneau	Not recorded
	Lingreville	Not recorded
	Malingue	In expansion
	Marais de Kerdual	Not recorded
	Marais de Lisle	Not recorded
	Marais de Marchesieux	Not recorded
	Mobeche Forest	Not recorded
	Moulin de Thévalles	Not recorded
	Pezou	Not recorded
	Rimarde	Not recorded
	Saint-Ursin	Not recorded
	Serrent	Not recorded
	Grozon	In expansion
	Hières sur Amby	Discontinuous
	Lac Cerin	Continuous
	Lac de Clairvaux	Not recorded
	Lac de Malpas	Discontinuous
	Lake of Annecy	In expansion
	Loras	Continuous
	Lutinière	Discontinuous
	Moselotte	Discontinuous
	Tourbière de Narbief	In expansion
	col du Petit Saint Bernard	Discontinuous
	La Beuffarde	Discontinuous
	Le Grand Lemps	Continuous
	Tourbière de Censeau	Not recorded
	Tourbière du Mou de Pleure (Francia)	Not recorded
	Tourbières des Granges des Chavants	Not recorded
	Ampoix	Not recorded
	Champ Gazon	Not recorded
	Etang Bouquin	Not recorded
	Etang de Cheylade	In contraction
	Etang de la Villetelle	Not recorded
	Lac du Bouchet	Not recorded
	Lac du Mont de Belier	Not recorded
	Le Grand Montarnu	Not recorded
	Le Jolan	Not recorded
	Le Miroir	Not recorded

	Les Chaux de Coudert	Continuous
	Marais de Maurepas	Discontinuous
	Mars	Discontinuous
	Montbé	Discontinuous
	Moulin de Prugnolas	Discontinuous
	Nataloup	Not recorded
	Peyre peat-bog	Not recorded
	Port des Lamberts	Not recorded
	Saint-Benoit-sur-Loire	Continuous
	Sources de l'Yonne	Discontinuous
	Tourbière de Longeyroux	Discontinuous
	Tourbière de Roussy	Discontinuous
	Tourbière des Dauges	Continuous
	La Baforière	Continuous
	Tourbière de Chabannes	Not recorded
	Font Carluze	Not recorded
	Ancenis	Discontinuous
	Basse-Ville	Not recorded
	Bois-Jésus	Not recorded
	Carquefou	Discontinuous
	Caves d'Amont	Not recorded
	Cinq-Mars-la-Pile	Continuous
	Cordemais	Discontinuous
	Corniche de Pail	Not recorded
	Jaunay	Not recorded
	La Bergerie en Charron	Not recorded
	La Caudelais	In expansion
	La Grande Brousse	Discontinuous
	La Grange	Not recorded
	La Prairie du Cassoir	Not recorded
	La boire Torse	In expansion
	Le Gesvres	Not recorded
	Le Marais de la Perge	Not recorded
	Logne	Not recorded
	Marais de Champtocé	Not recorded
	Marais de Mazerolles	Not recorded
	Marais de Munet	Not recorded
	Marais de Méron	Discontinuous
	Marais de la Poupinière	Not recorded
	Marais des Bourbes	Discontinuous
	Oudon	Discontinuous
	Pas du Gu	Not recorded
	Riabelais	Discontinuous
	Saint Viaud Contin	Discontinuous
	Tourbière de Nay	Not recorded
	Tourbière de Parcay-sur-Vienne	Not recorded
	Vertonne	Not recorded
	Correo	Discontinuous
	Embouchac	Discontinuous
	Etang d'Ouveillan	Discontinuous
	Fangeas	Not recorded
	Grand Ratz le Pellet	Not recorded
	Lac Long Inférieur	Not recorded
	Lac Saint Léger	Discontinuous
	Lac de Praver	Continuous
	Lac des Boites	Continuous
	Lac du Lauzon	In contraction
	Lake Racou	Not recorded
	Pelléautier	Not recorded
	Pré Rond	Not recorded
	Sabbion	Not recorded
	Saint Hilaire du Rosier	Discontinuous
	Saint Julien de Ratz	Continuous
	Saint Sixte	In expansion
	Tourbière de Gatimort	Not recorded
	Tourbière de Mont Sec	Not recorded
	Tourbière de Raux	Discontinuous
	Tourbière de la Lande	Not recorded
	Tourbière des Narses Mortes	Discontinuous
	Tourbière du Peschio	Not recorded
Spain	Albufera Alcudia	Discontinuous
	Cala Galdana	Not recorded
	Antas	Not recorded
	Laguna Salada Chiprana	Continuous
	Atxuri	Not recorded
	Cueto de la Avellanosa	Continuous

		Laguna de la Roya	Discontinuous
		PRD-4	Not recorded
		Puerto de Los Tornos	Not recorded
		Saldropo	Not recorded
		Sanabria Marsh	Not recorded
		El Payo	Not recorded
		Lanzahíta	Continuous
		Pataterος bog	Not recorded
		Peña Negra	Discontinuous
		Puerto de Serranillos	Not recorded
		Quintanar de la Sierra	Not recorded
		Salada Pequeña	Not recorded
	Portugal	Charco da Candieira	Not recorded
		Lagoa Comprida 2	Not recorded
		Lagoa Travessa I	Not recorded
	Syria	Bouara	Discontinuous
		Ghab	Not recorded
	Israel	Dead Sea-Ze'elim	Discontinuous
		Birkat Ram	Continuous
		Lake Kinneret	Discontinuous
		Hula Valley	Discontinuous
	Iran	Gomishan	Discontinuous
		Lake Almalou	In expansion
		Lake Zeribar	Discontinuous
		Maharlou Lake	Continuous
	Georgia	Adange	Discontinuous
		Amtkel	Discontinuous
		Gagra	Not recorded
		Lagodekhi	Not recorded
		Sibista	Discontinuous
		Supsa River	Not recorded
		Lake Bazaleti	Continuous
		Imera lake	Continuous
	Albania	Lake Maliq	Not recorded
1.5 – 1 Ka BP			
	Turkey	Lake Manyas	Continuous
		Marmara Sea	Continuous
		Abant Gölü	Continuous
		Beysehir Gölü I	In contraction
		Gölhisar Gölü I	Continuous
		Gölhisar Gölü II	In contraction
		Hoyran Gölü	Continuous
		Kararmik Batakligi	Not recorded
		Köycegiz Gölü	Discontinuous
		Ova Gölü	Continuous
		Pinarbasi	Continuous
		Sögüt Gölü	In contraction
		Sagalassos-Gravgaz	In contraction
		Ladik Gölü	Continuous
		Lake Van	Continuous
	Greece	Kournas	In expansion
		Asi Gonia II	Not recorded
		Asi Gonia I	Not recorded
		Paiko	Continuous
		Lailias	Continuous
		Elatia-Rhodopes	Continuous
		Orestias	Continuous
		Khimaditis III	Continuous
		Vegoritis	Continuous
		Edessa	Continuous
		Litochoro	Continuous
		Lerna	Continuous
		Trikhonis	Continuous
		Ioannina I	Discontinuous
		Ioannina II	Not recorded
		Halos	Not recorded
		Xinias	Continuous
		Voulkaria	Not recorded
		Pertouli	In expansion
		Myrtoon Basin	Continuous
	Bulgary	Beliya Kanton	Continuous
		Kupena II	Not recorded
		Ribno I	Continuous
		Popovo Ezero	Discontinuous
		Mutorog	Continuous
		Trilistnika	Continuous

	Suho Ezero	In expansion
	Sozopol	Continuous
	Arkutino II	Not recorded
	Black Sea South	Discontinuous
	Black Sea Southwest	Discontinuous
	Duranunlak II	Not recorded
	Shabla-Ezeretz	Discontinuous
	Varna I	Not recorded
	Srebarna	Continuous
	Mire Garvan	Continuous
	Osogovo	Continuous
	Begbunar	Continuous
	Sredna Gora	Discontinuous
	Straldzha mire	Not recorded
	Tchokljovo Marsh	Discontinuous
	Vitosha	Not recorded
Romania	Avrig I	Discontinuous
	Avrig II	Not recorded
	Mohos	Not recorded
	Luci	Discontinuous
	Bisoca	Not recorded
	Calinease	Not recorded
	Semenic	Not recorded
	Steregoiu	Not recorded
Hungary	Alsópáhok	Continuous
	Balaton Centre	Continuous
	Balaton Northeast	Continuous
	Balaton Southwest	In expansion
	Nagy-Mohos	Not recorded
	Pölöske	Discontinuous
	Pötréte	Not recorded
	Szigliget	Continuous
Slovakia	Bobrov	Not recorded
	Zlatnicka Dolina	Not recorded
	Strbské pleso	Not recorded
	Tlstá hora	Continuous
Czech Republic	Královec	Discontinuous
	Machová	Discontinuous
	Olbramovice	Not recorded
	Vracov	In expansion
	Palasiny	Not recorded
	Kozli	Discontinuous
	Rezabinec	Not recorded
	Borkovicka blata	Not recorded
	Branna	Discontinuous
	Loucky	Discontinuous
	Chrást	Not recorded
	Velka niva	Not recorded
	Malá niva	Not recorded
	Mrtv8 luh	Not recorded
	Strázenská slat	Not recorded
	Komoranské jezero	Discontinuous
	Dolskym	Discontinuous
	Na bahne	Not recorded
	Velky Ded	Continuous
	Velky Maj	Continuous
	Pancavská louka	Not recorded
	Pryskyricn8 dul	Not recorded
	Tpské raseliniste Mire	Not recorded
	Tpské raseliniste Mire II	Not recorded
	Tpské raseliniste Mire III	Discontinuous
Ukraina	Dovjok Swamp	Discontinuous
	Maly Podleski	Not recorded
	Pecheniya	Discontinuous
	Yukharina Balka	Continuous
Croatia	Bokanjacko	Discontinuous
	Mljet	Continuous
Poland	Bledowo Lake	Not recorded
	Giecz	Discontinuous
	Lake Gosciaz	Not recorded
	Lake Skrzetuszewskie	Discontinuous
	Slawsko	Not recorded
	Swietokrzyskie Lake	Not recorded
	Cergowa Gora	Not recorded
	Godziszewskie Lake	Not recorded
	Lake Mikolajki	Not recorded

	Puscizna Rekowianska	Not recorded
Austria	Buntes Moor	Continuous
	Dortmunder Hütte	Discontinuous
	Franz Senn-Hütte	Continuous
	Egelsee	Continuous
	Gerlos	Continuous
	Giering	Not recorded
	Gradenmoos	Continuous
	Grünau Moor	Discontinuous
	Lindenmoos	Not recorded
	Mieminger See	Continuous
	Moor Alpenrose	Not recorded
	Rotmoos Obergurgl	Not recorded
	Schwarzsee Reschenscheideck	Not recorded
	Schwemm	Continuous
	Seefelder See	In contraction
	Zirbenwaldmoor	Discontinuous
	Wasenmoos beim Zellhof	Not recorded
	Grosses Überling Schattseit-Moor	Not recorded
	Dürrenecksee-Moor	Not recorded
	Fuchsschwanzmoos	Not recorded
	Fuschlsee	Continuous
Germany	Ahlenmoor	Discontinuous
	Ahlequellmoor	Not recorded
	Bruchberg	Discontinuous
	Lüttersee	Discontinuous
	Silberhohl	Discontinuous
	Sonnenberger Moor	Not recorded
	Dunum (Hilliges Moor)	Not recorded
	Felchosee	Not recorded
	Großer Krebssee	Discontinuous
	Löddigsee	Not recorded
	Wachel 3	Not recorded
	Brentenlohe	Not recorded
	Bruckmisse	Not recorded
	Glaswaldsee	In expansion
	Herrenwiesser see	In expansion
	Durchenbergried	Discontinuous
	Feuenried	Discontinuous
	Gaienhofen	Discontinuous
	Hornstaad-Bodensee	Continuous
	Mindelsee	Continuous
	Oberderdingen-Großvillars	Discontinuous
	Steerenmoos	Discontinuous
	Wilder See beim Ruhestein	In expansion
Switzerland	Derrière les Embreux	Not recorded
	Etang de la Gruère	Discontinuous
	Le Loclat	Continuous
	Lobsigensee	Continuous
	Montilier	Continuous
	Aegelsee	Not recorded
	Amsoldingersee	Discontinuous
	Bachalpsee	In expansion
	Hinterburgseeli	Continuous
	Hängstli	Not recorded
	Linden	Discontinuous
	Oberaar	Discontinuous
	Rotsee	Continuous
	Schwarzsee FR	Discontinuous
	Schöpfenwaldmoor	Discontinuous
	Sägistalsee	In expansion
	Süftenenegg	Not recorded
	Aletschwald	Continuous
	Alp Lüsga Belalp 1	Discontinuous
	Alpi di Robièi Val Bavona	Not recorded
	Alpi di Robièi Val Bavona Bodenprofil	Not recorded
	Bitsch-Naters	Continuous
	Eggen ob Blatten	In expansion
	Etang d'y Cor Montana	Continuous
	Etang de Luissel Bex	Continuous
	Gondo Alpjen	In expansion
	Grächen See	In expansion
	Hopschensee	Continuous
	Mittlere Hellelen	Continuous
	Pillon Gsteig-Diablerets	In expansion
	Wallbach Lenk	Continuous

	Mont Roux	Continuous
	Gamperfin	Discontinuous
	Creux de Croue	In contraction
	Motta Naluns	Discontinuous
	Praz Rodet	Discontinuous
Italy	Dura-Moor	Continuous
	Malschötscher Hotter	Continuous
	Rinderplatz	In expansion
	Schwarzsee	Continuous
	Sommersüss	In expansion
	Lac de Lod	Continuous
	Lac de Villa	Continuous
	Lac du Verney-Dessus	Not recorded
	Torveraz	Discontinuous
	Tourbière de Pilaz	Continuous
	Tourbière de Santa Anna	Not recorded
	Laghi dell'Orgials	Not recorded
	Lago Piccolo di Avigliana	Continuous
	Ortasee	Not recorded
	Ortasee II	Continuous
	Refugio Mondovi	Continuous
	Selle di Carnino	Not recorded
	Lago della Costa	Continuous
	Lago Padule	Not recorded
	Colfiorito	Continuous
	Lago Pratignano	Discontinuous
	Ospitale	Discontinuous
	Pavullo	In expansion
	Parma	Discontinuous
	Lago di Martignano	Continuous
	Lago di Vico	Continuous
	Lago Albano	Continuous
	Lago di Nemi	Continuous
	Central Adriatic Sea	Continuous
	Salerno Bay	Discontinuous
	Lago Grande di Monticchio LGM	Continuous
	Bradano Valley	Continuous
France	Aronde	Not recorded
	Auneau	Discontinuous
	Baie de Seine estuary	Continuous
	Change-Glatinier	Discontinuous
	Coulvain	Discontinuous
	Fougères	Not recorded
	La Verderie	Discontinuous
	La Vie	Discontinuous
	Lavaré	Discontinuous
	Le Fourneau	Discontinuous
	Lingreville	Discontinuous
	Malingue	Continuous
	Marais de Kerdual	Discontinuous
	Marais de Lisle	Discontinuous
	Marais de Marchesieux	Discontinuous
	Mobeche Forest	Not recorded
	Moulin de Thévalles	Not recorded
	Pezou	Not recorded
	Rimarde	In expansion
	Saint-Ursin	Not recorded
	Serrent	Not recorded
	Grozon	Continuous
	Hières sur Amby	Not recorded
	Lac Cerin	Continuous
	Lac de Clairvaux	Not recorded
	Lac de Malpas	Not recorded
	Lake of Annecy	Continuous
	Loras	Continuous
	Lutinière	Discontinuous
	Moselotte	Not recorded
	Tourbière de Narbief	Continuous
	col du Petit Saint Bernard	In expansion
	La Beuffarde	In expansion
	Le Grand Lemps	Continuous
	Tourbière de Censeau	Discontinuous
	Tourbière du Mou de Pleure (Francia)	Discontinuous
	Tourbières des Granges des Chavants	Discontinuous
	Champ Gazon	Not recorded
	Etang Bouquin	Discontinuous

Etang de la Villetelle	In expansion
Lac du Bouchet	Not recorded
Lac du Mont de Belier	Not recorded
Le Grand Montarnu	Not recorded
Le Jolan	Not recorded
Le Miroir	Not recorded
Les Chaux de Coudert	Continuous
Les Nans	Continuous
Marais de Maurepas	Discontinuous
Mars	Discontinuous
Montbé	Discontinuous
Moulin de Prugnolas	Not recorded
Nataloup	Discontinuous
Peyre peat-bog	Continuous
Port des Lamberts	In expansion
Quart du Bois	Discontinuous
Sources de l'Yonne	Discontinuous
Tourbière de Longeyroux	Not recorded
Tourbière de Roussy	In expansion
Tourbière des Dauges	Continuous
La Baforière	Continuous
Tourbière de Chabannes	Continuous
Font Carluze	Discontinuous
Ancenis	Not recorded
Basse-Ville	Not recorded
Bois-Jésus	Discontinuous
Carquefou	Discontinuous
Caves d'Amont	Discontinuous
Cinq-Mars-la-Pile	Continuous
Cordemais	In expansion
Corniche de Pail	Continuous
Jaunay	Not recorded
La Bergerie en Charron	In expansion
La Caudelais	Continuous
La Grande Brousse	Discontinuous
La Grange	Discontinuous
La Prairie du Cassoir	Discontinuous
La boire Torse	Continuous
Le Gesvres	Not recorded
Le Marais de la Perge	Discontinuous
Les Naudières	Discontinuous
Logne	Discontinuous
Marais de Champtocé	Not recorded
Marais de Mazerolles	Discontinuous
Marais de Munet	Discontinuous
Marais de Méron	Discontinuous
Marais de la Poupinière	Not recorded
Marais des Bourbes	Not recorded
Oudon	In expansion
Pas du Gu	Discontinuous
Riabelais	Discontinuous
Saint Viaud Contin	Discontinuous
Tourbière de Nay	Discontinuous
Tourbière de Parcay-sur-Vienne	Discontinuous
Vertonne	In expansion
Correo	In expansion
Etang d'Ouveillan	Discontinuous
Fangeas	In expansion
Grand Ratz le Pellet	In expansion
Lac Long Inférieur	Discontinuous
Lac Saint Léger	In expansion
Lac de Praver	Continuous
Lac des Boites	Continuous
Lac du Lauzon	Continuous
Lake Racou	Continuous
Pelléautier	Not recorded
Pré Rond	Not recorded
Sabbion	Discontinuous
Saint Julien de Ratz	Continuous
Saint Sixte	Continuous
Tourbière de Gatimort	Discontinuous
Tourbière de Mont Sec	Not recorded
Tourbière de Raux	In expansion
Tourbière de la Lande	Discontinuous
Tourbière des Narses Mortes	Discontinuous
Tourbière du Peschio	Not recorded

	Spain	Albufera Alcudia (Spain)	Discontinuous
		Cala Galdana (Spain)	Not recorded
		Antas (Spain)	Not recorded
		Laguna Salada Chiprana	Continuous
		Atxuri	Not recorded
		Cueto de la Avellanosa	Continuous
		Laguna de la Roya	Not recorded
		PRD-4	Not recorded
		Posidonia Lligat	Not recorded
		Puerto de Los Tornos	Discontinuous
		Saldropo	Not recorded
		Sanabria Marsh	Discontinuous
		El Payo	Not recorded
		Lanzahíta	Continuous
		Patateros bog	Discontinuous
		Peña Negra	In expansion
		Puerto de Serranillos	Not recorded
		Quintanar de la Sierra	Not recorded
		Salada Pequeña	Not recorded
	Portugal	Charco da Candieira	Discontinuous
		Lagoa Comprida 2	Not recorded
		Lagoa Travessa I	Not recorded
	Syria	Bouara	Discontinuous
		Ghab	Not recorded
	Israel	Dead Sea-Ze'elim (Israel)	In contraction
		Birkat Ram	Continuous
		Lake Kinneret	Discontinuous
		Hula Valley	Discontinuous
	Iran	Gomishan	Not recorded
		Lake Almalou	Continuous
		Maharlou Lake	Continuous
	Georgia	Adange	Not recorded
		Amtkel	Discontinuous
		Gagra	Not recorded
		Khodzal	Not recorded
		Lagodekhi	Not recorded
		Sibista	Discontinuous
		Supsa River	Discontinuous
		Lake Bazaleti	Continuous
		Imera lake	Continuous
	Albania	Lake Maliq	Not recorded
1 – 0.5 Ka BP			
	Turkey	Lake Manyas	Continuous
		Marmara Sea	Continuous
		Abant Gölü	Not recorded
		Beysehir Gölü I	Discontinuous
		Gölhisar Gölü II	Discontinuous
		Hoyran Gölü	In contraction
		Kararmik Batakligi	Not recorded
		Köycegiz Gölü	Discontinuous
		Ova Gölü	Not recorded
		Pinarbasi	Continuous
		Sögüt Gölü	Discontinuous
		Sagalassos-Gravgaz	Discontinuous
		Ladik Gölü	Continuous
		Lake Van	Continuous
	Greece	Kournas	Continuous
		Asi Gonia II	Not recorded
		Asi Gonia I	Discontinuous
		Paiko	Continuous
		Lailias	Continuous
		Elatia-Rhodopes	Continuous
		Orestias	Continuous
		Khimaditis III	Continuous
		Vegoritis	Continuous
		Edessa	Continuous
		Beles Greece)	In expansion
		Litochoro	Continuous
		Lerna	Continuous
		Trikhonis	Continuous
		Ioannina II	In expansion
		Xinias	Continuous
		Voulkaria	In expansion
		Pertouli	Continuous
		Myrtoon Basin	Continuous
	Bulgary	Beliya Kanton	Continuous

	Kupena II	Discontinuous
	Ribno I	Continuous
	Popovo Ezero	Not recorded
	Mutorog	Continuous
	Trilistnika	Continuous
	Suho Ezero	Continuous
	Sozopol	Continuous
	Arkutino II	Discontinuous
	Black Sea South	Not recorded
	Black Sea Southwest	Not recorded
	Duranunlak II	Not recorded
	Shabla-Ezeretz	Discontinuous
	Varna I	Not recorded
	Srebarna	Continuous
	Mire Garvan	Continuous
	Osogovo	Continuous
	Begbunar	Continuous
	Sredna Gora	Not recorded
	Straldzha mire	Not recorded
	Vitosha	Continuous
Romania	Avrig I	Discontinuous
	Avrig II	Discontinuous
	Mohos	Not recorded
	Luci	Discontinuous
	Bisoca	Discontinuous
	Calinease	Not recorded
	Semenic	Discontinuous
	Steregoiu	Not recorded
Hungary	Alsópáhok	Continuous
	Balaton Centre	Continuous
	Balaton Northeast	Continuous
	Balaton Southwest	Continuous
	Nagy-Mohos	Not recorded
	Pölöske	Discontinuous
	Pötréte	Not recorded
	Szigliget	Continuous
Slovakia	Bobrov	Not recorded
	Zlatnicka Dolina	Not recorded
	Strbské pleso	Discontinuous
	Tlstá hora	Continuous
Czech Republic	Královec	Not recorded
	Machová	Discontinuous
	Olbramovice	Not recorded
	Vracov	Continuous
	Palasiny	Not recorded
	Kozli	Not recorded
	Rezabinec	Discontinuous
	Borkovicka blata	Discontinuous
	Branna	Discontinuous
	Loucky	Discontinuous
	Chrást	Not recorded
	Velka niva	Discontinuous
	Malá niva	Not recorded
	Mrtv8 luh	Discontinuous
	Strázenská slat	Discontinuous
	Komoranské jezero	Not recorded
	Dolskym	Not recorded
	Na bahne	Not recorded
	Velky Ded	Continuous
	Velky Maj	Not recorded
	Pancavská louka	Discontinuous
	Pryskyricn8 dul	Not recorded
	Tpské raseliniste Mire	Continuous
	Tpské raseliniste Mire II	Not recorded
	Tpské raseliniste Mire III	Not recorded
Ukraina	Dovjok Swamp	Not recorded
	Maly Podleski	Not recorded
	Pecheniya	Not recorded
	Yukharina Balka	Continuous
Croatia	Bokanjacko	Not recorded
	Mljet	Continuous
Poland	Bledowo Lake	Discontinuous
	Giecz	Not recorded
	Lake Gosciaz	Discontinuous
	Lake Skrzetuszewskie	Discontinuous
	Swietokrzyskie Lake	Discontinuous

	Cergowa Gora	Not recorded
	Godziszewskie Lake	Not recorded
	Lake Mikolajki	Not recorded
	Puscizna Rekowianska	Not recorded
Austria	Buntes Moor	Continuous
	Dortmunder Hütte	Not recorded
	Franz Senn-Hütte	Continuous
	Egelsee	Continuous
	Gerlos	In contraction
	Giering	Discontinuous
	Gradenmoos	Continuous
	Grünau Moor	Discontinuous
	Lindenmoos	In expansion
	Mieminger See	Continuous
	Moor Alpenrose	Not recorded
	Rotmoos Obergurgl	Discontinuous
	Schwarzsee Reschenscheideck	Discontinuous
	Schwemm	In contraction
	Seefelder See	Discontinuous
	Zirbenwaldmoor	Discontinuous
	Wasenmoos beim Zellhof	In expansion
	Grosses Überling Schattseit-Moor	Discontinuous
	Dürrenecksee-Moor	Not recorded
	Fuchsschwanzmoos	Discontinuous
	Fuschlsee	Continuous
Germany	Ahlenmoor	Discontinuous
	Ahlequellmoor	Not recorded
	Bruchberg	Discontinuous
	Lüttersee	Not recorded
	Silberhohl	Discontinuous
	Sonnenberger Moor	Discontinuous
	Dunum (Hilliges Moor)	Discontinuous
	Felchosee	Discontinuous
	Großer Krebssee	Discontinuous
	Löddigsee	Discontinuous
	Wachel 3	Not recorded
	Brentenlohe	Not recorded
	Bruckmisse	Not recorded
	Glaswaldsee	Continuous
	Herrenwiesser see	Continuous
	Durchenbergried	Discontinuous
	Feuenried	Discontinuous
	Gaienhofen	Not recorded
	Hornstaad-Bodensee	Continuous
	Mindelsee	Continuous
	Oberderdingen-Großvillars	Discontinuous
	Steerenmoos	Not recorded
	Wilder See beim Ruhestein	Continuous
Switzerland	Derrière les Embreux	Continuous
	Etang de la Gruère	Discontinuous
	Le Loclat	Continuous
	Lobsigensee	Continuous
	Montilier	Continuous
	Aegelsee	Not recorded
	Amsoldingersee	Discontinuous
	Bachalpsee	Continuous
	Hinterburgseeli	Continuous
	Hängstli	Not recorded
	Linden	In expansion
	Oberaar	Discontinuous
	Rotsee	Continuous
	Schwarzsee FR	In expansion
	Schöpfenwaldmoor	Discontinuous
	Sägistalsee	Continuous
	Süftenenegg	In expansion
	Trogenmoos	Discontinuous
	Aletschwald	Continuous
	Alp Lüsga Belalp 1	Discontinuous
	Alpi di Robièi Val Bavona	Discontinuous
	Alpi di Robièi Val Bavona Bodenprofil	Not recorded
	Bitsch-Naters	Continuous
	Eggen ob Blatten	Continuous
	Etang d'y Cor Montana	Continuous
	Etang de Luissel Bex	Continuous
	Gondo Alpjen	Continuous
	Grächen See	In contraction

	Hopschensee	Continuous
	Mittlere Hellelen	Continuous
	Pillon Gsteig-Diablerets	Continuous
	Wallbach Lenk	Continuous
	Gamperfin	Discontinuous
	Creux de Croue	Discontinuous
	Praz Rodet	Discontinuous
Italy	Dura-Moor	Continuous
	Malschötscher Hotter	Continuous
	Rinderplatz	Continuous
	Schwarzsee	Continuous
	Sommersüss	Continuous
	Lac de Lod	Continuous
	Lac de Villa	Continuous
	Lac du Verney-Dessus	Not recorded
	Torveraz	Not recorded
	Tourbière de Pilaz	Continuous
	Tourbière de Santa Anna	Discontinuous
	Laghi dell'Orgials	Not recorded
	Lago Piccolo di Avigliana	Continuous
	Ortasee	Discontinuous
	Ortasee II	Continuous
	Selle di Carnino	In expansion
	Lago della Costa	Continuous
	Lago Padule	Not recorded
	Colfiorito	Continuous
	Ospitale	Not recorded
	Pavullo	Continuous
	Parma	Discontinuous
	Lago di Martignano	Discontinuous
	Lago di Vico	Continuous
	Lago Albano	Continuous
	Lago di Nemi	Continuous
	Central Adriatic Sea	Continuous
	Salerno Bay	Discontinuous
	Lago Grande di Monticchio LGM	Continuous
	Bradano Valley	Discontinuous
France	Aronde	Discontinuous
	Auneau	Discontinuous
	Baie de Seine estuary	Continuous
	Change-Glatinier	In expansion
	Coulvain	Discontinuous
	Fougères	Discontinuous
	La Verderie	In expansion
	La Vie	Not recorded
	Lavaré	Discontinuous
	Le Fourneau	Discontinuous
	Lingreville	In expansion
	Malingue	Continuous
	Marais de Kerdual	Discontinuous
	Marais de Lisle	In expansion
	Marais de Marchesieux	Not recorded
	Mobeche Forest	Discontinuous
	Moulin de Thévalles	In expansion
	Pezou	In expansion
	Rimarde	In contraction
	Saint-Ursin	Discontinuous
	Serrent	Discontinuous
	Hières sur Amby	Not recorded
	Lac Cerin	Continuous
	Lac de Clairvaux	In expansion
	Lac de Malpas	In expansion
	Lake of Annecy	Continuous
	Loras	Continuous
	Lutinière	In expansion
	Moselotte	In expansion
	Tourbière de Narbief	In contraction
	col du Petit Saint Bernard	Continuous
	La Beuffarde	Continuous
	Le Grand Lemps	Continuous
	Tourbière de Censeau	In expansion
	Tourbière du Mou de Pleure (Francia)	In expansion
	Tourbières des Granges des Chavants	In expansion
	Champ Gazon	In expansion
	Etang Bouquin	In expansion
	Etang de la Villetelle	Continuous

	Lac du Bouchet	Continuous
	Lac du Mont de Belier	Not recorded
	Le Grand Montarnu	Continuous
	Le Jolan	Continuous
	Le Miroir	Discontinuous
	Les Cars	Discontinuous
	Les Chaux de Coudert	Continuous
	Les Nans	Continuous
	Marais de Maurepas	In expansion
	Mars	In expansion
	Montbé	In expansion
	Moulin de Prugnolas	Discontinuous
	Nataloup	Discontinuous
	Peyre peat-bog	Continuous
	Port des Lamberts	Continuous
	Quart du Bois	Discontinuous
	Sources de l'Yonne	In expansion
	Tourbière de Longeyroux	Not recorded
	Tourbière de Roussy	Continuous
	Tourbière des Dauges	Continuous
	La Baforière	Continuous
	Tourbière de Chabannes	Continuous
	Font Carluze	Discontinuous
	Ancenis	Not recorded
	Basse-Ville	In expansion
	Bois-Jésus	Discontinuous
	Carquefou	Discontinuous
	Caves d'Amont	In expansion
	Changeon	Continuous
	Cinq-Mars-la-Pile	Continuous
	Corniche de Pail	Continuous
	Jaunay	Discontinuous
	La Grange	Not recorded
	La Prairie du Cassoir	In expansion
	La boire Torse	Continuous
	Le Gesvres	In expansion
	Le Marais de la Perge	Discontinuous
	Les Naudières	Discontinuous
	Logne	Discontinuous
	Marais de Champtocé	In expansion
	Marais de Mazerolles	In expansion
	Marais de Munet	In expansion
	Marais de Méron	In expansion
	Marais de la Poupinière	Discontinuous
	Marais des Bourbes	Not recorded
	Oudon	Continuous
	Pas du Gu	Discontinuous
	Riabelais	Not recorded
	Saint Viaud Contin	Discontinuous
	Tourbière de Nay	In expansion
	Vertonne	Continuous
	Correo	Discontinuous
	Etang d'Ouveillan	Discontinuous
	Fangeas	Continuous
	Grand Ratz le Pellet	Continuous
	Lac de Praver	Continuous
	Lac des Boites	Continuous
	Lake Racou	Continuous
	Marais de Charauze	In expansion
	Pelléautier	In expansion
	Pré Rond	Discontinuous
	Sabbion	Discontinuous
	Saint Julien de Ratz	Continuous
	Saint Sixte	Continuous
	Tourbière de Gatimort	In expansion
	Tourbière de Mont Sec	In expansion
	Tourbière de Raux	Continuous
	Tourbière de la Lande	In expansion
	Tourbière des Narses Mortes	Discontinuous
	Tourbière du Peschio	In expansion
Spain	Albufera Alcudia	Discontinuous
	Cala Galdana	Not recorded
	Delta del Rio Besos	Discontinuous
	Laguna Salada Chiprana	In contraction
	Atxuri	Discontinuous
	Cueto de la Avellanosa	Continuous

		Laguna de la Roya	In expansion
		PRD-4	Not recorded
		Posidonia Lligat	Discontinuous
		Puerto de Los Tornos	In expansion
		Saldropo	In expansion
		Sanabria Marsh	Not recorded
		El Payo	Not recorded
		Lanzahíta	Continuous
		Patateros bog	Not recorded
		Peña Negra	Continuous
		Puerto de Serranillos	Not recorded
		Quintanar de la Sierra	Discontinuous
		Salada Pequeña	Not recorded
	Portugal	Charco da Candieira	Discontinuous
		Lagoa Comprida 2	Discontinuous
		Lagoa Travessa I	Not recorded
	Syria	Bouara	Not recorded
		Ghab	Not recorded
	Israel	Dead Sea-Ze'elim	Discontinuous
		Birkat Ram	Continuous
		Lake Kinneret	Discontinuous
		Hula Valley	Not recorded
	Iran	Gomishan	Not recorded
		Lake Almalou	Continuous
		Maharlou Lake	Continuous
	Georgia	Adange	Discontinuous
		Amtkel	Discontinuous
		Gagra	Not recorded
		Khodzal	Not recorded
		Lagodekhi	Not recorded
		Sibista	Discontinuous
		Supsa River	Discontinuous
		Lake Bazaleti	Continuous
		Imera lake	Continuous
	Albania	Lake Maliq	Discontinuous
0.5 – 0 Ka BP			
	Turkey	Lake Manyas	Continuous
		Marmara Sea	Continuous
		Beysehir Gölü I	Discontinuous
		Gölhisar Gölü II	Discontinuous
		Hoyran Gölü	Discontinuous
		Kararmik Batakligi	Discontinuous
		Köycegiz Gölü	Discontinuous
		Sögüt Gölü	Discontinuous
		Sagalassos-Gravgaz	Discontinuous
		Lake Van	Continuous
	Greece	Kournas	Continuous
		Asi Gonia II	Discontinuous
		Asi Gonia I	Discontinuous
		Paiko	Continuous
		Lailias	In contraction
		Elatia-Rhodopes	Continuous
		Orestias	Continuous
		Khimaditis III	Continuous
		Vegoritis	Continuous
		Edessa	Continuous
		Beles Greece)	Continuous
		Litochoro	Continuous
		Lerna	In contraction
		Trikhonis	Continuous
		Xinias	Continuous
		Voulkaria	In contraction
		Pertouli	Continuous
		Myrtoon Basin	Continuous
	Bulgary	Beliya Kanton	Continuous
		Kupena II	Not recorded
		Ribno I	Continuous
		Mutorog	Continuous
		Trilistnika	Continuous
		Suho Ezero	Continuous
		Sozopol	Continuous
		Black Sea South	Not recorded
		Black Sea Southwest	Discontinuous
		Duranunlak II	Not recorded
		Srebarna	Continuous
		Mire Garvan	Continuous

	Osogovo	Continuous
	Begbunar	Continuous
	Sredna Gora	In expansion
	Straldzha mire	Not recorded
	Vitosha	Continuous
Romania	Avrig I	Discontinuous
	Avrig II	Discontinuous
	Mohos	In expansion
	Luci	Discontinuous
	Bisoca	In expansion
	Calinease	Continuous
	Semenic	Discontinuous
	Steregoiu	Discontinuous
Hungary	Alsópáhok	Continuous
	Balaton Centre	Continuous
	Balaton Northeast	Continuous
	Balaton Southwest	Continuous
	Nagy-Mohos	Not recorded
Slovakia	Bobrov	Discontinuous
	Zlatnicka Dolina	Discontinuous
	Strbské pleso	Discontinuous
	Tlstá hora	Continuous
Czech Republic	Královec	In expansion
	Machová	In expansion
	Palasiny	Discontinuous
	Kozli	Not recorded
	Rezabinec	Not recorded
	Borkovicka blata	In expansion
	Branna	Not recorded
	Loucky	Discontinuous
	Chrást	Not recorded
	Velka niva	Not recorded
	Malá niva	Discontinuous
	Mrtv8 luh	Discontinuous
	Strázenská slat	Not recorded
	Komoranské jezero	Discontinuous
	Dolskym	Discontinuous
	Na bahne	Discontinuous
	Velky Maj	Discontinuous
	Pancavská louka	Discontinuous
	Pryskyricn8 dul	Not recorded
	Tpské raseliniste Mire	In contraction
	Tpské raseliniste Mire II	Not recorded
	Tpské raseliniste Mire III	In expansion
Ukraina	Dovjok Swamp	Discontinuous
	Maly Podleski	Not recorded
	Pecheniya	Not recorded
	Yukharina Balka	Continuous
Croatia	Mljet	Continuous
Poland	Bledowo Lake	Discontinuous
	Giecz	Not recorded
	Lake Gosciaz	Discontinuous
	Swietokrzyskie Lake	In expansion
	Cergowa Gora	Discontinuous
	Godziszewskie Lake	Discontinuous
	Lake Mikolajki	Discontinuous
	Puscizna Rekowianska	Continuous
Austria	Buntes Moor	Continuous
	Dortmunder Hütte	Discontinuous
	Franz Senn-Hütte	Continuous
	Egelsee	Continuous
	Gerlos	Discontinuous
	Giering	Discontinuous
	Gradenmoos	Continuous
	Grünau Moor	Discontinuous
	Mieminger See	In contraction
	Moor Alpenrose	Discontinuous
	Rotmoos Obergurgl	In expansion
	Schwarzsee Reschenscheideck	Discontinuous
	Schwemm	Discontinuous
	Seefelder See	Discontinuous
	Zirbenwaldmoor	In expansion
	Wasenmoos beim Zellhof	In contraction
	Grosses Überling Schattseit-Moor	Not recorded
	Dürrenecksee-Moor	Not recorded
	Fuchsschwanzmoos	Not recorded

	Fuschlsee	Continuous
Germany	Ahlequellmoor	Discontinuous
	Bruchberg	Discontinuous
	Lüttersee	In expansion
	Silberhohl	Discontinuous
	Sonnenberger Moor	Discontinuous
	Dunum (Hilliges Moor)	Discontinuous
	Großer Krebssee	Discontinuous
	Löddigsee	Discontinuous
	Wachel 3	Continuous
	Brentenlohe	In expansion
	Glaswaldsee	Continuous
	Herrenwiesser see	Continuous
	Durchenbergried	Discontinuous
	Feuenried	In expansion
	Gaienhofen	Discontinuous
	Hornstaad-Bodensee	Continuous
	Mindelsee	Continuous
	Oberderdingen-Großvillars	Not recorded
	Steerenmoos	Not recorded
Switzerland	Derrière les Embreux	Continuous
	Le Loclat	Continuous
	Aegelsee	Discontinuous
	Bachalpsee	Continuous
	Hinterburgseeli	Continuous
	Hängstli	Not recorded
	Oberaar	Discontinuous
	Rotsee	Continuous
	Schwarzsee FR	Continuous
	Schöpfenwaldmoor	In expansion
	Sägistalsee	Continuous
	Süftenenegg	In expansion
	Trogenmoos	In expansion
	Aletschwald	Continuous
	Alp Lüsga Belalp 1	Discontinuous
	Alpi di Robièi Val Bavona	In expansion
	Alpi di Robièi Val Bavona Bodenprofil	Discontinuous
	Bitsch-Naters	Continuous
	Eggen ob Blatten	Continuous
	Etang d'y Cor Montana	Continuous
	Etang de Luissel Bex	Continuous
	Gondo Alpjen	Continuous
	Grächen See	Discontinuous
	Hopschensee	Continuous
	Mittlere Hellelen	Continuous
	Wallbach Lenk	Continuous
	Gamperfin	Discontinuous
	Praz Rodet	In expansion
	Dura-Moor (Italy)	Continuous
	Malschötscher Hotter (Italy)	Continuous
	Rinderplatz (Italy)	Continuous
	Schwarzsee (Italy)	Continuous
	Sommersüss (Italy)	Continuous
	Lac de Lod (Italy)	Continuous
	Lac de Villa (Italy)	Continuous
Italy	Lac du Verney-Dessus	Discontinuous
	Torveraz	Not recorded
	Tourbière de Pilaz	Continuous
	Tourbière de Santa Anna	Discontinuous
	Laghi dell'Orgials	Discontinuous
	Lago Grande di Avigliana	Continuous
	Lago Piccolo di Avigliana	Continuous
	Ortasee II	Continuous
	Selle di Carnino	Continuous
	Lago della Costa	Continuous
	Lago Padule	Discontinuous
	Colfiorito	In contraction
	Ospitale	Not recorded
	Pavullo	Continuous
	Parma	Discontinuous
	Lago di Martignano	Not recorded
	Lago di Vico	Continuous
	Lago Albano	Continuous
	Lago di Nemi	Continuous
	Central Adriatic Sea	Continuous
	Salerno Bay	Discontinuous

	Lago Grande di Monticchio LGM	Continuous
France	Aronde	Discontinuous
	Change-Glatinier	In contraction
	Coulvain	Discontinuous
	Fougères	Discontinuous
	La Verderie	Continuous
	Lavaré	Discontinuous
	Le Fourneau	Not recorded
	Malingue	Continuous
	Marais de Kerdual	Not recorded
	Marais de Lisle	Continuous
	Marais de Marchesieux	Not recorded
	Mobeche Forest	Not recorded
	Moulin de Thévalles	Continuous
	Pezou	In contraction
	Saint-Ursin	Not recorded
	Serrent	Discontinuous
	Hières sur Amby	Not recorded
	Lac de Clairvaux	Continuous
	Lac de Malpas	In contraction
	Lake of Annecy	Continuous
	Loras	Continuous
	Moselotte	In contraction
	Tourbière de Narbief	Not recorded
	col du Petit Saint Bernard	In contraction
	La Beuffarde	In contraction
	Le Grand Lemps	Continuous
	Tourbière de Censeau	In contraction
	Tourbière du Mou de Pleure (Francia)	In contraction
	Tourbières des Granges des Chavants	Continuous
	Champ Gazon	Continuous
	Etang Bouquin	Continuous
	Etang de la Villetelle	Continuous
	Lac du Bouchet	Continuous
	Lac du Mont de Belier	Discontinuous
	Le Grand Montarnu	Continuous
	Les Cars	Discontinuous
	Les Chaux de Coudert	Continuous
	Les Nans	Continuous
	Marais de Maurepas	Continuous
	Mars	Continuous
	Montbé	In contraction
	Moulin de Prugnolas	Discontinuous
	Nataloup	In expansion
	Peyre peat-bog	In contraction
	Port des Lamberts	Continuous
	Quart du Bois	In expansion
	Sources de l'Yonne	In contraction
	Tourbière de Longeyroux	Not recorded
	Tourbière de Roussy	Continuous
	Tourbière des Dauges	In contraction
	La Baforière	Continuous
	Tourbière de Chabannes	In contraction
	Font Carluze	Discontinuous
	Basse-Ville	Continuous
	Bois-Jésus	Not recorded
	Carquefou	In expansion
	Caves d'Amont	Discontinuous
	Changeon	Continuous
	Cinq-Mars-la-Pile	In contraction
	Jaunay	In expansion
	La boire Torse	Continuous
	Le Gesvres	Continuous
	Le Marais de la Perge	Discontinuous
	Les Naudières	Discontinuous
	Logne	Discontinuous
	Marais de Champtocé	Continuous
	Marais de Mazerolles	Continuous
	Marais de Munet	Continuous
	Marais de Méron	Continuous
	Marais de la Poupinière	Discontinuous
	Marais des Bourbes	In expansion
	Oudon	In contraction
	Pas du Gu	Discontinuous
	Riabelais	Not recorded
	Saint Viaud Contin	Discontinuous

	Tourbière de Nay	In contraction
	Vertonne	Continuous
	Etang d'Ouveillan	Not recorded
	Grand Ratz le Pellet	Continuous
	Lac de Praver	Continuous
	Lac des Boites	Continuous
	Lake Racou	Continuous
	Marais de Charauze	Continuous
	Pelléautier	Continuous
	Pré Rond	In expansion
	Saint Sixte	Continuous
	Tourbière de Gatimort	Continuous
	Tourbière de Mont Sec	Continuous
	Tourbière de Raux	In contraction
	Tourbière de la Lande	Continuous
	Tourbière des Narses Mortes	Discontinuous
	Tourbière du Peschio	Continuous
Spain	Albufera Alcudia	Discontinuous
	Cala Galdana	Not recorded
	Delta del Rio Besos	Discontinuous
	Laguna Salada Chiprana	Discontinuous
	Atxuri	Not recorded
	Cueto de la Avellanosa	Continuous
	Laguna de la Roya	Continuous
	PRD-4	Not recorded
	Posidonia Lligat	Discontinuous
	Puerto de Los Tornos	Continuous
	Sanabria Marsh	Discontinuous
	El Payo	Not recorded
	Lanzahíta	Continuous
	Patateros bog	Discontinuous
	Peña Negra	Continuous
	Puerto de Serranillos	Discontinuous
	Quintanar de la Sierra	Discontinuous
	Salada Pequeña	Not recorded
	Turbera de La Panera Cabras	Discontinuous
Portugal	Charco da Candieira	Discontinuous
	Lagoa Comprida 2	Discontinuous
	Lagoa Travessa I	Not recorded
Syria	Bouara	Discontinuous
	Birkat Ram (Israel)	Continuous
	Lake Kinneret (Israel)	Discontinuous
Israel	Carme Coastal Plain (Israel)	Not recorded
Iran	Gomishan	Not recorded
	Lake Almalou	Continuous
	Maharlou Lake	Continuous
Georgia	Adange	Not recorded
	Amtkel	Discontinuous
	Khodzal	Discontinuous
	Lagodekhi	Discontinuous
	Sibista	Discontinuous
	Supsa River	Discontinuous
	Lake Bazaleti	Continuous
	Imera lake	Continuous
Albania	Lake Maliq (Albania)	Not recorded

[a] The fossil pollen data have been extracted from the existing European Pollen Database (EPD; http://www.ncdc.noaa.gov/paleo/epd) and from 38 pollen diagrams published in scientific journals until September 2016. We downloaded all suitable pollen data from 412 Eurasian sites (unrestricted EPD data) in order to detect the discontinuous and continuous occurrence of *Juglans*-type fossil pollen before (1700 -11.923 Ka BP) and during the Holocene in Europe and Western Asia. Restricted pollen spectra or pollen data without calibrated radiocarbon timescale were excluded from our study.

[b] Despite being produced in large quantities, *Juglans*-type fossil pollen is poorly dispersed. Pollen values above 2% indicate a relative high density of walnut trees near the sampling site [240]. Although possible contaminations due to re-deposition of Juglandaceae pollen from the Tertiary deposits have been detected in the recent sediments of Crimea (Ukraine) [241] and Western Caucasus [242], the long-distance transport of walnut pollen is considered rather unlikely. Therefore, we chose to map the occurrence of *Juglans*-type fossil pollen using the

following discrete classification. The presence/absence of *Juglans*-type fossil pollen into fifteen distinct time intervals before (early presence) and during the Holocene and the subsequent pollen classification were reported for each selected site: Continuous = pollen grains occurred regularly and abundantly (Red); Discontinuous = at least one single pollen grain or few pollen grains discontinuously distributed (Blue); In expansion = transition from discontinuous to continuous occurrence of pollen grains (Orange); In contraction = transition from continuous to discontinuous occurrence of pollen grains (Green); Not recorded = absence of pollen grains (Gray). Fifteen geo-referenced distribution maps were drawn with ArcGIS 9.3 software (ESRI, Redlands, Calif. USA) and assembled in S1 Video.

References

1. Cordova CE, Harrison SP, Mudie PJ, Riehl S, Leroy SAG, Ortiz N. Pollen, plant macrofossil and charcoal records for palaeovegetation reconstruction in the Mediterranean-Black Sea Corridor since the Last Glacial Maximum. Quatern Int. 2009; 197: 12-26.

2. Bottema S. The Holocene history of walnut, sweet-chestnut, manna-ash and plane tree in the Eastern Mediterranean. Pallas. 2000; 52: 35-59.

3. Eastwood WJ, Roberts N, Lamb HF, Tibby JC. Holocene environmental change in southwest Turkey: a palaeoecological record of lake and catchment-related changes. Quaternary Sci Rev. 1999; 18: 671-695.

4. Van Zeist, W, Woldring H, Stapert D. Late quaternary vegetation and climate of southwestern Turkey. Palaeohistoria. 1975; 17: 53-144.

5. Vermoere M, Vanhecke L, Waelkens M, Smets E. Modern pollen studies in the territory of Sagalassos (Southwest Turkey) and their use in the interpretation of a Late Holocene pollen diagram. Rev Palaeobot Palynol. 2001; 114: 29-56.

6. Van Zeist W, Woldring H. A postglacial pollen diagram from Lake Van in east Anatolia. Rev Palaeobot Palynol. 1978; 26: 249-276.

7. Bottema S, Sarpaki A. Environmental change in Crete: a 9000-year record of Holocene vegetation history and the effect of the Santorini eruption. Holocene. 2003; 13: 733-749.

8. Atherden MA, Hall JA. Human impact on vegetation in the White Mountains of Crete since AD 500. Holocene. 1999; 9: 183-193.
9. Gerasimidis A, Athanasiadis N. Woodland history of northern Greece from the mid Holocene to recent time based on evidence from peat pollen profiles. Veg Hist Archaebot. 1995; 4: 109-116.
10. Kouli K, Dermitzakis MD. Contributions to the European Pollen Database. 11. Lake Orestiás (Kastoria, northern Greece). Grana. 2010; 49: 154-156.
11. Bottema S. Palynological investigations in Greece with special reference to pollen as indicator of human activity. Palaeohistoria. 1982; 24: 257-289.
12. Athanasiadis N, Gerasimidis A, Panajiotidis S. A palynological study in the Beles Mountains, Northern Greece. "Aspects of Palynology and Palaeoecology" Festschrift in hinour of Elissaveta Bozilova. PENSOFT, Sofia-Moscow; 2003. pp. 185-197.
13. Athanasiadis N. Zur postglazialen Vegetationsentwicklung von Litochoro Katarinis und Pertouli Trikalon (Griechenland). Flora. 1975; 164: 99-132.
14. Turner J, Greig JRA. Some Holocene pollen diagrams from Greece. Rev Palaeobot Palynol. 1975; 20: 171-204.
15. Jahns S. On the Holocene history of the Argive plain (Peloponese, southern Greece). Veg Hist Archaebot. 1993; 2: 187-203.
16. Bottema S. Pollen analytical investigations in Thessaly . Palaeohistoria. 1979; 21: 19-40.
17. Jahns S. The Holocene history of vegetation and settlement at the coastal site of Lake Voulkaria in Acarnania, western Greece. Veg Hist Archaebot. 2005; 14: 55-66.
18. Lazarova MA, Tonkov S, Marinova E, Ivanov D, Bozilova EDB. Contributions to the European Pollen Database. 12. Western Rhodopes Mountains (Bulgaria): peat bog Beliya Kanton. Grana. 2011; 50: 162-164.

19. Huttunen A, Huttunen RL, Vasari Y, Panovska HIP, Bozilova EDB. Late Glacial and Holocene history of flora and vegetation in the Western Rhodopes Mountains Bulgaria. Acta Bot Fennica. 1992; 144: 63-80.

20. Tonkov S, Panovska H, Possnet G, Bozilova E. The Holocene vegetation history of Northern Pirin Mountain, southwestern Bulgaria: pollen analysis and radiocarbon dating of a core from Lake Ribno Banderishko. Holocene. 2002; 12: 201-210.

21. Stefanova I, Bozilova EDB. Studies on the Holocene history of vegetation in the Northern Pirin Mountains, southwestern Bulgaria. In: Bozilova E, Tonkov S, editors. Advances in Holocene Palaeoecology in Bulgaria. Sofia, Bulgaria: Pensoft Publications; 1995. pp. 9-31.

22. Panovska HIP, Bozilova EDB, Tonkov S. A palaeoecological investigation on the vegetation history in the Southern Pirin Mountains (southwestern Bulgaria). In: Bozilova E, Tonkov S, editors. Advances in Holocene Palaeoecology in Bulgaria. Sofia, Bulgaria: Pensoft Publications; 1995; pp 32-46.

23. Marinova E, Tonkov S, Bozilova E, Vajsor I. Holocene anthropogenic landscapes in the Balkans: the palaeobotanic evidence from southwestern Bulgaria. Veget Hist Archaeobot. 2012; 21: 413–427.

24. Tonkov S, Atanassova JR, Bozilova EDB, Skog G. A Late Holocene pollen diagram from Suho Ezero, Rila Monastery area (Central Rila Mountains, southwestern Bulgaria). Phytol Balcan. 1998; 4:31-38.

25. Marinova-Filipova M, Giosan L, Angelova H, Preisinger A, Pavlov D, Vergiev S. Palaeoecology of Submerged prehistoric settlements in Sozopol Harbour, Bulgaria. In: Benjamin J, Bonsall C, Pickard C, Fischer A, editors. Submerged Prehistory. Oxford, United Kingdom: Oxbow Books; 2011. pp. 230-244.

26. Bozilova EDB, Beug HJ. On the Holocene history of vegetation in SE Bulgaria (Lake Arkutino, Ropotamo region). Veget Hist Archaeobot. 1992; 1: 19-32.

27. Shopov VS, Bozilova EDB, Atanassova JR. Biostratigraphy and radiocarbon data of upper quaternary sediments from the western part of the Black Sea. Geol Balc. 1992; 22: 59-70.
28. Atanassova JR. Dinoflagelate cysts of Late Quaternary and recent sediments from the western Black sea. Annual of Sofia University, Faculty of Biology. 1995; 87: 17-28.
29. Bozilova EDB, Filipova MVFM. Paleoecological environment in northeastern Black Sea area during Neolithic, Eneolithic and Bronze periods. Stud Praehist. 1986; 8: 160-165.
30. Bozilova EDB, Lazarova MA, Straszewska K.. Geomorphological characteristics and development of the vegetation in the region of Srebarna Lake Bulgaria (in Bulgarian). Godishnik na Sofiiskiya Universitet "Kliment Okhridski" Biologicheski Fakultet. 1985; 79: 99-109.
31. Lazarova MA. Human impact on the natural vegetation in the region of Lake Srebarna and mire Garvan (northeastern Bulgaria). Palynological and Palaeoethnobotanical evidence. In: Bozilova EDB, Tonkov S, editors. Advances in Holocene Palaeoecology in Bulgaria. Sofia-Moscow: Pensoft Publication; 1995. pp. 47-67.
32. Tonkov S, Bozilova EDB. Pollen analysis of peat bog in Maleshevska mountain (SW Bulgaria). Annual of Sofia University, Faculty of Biology 1992; 81: 11-21.
33. Lazarova MA, Tonkov S, Snowball I, Marinova E. Contributions to the European Pollen Database. 6. Peat-bog Begubar (Osogovo Mountains, south-west Bulgaia): Four millennia of vegetation history. Grana. 2009; 48: 147-149.
34. Petrov SI, Filipovitch L. Postglacial changes of the vegetation on the slopes of Sredna Gora mountain. Proceedings Nat. Conf. of Botany (Bulgaria). 1987; 1: 339-406.
35. Tonkov S, Bozilova EDB, Marinova E, Jungner H. History of vegetation and landscape during the last 4000 years in the area of Straldzha mire (southeastern Bulgaria). Phytol Balcan. 2008; 14: 185-191.

36. Bozilova EDB, Tonkov S. Vegetational development in the mountainous areas of SW Bulgaria. I. Palynological investigation and reconstruction of past vegetation. Ecol Mediterr. 1985; 9: 33-37.

37. Filipovitch L. Palynological studies of peat bogs on the southern slopes of Vitosha mountain. For Sci. 1985; 2: 3-16.

38. Tantau I, Reille M, de Beaulieu JL, Farcas S. Late Glacial and Holocene vegetation history in the southern part of Transylvania : pollen analysis of two sequences from Avrig. J Quat Sci. 2006; 21: 49-61.

39. Tantau I, Reille M, de Beaulieu JL, Farcas S, Goslar T, Paterne M. Vegetation history in the Eastern Romanian Carparthians: pollen analysis of the two sequences from the Mohos crater. Veg Hist Archaebot. 20013; 12:113-125.

40. Tantau I, Feurdean A, De Beaulieu JL, Reille M, Farcas S. Vegetation sensitivity to climate changes and human impact in the Harghita Mountains (Eastern Romanian Carpathians) over the past 15000 years. J. Quat Sci. 2014; 29: 141-152.

41. Tantau I, Reille M, de Beaulieu JL, Farcas S, Brewer S. Holocene vegetation history in Romanian Subcarpathians. Quat Res. 2009; 72: 164-173.

42. Feurdean AN, Willis KJ, Astalos C. Legacy of the past land-use changes and management on the 'natural' upland forest composition in the Apuseni Natural Park, Romania. Holocene. 2009; 19: 967-981.

43. Rösch M., Fischer E. A radiocarbon dated Holocene pollen profile from the Banat mountains (Southwestern Carpathians, Romania). Flora. 2000; 195: 277-286.

44. Björkman, L., A. Feurdean, and B. Wohlfarth. Late- Glacial and Holocene forest dynamics at Steregoiu in the Gutaiului Mountains, Northwest Romania. Rev Palaeobot Palynol. 2003; 124: 79-111.

45. Zatykó CS, Juhász I, Sümegi P. Environmental archaeology in Transdanubia. Budapest: Archaeological Institute of the Hungarian Academy of Sciences. VAH. 2007; 1: 20: 391.

46. Juhász I. Reconstitution palynologique de la végétation depuis le Tardiglaciaire dans la région de Zala, sud-est de la Hongrie. [Palynological Reconstruction of the Lateglacial and Holocene Vegetation in South-west Hungary]. PhD Dissertation in Biosciences of Environment (Palynology), University of Aix-Marseille III (Marseille, France) and University of Pécs (Pécs, Hungary); 2002.

47. Juhász I, Drescher-Schneider RE, Andrieu-Ponel V, de Beaulieu JL. Anthropogenic indicators in a palynological record from Pölöske, Zala Region, Western Hungary. Universitätsforschungen zur Prähistorischen Archäologie Bonn. 2001: 78: 29-38.

48. Rybnícek K, Rybnícková E. A palaecological reconstruction of precultural vegetation in the intermontane basins of the western Carpathians. Ecol. Medit. 1985; 11: 27-31.

49. Rybnícková E, Rybnícek K. Pollen and macroscopic analyses of sediments from two lakes in the High Tatra mountains, Slovakia. Veg Hist Archaebot. 2003; 15: 345-356.

50. Rybnícek K, Rybnícková E. Upper Holocene dry land vegetation in the Moravian-Slovakian borderland (Czech and Slovak Republics). Veg Hist Archaebot. 2008; 17: 701-711.

51. Dolakova N, Roszkova A, Prichystal A. Palynology and natural environment in the Pannonian to Holocene sediments of the Early Medieval centre Pohansko near Breclav . J Archaeol Sci. 2010; 37: 2538-2550.

52. Svobodová H. The development of the southern Moravian vegetation in the Late Glacial and Holocene [in Czech]. Master Thesis, Institute of Botany Pruhonice, Czech Republic. 1992.

53. Jankovská V. The evolution of Late-Glacial and Holocene vegetation in the vicinity of Svetla nad Sazavou (in the western Forland of the Bohemian-Moravian uplands). Folia Geobot Phytotaxon. 1989; 24: 337-448.

54. Rybnícek K, Rybnícková E. The history of flora and vegetation on the Blato mire in southeast Bohemia (palaeoecological study). Folia Geobot Phytotaxon. 1968; 3: 117-142.

55. Pokorn8 P, Kunes P. Contributions to the European Pollen Database. 5. Kozlí (S. Bohemia, Czech Republic). Grana, 2009; 48: 77-78.

56. Rybnícková E, Rybnícek K. Palaeogeobotanical evaluation of the Holocene profile from the Rezabinec fishpond. Folia Geobot Phytotaxon. 1985; 20: 419-437.

57. Jankovská V. Paläogeobotanische Rekonstruktion der Vegetationsentwicklung im Becken Trebonská pánev während des Spätglazials und Holozäns. Vegetace CSSR A11, Academia, Praha. 1980.

58. Rybnícek K. Die vegetationsverhaltnisse der moore im sudlichen Teil der bohmisch-Mahrischen Hohe. Vegetace CSSR, Praha, 1974; A 6: 1-236.

59. Brízová E. Late Glacial and Holocene development of the vegetation in the Labe (Elbe) River flood-plain (Central Bohemia, Czech Republic). Acta Palaeobot. 1999; 2(Suppl.): 549-554.

60. Svobodová H, Soukupová L, Reille M. Diversified development of mountain mires, Bohemian Forest, Central Europe, in the last 13,000 years. Quat Int. 2002; 91: 123-135.

61. Svobodová H, Reille M, Goeury C,. Past vegetation dynamics of Vltavsk8 luh, upper Vltava river valley in the Sumava mountains, Czech Republic. Veg Hist Archaebot. 2001; 10: 185-199.

62. Jankovská V. Palynologische Erforschung archäologischer Proben aus dem Komoranské jezero-See bei Most (NW-Böhmen). Folia Geobot Phytotaxon. 1988; 23: 45-78.

63. Abraham V. Prirozená vegetace a její zmeny v dusledku kolonizace a lesnického hospodarení v Ceském Sv8carsku (The natural vegetation of Bohemian Switzerland and its changes an impact of habitation and forest management). M.Sc. Thesis, Charles University, Praha. 2006.

64. Pokorn8 P, van der Knaap WO. Contributions to the European Pollen Database. 10.Na Bahne : Vegetation development over the last 2.5 millenia in the Eastern Bohemian lowland. Grana. 2010; 49: 79-81.
65. Speranza A. Solar and anthropogenic forcing of late- Holocene vegetation changes in the Czech Giant Mountains. PhD Thesis, University of Amsterdam, Amsterdam; 2000.
66. Kunes P, Pokorn8 P, Jankovská V. Post-glacial vegetation development in sandstone areas of Czech Republic. In: Härtel H, Cílek V, Herben T, Jackson A, Williams R, editors. Sandstone Landscapes. Praha: Academia; 2007. pp. 224-257.
67. Kremenetski CV. Holocene vegetation and climate history of southwestern Ukraine. Rev Palaeobot Palynol. 1995; 85: 289-301.
68. Artushenko AT, Ya. Arap R, Bezusko LG. History of vegetation of western areas of Ukraine in Quartenary period. Naukova dumka, Kiev, Ukraine; 1982.
69. Cordova CE, Lehman PH. Archaeolalynology of synanthropic vegetation in the chora of Chersonesos, Crimea, Ukraine. J Archaeol Sci. 2003; 30: 1483-1501.
70. Grüger E. The Changing Face of Dalmatia. In: Chapman J, Shiel R, Batovic S, editors. Archaeological and Ecological Studies in a Mediterranean Landscape. Vegetation change. Leicester: Leicester University Press; 1996. pp. 33-44.
71. Jahns S, van der Bogaard C. New palynological and tephrostratigrapical investigations of two salt lagoons on the island of Mljet, south Dalmatia, Croatia. Veget Hist Archaeobot 1998; 7: 219-234.
72. Binka K, Ciesla A, Lacka B, Madeyska T, Marciniak B, Szeroczynska K, The development of Bledowo Lake (central Poland). A palaeoecological study. In: Madeyska T, editor. Pleistocene of Poland. Part 17, Studia Geologica Polonica 100; 1991. pp. 3-85.

73. Szczepanek K. Anthropogenic vegetation changes in the region of the Dukla Pass, the Lower Beskid Mountains. Polska Akademia Umiejetnosci. Prace Komisji Prehistorii Karpat. 2001; 2: 171-182.

74. Milecka K. Pollen analysis of lake sediments in Giecz - The state of the investigation. In: Tobolski K, editor. Wstep do paleoecologii lednideiego parku Krajobvazowego; 1991. pp. 147-150.

75. Tobolski, K. (Polish). In: Tobolski K, editor. Wstep do paleoecologii lednideiego parku Krajobvazowego; 1991. pp. 11-34.

76. Makohonienko M. Przyrodnicza historia Gniezna. Prace Zakladu Biogeografii i paleoekologii. UAM. Homini. Poznan-Gniezno. 2000; 1: 1-121.

77. Miotk G. Badania palinologiczne osadow z polnocnego obrzeza jeziora Godziszewskiego kolo Tczewa/woj. gdanskie Fizjograficzne na Polska Zach. T.XXXVI, Seria A, Geogr.Fiz.: 1986; 1: 123-135.

78. Ralska-Jasiewiczowa M. Type Region P-x: Masurian Great Lakes District. Acta Palaeobot. 1989; 29: 95-100.

79. Obidowicz A. Type Region P-a: Inner West Carpathians- Nowy Targ Basin. Acta Palaeobot. 1989; 29: 11-15.

80. Weirich J, Bortenschlager S. Beiträge zur Vegetationsgeschichte Tirols III: Stubaier Alpen - Zillertaler Alpen. Ber. nat.-med. Verein Innsbruck. 1980; 67: 7-30.

81. Hüttemann H, Bortenschlager S. Beiträge zur Vegetationsgeschichte Tirols VI: Riesengebirge, Hohe Tatra - Zillertal, Kühtai.Ber. nat.-med. Verein Innsbruck. 1987; 74: 81-112.

82. Wahlmüller N. Beiträge zur Vegetationsgeschichte Tirols V: Nordtiroler Kalkalpen. Ber. nat.-med. Verein Innsbruck 1985; 72: 101-144.

83. Bortenschlager S. Beiträge zur Vegetationsgeschichte Tirols I. Inneres Ötztal und unteres Inntal. Ber. nat.-med. Verein Innsbruck 1984; 71: 19-56.

84. van der Knaap WO, Ammann B. Depth-age relationships of 25 well-dated Swiss Holocene pollen sequences archived in the Alpine Palynological Data-Base. Revue de Paléobiologie 1997; 16: 433-480.

85. Bortenschlager I. Beiträge zur Vegetationsgeschichte Tirols II: Kufstein - Kitzbühel - Pass Thurn. Ber. nat.-med. Verein Innsbruck. 1976; 63: 105-137.

86. Krisai R, Mayer W, Schröck C, Türk R. Das Gradenmoos in der Schobergruppe (NP Hohe Tauern, Kärten) Vegetation und Entstehung. Carinthia II. 2006; 196/116: 359-386.

87. Oeggl K. Beiträge zur Vegetationgeschichte Tirols VII: Das Hochmoor Schwemm bei Wachsee. Ber. net.-med. Verein Innsbruck. 1988; 75:3 7-60.

88. Rybnícek K, Rybnícková E. Mooruntersuchung im ob oberen. Folia Geobot Phytotaxon. 1977; 12: 245-291.

89. Krisai R. Die Ufervegetation der Trumer Seen (Salzburg). Dissertationes Botanicae. 1975; 29: 1-202.

90. Krisai R, Burgstaller B, Ehmer-Künkele U, Schiffer R, Wurm E. Die Moore des Ost-Lungaues - Heutige Vegetation, Enstehung, Waldgeschichte ihrer Umgebung. Sauteria, 1991; 5: 1-240.

91. Voigt R. Paläolomnolische und vegetationsgeschichtliche Untersuchungen an Sedimenten aus Fuschlsee und Chiemsee (Salzburg und Bayern). Diss Bot. 1996; 270:1-303.

92. Behre KE. Pollenanalytische Untersuchungen zur Vegetations- und Siedlungsgeschichte bei Flögeln und im Ahlenmoor (Elb-Weser-Winkel). Probleme der Küstenforschung 1976; 11: 101–118.

93. Jahns S. The later Holocene history of vegetation, land-use and settlements around the Ahlequellmoor in the Solling area, Germany. Veg Hist Archaeobot. 2005; 15: 57–65.

94. Willutzki H. Zur Waldgeschichte und Vermoorung sowie über Rekurrenzflächen im Oberharz. Nova Acta Leopold. 1962; 25: 1–52.

95. Chen SH. Neue Untersuchengen über die spät- und postglaziale Vegetationsgeschichte im Gebiet zwischen Harz und Leine (BDR). Flora. 1998; 181: 147-177.

96. Jahns S. Late-glacial and Holocene woodland dynamics and land-use history of the Lower Oder valley, north-eastern Germany, based on two, AMS 14 C-dated, pollen profiles. Veg Hist Archaeobot. 2000; 9: 111–123.

97. Jahns S. Palynological investigations into the Late Pleistocene and Holocene history of vegetation and settlement at the Löddigsee, Mecklenburg, Germany. Veg Hist Archaeobot. 2007; 16: 157–169.

98. Dörfler W. Pollenanalytische Untersuchungen zur Vegetations- und Siedlungsgeschichte im Süden des Landkreises Cuxhaven, Niedersachsen. Probleme der Küstenforschung im südlichen Nordseegebiet. 1989; 17: 1–75.

99. Knipping M.Pollenanalytische Untersuchungen zur Siedlungsgeschichte des Oberpfälzer Waldes. Telma. 1997; 27: 61–74.

100. Rösch M. Botanical evidence for prehistoric and medieval land use in Black Forest. In: Klápšte J, Sommer P, editors. Medieval Rural Settlement in Marginal Landscapes, Ruralia 7. Cardiff: Turnhout Brepols Publishers; 2007. pp. 335–343.

101. Rösch M. Vegetation und Waldnutzung im Nordschwarzwald während sechs Jahrtausenden anhand von Profundalkernen aus dem Herrenwieser See. Standort.wald. 2012; 47: 43-64.

102. Rösch M. Holocene sediment accumulation in the shallow water zone of Lower Lake Constance. Arch Hydrobiol. 1997; 4: 541–562.

103. Rösch M, Ostendorf W. Pollenanalytische, torf- und sedimentpetrographische Untersuchungen an einen telmatischen Profil vom Bodensee-Ufer bei Gaienhofen. Telma. 1988; 18: 373-395.

104. Rösch M, Tserendorj G. a: Der Nordschwarzwald - früher besiedelt als gedacht? Pollenprofile belegen ausgedehnte vorgeschichtliche Besiedlung und Landnutzung. Denkmalpflege in Baden-Württemberg. 2011; 40:6 6-73.

105. Rösch M. Change of land use during the last two millennia as indicated in the pollen record of a profundal core from Mindelsee, Lake Constance region, southwest Germany. Offa. 2013; 69/70: 355-370.

106. Rösch M. Zur Vegetationsgeschichte des südlichen Kraichgaus - Botanische Untersuchungen bei Großvillars, Gemeinde Oberderdingen, Landkreis Karlsruhe. Fundberichte. Aus Baden-Wüttemberg. 2005; 28/1: 839-370.

107. Rösch M. Long-term human impact as registered in an upland pollen profile from the southern Black Forest, southwestern Germany. Veget. Hist. Archaeobot. 2000; 9: 205-218.

108. Richard H, Eschenlohr L. Essai de corrélation entre les données polliniques et les données archéologiques : le cas des forêts de Lajoux dans les Franches-Montagnes (Lajoux, Ju, Suisse). Rev. Archéom. 1998; 22: 29-37.

109. Roos-Barraclough F, van der Knaap WO, van Leeuwen JFN, Shotyk W. A Late-glacial and Holocene record of climate change from a Swiss peat humification profile. Holocene. 2004; 14: 7-19.

110. Hadorn P. Saint-Blaise/Bains des Dames, 1. Palynologie d'un site néolithique et histoire de la végétation des derniers 16000 ans. Neuchâtel. Musée cantonal d'archéologie. Archéologie neuchâteloise. 1994; 18: 1-121.

111. Ammann B. Introduction and Palynology: vegetational history and core correlation at Lobsigensee (Swiss Plateau). In: Lobsigensee - Late-Glacial and Holocene environments of a lake on the central Swiss Plateau. Diss Bot. 1985 87:127-170.

112. Magri D, Sadori L. Late Pleistocene and Holocene pollen stratigraphy at Lago di Vico, central Italy. Veg Hist Archaeobot. 1999; 8: 247-260.

113. Lotter AF, Eicher U, Birks HJB, Siegenthaler U. Late-glacial climatic oscillations as recorded in Swiss lake sediments. J. Quat Sci. 1992; 7: 187-204.

114. Lotter AF, Heiri O, Hofmann W, van der Knaap WO,. van Leeuwen JFN, Walker IR, et al. Holocene timber-line dynamics at Bachalpsee, a lake at 2265 m a.s.l. in the northern Swiss Alps. Veg Hist Archaeobot. 2006; 15: 295-307.

115. Heeb K, Welten M. Moore und Vegetationsgeschichte der Schwarzenegg und des Molassevorlandes zwischen dem Aaretal unterhalb Thun und dem obern Emmental. Mitteilungen der Naturforschenden Gesellschaft in Bern. Neue Folge. 1972; 29: 1-54.

116. Ammann K. Der Oberaargletscher im 18., 19. und 20. Jahrhundert. Zeitschrift für Gletscherkunde und Glazialgeologie XII. 1976; 2: 253-291.

117. Dapples F, Lotter AF, van Leeuwen JFN, van der Knaap WO, Dimitriadis S, Oswald D (2002). Paleolimnological evidence for increased landslide activity due to forest clearing and land-use since 3600 cal BP in the western Swiss Alps. J Paleomnol. 2002; 27: 239-248.

118. van der Knaap WO, van Leeuwen JFN, Fankhauser A, Ammann B (2000). Palynostratigraphy of the last centuries in Switzerland based on 23 lake and mire deposits: chronostratigraphic pollen markers, regional patterns, and local histories. Rev Palaeobot Palynol. 2000; 108: 85-142.

119. Welten M. Vegetationsgeschichtliche Untersuchungen in den westlichen Schweizer Alpen: Bern-Wallis. Denkschriften der Schweizerischen Naturforschenden Gesellschaft, Vol. 95. DSNG 95, 1982. pp. 1-104.

120. Schneebeli M, Küttel M, Fäh J. Die dreidimensionale Entwicklung eines Hanghochmoores im Toggenburg, Schweiz. Vierteljahrsschrift der Naturforschenden Gesellschaft in Zürich. 1989; 134: 1-32.

121. Gfeller C, Oeschger H. Bern Radiocarbon Dates III. Radiocarbon, 1963; 5: 305-311.

122. Welten M Pollenanalytische Untersuchungen zur Vegetationsgeschichte des Schweizerischen Nationalparks. Ergebnisse der wissenschaftlichen Untersuchungen im Schweizerischen Nationalpark. 1982; XVI/80: 1-43.

123. Bégeot C. Histoire de la végétation et du climat au cours du Tardiglaciaire et du début de l'Holocène sur le massif jurassien central à partir de l'analyse pollinique et l'étude des macrorestes végétaux. PhD Thesis, Université de Franche-Comté, Besançon, France; 2000.

124. Grüger J. Untersuchungen zur spätglazialen und frühpostglazialen Vegetationsentwicklung der Südalpen im Umkreis des Gardassees. Botanische Jahrbücher für Systematik Pflanzengeschichte und Pflanzengeographie. 1968; 88: 163-199.

125. Seiwald A. Beiträge zur Vegetationsgeschichte Tirols IV: Natzer Plateau - Villanderer Alm. Ber. nat.-med. Verein Innsbruck. 1980; 67: 31-72.

126. Brugiapaglia E. Dynamique de la végétation tardiglaciaire et holocène dans les Alpes Italiennes nord-occidentales. Unpublished thesis, Marseille, France; 1996.

127. Miras Y, Millet L, Guiter F, Ponel P, de Beaulieu JL, Goslar T. Dynamique des écosystèmes et impact de l'homme dans le secteur du col du Petit Saint Bernard au cours de l'Holocène. Actes du Programme Interreg "Alpis Graïa", 2-4 mars, Aoste. 2006; 1: 31-50.

128. Ortu E, Peyron O, Bordon A, de Beaulieu JL, Siniscalco C, Caramiello R. Lateglacial and Holcene climate oscillations in the South-western Alps: An attempt quantitative reconstruction. Quatern Int. 2008; 190: 71-88.

129. Finsinger W, Bigler C, Krähenbühl U, Lotter AF, Ammann B. Human impacts and eutrophication patterns during the past ~200 years at Lago di Avigliana (N. Italy). J Paleolimnol. 2006; 36: 55-67.
130. Rösch M, Fischer E, Lechterbeck J, Wick L. Pollenanalysen an drei Bohrkernen aus dem Profundal des Ortasees (Piemont, Italien). In: A. Stobbe/U. Tegtmeier, Verzweigungen, eine Würdigung für A.J. Kalis und J. Meurers- Balke, Frankfurter Archäologische Schriften. 2012; 18: 225-247.
131. de Beaulieu JL. Contribution pollenanalytique à l'histoire tardiglaciaire et Holocène de la végétation des Alpes méridionales françaices. PhD Thesis, Université d'Aix-Marseille, Marseille, France; 1977.
132. Kaltenrieder P, Procacci G, Vanniere B, Tinner W. Vegetation and fire history of the Euganean hills (Colli Euganei) as recorded by Lateglacial and Holocene sedimentary series from lago della costa (northeatern Italy). Holocene. 2010; 20: 679-695.
133. Watson CS. The vegetational history of the northern Apennines, Italy: information from three new sequences and a review of Regional vegetational change. J Biogeogr. 1996; 23: 805-841.
134. Drescher-Schneider R., de Beaulieu JL, Magny M, Walter-Simonnet AV, Bossuet G, Millet L, et l. Vegetation history, climate and human impact over the last 15,000 years at Lago dell'Accesa (Tuscany, Central Italy). Veg Hist Archaeobot. 2007; 16: 279-299.
135. Brugiapaglia E, de Beaulieu JL. Etude de la dynamique végétale tardiglaciaire et Holocène en Italie centrale: le marais de Colfiorito (Ombrie). C.R. Acad. Sci. Paris, 1995; t. 321, série IIa: 617-622.
136. Vescovi E, Kaltenrieder P, Tinner W. Late-glacial and Holocene vegetation history of Pavullo nel Frignano, Northern Apennines, . Rev Palaeobot Palynol. 2010; 160: 32-45.

137. Bosi G, Mazzanti MB, Florenzano A, Massamba N'siala I, Pederzoli A, Rinaldi R, et al. Seeds/fruits, pollen and parasite remains as evidence of site function: piazza Garibaldi - Parma (N Italy) in Roman and medieval times. . J Archaeol Sci. 2011; 38: 1621-1633.
138. Kelly MG, Huntley B. An 11000 year record of vegetation and environment from Lago di Martignano, Latium, Italy. J Quaternary Sci. 1991; 6: 209-224.
139. Mercuri AM, Baldini Mazzanti M, Florenzano A, Montecchi MC, Rattighieri E. *Olea, Juglans and Castanea*: the OJC group as pollen evidence of the development of human-induced environments in the Italian peninsula. Quat. Int. 2013; 303: 24-42.
140. Russo Ermolli E, di Pasquale G. Vegetation dynamics of south-western Italy in the last 28 kyr inferred from pollen analysis of a Tyrrhenian Sea core. Veget Hist Archaeobot 2002; 11: 211-219.
141. Allen JRM, Huntley B. last interglacial palaeovegetation, palaeoenvironments and cronology: a new record from lago Grande di Monticchio, southern Italy. Quat Sci Rev. 2009; 28: 1521-1538.
142. Mercuri AM; Florenzano A; Massamba N’siala I; Olmi L; Roubis D; Sogliani F. Pollen from archaeological layers and cultural landscape reconstruction: case studies from the Bradano Valley (Basilicata, southern Italy). Plant Biosyst. 2010; 144: 888-901.
143. Garnaud S, Lesueur P, Clet-Pellerin M, Lesourd S, Garlan T, Lafite R, et al. Holocene to modern fine-grained sedimentation on macrotidal shoreface-to -inner-shelf setting (eastern Bay of the Seine, France). Mar Geol. 2003; 202:33-54.
144. Barbier D. Histoire de la végétation du nord-mayennais de la fin du Weichsélien à l'aube du XXIeme siècle. Mise en évidence d'un Tardiglaciaire armoricain. Interactions Homme-Milieu. PhD Thesis, Université de Nantes. Biologie. Spécialité: Palynologie. 1999.

145. Clet-Pellerin M, Helluin M, Pellerin J, Pilet-Lemiere J, Fontugne M. L'evolution des environnements végétaux pendant les deux derniers millénaires dans la region de Fougers (Ille-et-Vilaine, France). Palynosciences. 1993; 2: 39-55.
146. Billard C, Clet-Pellerin M, Lautridou JP, Giffault M. Un site protohistorique littoral dans le havre de la Vanlee à Lingreville et Bricqueville-sur-mer (Manche). Rev Archéol Ouest. 1995; 12:73-110.
147. Visset L, Sellier D, L'Helgouach J. Le paléoenvironnement de la région de Carnac. Sondage dans le marais de Kerdual, La Trinité-sur-Mer (Morbihan). Rev Archéol Ouest. 1995; 12: 57-71.
148. Ouguerram A, Visset L. Palynologie de la tourbière de Nay dans la vallée de l'Erdre, affluent de la Loire (Massif armoricain, France). Histoire de la végétation et du peuplement humain. Journal de la Société botanique de France, 2001; 13:35-45.
149. Verron G. Le marais de Marchésieux. Découvertes de l' âge du Bronze et étude palynologique des tourbes. Gallia Préhist. 1977; 20-2: 370-374.
150. Visset L, Barbier D, Ouguerram A. Le paysage végétal dans le Bas-Maine (Mayenne, France), le long de la vallée de l'Erve, de la fin du Mésolithique à l'époque récente [Botanic landscape in Bas-maine (Mayenne, France), along the Erve valley, from the end of Mesolithic to recent times]. Rev Archéol Ouest. 2005; 22: 85-92.
151. Piana J, Carcaud N, Cyprien AL, Visset L, Leroy D. Dynamique paysagère tardiglaciaire et holocène dans la vallée du Loir à Pezou (Loir-et-Cher): développements méthodologiques et premiers résultats. Norois. 2009; 213: 73-88.
152. Joly C, Visset L, Scaon C, Pont-Tricoire C, Froquet-Uzel H. Archéologie préventive et évolution du paysage végétal de l Age du Fer au Moyen Age dans le Gâtinais: mise en évidence de culture de chanvre et d'activités de rouissage (Courcelles et Sceaux-en-Gâtinais, Loiret, France). [Preventive archaeology and evolution of the vegetation landscape from the

Iron Age to the Middle Ages in Gâtinais: highlighting hemp culture and retting activities (Courcelles and Sceaux-en-Gâtinais, Loiret, France)]. ArchéoSciences, revue d'archéométrie. 2008; 32: 15-30.

153. De Valk EJ. Late Holocene and present vegetation of the Kastelberg (Vosges, France). PhD Thesis, University of Utrecht, Utrecht, The Netherlands. 1981.

154. Gauthier E. Evolution de l'impact de l'homme sur la végétation du massif jurassien au cours des quatre derniers millénaires. Nouvelles données palynologiques. PhD Thesis, Archéologie-Préhistoire Besançon, Université de Franche-Comté, UFR des Sciences de l'Homme et de la Société. 2001.

155. Clerc J. Recherches pollenanalytiques sur la paléo-écologie Tardiglaciaire et Holocène du Bas-Dauphiné. PhD Thesis, Université St. Jérôme, Marseille, France. 1988.

156. Bossuet G, Ruffaldi P, Magny M, Richard H, Mouthon J. Dynamique et approche quantitative des remplissages fini- et postwürmiens du bassin lacustre de Cerin (Jura, France). B Soc Geol Fr. 1996; 167: 483-493.

157. Noël H, Garbolino E, Brauer A, Lallier-Vergès E, de Beaulieu JL, Disnar JR. Human impact and soil erosion during the last 5000 yrs as recorded in lacustrine sedimentary organic matter at Lac d'Annecy, the French Alps. J Paleolimnol. 2001; 25: 229-244.

158. Joly C, Visset L. Evolution of vegetation landscapes since the Late Mesolithic on the French West Atlantic coast. Rev Palaeobot Palynol. 2009; 154: 124-179.

159. de Beaulieu JL, Kostenzer J, Reich K. Dynamique forestière holocène dans la haute vallée de l'Arve (Haute-Savoie) et migrations de Abies et Picea dans les Alpes occidentales. Diss Bot. 1993; 196: 387-398.

160. de Beaulieu JL, Goeury C. Zonation automatique appliquée à l'analyse pollinique: exemple de la narse d'Ampoix (Puy de Dome, France). Bulletin de l'Association Française pour l'Etude du Quaternaire. 1987; 1: 49-61.

161. Jouffroy-Bapicot I, Vannière B, Gauthier E, Richard H, Monna F, Petit C. 7000 years of vegetation history and land-use changes in the Morvan Mountains : A regional synthesis. Holocene. 2013; 23: 1888-1902.

162. Reille M, de Beaulieu JL. Analyse pollinique de l'étang de Cheylade (Massif Central, France): histoire tardiglaciaire et holocène de la végétation de la planèze de Saint-Flour. CR Acad Sc Paris. 1981; 292: 243-246.

163. Miras Y. L'analyse pollinique du plateau de Millevaches (Massif central, France) et de sites périférIques limousins et auvergnats: Approche des paléoenvironnements, des systèmes agro-pastoraux et évolution des territoires ruraux. [The pollen analysis of the plateau de Millevaches (Massif central, France) and of surrounding sites in Limousin and Auvergne] Thesis of Biology. Université de Franche-Comté. 2004.

164. de Beaulieu JL, Pons A, Reille M. Recherches pollenanalytiques sur l'histoire de la végétation de la bordure nord du Massif du Cantal (Massif-Central, France). Pollen et Spores 1982; 24: 251-300.

165. Reille M, de Beaulieu JL. History of the Würm and Holocene vegetation in western Velay (Massif Central, France): a comparison of pollen analysis from three corings at Lac du Bouchet. Rev Palaeobot Palynol. 1988; 54: 233-248.

166. Richard H. Nouvelles données polliniques en Bresse: le marais de La Peupleraie à Le Miroir (Saône-et-Loire, France) [New pollinic data in Bresse: the peat bog of La Peupleraie at Le Miroir (Saône-et-Loire)]. Comptes rendus de l'Académie des sciences. Série 2. Sciences de la terre et des planets. 1996; 323: 531-538.

167. Vannière B, Martineau R. Histoire des feux et pratiques agraires du Néolithique à l'âge du Fer en région Centre: implications territoriales, démographiques et environnementales. Gallia Préhist. 2005; 47: 167-186.

168. Miras Y, Guenet P, Cruz F, Garcia JP, Petit C, Guillaumet JP. Gestion des ressources naturelles dans le Pays de Tulle: impacts paysagers et histoire du châtaignier (*Castanea sativa* Mill.) de l'Antiquité à la Renaissance d'après la palynologie. Aquitania. 2013; 29: 311-330.
169. Guenet P. Analyses pollenanalytique en Artense et sur le plateau de Millevaches (Massif Central, France). Palynosciences. 1993; 2: 79-108.
170. Surmely F, Miras Y, Guenet P, Nicolas V, Savignat A, Vannière B, et al. Occupation and land-use history of a medium mountain from the Mid-Holocene: A multidisciplinary study performed in the South Cantal (French Massif Central). CR Palevol. 2009; 8: 737-748.
171. Monna F, Petit C, Guillaumet JP, Jouffroy-Bapicot I, Blanchot C, Dominik J, et al. History and Environmental Impact of Mining Activity in Celtic Aeduan Territory Recorded in a Peat Bog (Morvan, France). Research. 2004; 38: 665-673.
172. Castanet C. La Loire en val d'Orléans. Dynamiques fluviales et socio-environnementales durant les derniers 30 000 ans: de l'hydrosystème à l'anthroposystème. Master Thesis in Archéologies Environnementales et Géoarchéologie, Université de Paris 1, Panthéon-Sorbonne. 2008.
173. Miras Y, Guenet P, Richard H. Holocene vegetation, landscape and reconstruction of human activity from Prehistory to the Roman period based on new pollen data performed in "the Plateau de Millevaches" (Limousin, Massif Central, France). Quaternaire. 2011; 22: 147-164.
174. Miras Y, Guenet P, Richard H. La genèse du paysage culturel du plateau de Millevaches (Limousin, Massif Central, France): plus de 2000 ans d'histoire révélés par l'analyse pollinique. In: Grandcoing P. editor. Paysage et environnement en Limousin, de l'Antiquité à nos jours. Limoges: Presses Universitaires de Limoges; 2010. pp. 99-124.

175. Cyprien AL, Visset L, Charrieau L. Etudes palynologiques du site de la Grande Brousse, Vallée de la Choisille, Communes de Cérelles et Chanceaux-sur-Choisille (Indre-et-Loire). Rapport Définitif. Université de Nantes. 2004.

176. Voeltzel D. Recherches pollenanalytiques sur la vegetation holocene de la plaine alluviale de l'estuaire de la Loire et des coteaux environnants. PhD Thesis, University of Aix-Marseille, France. 1987.

177. Cyprien AL, Visset L. Analyse palynologique du site de Bois-Jésus BJE12 - Rapport complémentaire. Université de Nantes. 2006.

178. Visset L, Pont C, Carcaud N, Bernard J, Violot JM. Etude paléoenvironnementale de la vallée du Lane du Néolithique au Moyen-Age, Saint Nicolas de Bourgueil (Indre-et-Loire), la Prairie du Cassoir [Palaeoenvironmental study of the Loire valley from the Neolithic to the Middle Ages]. Quaternaire. 1999; 10: 247-261.

179. Cyprien AL, Carcaud N, Visset L. Etude paléoenvironnementale du Marais de Distré (Saumurois): géoarchéologie d'une zone himide depuis le Préboréal. Quaternaire. 2001; 12: 89-101.

180. Carcaud N, Garcin M, Visset L, Musch J, Burnouf J. Nouvelle lecture de l'évolution des paysages fluviaux à l'Holocène dans le bassin de la Loire moyenne. In: Bravard JP, Magny M, editors. Les fleuves ont une histoire, paléo-environnement des rivières et des lacs français depuis 15000 ans. Errance; 2002. pp. 71–84.

181. Cyprien AL. Chronologie de l'interaction de l'homme et du milieu dans l'espace central et aval de la Loire (Ouest de la France). Thesis in Biological Science, Université de Nantes. 2001.

182. Visset L. Etude pollenanlytique de quelques sites du Marais Poitevin. Bulletin de l'Association Française pour l'étude du Quaternaire. 1987; 2: 81-91.

183. Joly C, Visset L. Etude paléoenvironnementale de la Grande Prée de Varades (La boire Torse) dans le bassin aval de la Loire (Loire-Atlantique, France). Rev Arch Loir. 2011; 35: 9-16.
184. Ouguerram A. Histoire de la vallée de l'Erdre (affluent de la Loire, Massif armoricain, France) de la fin du tardiglaciaire aux époques actuelles. Thesis in Biological Science, Université Moulay Ismaïl (Maroc) and Université de Nantes . 2002.
185. Diot MF, Tastet JP. Paleo-environnements Holocenes et limites chrono-climatiques enregistres dans un marais estuarien de la Gironde . Quaternaire. 1995; 6: 63-75.
186. Poirier N, Morin E, Joly C, Leturcq S, Visset L. Occupation du sol et impact érosif dans la vallée de la Choisille (France, Indre-et-Loire): approches croisées pour la restitution des paysages anciens. [Land Use Dynamics and theit Impact on Erosion in the Choisille Valley (France, Indre-et-Loire): Crossed Approaches to describe Past Landscapes]. ArchéoSciences, revue d'archéometrie. 2013; 37: 67-88.
187. Bernard J. Paléoenvironnement du Pays de Retz et du marais breton-vendéen. PhD Thresis, Université de Nantes. 1996.
188. Marchand G, Tessier M, Bernard J. Les occupations mésolithiques et néolithiques de la Fillauderie (Saint-Père-en-Retz, Loire-Atlantique) et la préhistoire récente de la basse vallée du Boivre. Rev Arch Ouest 1999; 16: 39-65.
189. Cyprien AL, Visset L. Complément à l'étude palynologique du sondage Parcay 1 (Vallée de la Vienne), Laboratoire d'Ecologie et des Paléoenvironnements Atlantiques, 1 vol., dactyl., Université de Nantes. 2007.
190. Wegmüller S. Pollenanalytische Untersuchungen zur spät- und postglazialen Vegetationsgeschichte der französichen Alpen (Dauphiné). Verlag Paul Haupt Bern. 1977; 1: 1-185.

191. Nakagawa T. Etudes palynologiques dans les Alpes Françaises centrales et méridionales: histoire de la végétation Tardiglaciaire et Holocène [Pollen studies in the central meridional French Alps: Lateglacial and Holocene vegetation history] PhD Thesis of Biology and Ecology, University of Marseille. 1998.

192. Puertas O. Premiers indices polliniques de néolithisation dans la plaine de Montpellier (Hérault, France). Bull Soc Préhist Fr. 1999; 96: 15-20.

193. Sanchez-Goñi MF. Analyse palynologique de l'Etang d'Ouveillan. In: Guilaine I, editor. Temps et espace da ns le bassin de l'Aude du Neolithique a l'Age du Fer. CNRS; 1995. pp 265-275.

194. Walsh K, Mocci F. 9000 ans d'occupation du sol en moyenne et haute montagne: la vallée de Freissinières dans le Parc national des Ecrins (Freissinières, Hautes- Alpes). Archéologie du Midi Médiéval. 2003; 21: 185-198.

195. Argant J, Argant A (2000). Mise en évidence de l'occupation ancienne d'un site d'altitude: analyse pollinique du lac de Lauzon (Drôme) Géologie Alpine. Mém H S. 2000; 31: 61-71.

196. Muller SD, David F, Wicha S. Impact de l'exposition des versants et de l'anthropisation sur la dynamique forestiére dans les alpes du sud . Géogr Phys Quat. 2000; 54: 231-243.

197. Pulido M. Conséquenses de l'anthropisation sur la dynamique postglaciaire de la végétation dans le sud du Massif Central, France. [Anthropogenic impact on the postglacial vegetation dynamics in South Massif Central, France] Thesis of Palaeoecology. University of Marseille. 2006.

198. Jouffroy-Bapicot I, Pulido M, Baron S, Galop D, Monna F, Lavoie M, et al. Environmental impact of early palaeometallurgy: pollen and geochemical analysis. Veg Hist Archaebot. 2007; 16: 251-258.

199. Burjachs F, Pérez-Obiol RP, Roure JM, Julia R. Dinamica de la vegetation durante el Holoceno en la isla de Mallorca. In: X Simposio de Palinologia (APLE), Valencia, Spain; 1994. pp. 199-210.

200. Yll EI, Pérez-Obiol RP, Pantaléon-Cano J, Roure JM. Palynological evidence for climatic change and human activity during the Holocene on Minorca (Balearic Islands). Quat Res. 1997; 48: 339-347.

201. Yll EI, Pérez-Obiol RP, Pantaléon-Cano J, Roure JM (1995). Dinamica del paisaje vegetal en la vertiente Mediterra nea de la peninsula Iberica e islas Baleares desde el tardig glaciar hasta el presente. In: Aleixandre T, Perez A, editors. Reconstituction de la ambient es y cambios climaticos durante el Cuaternarion. Centro de Ciencias Medioambientales. CSIC, Madrid, Spain; 1995. pp. 319-328.

202. Pantaléon-Cano J, Yll EI, Pérez-Obiol RP, Roure J.M. Palynological evidence for vegetational history in semi-arid areas of the western Mediterranean (Almeria, Spain). Holocene. 2003; 13:109-119.

203. Cortés-Sánchez M, Morales-Muñiz A, Simón-Vallejo MD, Lozano-Francisco MC, Vera-Peláez JL, Finlayson C, et al. Earliest Known Use of Marine Ressources by Neanderthals. PLoS ONE. 2011; 6: 1-15.

204. Pantaléon-Cano J, Pérez-Obiol RP, Roure JM. La representacion del paisaje vegetal del area de Barcelona durante en ultimo milenio a partir del analisis polinico de sedimentos marinos del delta del rio Besos datados por 210Pb. In: Mateu I, Dupre M, Guemes J, Burgaz ME, editors. Trabajos de palinologia basica y aplicada. Universitat de València; 1994. pp. 211-223.

205. López-Sáez JA, López-Merino L, Alba-Sánchez F, Pérez-Díaz S, Abel-Schaad D, Carrión JS. Late Holocene ecological history of Pinus pinaster forests in the Sierra de Gredos of central Spain. Plant Ecol. 2010; 206: 195-209.

206. Carrión JS, van Geel B (1999). Fine-resolution Upper Weichselian and Holocene palynological record from Navarrés (Valencia, Spain) and a discussion about factors of Mediterranean forest succession. Rev Palaeobot Palynol. 1999; 106: 209-236.

207. Carrion JS, Sanchez-Gomez P (1992). Palynological data in support of the survival of walnut (*Juglans regia* L.) in the western Mediterranean area during last glacial times. J Biogeogr. 1992; 19: 623-630.

208. Carrión JS., Dupré M, Fumanal MP, Montes R. A palaeoenvironmental study in the semiarid southeastern Spain: the palynological and sedimentological sequence at Perneras Cave (Lorca, Murcia). J Archaeol Sci. 1995; 22: 355-367.

209. Penalba MC. The history of the Holocene vegetation in northern Spain from pollen analysis. J Ecol. 1994; 82: 815-832.

210. Mariscal B. Estudio polinico de la turbera del Cueto de la Avellanosa polaciones (Cantabria). VI Reunion do grupo espanol de traballo de Quaternario. Cuadernos do laboratorio xeloxico de laxe. 1983; 5: 205-226.

211. Allen JRM, Huntley B, Watts WA. The vegetation and climate of northwest Iberia over the last 14,000 years. J Quat Sci. 1996; 11: 125-147.

212. López-Merino L, Silva-Sánchez N, Kaal J, López-Sáez JA, Martínez-Cortizas A. Post-disturbance vegetation dynamics during the Late Pleistocene and Holocene : An example from NW Iberia. Global Planet Change. 2012; 92- 93: 58-70.

213. López-Sáez JA, López-Merino L, Mateo MA, Serrano O, Pérez-Díaz S, Serrano L. Palaeoecological potential of the marine organic deposits of Posidonia oceanica: A case study in the NE Iberian Peninsula. Palaeogeogr Palaeoclimatol Palaeoecol. 2009; 271: 215-224.

214. Penalba MC. Dynamique de vegetation Tardiglaciaire et Holocene du centre-nord de l'Espagne d'après l'analyse pollinique. PhD Thesis, Université d'Aix-Marseille, Marseille, France. 1989.

215. Abel-Schaad D, Hernández-Carretero AM, López-Sáez JA, Pulido-Díaz FJ, López-Merino L, Martínez-Cortizas A. Evolución de la vegetación en la Sierra de Gata (Cáceres-Salamanca, España) durante el Holoceno reciente. Implicaciones biogeográficas. Rev Esp Micropaleontol. 2009; 41: 91-105.

216. Dorado-Valiño M, López-Sáez JA, García-Gómez E. Contribution to the European Pollen Database. 21. Patateros, Toledo Mountains (central Spain). Grana. 2014; 53: 171-173.

217. Abel-Schaad D, López-Sáez JA. Vegetation changes in relation to fire history and human activities at the Peña Negra mire (Bejar Range, Iberian Central Mountain Sysrem, Spain) during the past 4,000 years. Veg Hist Archaeobot. 2013; 22: 199-214.

218. López-Merino L, López-Sáez JA, Pérez-Díaz S, Carrión JS. 2000 years of pastoralism and fire shaping high-altitude vegetation of Sierra de Gredos in central Spain. Rev. Palaeobot. Palynol. 2009; 158: 42-51.

219. van der Knaap WO, van Leeuwen JFN. Late-Glacial and early-Holocene vegetation succession, altitudinal vegetation zonation, and climatic change in the Serra da Estrela, Portugal. Rev Palaeobot Palynol. 1997; 97: 239-285.

220. van der Brink LM, Janssen CR. The effect of human activities during cultural phases on the development of montane vegetation in the Serra da Estrela, Portugal. Rev Palaeobot Palynol. 1985; 44: 193-215.

221. Mateus JE. Holocene and present-day ecosystems of the Carvalhal region, South-west Portugal. PhD Thesis, University of Utrecht, Utrecht, The Netherlands. 1992.

222. Gremmen WHE, Bottema S. Palynological investigations in the Syrian Gazira. In: Kühne H. editor. Die rezente Umwelt von Tell Šēh Hamad und Daten zur Umweltrekonstruktion der assyrischen Stadt Dūr Katlimmu, Berlin; 1991. pp. 105–116.

223. Yasuda Y, Kitagawa H, Nakagawa T. The earliest record of major anthropogenic deforestation in the Ghab Valley, northwest Sirya: a palynological study. Quatern Int. 2000; 73/74: 127-136.
224. Neumann FH, Kagana EJ, Schwab MJ, Stein M. Palynology, sedimentology and palaeoecology of the late Holocene Dead Sea. Quat Sci Rev. 2007; 26: 1476-1498.
225. Neumann F, Scholzel C, Litt T, Hense A, Stein M. Holocene vegetation and climate history of the northern Golan heoghts (Near East). Veget Hist Archaeobot .2007; 16: 329-346.
226. Baruch U. The Late Holocene Vegetational History of Lake Kinneret (Sea of Galilee), Israel. Palèorient. 1986; 12: 37-48.
227. van Zeist W, Baruch U, Bottema S (2009). Holocene palaeoecology of the Hula area, northeastern Israel. In: Kaptijn K, Petit LP. editors. A Timeless Vale. Archaeological and Related Essays on the Jordan Valley in Honour of Gerrit Van Der Kooij on the Occasion of his Sixty-Fifth Birthday. Leiden: Leiden University Press. p 29-64.
228. Leroy SAG, Kakroodi AA, Kroonenberg S, Lahijani H, Alimohammadian H, Nigarov A. Holocene vegetation hitory and sea level changes in the SE corner of the Caspian Sea: relevance to SW Asia climate. Quat Sci Rev. 2013; 70: 28-47.
229. Djamali M, de Beaulieu JL, Miller NF, Andrieu-Ponel V, Ponel P, Lak R, Sadeddin N, et al. Vegetation history of the SE section of the Zagros Mountains during the last five millennia; a pollen record from the Maharlou Lake, Fars Province, Iran. Veg Hist Archaeobot. 2009; 18: 123-136.
230. Djamali M, de Beaulieu JL, Shah-hosseini M, Andrieu-Ponel V, Ponel P, Amini A, et al. A late Pleistocene long pollen record from Lake Urmia, NW Iran. Quat Res. 2008; 69: 413-420.

231. Henry AG, Brooks AS, Piperno DR. Microfossils in calculus demonstrate consumption of plants and cooked foods in Neanderthal diets (Shanidar III, Iraq; Spy I and II, Belgium). Proc Natl Acad Sci USA. 2011; 108: 486-491.

232. Kvavadze E, Rukhadze LP. Vegetation and climate of the Holocene of Abkhazia. Metsniereba, Tbilisi, Georgia, 1989.

233. Kvavadze EV. The new data on the stratigraphy and palaeogeoraphy of the Holocene of the Colchida lowland. In: Quaternary system of Georgia. Tbilisi, Metsniereba (in Russian); 1982. pp. 123-130.

234. Kvavadze EV, Efremov YuV. The results of palynogical studies of the Holocene deposites in the highlands of the Lagodekhi Reservation (Eastern Georgia). Bulletin of the Georgian Academy of Sciences. 1990; 139: 641-644.

235. Bar-Yosef O, Belfer-Cohen A, Mesheviliani T, Jakeli N, Bar-Oz G, Boaretto E, Goldberg P, et al. Dzudzuana: an Upper Palaeolithic cave site in the Caucasus foothills (Georgia). Antiquity. 2011; 85: 331-349.

236. Messager E, Lebreton V, Marquer L, Russo-Ermolli E, Orain R, Renault-Miskovsky J, et al. Palaeoenvironments of early hominins in temperate and Mediterranean Eurasia: new palaeobotanical data from Palaeolithic key-sites and synchronous natural sequences. Quaternary Sci Rev 2011; 30: 1439-1447.

237. Kvavadze EV, Connor SE. Zelkova carpinifolia (Pallas) K. Koch in Holocene sediments of Georgia- an indicator of climatic optima. Rev Palaeobot Palynol. 2005; 133: 69-89.

238. Bordon A, Peyron O, Lezine AM, Brewer S, Fouache E. Pollen-inferred Late-Glacial and Holocene climate in southern Balkans (Lake Maliq). Quatern Int. 2009; 200: 19-30.

239. Sadori L, Koutsodendris A, Panagiotopoulos K, Masi A, Bertini A, Combourieu-Nebout N, et al. Pollen-based paleoenvironmental and paleoclimatic change at Lake Ohrid (SE Europe) during the past 500 ka. Biogeosciences 2016; 13: 1423–1437.

240. Huntley B, Birks HJB. An atlas of present and past pollen maps for Europe, 0–13,000 years ago. 1st ed New York: Cambridge University Press; 1983.

241. Cordova CE, Lehmanb PH. Archaeopalynology of synanthropic vegetation in the chora of Chersonesos, Crimea, Ukraine. J Archaeol Sci. 2003; 30: 1483–1501.

242. Kvavadze EV. Redeposited pollen in recent Holocene sediments of the Caucasus Mountains. Grana. 1996; 35: 33–37.

243. Geraga M, Tsaila-Monopolis St, Ioakim Chr, Papatheodorou G, Ferentinos G. Evaluation of palaeoenvironmental changes during the last 18,000 years in the Myrtoon basin, SW Aegean Sea. Palaeogeogr. Palaeoclimatol. Palaeoecol. 2000; 156: 1-17.
