# Supplementary material for: Rethinking the history of common walnut (Juglans regia L.) in Europe: Its origins and human interactions
Source: PLoS One. 2017 Mar 3;12(3):e0172541. doi: 10.1371/journal.pone.0172541 (PMC5336217; doi:10.1371/journal.pone.0172541)
Supplement: S2 Table — Number of samples (N), geographic coordinates (Lat, Long) and elevation above sea level (Elev) for 91 common walnut populations collected in Eurasia. (DOCX) [file pone.0172541.s005.docx]

**S2 Table. Description of 91 common walnut populations sampled in Eurasia.** Number of samples (N), geographic coordinates (Lat, Long) and elevation above sea level (Elev) for 91 common walnut populations collected in Eurasia.

| Country | Province | Mountain range | Site  N° | Population | Abbreviation | Lat. | Long. | Elev.  (m) | N |
| --- | --- | --- | --- | --- | --- | --- | --- | --- | --- |
| Kyrgyzstan | Jalal-Abad | Western Tien Shan | 1 | Ak-Terek | TEREK | 40°27’00’’N | 72°45’00’’E | 1700 | 45 |
|  |  | Western Tien Shan | 2 | Sharap | SHARAP | 41°16’12’’N | 72°50’59’’E | 1620 | 18 |
|  |  | Western Tien Shan | 3 | Yaradar | YARADAR | 41°15’00’’N | 73°00’00’’E | 1260 | 16 |
|  |  | Western Tien Shan | 4 | Shaidan | SHAIDAN | 41°04’48’’N | 72°42’00’’E | 1590 | 24 |
|  |  | Western Tien Shan | 5 | Kyzyl-Ungur | KYZYL | 41°24’00’’N | 73°00’00’’E | 1400 | 45 |
|  |  | Western Tien Shan | 6 | Katar-Yangak | KATAR | 41°15’00’’N | 72°45’00’’E | 1900 | 19 |
|  |  | Western Tien Shan | 7 | Kyok-Sarau | KYOK | 41°17’59’’N | 72°52'47’’E | 1830 | 25 |
|  |  | Western Tien Shan | 8 | Kyr-sai ^a^ | KYR | 41°50’24’’N | 71°57’00’’E | 1320 | 27 |
|  |  | Western Tien Shan | 9 | Ters-Kolt ^a^ | TERS | 41°49’12’’N | 71°56’23’’E | 1440 | 24 |
| Uzbekistan | Namangan | Western Tien Shan | 10 | Kamchik | KAMCHIK | 41°04’60’’N | 70°31’00’’E | 1431 | 18 |
|  | Fergana | Western Tien Shan | 11 | Yakkatut | YAKKATUT | 40°26’21’’N | 70°53’14’’E | 389 | 18 |
|  | Tashkent | Western Tien Shan | 12 | Sidjak | SIDJAK | 41°41’27’’N | 70°03’28’’E | 845 | 10 |
|  |  | Western Tien Shan | 13 | Charvak | CHARVAK | 41°64’00’’N | 70°03’00’’E | 1350 | 18 |
|  |  | Western Tien Shan | 14 | Nanai | NANAI | 41°43’00’’N | 70°05’60’’E | 1338 | 18 |
|  |  | Western Tien Shan | 16 | Bogustan | BOGUSTAN | 41°40’60’’N | 70°04’60’’E | 1314 | 20 |
|  |  | Western Tien Shan | 17 | Bostanlyk ^b^ | BOSTANLIK | 41°34’18’’N | 69°45’51’’E | 455 | 18 |
|  |  | Western Tien Shan | 19 | Karankul | KARANKUL | 41°34’26’’N | 69°52’55’’E | 1680 | 36 |
|  | Surkhandarya | Zaamin | 15 | Djarkurgan | DJARKU | 37°30’00’’N | 67°25’00’’E | 480 | 16 |
|  | Dijzak | Gissar | 18 | Bakhmal ^c^ | BAKHMAL | 39°43’00’’N | 68°01’00’’E | 1229 | 15 |
|  | Jizak | Nurata | 20 | Farish | FARISH | 40°34’60’’N | 66°52’00’’E | 486 | 19 |
|  |  | Nurata | 21 | Andigen | ANDIGEN | 40°35’14’’N | 66°45’29’’E | 662 | 12 |
|  |  | Nurata | 22 | Katta-Bogdan | KATTA | 40°25’29’’N | 67°12’13’’E | 920 | 38 |
|  |  | Nurata | 23 | Khayat | KHAYAT | 40°31’38’’N | 66°46’24’’E | 968 | 16 |
|  |  | Nurata | 24 | Yamchi-sai | YAMCHI | 40°20’23’’N | 67°11’32’’E | 906 | 10 |
|  |  | Nurata | 25 | Karri-sai | KARRI | 40°30’05’’N | 66°47’27’’E | 1043 | 20 |
|  |  | Nurata | 26 | Madjerum-sai^d^ | MADJERUM | 40°35’00’’N | 66°42’30’’E | 728 | 28 |
| China | Xinjiang | Eastern Tien Shan | 27 | Gongliu-1 ^e^ | GUILI-1 | 43°22’15’’N | 82°16’03’’E | 650 | 34 |
|  |  | Eastern Tien Shan | 28 | Gongliu-2 ^e^ | GUILI-2 | 43°20’46’’N | 82°16’57’’E | 600 | 67 |
|  |  | Eastern Tien Shan | 29 | Gongliu-3 ^e^ | GUILI-3 | 43°20’24’’N | 82°15’32’’E | 569 | 27 |
|  |  | Eastern Tien Shan | 30 | Urumqi | URUMQI | 43°48’00’’N | 87°35’00’’E | 850 | 29 |
|  | Shandong | - | 31 | Sunbè | SUNBE | 36°12’00’’N | 117°05’00’’E | 167 | 19 |
|  | Tibet | Eastern Himalayas | 32 | Dashuicun | DASH | 29°25’00’N | 90°58’00’’E | 3650 | 48 |
| Pakistan | Gilgit-Baltistan | Western Himalayas | 33 | Gilgit Valley | GILGIT | 35°55’00’’N | 74°20’02’’E | 1493 | 21 |
|  |  | Western Himalayas | 34 | Hunza Valley | HUNZA | 36°19’00’’N | 74°39’00’’E | 2500 | 25 |
| Tadjikistan | Karotegin | Pamir | 35 | Shouli | SHOULI | 38°57’00’’N | 70°00’00’’E | 1882 | 16 |
| Iran | Mazandaran | Alborz | 36 | Karaj | KARAJ | 36°28’00’’N | 52°00’00’’E | 1401 | 12 |
| Georgia | Kakheti | Trans-Caucasus | 37 | Lagodekhi ^f^ | LAGO | 41°49’00’’N | 46°17’00’’E | 1500 | 15 |
|  | Shida Kartli | Trans-Caucasus | 38 | Skra | SKRA | 41°59’28’’N | 44°00’49’’E | 1182 | 21 |
| Turkey |  | Trans-Caucasus | 39 | Anatolia | ANATOLIA | 38°42’00’’N | 42°27’00’’E | 1650 | 19 |
|  | Trabzon | Trans-Caucasus | 40 | Trabzon | TRABZON | 41°00’00’’N | 39°44’00’’E | 776 | 22 |
| Greece | Macedonia | Paiko-Balkans | 41 | Paiko_A | PAIKO-A | 40°57’11’’N | 22°20’8.9’’E | 1200 | 24 |
|  | Macedonia | Paiko-Balkans | 42 | Paiko_B | PAIKO-B | 40°57’09’’N | 22°22’50’’E | 1300 | 27 |
|  | Peloponnese | Arcadia-Balkans | 43 | Arcadia | ARCADIA | 37°35’00’’N | 22°15’00’’E | 482 | 20 |
|  | West Crete | - | 44 | Chania | CHANIA | 35°25’00’’N | 23°55’00’’E | 450 | 33 |
| Romania | Transylvania | Southern Carpathians | 45 | Brasov | BRASOV | 45°39’00’’N | 25°36’00’’E | 478 | 26 |
| Moldova | Chișinău | _ | 46 | Chișinău | CHISINAU | 46°52’5.14’’N | 28°47’42’’E | 85 | 34 |
| Hungary | Bács-Kiskun | - | 47 | Ctsatalja | CTSATALJA | 46°01’59’’N | 18°57’00’’E | 88 | 30 |
|  |  | - | 48 | Melykut | MELYKUT | 46°12’40’’N | 19°23’13’’E | 132 | 32 |
|  | Baranya | - | 49 | Pecs | PECS | 46°08’00’’N | 18°17’00’’E | 440 | 31 |
|  | Fejér | - | 50 | Dunava | DUNAVA | 46°58’50’’N | 18°54’45’’E | 132 | 20 |
|  | Szabolcs-Szatmár-Bereg | Eastern Carpathians | 51 | Milota | MILOTA | 48°06’15’’N | 22°46’54’’E | 119 | 20 |
|  |  | Eastern Carpathians | 52 | Nagyar | NAGYAR | 48°03’00’’N | 22°34’00’’E | 112 | 30 |
|  |  | Eastern Carpathians | 53 | Tiszakorod | TISZAKOROD | 48°06’29’’N | 22°42’39’’E | 111 | 29 |
|  |  | Eastern Carpathians | 54 | Vasarosnameny | VASARO | 48°07’35’’N | 22°19’05’’E | 122 | 20 |
|  | Györ-Moson-Sopron | - | 55 | Bony | BONY | 47°39’07’’N | 17°52’13’’E | 120 | 38 |
|  |  | - | 56 | Mosonmagyar | MOSONM | 47°52’25’’N | 17°16’07’’E | 119 | 20 |
| Slovakia | Nitra | Western Carpathians | 57 | Nová Dedina | DEDINA | 48°16’44’’N | 18°38’29’’E | 220 | 34 |
| France | Centre | - | 58 | Orleans | ORLEAN | 47°55’00’’N | 1°54’00’’E | 116 | 16 |
|  | Poitou-Charentes | - | 59 | Poitiers | POITIERS | 46°34’55’’N | 0°20’10’’E | 129 | 31 |
|  | Auvergne | - | 60 | Puy-de-Dome | PUYDOME | 45°46’59’’N | 3°4’56’’E | 526 | 45 |
|  | Rhone-Alpes | Rhône-Alpes | 61 | Chambéry | CHAMBERY | 45°45’35’’N | 4°50’32’’E | 270 | 44 |
| Spain | Catalogna | Pyrenees | 62 | Girona | GIRONA | 41°59’04’’N | 2°49’16’’E | 175 | 20 |
| Italy | Veneto | Belluno's pre-Alps | 63 | Osigo | OSIGO | 46°0'0"N | 12°20'00"E | 327 | 13 |
|  | Friuli | - | 64 | Pordenone | PORD | 45°57'45"N | 12°39'22"E | 24 | 10 |
|  |  | Eastern Alps | 65 | Preone | PREONE | 46°23'43"N | 12°52'3"E | 460 | 12 |
|  |  | - | 66 | Gabria | GABRIA | 45°54'26"N | 13°34'32"E | 49 | 10 |
|  | Umbria | - | 67 | Castel Giorgio | GIORGIO | 42°42'23"N | 11°58'38"E | 559 | 15 |
|  | Lazio | - | 68 | Palombara Sabina | SABINA | 42°4'3"N | 12°46'8"E | 372 | 8 |
|  | Abruzzo | Southern Apennines | 69 | Pescasseroli^g^ | PESC | 41°48'18"N | 13°47'23"E | 1200 | 20 |
|  |  | Southern Apennines | 70 | Civitella Alfedena^g^ | ALF | 41°45'58"N | 13°56'36"E | 1123 | 18 |
|  |  | Southern Apennines | 71 | Villetta Barrea^g^ | BARREA | 41°46'36"N | 13°56'21"E | 1000 | 21 |
|  | Molise | Southern Apennines | 72 | Montenero Val Cocchiaro | VALCO | 41°43'4"N | 14°4'13"E | 950 | 10 |
|  |  | Southern Apennines | 73 | Rionero Sannitico | RIONERO | 41°42'46"N | 14°8'22"E | 1051 | 8 |
|  |  | Southern Apennines | 74 | Forlì del Sannio | SANNIO | 41°41'48"N | 14°10'49"E | 610 | 20 |
|  |  | Southern Apennines | 75 | Miranda | MIRA | 41°38'45"N | 14°14'49"E | 860 | 14 |
|  |  | Southern Apennines | 76 | S. Maria del Molise | FONT | 41°33'13"N | 14°22'8"E | 650 | 20 |
|  |  | Southern Apennines | 77 | San Massimo | MAS | 41°29'38"N | 14°24'39"E | 630 | 20 |
|  |  | Southern Apennines | 78 | Sepino^h^ | ALTILIA | 41°24'32"N | 14°37'9"E | 698 | 20 |
|  | Campania | Southern Apennines | 79 | Santa Croce del Sannio | CROCE | 41°23'21"N | 14°43'59"E | 478 | 11 |
|  |  | Southern Apennines | 80 | Circello | CIRCE | 41°21'21"N | 14°48'35"E | 650 | 20 |
|  |  | Southern Apennines | 81 | San Marco dei Cavoti | CAVOTI | 41°18'39"N | 14°52'50"E | 695 | 29 |
|  |  | Southern Apennines | 82 | San Giorgio la Molara | MOLARA | 41°16'26"N | 14°55'52"E | 667 | 8 |
|  |  | Southern Apennines | 83 | Montecalvo Irpino | MONTEC | 41°11'47"N | 15°2'3"E | 623 | 26 |
|  |  | Southern Apennines | 84 | Ariano Irpino | ARIANO | 41°8'59"N | 15°5'3"E | 788 | 23 |
|  |  | Southern Apennines | 85 | Casolla | CASOLLA | 41°5'43"N | 14°21'24"E | 200 | 10 |
|  |  | Southern Apennines | 86 | Tufino | TUFINO | 40°57'18"N | 14°34'0"E | 306 | 10 |
|  |  | Southern Apennines | 87 | San Michele Serino | SERINO | 40°53'35"N | 14°51'19"E | 364 | 10 |
|  |  | Southern Apennines | 88 | Montella | MONT | 40°50'40"N | 15°1'7"E | 670 | 20 |
|  | Sicily | - | 89 | Ragusa | RAGUSA | 36°55'45"N | 14°43'4"E | 502 | 10 |
|  |  | - | 90 | Anapo Valley | ANAPO | 37°9'27"N | 15°1'36"E | 438 | 10 |
|  |  | - | 91 | Bivona | BIVONA | 37°37'13"N | 13°27'21"E | 503 | 10 |
| Total | - |  |  | - |  |  | - | - | 2008 |

^a^ Sary-Chalek Bioshpere Reserve

^b^ Ugam-Chatkal National Park

^c^ Zaamin National Park

^d^ Nurata Nature Reserve

^e^ Gongliu Wild Walnut Nature Reserve

^f^ Lagodekhi Nature Reserve

^g^ National Park of Abruzzo

^h^ Archeological site of the Roman town Saepinum/Altili
