# Supplementary material for: Rethinking the history of common walnut (Juglans regia L.) in Europe: Its origins and human interactions
Source: PLoS One. 2017 Mar 3;12(3):e0172541. doi: 10.1371/journal.pone.0172541 (PMC5336217; doi:10.1371/journal.pone.0172541)
Supplement: S3 Table — Population pools and the prior distributions of the parameters used for the two stages of DIYABC analysis. (DOCX) [file pone.0172541.s006.docx]

**S3 Table. Parameters used for DIYABC analysis.** Population pools and the prior distributions of the parameters used for the two stages of DIYABC analysis.

| Stage | Scenario tested | Population Pools | Parameter priors |
| --- | --- | --- | --- |
|  |  |  |  |
| 1 | 1a - 5a | **Pool 1** – ANATOLIA, TRABZON  **Pool 2** - PAIKO_A, PAIKO_B, ARCADIA, CHANIA, BRASOV, CHISINAU  **Pool 3** - CTSATALJIA, MELYKUT PECS, DUNAVA, MILOTA, NAGYAR, TISZAKOROD, VASARO  **Pool 4** - BONY, MOSONM, ORLEAN, POITIERS, PUYDOME CHAMBERY, GIRONA, OSIGO, PORD, PREONE, GABRIA, GIORGIO, SABINA, PESC, ALF, BARREA, VALCO, RIONERO, SANNIO, MIRA, FONT, MAS, ALTILIA, CROCE, CIRCE, CAVOTI, MOLARA, MONTEC, ARIANO, CASOLLA, TUFINO, SERINO, MONT, RAGUSA, ANAPO, BIVONA | Effective population size  N1 = [10 – 10E+03] (Uniform)  N2 = [10 – 10E+03] (Uniform)  N3 = [10 – 10E+03] (Uniform)  N4 = [10 – 10E+03] (Uniform)  N1b = [10 – 10E+03] (Uniform)  N2b = [10 – 10E+03] (Uniform)  N3b = [10 – 10E+03] (Uniform)  Nm = [10 – 10E+03] (Uniform)  Na = [10 – 10E+03] (Uniform)  N2b < N2; N3b < N3; N4b < N4; Nm > N4  Time of events in generations  *t_m_* = [1-10]  *t_1b_* = [1-20]  *t_1_* = [1-20]  *t_2b_* = [1-30]  *t_2_* = [1-30]  *t_3b_* = [20-60]  *t_3_* = [20-60]  t_4_ = [10-10E+03]  *t_1_ ≥ t_1b_; t_2b_ > t_1b_; t_2b_ > t_1_; t_2_ ≥ t_2b_; t_2_ > t_1_; t_2_ > t_1b_; t_3b_ > t_1_; t_3b_ > t_1b_; t_3b_ > t_2_; t_3b_ > t_2b_; t_3_ ≥ t_3b_; t_3_ > t_1_; t_3_ > t_2_; t_4_ > t_3_; t_4_ > t_2_*  Admixture rate  ra = [0.001-0.999] (Uniform)  Mean mutation rate  u = [1.0E-04 - 1.0E-03] (Uniform) |
|  |  |  |  |
| 2 | 1b – 6b | **Pool 1** – ANATOLIA, TRABZON  **Pool 2** - PAIKO_A, PAIKO_B, ARCADIA, CHANIA, BRASOV, CHISINAU  **Pool 3** - CTSATALJIA, MELYKUT PECS, DUNAVA, MILOTA, NAGYAR, TISZAKOROD, VASARO  **Pool 4** - BONY, MOSONM, ORLEAN, POITIERS, PUYDOME CHAMBERY, GIRONA, OSIGO, PORD, PREONE, GABRIA, GIORGIO, SABINA, PESC, ALF, BARREA, VALCO, RIONERO, SANNIO, MIRA, FONT, MAS, ALTILIA, CROCE, CIRCE, CAVOTI, MOLARA, MONTEC, ARIANO, CASOLLA, TUFINO, SERINO, MONT, RAGUSA, ANAPO, BIVONA | Effective population size  N1 = [10 – 10E+03] (Uniform)  N2 = [10 – 10E+03] (Uniform)  N3 = [10 – 10E+03] (Uniform)  N4 = [10 – 10E+03] (Uniform)  NG1 = [10 – 10E+03] (Uniform)  Nd = [10 – 10E+03] (Uniform)  Nm = [10 – 10E+03] (Uniform)  Na = [10 – 10E+03] (Uniform)  Nm > N1; Nd < N2  Time of events in generations  *t_m_* = [1-10]  *t_d_* = [1-15]  *t_1_* = [10-25]  *t_2_* = [20-60]  *t_3_* = [10-200]  *t_4_* = [100-10E+04]  *t_1_ > t_m_; t_1_ ≥ t_d_; t_2_ > t_1_; t_2_ > t_d_; t_2_ > t_m_; t_3_ > t_2_; t_3_ > t_1_; t_3_ > t_d_; t_4_ > t_2_; t_4_ ≥ t_3_*  Admixture rate  ra = [0.001-0.999] (Uniform)  rb = [0.001-0.999] (Uniform)  Mean mutation rate  u = [1.0E-04 - 1.0E-03] (Uniform) |
