# Supplementary material for: Rethinking the history of common walnut (Juglans regia L.) in Europe: Its origins and human interactions
Source: PLoS One. 2017 Mar 3;12(3):e0172541. doi: 10.1371/journal.pone.0172541 (PMC5336217; doi:10.1371/journal.pone.0172541)
Supplement: S4 Table — Description of the scenarios of common walnut expansion across Europe considered in the two stages of DIYABC analysis. (DOCX) [file pone.0172541.s007.docx]

**S4 Table. Scenarios considered in the two stages of DIYABC analysis**. Description of the scenarios of common walnut expansion across Europe considered in the two stages of DIYABC analysis.

| Scenario | Refugial Location |  | DIYABC Analysis |
| --- | --- | --- | --- |
| 1° Stage |  |  |  |
| 1a | Anatolia | Human-mediated expansion of walnut from Anatolia (Pool 1) through the Balkans (Pool 2) and North-Eastern Europe (Pool 3) to Western Europe (Pool 4). | Three colonization events (*t_3_, t_2_, t_1_*) with initial size reduction for each colonization (*t_3b_*, N2b for Pool 2; *t_2b_*, N3b for Pool 3; *t_1b_*, N4b for Pool 4). |
| 2a | Anatolia | Human-mediated expansion of walnut from Anatolia (Pool 1) through the Balkans (Pool 2) to Western Europe (Pool 4) and subsequent spread from Western to North-Eastern Europe (Pool 3). | Three colonization events (*t_3_, t_2_, t_1_*) with initial size reduction for each colonization (*t_3b_*, N2b for Pool 2; *t_2b_*, N2b for Pool 2; *t_1b_*, N3b for Pool 3). |
| 3a | Anatolia  Balkans | Human-mediated expansion of walnut from the Balkans (Pool 2) through the North-Eastern Europe (Pool 3) to Western Europe (Pool 4) using natural stands of *J. regia* located in the Balkans. Independent evolution of walnut in Anatolia (Pool 1). | Pool 1 and Pool 2 diverged at *t_4_*. Two colonization events (*t_2_, t_1_*) with initial size reduction for each colonization (*t_2b_*, N3b for Pool 3; *t_1b_*, N4b for Pool 4). |
| 4a | Anatolia  Balkans | Human-mediated expansion of walnut from the Balkans (Pool 2) to Western Europe (Pool 4) using natural stands of *J. regia* located in the Balkans. Subsequent expansion from Western to North-Eastern Europe (Pool 3). Independent evolution of walnut in Anatolia (Pool 1). | Pool 1 and Pool 2 diverged at *t_4_*. Two colonization events (*t_2_, t_1_*) with initial size reduction for each colonization (*t_2b_*, N4b for Pool 4, *t_1b_*, N3b for Pool 3). |
| 5a | Anatolia  W Europe | Human-mediated dispersal of walnut from Anatolia (Pool 1) and Western Europe (Pool 4) to the Balkans promoting the admixture event (Pool 2). Subsequent walnut expansion from Western Europe to North-Eastern Europe (Pool 3). Final human-induced population size decline in Western Europe. | Pool 1 and Pool 4 diverged at *t_4_*. One admixture event between Pool 1 and Pool 4 (*t_3_*) with an admixture rate *ra* given rise to Pool 2. One colonization event (*t_2_*) with initial size reduction (*t_2b_*, N3b for Pool 3) and one bottleneck event (*t_m_*) with final size reduction (Nm for Pool 4). |
| 2° Stage |  |  |  |
| 1b | Anatolia  W Europe | Human-mediated dispersal of walnut from Anatolia (Pool 1) and Western Europe (Pool 4) to the Balkans promoting the admixture event (Pool 2). Subsequent walnut expansion from Western Europe to North-Eastern Europe (Pool 3). Final human-induced population size decline in Western Europe. | Pool 1 and Pool 4 diverged at *t_3_*. One admixture event between Pool 1 and Pool 4 (*t_2_*) with an admixture rate *ra* given rise to Pool 2. One colonization event from Pool 4 (*t_1_*) with initial size reduction (*t_d_*, Nd for Pool 3) and one bottleneck event (*t_m_*) with final size reduction (Nm for Pool 4). |
| 2b | Anatolia  W Europe | Human-mediated dispersal of walnut from Anatolia (Pool 1) and Western Europe (Pool 4) to the Balkans promoting the admixture event (Pool 2)*.* Subsequent walnut expansion from the Balkans to North-Eastern Europe (Pool 3). Final human-induced population size decline in Western Europe. | Pool 1 and Pool 4 diverged at *t_3_*. One admixture event between Pool 1 and Pool 4 (*t_2_*) with an admixture rate *ra* given rise to Pool 2. One colonization event from Pool 2 (*t_1_*) with initial size reduction (*t_d_*, Nd for Pool 3) and one bottleneck event (*t_m_*) with final size reduction (Nm for Pool 4). |
| 3b | Anatolia  W Europe | Human-mediated dispersal of walnut from Anatolia (Pool 1) and Western Europe (Pool 4) to the Balkans promoting the admixture event (Pool 2)*.* Subsequent walnut dispersal from the Balkans and Western Europe to North-Eastern Europe promoting the second admixture event (Pool 3). Final human-induced population size expansion in the North-Eastern Europe and population size decline in Western Europe. | Pool 1 and Pool 4 diverged at *t_3_*. Two admixture events between Pool 1 and Pool 4 (*t_2_*) given rise to Pool 2 and between Pool 4 and Pool 2 (*t_1_*) given rise to Pool 3 with an admixture rate *ra* and *rb* respectively. One expansion event (*t_d_*, Nd for Pool 3) and one bottleneck event (*t_m_*) with final size reduction (Nm for Pool 4). |
| 4b | Anatolia  Balkans  W Europe | Human-mediated dispersal of walnut from Anatolia (Pool 1) to the Balkans where natural stands of *J. regia* existed (NG1) promoting the admixture event (Pool 2)*.* Subsequent walnut expansion from Western Europe (Pool 4) to North-Eastern Europe (Pool 3). Final human-induced population size decline in Western Europe. | Pool 1 and NG1 diverged at *t_4_*. Pool 4 diverged from NG1 at *t_3_*. One admixture event between Pool 1 and Pool 4 (*t_2_*) with an admixture rate *ra* given rise to Pool 2. One colonization event from Pool 4 (*t_1_*) with initial size reduction (*t_d_*, Nd for Pool 3) and one bottleneck event (*t_m_*) with final size reduction (Nm for Pool 4). |
| 5b | Anatolia  Balkans  W Europe | Human-mediated dispersal of walnut from Anatolia (Pool 1) to the Balkans where natural stands of *J. regia* existed (NG1) promoting the admixture event (Pool 2)*.* Subsequent walnut expansion from the Balkans to North-Eastern Europe (Pool 3). Final human-induced population size decline in Western Europe (Pool 4). | Pool 1 and NG1 diverged at *t_4_*. Pool 4 diverged from NG1 at *t_3_*. One admixture event between Pool 1 and Pool 4 (*t_2_*) with an admixture rate *ra* given rise to Pool 2. One colonization event from Pool 2 (*t_1_*) with initial size reduction (*t_d_*, Nd for Pool 3) and one bottleneck event (*t_m_*) with final size reduction (Nm for Pool 4). |
| 6b | Anatolia  Balkans  W Europe | Human-mediated dispersal of walnut from Anatolia (Pool 1) to the Balkans where natural stands of *J. regia* existed (NG1) promoting the admixture event (Pool 2)*.* Subsequent walnut dispersal from the Balkans and Western Europe (Pool 4) to North-Eastern Europe promoting the second admixture event (Pool 3). Final human-induced population size expansion in the North-Eastern Europe and population size decline in Western Europe. | Pool 1 and NG1 diverged at *t_4_*. Pool 4 diverged from NG1 at *t_3_*. Two admixture events between Pool 1 and NG1 (*t_2_*) given rise to Pool 2 and between Pool 4 and Pool 2 (*t_3_*) given rise to Pool 3 with an admixture rate *ra* and *rb* respectively. One expansion event (*t_d_*, Nd for Pool 3) and one bottleneck event (*t_m_*) with final size reduction (Nm for Pool 4). |
