# Supplementary material for: Rethinking the history of common walnut (Juglans regia L.) in Europe: Its origins and human interactions
Source: PLoS One. 2017 Mar 3;12(3):e0172541. doi: 10.1371/journal.pone.0172541 (PMC5336217; doi:10.1371/journal.pone.0172541)
Supplement: S5 Table — Total number of alleles (A), the effective number of alleles (Ae), observed (Ho) and expected heterozygosity (HE), polymorphic information content (PIC), and the unbiased estimate of Wright’s fixation indices, within-population inbreeding coefficient f (FIS), total-population inbreeding coefficient F (FIT) and among-population genetic differentiation coefficient θ (FST), among-population genetic differentiation coefficient calculated on allele frequencies adjusted for null allele estimates FST (null) and the estimator of actual differentiation Dest, [29] are shown are for each locus. (DOCX) [file pone.0172541.s008.docx]

**S5 Table. Genetic characterization of 14 microsatellite loci for 91 common walnut populations**. Total number of alleles (A), the effective number of alleles (Ae), observed (Ho) and expected heterozygosity (H_E_), polymorphic information content (PIC), and the unbiased estimate of Wright’s fixation indices, within-population inbreeding coefficient *f* (F_IS_), total-population inbreeding coefficient F (F_IT_) and among-population genetic differentiation coefficient θ (F_ST_), among-population genetic differentiation coefficient calculated on allele frequencies adjusted for null allele estimates F_ST (null)_ and the estimator of actual differentiation D_est_, [25] are shown are for each locus.

| Locus | Size range  (bp) | A | PIC | Ae | H_O_ | H_E_ | *f* (F_IS_) ^a^ | F (F_IT_) ^a^ | *θ* (F_ST_) ^a^ | F_ST (null)_ | D_est_ |
| --- | --- | --- | --- | --- | --- | --- | --- | --- | --- | --- | --- |
|  |  |  |  |  |  |  |  |  |  |  |  |
| WGA1 | 176-194 | 12 | 0.750 | 4.5119 | 0.608 | 0.779 | 0.068** | 0.221** | 0.164** | 0.159 | 0.3633 |
| WGA4 | 231-252 | 7 | 0.569 | 2.6729 | 0.509 | 0.626 | 0.028 | 0.188** | 0.164** | 0.163 | 0.2001 |
| WGA9 | 231-251 | 11 | 0.709 | 4.0061 | 0.656 | 0.751 | 0.005 | 0.127** | 0.122** | 0.120 | 0.2703 |
| WGA27 | 203-212 | 5 | 0.398 | 1.8910 | 0.397 | 0.471 | -0.029 | 0.160** | 0.184** | 0.177 | 0.1426 |
| WGA32 | 157-229 | 40 | 0.817. | 6.0208 | 0.636 | 0.834 | 0.066** | 0.239** | 0.185** | 0.179 | 0.4612 |
| WGA69 | 157-193 | 12 | 0.786 | 5.2809 | 0.530 | 0.811 | 0.239** | 0.347** | 0.142** | 0.131 | 0.3836 |
| WGA72 | 126-184 | 10 | 0.496 | 2.2221 | 0.389 | 0.550 | 0.109** | 0.295** | 0.208** | 0.205 | 0.1970 |
| WGA79 | 198-216 | 9 | 0.578 | 2.7967 | 0.491 | 0.643 | 0.042* | 0.238** | 0.204** | 0.199 | 0.2920 |
| WGA89 | 209-226 | 9 | 0.648 | 3.2753 | 0.574 | 0.695 | 0.018 | 0.176** | 0.160** | 0.156 | 0.2781 |
| WGA118 | 183-293 | 15 | 0.755 | 4.7142 | 0.576 | 0.788 | 0.043** | 0.272** | 0.238** | 0.230 | 0.4593 |
| WGA202 | 248-301 | 25 | 0.833 | 6.6537 | 0.699 | 0.850 | 0.050** | 0.179** | 0.136** | 0.133 | 0.4370 |
| WGA276 | 140-199 | 26 | 0.893 | 10.0166 | 0.750 | 0.900 | 0.027** | 0.169** | 0.146** | 0.141 | 0.5590 |
| WGA321 | 224-249 | 13 | 0.693 | 3.7858 | 0.600 | 0.736 | 0.050** | 0.187** | 0.144** | 0.138 | 0.2701 |
| WGA331 | 270-278 | 5 | 0.580 | 2.8318 | 0.415 | 0.647 | 0.117** | 0.362** | 0.277** | 0.259 | 0.3416 |
| Mean  (SE) | - | 14.214  (9.752) | 0.679  (0.140) | 4.334  (2.165) | 0.559  (0.106) | 0.720  (0.116) | 0.062** | 0.225** | 0.174** | 0.168 | 0.295 |

^a^ Level of significance of unbiased estimate of Wright’s fixation indices were tested using a non-parametric approach described in Excoffier et al., (1992) with 1000 permutations : * = p < 0.05, ** = p < 0.001.
