# Supplementary material for: Rethinking the history of common walnut (Juglans regia L.) in Europe: Its origins and human interactions
Source: PLoS One. 2017 Mar 3;12(3):e0172541. doi: 10.1371/journal.pone.0172541 (PMC5336217; doi:10.1371/journal.pone.0172541)
Supplement: S6 Table — Mean number of alleles per locus (A), effective number of alleles (Ne), allelic richness (Rs) and private allelic richness (PAR) standardized to eight individuals from the original number of trees per population, observed (HO), expected (HE), and unbiased expected heterozygosity (UHE) and inbreeding coefficient (FIS) are shown. (DOCX) [file pone.0172541.s009.docx]

**S6 Table. Genetic diversity of 91 common walnut populations**. Mean number of alleles per locus (A), effective number of alleles (Ne), allelic richness (Rs) and private allelic richness (PAR) standardized to eight individuals from the original number of trees per population, observed (H_O_), expected (H_E_), and unbiased expected heterozygosity (UH_E_) and inbreeding coefficient (F_IS_) are shown.

| Population | A | Ne | Rs | H_o_ | H_E_ | UH_E_ | F_IS_ ^a^ | Private alleles (Population) | | |
| --- | --- | --- | --- | --- | --- | --- | --- | --- | --- | --- |
|  |  |  |  |  |  |  |  | Locus (N ^b^) |  | PAR |
|  |  |  |  |  |  |  |  |  |  |  |
| 1-TEREK | 5.314 | 2.580 | 3.570 | 0.548 | 0.555 | 0.561 | 0.024 |  |  | 0.00 |
| 2-SHARAP | 4.214 | 2.743 | 3.620 | 0.599 | 0.576 | 0.592 | -0.012 |  |  | 0.00 |
| 3-YARADAR | 3.571 | 2.431 | 3.160 | 0.487 | 0.523 | 0.540 | 0.102* |  |  | 0.01 |
| 4-SHAIDAN | 4.571 | 2.372 | 3.390 | 0.518 | 0.525 | 0.536 | 0.034 |  |  | 0.00 |
| 5-KYZYL | 4.214 | 2.185 | 3.040 | 0.484 | 0.492 | 0.498 | 0.028 |  |  | 0.00 |
| 6-KATAR | 3.643 | 2.422 | 3.160 | 0.511 | 0.518 | 0.532 | 0.040 |  |  | 0.00 |
| 7-KYOK | 3.786 | 2.551 | 3.270 | 0.529 | 0.546 | 0.557 | 0.052 | WGA202(1) |  | 0.02 |
| 8-KYR | 4.071 | 2.629 | 3.370 | 0.529 | 0.560 | 0.571 | 0.075 |  |  | 0.01 |
| 9-TERS | 4.500 | 2.752 | 3.540 | 0.592 | 0.582 | 0.594 | 0.004 |  |  | 0.00 |
| 10-KAMCHIK | 4.071 | 2.683 | 3.520 | 0.528 | 0.575 | 0.591 | 0.110* |  |  | 0.00 |
| 11-YAKKATUT | 4.429 | 2.998 | 3.780 | 0.536 | 0.602 | 0.620 | 0.139** |  |  | 0.00 |
| 12-SIDJAK | 3.714 | 2.403 | 3.510 | 0.586 | 0.542 | 0.571 | -0.028 |  |  | 0.01 |
| 13-CHARVAK | 4.875 | 2.836 | 3.870 | 0.540 | 0.598 | 0.615 | 0.126** | WGA72(2) |  | 0.06 |
| 14-NANAI | 5.000 | 3.072 | 4.120 | 0.587 | 0.610 | 0.628 | 0.066 | WGA72(1) |  | 0.06 |
| 15- DJARKU | 4.786 | 3.052 | 4.120 | 0.563 | 0.622 | 0.642 | 0.127** |  |  | 0.00 |
| 16-BOGUSTAN | 4.714 | 2.877 | 3.780 | 0.554 | 0.578 | 0.593 | 0.068 |  |  | 0.00 |
| 17-BOSTANLYK | 5.429 | 3.584 | 4.440 | 0.567 | 0.643 | 0.662 | 0.146** | WGA276(1) |  | 0.04 |
| 18-BAKHMAL | 5.214 | 3.293 | 4.410 | 0.595 | 0.635 | 0.657 | 0.097* |  |  | 0.00 |
| 19-KARANKUL | 6.857 | 3.889 | 4.710 | 0.623 | 0.699 | 0.709 | 0.123** | WGA276(1) |  | 0.03 |
| 20-FARISH | 5.071 | 3.239 | 4.150 | 0.529 | 0.601 | 0.622 | 0.122** |  |  | 0.00 |
| 21-ANDIGEN | 3.571 | 2.468 | 3.290 | 0.583 | 0546 | 0.570 | -0.025 |  |  | 0.00 |
| 22-KATTA | 5.571 | 2.983 | 4.080 | 0.586 | 0.612 | 0.620 | 0.055 |  |  | 0.00 |
| 23-KHAYAT | 4.214 | 2.860 | 3.810 | 0.594 | 0.587 | 0.606 | 0.022 |  |  | 0.00 |
| 24-YAMCHI | 3.929 | 2.807 | 3.750 | 0.571 | 0.552 | 0.581 | 0.018 |  |  | 0.00 |
| 25-KARRI | 4.429 | 2.728 | 3.740 | 0.589 | 0.571 | 0.586 | -0.006 |  |  | 0.00 |
| 26-MADJERUM | 5.000 | 2.589 | 3.820 | 0.548 | 0.560 | 0.571 | 0.040 |  |  | 0.00 |
| 27-GUILI-1 | 4.643 | 2.610 | 3.370 | 0.494 | 0.515 | 0.522 | 0.056 |  |  | 0.00 |
| 28-GUILI-2 | 6.071 | 3.121 | 4.100 | 0.464 | 0.638 | 0.643 | 0.280** | WGA4(1), WGA276(1) |  | 0.07 |
| 29-GUILI-3 | 5.000 | 2.742 | 3.880 | 0.519 | 0.588 | 0.599 | 0.137** |  |  | 0.01 |
| 30-URUMQI | 5.214 | 2.710 | 3.900 | 0.579 | 0.588 | 0.599 | 0.034 |  |  | 0.01 |
| 31-SUNBE | 3.000 | 2.024 | 2.680 | 0.425 | 0.439 | 0.451 | 0.060 |  |  | 0.00 |
| 32-DASH | 5.929 | 3.029 | 3.980 | 0.542 | 0.596 | 0.602 | 0.101** | WGA9(1), WGA69(1), WGA79(1) |  | 0.01 |
| 33-GILGIT | 6.500 | 3.941 | 4.870 | 0.609 | 0.658 | 0.674 | 0.099* |  |  | 0.011 |
| 34-HUNZA | 6.786 | 3.887 | 4.860 | 0.611 | 0.679 | 0.693 | 0.120** | WGA72(1), WGA79(2), WGA321(1) |  | 0.03 |
| 35-SHOULI | 5.214 | 3.226 | 4.340 | 0.705 | 0.658 | 0.680 | -0.039 |  |  | 0.00 |
| 36-KARAJ | 4.000 | 2.925 | 3.700 | 0.661 | 0.618 | 0.645 | -0.026 | WGA9(1), WGA32(2), WGA118(4) |  | 0.35 |
| 37-LAGO | 5.071 | 3.502 | 4.490 | 0.619 | 0.665 | 0.688 | 0.103* |  |  | 0.07 |
| 38-SKRA | 5.214 | 3.407 | 4.380 | 0.592 | 0.645 | 0.660 | 0.106** | WGA89(1) |  | 0.08 |
| 39-ANATOLIA | 4.857 | 3.030 | 4.030 | 0.519 | 0.611 | 0.628 | 0.177** |  |  | 0.01 |
| 40-TRABZON | 5.714 | 3.303 | 4.520 | 0.565 | 0.638 | 0.653 | 0.137** |  |  | 0.04 |
| 41-PAIKO_A | 4.857 | 3.389 | 4.040 | 0.539 | 0.634 | 0.647 | 0.170** | WGA1(1), WGA321(1) |  | 0.08 |
| 42-PAIKO_B | 4.714 | 3.316 | 3.930 | 0.577 | 0.625 | 0.637 | 0.096** |  |  | 0.04 |
| 43-ARCADIA | 3.929 | 2.540 | 3.460 | 0.543 | 0.574 | 0.588 | 0.079* |  |  | 0.03 |
| 44-CHANIA | 4.500 | 2.427 | 3.380 | 0.459 | 0.539 | 0.547 | 0.163** | WGA1(1) |  | 0.06 |
| 45-BRASOV | 6.000 | 3.734 | 4.510 | 0.635 | 0.662 | 0.675 | 0.060* |  |  | 0.02 |
| 46-CHISINAU | 5.286 | 3.576 | 4.180 | 0.664 | 0.659 | 0.669 | 0.007 |  |  | 0.02 |
| 47-CTSATALJA | 5.000 | 3.575 | 3.990 | 0.607 | 0.641 | 0.651 | 0.068* |  |  | 0.03 |
| 48-MELYKUT | 4.286 | 2.608 | 3.370 | 0.563 | 0.566 | 0.575 | 0.021 | WGA202(1) |  | 0.06 |
| 49-PECS | 4.143 | 2.782 | 3.380 | 0.530 | 0.580 | 0.590 | 0.102** |  |  | 0.01 |
| 50-DUNAVA | 4.643 | 3.050 | 3.770 | 0.579 | 0.611 | 0.627 | 0.078 |  |  | 0.02 |
| 51-MILOTA | 3.643 | 2.519 | 3.220 | 0.507 | 0.550 | 0.564 | 0.103* |  |  | 0.00 |
| 52-NAGYAR | 3.857 | 2.518 | 3.150 | 0.602 | 0.562 | 0.571 | -0.055 |  |  | 0.01 |
| 53-TISZAKOROD | 4.214 | 2.870 | 3.560 | 0.579 | 0.601 | 0.612 | 0.055 |  |  | 0.00 |
| 54-VASARO | 4.143 | 2.952 | 3.570 | 0.618 | 0.614 | 0.629 | 0.018 | WGA118(1) |  | 0.06 |
| 55-BONY | 4.000 | 2.904 | 3.380 | 0.622 | 0.617 | 0.626 | 0.005 |  |  | 0.00 |
| 56-MOSONM | 3.643 | 2.607 | 3.120 | 0.575 | 0.587 | 0.602 | 0.046 |  |  | 0.00 |
| 57-DEDINA | 5.286 | 3.064 | 3.830 | 0.561 | 0.614 | 0.624 | 0.101** | WGA32(1) |  | 0.04 |
| 58-ORLEAN | 3.071 | 2.367 | 2.850 | 0.571 | 0.549 | 0.567 | -0.007 |  |  | 0.00 |
| 59-POITIERS | 3.500 | 2.039 | 2.770 | 0.403 | 0.474 | 0.482 | 0.165** | WGA118(1) |  | 0.02 |
| 60-PUYDOME | 3.571 | 2.508 | 2.920 | 0.506 | 0.571 | 0.577 | 0.123** |  |  | 0.00 |
| 61-CHAMBERY | 3.714 | 2.400 | 2.900 | 0.536 | 0.554 | 0.560 | 0.043 |  |  | 0.00 |
| 62-GIRONA | 3.929 | 2.766 | 3.540 | 0.579 | 0.593 | 0.608 | 0.049 |  |  | 0.03 |
| 63-OSIGO | 3.500 | 2.501 | 3.190 | 0.593 | 0.571 | 0.594 | 0.000 |  |  | 0.01 |
| 64-PORD | 3.357 | 2.483 | 3.220 | 0.557 | 0.550 | 0.579 | 0.039 |  |  | 0.00 |
| 65-PREONE | 3.429 | 2.490 | 3.210 | 0.536 | 0.577 | 0.602 | 0.114 |  |  | 0.00 |
| 66-GABRIA | 3.143 | 2.451 | 3.060 | 0.600 | 0.570 | 0.600 | 0.000 |  |  | 0.00 |
| 67-GIORGIO | 3.500 | 2.517 | 3.150 | 0.519 | 0.570 | 0.590 | 0.124* |  |  | 0.01 |
| 68-SABINA | 2.857 | 2.266 | 2.860 | 0.491 | 0.546 | 0.583 | 0.166* |  |  | 0.00 |
| 69-PESC | 3.429 | 2.525 | 3.080 | 0.600 | 0.577 | 0.592 | -0.014 |  |  | 0.00 |
| 70-ALF | 3.357 | 2.552 | 3.080 | 0.544 | 0.563 | 0.579 | 0.062 |  |  | 0.00 |
| 71-BARREA | 3.357 | 2.407 | 2.940 | 0.616 | 0.546 | 0.560 | -0.102 |  |  | 0.00 |
| 72-VALCO | 3.071 | 2.373 | 2.980 | 0.564 | 0.539 | 0.567 | 0.004 |  |  | 0.00 |
| 73-RIONERO | 2.857 | 2.291 | 2.860 | 0.563 | 0.530 | 0.565 | 0.004 | WGA276(1) |  | 0.07 |
| 74-SANNIO | 3.286 | 2.464 | 2.980 | 0.600 | 0.549 | 0.563 | -0.066 |  |  | 0.00 |
| 75-MIRA | 3.357 | 2.457 | 3.130 | 0.571 | 0.553 | 0.574 | 0.004 |  |  | 0.00 |
| 76-FONT | 3.357 | 2.427 | 2.970 | 0.586 | 0.547 | 0.561 | -0.044 |  |  | 0.00 |
| 77-MAS | 3.286 | 2.342 | 2.890 | 0.646 | 0.539 | 0.553 | -0.174 |  |  | 0.00 |
| 78-ALTILIA | 3.286 | 2.491 | 3.010 | 0.618 | 0.576 | 0.591 | -0.046 |  |  | 0.00 |
| 79-CROCE | 3.214 | 2.321 | 3.040 | 0.591 | 0.530 | 0.555 | -0.068 |  |  | 0.00 |
| 80-CIRCE | 3.357 | 2.414 | 3.060 | 0.557 | 0.547 | 0.561 | 0.007 |  |  | 0.00 |
| 81-CAVOTI | 3.357 | 2.445 | 2.990 | 0.534 | 0.556 | 0.566 | 0.056 |  |  | 0.01 |
| 82-MOLARA | 2.786 | 2.036 | 2.790 | 0.482 | 0.481 | 0.513 | 0.064 |  |  | 0.00 |
| 83-MONTEC | 3.571 | 2.647 | 3.150 | 0.591 | 0.587 | 0.599 | 0.013 |  |  | 0.00 |
| 84-ARIANO | 3.429 | 2.389 | 2.980 | 0.640 | 0.560 | 0.572 | -0.121 |  |  | 0.00 |
| 85-CASOLLA | 2.929 | 2.236 | 2.890 | 0.557 | 0.535 | 0.563 | 0.011 |  |  | 0.00 |
| 86-TUFINO | 2.929 | 2.432 | 2.890 | 0.529 | 0.540 | 0.568 | 0.073 |  |  | 0.01 |
| 87-SERINO | 3.071 | 2.223 | 2.960 | 0.543 | 0.522 | 0.549 | 0.012 | WGA32(1) |  | 0.06 |
| 88-MONT | 3.357 | 2.734 | 3.090 | 0.654 | 0.603 | 0.618 | -0.058 | WGA32(1) |  | 0.03 |
| 89-RAGUSA | 2.857 | 2.389 | 2.830 | 0.586 | 0.527 | 0.555 | -0.058 |  |  | 0.00 |
| 90-ANAPO | 3.143 | 2.391 | 3.030 | 0.543 | 0.530 | 0.558 | 0.029 |  |  | 0.00 |
| 91-BIVONA | 2.929 | 2.292 | 2.860 | 0.507 | 0.510 | 0.537 | 0.058 |  |  | 0.00 |
|  |  |  |  |  |  |  |  |  |  |  |
