# Supplementary material for: Rethinking the history of common walnut (Juglans regia L.) in Europe: Its origins and human interactions
Source: PLoS One. 2017 Mar 3;12(3):e0172541. doi: 10.1371/journal.pone.0172541 (PMC5336217; doi:10.1371/journal.pone.0172541)
Supplement: S7 Table — Wilcoxon’s signed-rank’ test [35], shifted allele distribution analysis [37] and the M-ratio test [38] for each walnut population are reported. (DOCX) [file pone.0172541.s010.docx]

**S7 Table. Bottleneck analysis of 91 common walnut populations sampled across Eurasia using 14 SSR markers**. Wilcoxon’s signed-rank’ test [31], shifted allele distribution analysis [33] and the M-ratio test [34] for each walnut population are reported.

| Population | Wilcoxon signed rank test ^a^ | | L-shape ^b^ | M ratio value ^c^ |
| --- | --- | --- | --- | --- |
|  | Heterozygote excess | Heterozygote deficiency |  |  |
|  |  |  |  |  |
| 1-TEREK | 0.94055 | 0.06763 | no deviation | 0.34504 ‡ |
| 2-SHARAP | 0.27081 | 0.74921 | no deviation | 0.34722‡ |
| 3-YARADAR | 0.14789 | 0.86621 | no deviation | 0.33148‡ |
| 4-SHAIDAN | 0.97528 | 0.02899* | no deviation | 0.32650‡ |
| 5-KYZYL | 0.96619 | 0.03925* | no deviation | 0.34735‡ |
| 6-KATAR | 0.20715 | 0.81213 | no deviation | 0.30182‡ |
| 7-KYOK | 0.10828 | 0.90314 | no deviation | 0.33330‡ |
| 8-KYR | 0.13379 | 0.87939 | no deviation | 0.34607‡ |
| 9-TERS | 0.35742 | 0.66510 | no deviation | 0.34888‡ |
| 10-KAMCHIK | 0.12061 | 0.89172 | no deviation | 0.35291‡ |
| 11-YAKKATUT | 0.10828 | 0.90314 | no deviation | 0.31242‡ |
| 12-SIDJAK | 0.47577 | 0.54840 | no deviation | 0.33313‡ |
| 13-CHARVAK | 0.82123 | 0.19550 | no deviation | 0.30347‡ |
| 14-NANAI | 0.45160 | 0.57239 | no deviation | 0.31648‡ |
| 15- DJARKU | 0.29150 | 0.72919 | no deviation | 0.35333‡ |
| 16-BOGUSTAN | 0.72919 | 0.29150 | no deviation | 0.35399‡ |
| 17-BOSTANLYK | 0.01764 * | 0.98523 | deviation | 0.35438‡ |
| 18-BAKHMAL | 0.64258 | 0.38043 | no deviation | 0.36093‡ |
| 19-KARANKUL | 0.52423 | 0.50000 | no deviation | 0.36718‡ |
| 20-FARISH | 0.64258 | 0.38043 | no deviation | 0.34225‡ |
| 21-ANDIGEN | 0.14789 | 0.86621 | no deviation | 0.30209‡ |
| 22-KATTA | 0.78687 | 0.23157 | no deviation | 0.34933‡ |
| 23-KHAYAT | 0.1082 | 0.90314 | no deviation | 0.33315‡ |
| 24-YAMCHI | 0.36768 | 0.65759 | no deviation | 0.34145‡ |
| 25-KARRI | 0.72919 | 0.29150 | no deviation | 0.32298‡ |
| 26-MADJERUM | 0.95471 | 0.05200 | no deviation | 0.34340‡ |
| 27-GUILI-1 | 0.85211 | 0.16290 | no deviation | 0.32711‡ |
| 28-GUILI-2 | 0.74921 | 0.27081 | no deviation | 0.33713‡ |
| 29-GUILI-3 | 0.86621 | 0.14789 | no deviation | 0.34097‡ |
| 30-URUMQI | 0.93237 | 0.07654 | no deviation | 0.33898‡ |
| 31-SUNBE | 0.34241 | 0.68225 | no deviation | 0.28185‡ |
| 32-DASH | 0.94055 | 0.06763 | no deviation | 0.35321‡ |
| 33-GILGIT | 0.80450 | 0.21313 | no deviation | 0.35823‡ |
| 34-HUNZA | 0.85211 | 0.16290 | no deviation | 0.32532‡ |
| 35-SHOULI | 0.52423 | 0.50000 | no deviation | 0.31404‡ |
| 36-KARAJ | 0.01764* | 0.98523 | deviation | 0.28117‡ |
| 37-LAGO | 0.10828 | 0.90314 | no deviation | 0.29291‡ |
| 38-SKRA | 0.29150 | 0.72919 | no deviation | 0.33511‡ |
| 39-ANATOLIA | 0.38043 | 0.64258 | no deviation | 0.32629‡ |
| 40-TRABZON | 0.85211 | 0.16290 | no deviation | 0.33159‡ |
| 41-PAIKO_A | 0.00671 * | 0.99731 | no deviation | 0.37989‡ |
| 42-PAIKO_B | 0.01764 * | 0.98523 | no deviation | 0.34142‡ |
| 43-ARCADIA | 0.27081 | 0.74921 | no deviation | 0.34801‡ |
| 44-CHANIA | 0.76843 | 0.25079 | no deviation | 0.37792‡ |
| 45-BRASOV | 0.05945 | 0.94800 | no deviation | 0.34609‡ |
| 46-CHISINAU | 0.01764* | 0.98523 | no deviation | 0.34789‡ |
| 47-CTSATALJA | 0.00168 ** | 0.99869 | no deviation | 0.36079‡ |
| 48-MELYKUT | 0.25079 | 0.76843 | no deviation | 0.37543‡ |
| 49-PECS | 0.07654 | 0.93237 | no deviation | 0.31177‡ |
| 50-DUNAVA | 0.07654 | 0.93237 | no deviation | 0.35559‡ |
| 51-MILOTA | 0.08630 | 0.92346 | no deviation | 0.34079‡ |
| 52-NAGYAR | 0.07654 | 0.93237 | no deviation | 0.35239‡ |
| 53-TISZAKOROD | 0.01477* | 0.98773 | no deviation | 0.35142‡ |
| 54-VASARO | 0.00671 ** | 0.99731 | no deviation | 0.33308‡ |
| 55-BONY | 0.00015** | 0.99991 | no deviation | 0.36879‡ |
| 56-MOSONM | 0.00168** | 0.99869 | no deviation | 0.32806‡ |
| 57-DEDINA | 0.40387 | 0.61957 | no deviation | 0.37624‡ |
| 58-ORLEAN | 0.00269 ** | 0.99786 | deviation | 0.34505‡ |
| 59-POITIERS | 0.35742 | 0.66510 | no deviation | 0.32387‡ |
| 60-PUYDOME | 0.00830 ** | 0.99329 | no deviation | 0.35661‡ |
| 61-CHAMBERY | 0.03925* | 0.96619 | no deviation | 0.35983‡ |
| 62-GIRONA | 0.04529* | 0.96075 | no deviation | 0.33418‡ |
| 63-OSIGO | 0.04529* | 0.96075 | no deviation | 0.35268‡ |
| 64-PORD | 0.04529 | 0.96075 | no deviation | 0.35151‡ |
| 65-PREONE | 0.00830** | 0.99329 | deviation | 0.34504‡ |
| 66-GABRIA | 0.00671** | 0.99731 | deviation | 0.33735‡ |
| 67-GIORGIO | 0.01477* | 0.98773 | no deviation | 0.36334‡ |
| 68-SABINA | 0.01477* | 0.98773 | deviation | 0.34543‡ |
| 69-PESC | 0.00214 ** | 0.99832 | deviation | 0.36837‡ |
| 70-ALF | 0.00101 ** | 0.99924 | no deviation | 0.34435‡ |
| 71-BARREA | 0.02094 * | 0.98236 | no deviation | 0.32384‡ |
| 72-VALCO | 0.02899 * | 0.97528 | deviation | 0.37939‡ |
| 73-RIONERO | 0.03925* | 0.96619 | deviation | 0.37591‡ |
| 74-SANNIO | 0.00830 ** | 0.99329 | no deviation | 0.35387‡ |
| 75-MIRA | 0.00671** | 0.99731 | deviation | 0.35203‡ |
| 76-FONT | 0.02094* | 0.98236 | no deviation | 0.36365‡ |
| 77-MAS | 0.02472 * | 0.97906 | no deviation | 0.34016‡ |
| 78-ALTILIA | 0.00043 ** | 0.99969 | deviation | 0.34393‡ |
| 79-CROCE | 0.03381* | 0.97101 | no deviation | 0.34648‡ |
| 80-CIRCE | 0.00269 ** | 0.99786 | no deviation | 0.37151‡ |
| 81-CAVOTI | 0.00168 ** | 0.99869 | no deviation | 0.32147‡ |
| 82-MOLARA | 0.23157 | 0.78687 | no deviation | 0.33244‡ |
| 83-MONTEC | 0.00671** | 0.99731 | no deviation | 0.34703‡ |
| 84-ARIANO | 0.02094 * | 0.98236 | no deviation | 0.33919‡ |
| 85-CASOLLA | 0.0013 ** | 0.99899 | deviation | 0.34632‡ |
| 86-TUFINO | 0.00058 ** | 0.99957 | deviation | 0.33057‡ |
| 87-SERINO | 0.17877 | 0.83710 | no deviation | 0.33551‡ |
| 88-MONT | 0.00003** | 1.00000 | deviation | 0.35593‡ |
| 89-RAGUSA | 0.00018** | 0.99988 | deviation | 0.32682‡ |
| 90-ANAPO | 0.06360 | 0.94507 | deviation | 0.32372‡ |
| 91-BIVONA | 0.03925* | 0.96619 | deviation | 0.37036‡ |
|  |  |  |  |  |

^a^ Significance of heterozygote excess and/or deficiency according to the Wilcoxon signed rank test under the Two-Phase Model of loci (TPM) for each walnut population: * = p < 0.05; **= p < 0.01

^b^ Deviation of allele frequency classes from a normal L-shaped.

^c^ ‡ M ratio value significantly smaller than the critical value 0.68.
