# Supplementary material for: Rethinking the history of common walnut (Juglans regia L.) in Europe: Its origins and human interactions
Source: PLoS One. 2017 Mar 3;12(3):e0172541. doi: 10.1371/journal.pone.0172541 (PMC5336217; doi:10.1371/journal.pone.0172541)
Supplement: S8 Table — Percentage of membership (admixture proportion-Q) of each predefined common walnut population in each of the four (K = 4) clusters, two (K’ = 4) sub-clusters for cluster 1, two (K” = 2) sub-clusters for cluster 2 and two (K”‘ = 2) sub-clusters for cluster 4 inferred by Bayesian approach using STRUCTURE software (Pritchard et al., 2000). Q-values greater than 0.75 are reported in bold. (DOCX) [file pone.0172541.s011.docx]

**S8 Table. Mean percentage of membership (Qi) of each common walnut population inferred by STRUCTURE**. Percentage of membership (admixture proportion-*Q*) of each predefined common walnut population in each of the four (K = 4) clusters, two (K’ = 4) sub-clusters for cluster 1, two (K’’ = 2) sub-clusters for cluster 2 and two (K’’’ = 2) sub-clusters for cluster 4 inferred by Bayesian approach using STRUCTURE software (Pritchard *et al*., 2000). *Q*-values greater than 0.75 are reported in bold.

|  |  | K 4 | | | |  | K’ 4 | | | |  | K’’ 2 | |  | K’’’ 2 | |
| --- | --- | --- | --- | --- | --- | --- | --- | --- | --- | --- | --- | --- | --- | --- | --- | --- |
| ID |  | Q1 | Q2 | Q3 | Q4 |  | Q1 | Q2 | Q3 | Q4 |  | Q1 | Q2 |  | Q1 | Q2 |
| 1-TEREK |  | 0.0070 | 0.0170 | **0.9200** | 0.0560 |  |  |  |  |  |  |  |  |  |  |  |
| 2-SHARAP |  | 0.0070 | 0.0210 | **0.9660** | 0.0060 |  |  |  |  |  |  |  |  |  |  |  |
| 3-YARADAR |  | 0.0115 | 0.0108 | **0.9692** | 0.0085 |  |  |  |  |  |  |  |  |  |  |  |
| 4-SHAIDAN |  | 0.0050 | 0.0060 | **0.9440** | 0.0450 |  |  |  |  |  |  |  |  |  |  |  |
| 5-KYZYL |  | 0.0042 | 0.0070 | **0.9848** | 0.0040 |  |  |  |  |  |  |  |  |  |  |  |
| 6-KATAR |  | 0.0078 | 0.0060 | **0.9832** | 0.0030 |  |  |  |  |  |  |  |  |  |  |  |
| 7-KYOK |  | 0.0200 | 0.0097 | **0.9653** | 0.0050 |  |  |  |  |  |  |  |  |  |  |  |
| 8-KYR |  | 0.0050 | 0.0110 | **0.9800** | 0.0040 |  |  |  |  |  |  |  |  |  |  |  |
| 9-TERS |  | 0.0070 | 0.0125 | **0.9763** | 0.0042 |  |  |  |  |  |  |  |  |  |  |  |
| 10-KAMCHIK |  | 0.0213 | 0.0275 | **0.9285** | 0.0227 |  |  |  |  |  |  |  |  |  |  |  |
| 11-YAKKATUT |  | 0.0350 | 0.1075 | **0.8525** | 0.0050 |  |  |  |  |  |  |  |  |  |  |  |
| 12-SIDJAK |  | 0.0148 | 0.1673 | **0.8118** | 0.0060 |  |  |  |  |  |  |  |  |  |  |  |
| 13-CHARVAK |  | 0.0528 | 0.1525 | **0.7856** | 0.0090 |  |  |  |  |  |  |  |  |  |  |  |
| 14-NANAI |  | 0.0445 | 0.2730 | 0.6694 | 0.0130 |  |  |  |  |  |  |  |  |  |  |  |
| 15- DJARKU |  | 0.0513 | 0.2013 | 0.7118 | 0.0355 |  |  |  |  |  |  |  |  |  |  |  |
| 16-BOGUSTAN |  | 0.0150 | 0.2994 | 0.6816 | 0.0040 |  |  |  |  |  |  |  |  |  |  |  |
| 17-BOSTANLYK |  | 0.0180 | 0.3718 | 0.5962 | 0.0140 |  |  |  |  |  |  |  |  |  |  |  |
| 18-BAKHMAL |  | 0.0763 | 0.3740 | 0.4987 | 0.0510 |  |  |  |  |  |  |  |  |  |  |  |
| 19-KARANKUL |  | 0.6842 | 0.1257 | 0.1650 | 0.0252 |  | 0.039 | 0.0475 | 0.077 | **0.8365** |  |  |  |  |  |  |
| 20-FARISH |  | 0.0087 | **0.7639** | 0.2224 | 0.0050 |  |  |  |  |  |  | 0.1860 | **0.8140** |  |  |  |
| 21-ANDIGEN |  | 0.0060 | **0.9850** | 0.0050 | 0.0040 |  |  |  |  |  |  | 0.0040 | **0.9960** |  |  |  |
| 22-KATTA |  | 0.0160 | **0.9535** | 0.0135 | 0.0170 |  |  |  |  |  |  | 0.0278 | **0.9722** |  |  |  |
| 23-KHAYAT |  | 0.0135 | **0.9762** | 0.0053 | 0.0050 |  |  |  |  |  |  | 0.0123 | **0.9877** |  |  |  |
| 24-YAMCHI |  | 0.0080 | **0.9687** | 0.0183 | 0.0050 |  |  |  |  |  |  | 0.0213 | **0.9787** |  |  |  |
| 25-KARRI |  | 0.0090 | **0.9785** | 0.0065 | 0.0060 |  |  |  |  |  |  | 0.0080 | **0.9920** |  |  |  |
| 26-MADJERUM |  | 0.0053 | **0.9825** | 0.0072 | 0.0050 |  |  |  |  |  |  | 0.0110 | **0.9890** |  |  |  |
| 27-GUILI-1 |  | 0.0805 | **0.8731** | 0.0188 | 0.0275 |  |  |  |  |  |  | **0.9852** | 0.0148 |  |  |  |
| 28-GUILI-2 |  | 0.1508 | 0.4027 | 0.4395 | 0.0070 |  | 0.025 | 0.006 | **0.9403** | 0.0287 |  | **0.9860** | 0.0140 |  |  |  |
| 29-GUILI-3 |  | 0.0312 | **0.8442** | 0.0967 | 0.0280 |  |  |  |  |  |  | **0.9890** | 0.0110 |  |  |  |
| 30-URUMQI |  | 0.0684 | **0.8461** | 0.0619 | 0.0237 |  |  |  |  |  |  | **0.9695** | 0.0305 |  |  |  |
| 31-SUNBE |  | 0.0225 | **0.8590** | 0.0992 | 0.0193 |  |  |  |  |  |  | **0.9960** | 0.0040 |  |  |  |
| 32-DASH |  | **0.9487** | 0.0333 | 0.0070 | 0.0110 |  | 0.0163 | 0.0137 | **0.9558** | 0.0142 |  |  |  |  |  |  |
| 33-GILGIT |  | **0.7612** | 0.1595 | 0.0148 | 0.0645 |  | 0.7388 | 0.0100 | 0.0123 | 0.2388 |  |  |  |  |  |  |
| 34-HUNZA |  | **0.9023** | 0.0412 | 0.0475 | 0.0090 |  | **0.9467** | 0.0148 | 0.0272 | 0.0113 |  |  |  |  |  |  |
| 35-SHOULI |  | 0.2771 | 0.0940 | 0.1709 | 0.4580 |  |  |  |  |  |  |  |  |  |  |  |
| 36-KARAJ |  | **0.8227** | 0.1140 | 0.0242 | 0.0392 |  | 0.2555 | 0.0157 | 0.006 | 0.7229 |  |  |  |  |  |  |
| 37-LAGO |  | **0.9570** | 0.0120 | 0.0227 | 0.0083 |  | 0.0122 | **0.9553** | 0.0225 | 0.0100 |  |  |  |  |  |  |
| 38-SKRA |  | **0.9489** | 0.0345 | 0.0110 | 0.0057 |  | 0.0135 | **0.9520** | 0.0170 | 0.0175 |  |  |  |  |  |  |
| 39-ANATOLIA |  | **0.9068** | 0.0112 | 0.0620 | 0.0200 |  | 0.0473 | 0.0205 | 0.0100 | **0.9222** |  |  |  |  |  |  |
| 40-TRABZON |  | **0.9308** | 0.0173 | 0.0278 | 0.0240 |  | 0.0538 | 0.2222 | 0.0530 | 0.6710 |  |  |  |  |  |  |
| 41-PAIKO_A |  | 0.7112 | 0.0150 | 0.0090 | 0.2648 |  |  |  |  |  |  |  |  |  |  |  |
| 42-PAIKO_B |  | 0.7420 | 0.0187 | 0.0063 | 0.2230 |  |  |  |  |  |  |  |  |  |  |  |
| 43-ARCADIA |  | 0.3101 | 0.0167 | 0.0070 | 0.6663 |  |  |  |  |  |  |  |  |  |  |  |
| 44-CHANIA |  | 0.2112 | 0.0180 | 0.0060 | **0.7648** |  |  |  |  |  |  |  |  |  | 0.3143 | 0.6857 |
| 45-BRASOV |  | 0.5001 | 0.0148 | 0.0467 | 0.4384 |  |  |  |  |  |  |  |  |  |  |  |
| 46-CHISINAU |  | 0.6464 | 0.0150 | 0.0050 | 0.3336 |  |  |  |  |  |  |  |  |  |  |  |
| 47-CTSATALJA |  | 0.1208 | 0.0120 | 0.0050 | **0.8622** |  |  |  |  |  |  |  |  |  | 0.0847 | **0.9153** |
| 48-MELYKUT |  | 0.0357 | 0.0060 | 0.0070 | **0.9513** |  |  |  |  |  |  |  |  |  | 0.3923 | 0.6077 |
| 49-PECS |  | 0.0170 | 0.0040 | 0.0030 | **0.9760** |  |  |  |  |  |  |  |  |  | 0.3578 | 0.6422 |
| 50-DUNAVA |  | 0.0447 | 0.0080 | 0.0060 | **0.9413** |  |  |  |  |  |  |  |  |  | 0.1175 | **0.8825** |
| 51-MILOTA |  | 0.0697 | 0.0060 | 0.0050 | **0.9193** |  |  |  |  |  |  |  |  |  | 0.0333 | **0.9667** |
| 52-NAGYAR |  | 0.0533 | 0.0080 | 0.0050 | **0.9337** |  |  |  |  |  |  |  |  |  | 0.0252 | **0.9748** |
| 53-TISZAKOROD |  | 0.0557 | 0.0050 | 0.0040 | **0.9353** |  |  |  |  |  |  |  |  |  | 0.1137 | **0.8863** |
| 54-VASARO |  | 0.0822 | 0.0067 | 0.0030 | **0.9082** |  |  |  |  |  |  |  |  |  | 0.1985 | **0.8015** |
| 55-BONY |  | 0.0182 | 0.0102 | 0.0050 | **0.9666** |  |  |  |  |  |  |  |  |  | 0.6763 | 0.3237 |
| 56-MOSONM |  | 0.0080 | 0.0040 | 0.0043 | **0.9837** |  |  |  |  |  |  |  |  |  | 0.6728 | 0.3272 |
| 57-DEDINA |  | 0.0927 | 0.0152 | 0.0080 | **0.8841** |  |  |  |  |  |  |  |  |  | 0.3733 | 0.6267 |
| 58-ORLEAN |  | 0.0057 | 0.0030 | 0.0030 | **0.9883** |  |  |  |  |  |  |  |  |  | **0.8622** | 0.1378 |
| 59-POITIERS |  | 0.0075 | 0.0220 | 0.0030 | **0.9675** |  |  |  |  |  |  |  |  |  | **0.7730** | 0.2270 |
| 60-PUYDOME |  | 0.0047 | 0.0040 | 0.0030 | **0.9883** |  |  |  |  |  |  |  |  |  | **0.8203** | 0.1797 |
| 61-CHAMBERY |  | 0.0043 | 0.0040 | 0.0030 | **0.9887** |  |  |  |  |  |  |  |  |  | **0.8285** | 0.1715 |
| 62-GIRONA |  | 0.0853 | 0.0188 | 0.0670 | **0.8288** |  |  |  |  |  |  |  |  |  | 0.6938 | 0.3062 |
| 63-OSIGO |  | 0.0062 | 0.0155 | 0.0050 | **0.9733** |  |  |  |  |  |  |  |  |  | **0.7823** | 0.2177 |
| 64-PORD |  | 0.0060 | 0.0035 | 0.0040 | **0.9865** |  |  |  |  |  |  |  |  |  | 0.5810 | 0.4190 |
| 65-PREONE |  | 0.0260 | 0.0072 | 0.0100 | **0.9568** |  |  |  |  |  |  |  |  |  | 0.6675 | 0.3325 |
| 66-GABRIA |  | 0.0080 | 0.0032 | 0.0030 | **0.9858** |  |  |  |  |  |  |  |  |  | **0.7788** | 0.2212 |
| 67-GIORGIO |  | 0.0102 | 0.0040 | 0.0040 | **0.9818** |  |  |  |  |  |  |  |  |  | **0.8392** | 0.1608 |
| 68-SABINA |  | 0.0060 | 0.0040 | 0.0030 | **0.9870** |  |  |  |  |  |  |  |  |  | **0.7933** | 0.2067 |
| 69-PESC |  | 0.0208 | 0.0060 | 0.0030 | **0.9702** |  |  |  |  |  |  |  |  |  | **0.7977** | 0.2023 |
| 70-ALF |  | 0.0050 | 0.0030 | 0.0030 | **0.9890** |  |  |  |  |  |  |  |  |  | 0.7330 | 0.2670 |
| 71-BARREA |  | 0.0070 | 0.0040 | 0.0030 | **0.9860** |  |  |  |  |  |  |  |  |  | 0.7167 | 0.2833 |
| 72-VALCO |  | 0.0050 | 0.0030 | 0.0030 | **0.9890** |  |  |  |  |  |  |  |  |  | **0.7662** | 0.2338 |
| 73-RIONERO |  | 0.0102 | 0.0030 | 0.0030 | **0.9838** |  |  |  |  |  |  |  |  |  | 0.7238 | 0.2762 |
| 74-SANNIO |  | 0.0050 | 0.0032 | 0.0040 | **0.9878** |  |  |  |  |  |  |  |  |  | **0.8068** | 0.1932 |
| 75-MIRA |  | 0.0067 | 0.0040 | 0.0030 | **0.9863** |  |  |  |  |  |  |  |  |  | 0.7552 | 0.2448 |
| 76-FONT |  | 0.0050 | 0.0040 | 0.0030 | **0.9880** |  |  |  |  |  |  |  |  |  | **0.7950** | 0.2050 |
| 77-MAS |  | 0.0070 | 0.0030 | 0.0030 | **0.9870** |  |  |  |  |  |  |  |  |  | **0.8357** | 0.1643 |
| 78-ALTILIA |  | 0.0063 | 0.0040 | 0.0030 | **0.9867** |  |  |  |  |  |  |  |  |  | 0.7202 | 0.2798 |
| 79-CROCE |  | 0.0050 | 0.0043 | 0.0030 | **0.9877** |  |  |  |  |  |  |  |  |  | **0.8918** | 0.1082 |
| 80-CIRCE |  | 0.0148 | 0.0090 | 0.0042 | **0.9720** |  |  |  |  |  |  |  |  |  | **0.7705** | 0.2295 |
| 81-CAVOTI |  | 0.0050 | 0.0030 | 0.0040 | **0.9880** |  |  |  |  |  |  |  |  |  | **0.8432** | 0.1568 |
| 82-MOLARA |  | 0.0050 | 0.0078 | 0.0030 | **0.9842** |  |  |  |  |  |  |  |  |  | **0.8905** | 0.1095 |
| 83-MONTEC |  | 0.0050 | 0.0053 | 0.0217 | **0.9680** |  |  |  |  |  |  |  |  |  | **0.8702** | 0.1298 |
| 84-ARIANO |  | 0.0050 | 0.0030 | 0.0060 | **0.9860** |  |  |  |  |  |  |  |  |  | **0.8710** | 0.1290 |
| 85-CASOLLA |  | 0.0040 | 0.0020 | 0.0020 | **0.9920** |  |  |  |  |  |  |  |  |  | **0.8368** | 0.1632 |
| 86-TUFINO |  | 0.0047 | 0.0030 | 0.0030 | **0.9893** |  |  |  |  |  |  |  |  |  | 0.6277 | 0.3723 |
| 87-SERINO |  | 0.0058 | 0.0030 | 0.0030 | **0.9882** |  |  |  |  |  |  |  |  |  | **0.8885** | 0.1115 |
| 88-MONT |  | 0.0070 | 0.0032 | 0.0030 | **0.9868** |  |  |  |  |  |  |  |  |  | **0.7753** | 0.2247 |
| 89-RAGUSA |  | 0.0197 | 0.0080 | 0.0040 | **0.9683** |  |  |  |  |  |  |  |  |  | 0.4812 | 0.5188 |
| 90-ANAPO |  | 0.0135 | 0.0032 | 0.0030 | **0.9803** |  |  |  |  |  |  |  |  |  | **0.7918** | 0.2082 |
| 91-BIVONA |  | 0.0060 | 0.0030 | 0.0030 | **0.9880** |  |  |  |  |  |  |  |  |  | **0.7278** | 0.2722 |
|  |  |  |  |  |  |  |  |  |  |  |  |  |  |  |  |  |
