# Supplementary material for: Rethinking the history of common walnut (Juglans regia L.) in Europe: Its origins and human interactions
Source: PLoS One. 2017 Mar 3;12(3):e0172541. doi: 10.1371/journal.pone.0172541 (PMC5336217; doi:10.1371/journal.pone.0172541)
Supplement: S9 Table — Model checking of the most likely scenario inferred in the first stage (scenario 5a) and in the second stage (scenario 6b) of DIYABC analysis. Deviation of summary statistics computed for the observed dataset from the posterior predictive distribution of the most likely scenario is given as a proportion of data sets simulated from the posterior having a value lower than the observed dataset (Ssimul. < Sobs.). (DOCX) [file pone.0172541.s012.docx]

**S9 Table. Model checking of the most likely scenario inferred in the first and second stage.** Model checking of the most likely scenario inferred in the first stage (scenario 5a) and in the second stage (scenario 6b) of DIYABC analysis. Deviation of summary statistics computed for the observed dataset from the posterior predictive distribution of the most likely scenario is given as a proportion of data sets simulated from the posterior having a value lower than the observed dataset (*S_simul._ < S_obs_*_._).

| **Stage 1** |  |  |  |
| --- | --- | --- | --- |
| Summary statistics (*S*) | Observed *S* | Probability  (S_simul._  < S_obs._) |  |
| NAL_1_1 | 6.5000 | 0.6270 |  |
| NAL_1_2 | 8.0000 | 0.6555 |  |
| NAL_1_3 | 7.5714 | 0.6485 |  |
| NAL_1_4 | 7.4286 | 0.5270 |  |
| HET_1_1 | 0.6747 | 0.7420 |  |
| HET_1_2 | 0.6666 | 0.6780 |  |
| HET_1_3 | 0.6343 | 0.5980 |  |
| HET_1_4 | 0.6038 | 0.4485 |  |
| VAR_1_1 | 15.0415 | 0.9510 | (*) |
| VAR_1_2 | 13.4998 | 0.9320 |  |
| VAR_1_3 | 11.0202 | 0.8970 |  |
| VAR_1_4 | 8.0746 | 0.8050 |  |
| FST_1_1&2 | 0.0533 | 0.4730 |  |
| FST_1_1&3 | 0.0821 | 0.6085 |  |
| FST_1_1&4 | 0.1293 | 0.9205 |  |
| FST_1_2&3 | 0.0328 | 0.9910 | (**) |
| FST_1_2&4 | 0.0559 | 1.0000 | (***) |
| FST_1_3&4 | 0.0267 | 0.9885 | (*) |
| LIK_1_1&2 | 1.5327 | 0.8390 |  |
| LIK_1_1&3 | 1.7628 | 0.7700 |  |
| LIK_1_1&4 | 2.3269 | 0.9655 | (*) |
| LIK_1_2&1 | 1.4643 | 0.6615 |  |
| LIK_1_2&3 | 1.2531 | 0.8240 |  |
| LIK_1_2&4 | 1.5518 | 0.9810 | (*) |
| LIK_1_3&1 | 1.4901 | 0.5925 |  |
| LIK_1_3&2 | 1.0556 | 0.6125 |  |
| LIK_1_3&4 | 1.1006 | 0.7480 |  |
| LIK_1_4&1 | 1.5030 | 0.6125 |  |
| LIK_1_4&2 | 0.9902 | 0.4930 |  |
| LIK_1_4&3 | 0.8664 | 0.3165 |  |
| DAS_1_1&2 | 0.2911 | 0.2920 |  |
| DAS_1_1&3 | 0.2880 | 0.3120 |  |
| DAS_1_1&4 | 0.2706 | 0.2365 |  |
| DAS_1_2&3 | 0.3275 | 0.2940 |  |
| DAS_1_2&4 | 0.3285 | 0.2980 |  |
| DAS_1_3&4 | 0.3641 | 0.4255 |  |
|  |  |  |  |
| **Stage 2** |  |  |  |
| Summary statistics (*S*) | Observed *S* | Probability  (S_simul._  < S_obs._) |  |
| NAL_1_1 | 6.5000 | 0.4960 |  |
| NAL_1_2 | 8.0000 | 0.7705 |  |
| NAL_1_3 | 7.5714 | 0.5115 |  |
| NAL_1_4 | 7.4286 | 0.4145 |  |
| HET_1_1 | 0.6747 | 0.5810 |  |
| HET_1_2 | 0.6666 | 0.6945 |  |
| HET_1_3 | 0.6343 | 0.5685 |  |
| HET_1_4 | 0.6038 | 0.4910 |  |
| VAR_1_1 | 15.0415 | 0.9335 |  |
| VAR_1_2 | 13.4998 | 0.9060 |  |
| VAR_1_3 | 11.0202 | 0.8650 |  |
| VAR_1_4 | 8.0746 | 0.7690 |  |
| FST_1_1&2 | 0.0533 | 0.4015 |  |
| FST_1_1&3 | 0.0821 | 0.6015 |  |
| FST_1_1&4 | 0.1293 | 0.7820 |  |
| FST_1_2&3 | 0.0328 | 0.9680 | (*) |
| FST_1_2&4 | 0.0559 | 0.9630 | (*) |
| FST_1_3&4 | 0.0267 | 0.9880 | (*) |
| LIK_1_1&2 | 1.5327 | 0.7550 |  |
| LIK_1_1&3 | 1.7628 | 0.8320 |  |
| LIK_1_1&4 | 2.3269 | 0.8835 |  |
| LIK_1_2&1 | 1.4643 | 0.7020 |  |
| LIK_1_2&3 | 1.2531 | 0.8915 |  |
| LIK_1_2&4 | 1.5518 | 0.9505 | (*) |
| LIK_1_3&1 | 1.4901 | 0.6510 |  |
| LIK_1_3&2 | 1.0556 | 0.5800 |  |
| LIK_1_3&4 | 1.1006 | 0.7235 |  |
| LIK_1_4&1 | 1.5030 | 0.6270 |  |
| LIK_1_4&2 | 0.9902 | 0.4125 |  |
| LIK_1_4&3 | 0.8664 | 0.3490 |  |
| DAS_1_1&2 | 0.2911 | 0.3915 |  |
| DAS_1_1&3 | 0.2880 | 0.3980 |  |
| DAS_1_1&4 | 0.2706 | 0.3210 |  |
| DAS_1_2&3 | 0.3275 | 0.3045 |  |
| DAS_1_2&4 | 0.3285 | 0.3145 |  |
| DAS_1_3&4 | 0.3641 | 0.4110 |  |
|  |  |  |  |

The probability Prob. (*S_simul._ < S_obs_*_._) given for each summary statistics was computed from 1,000 virtual data sets simulated from the posterior distributions of parameters obtained under a given scenario. Corresponding tail-area probabilities (*p*-values) were obtained as Prob. (*S_simul._ < S_obs_*_._) and 1.0 - Prob. (*S_simul._ < S_obs_*_._) for Prob. (*S_simul._ < S_obs_*_._) $\leq$ 0.5 and > 0.5, respectively. For one sample summary statistics we used: mean number of alleles (NAL), mean genetic diversity (HET) and mean allele size variance (VAR). For two sample summary statistics we used: Wright’s F_ST_ (F_ST_), mean individual assignment likelihoods (LIK) and mean index of classification (DAS).*,**, *** = tail-area probability < 0.05, < 0.01 and < 0.001, respectively.
